# Ligand Relay for Nickel‐Catalyzed Decarbonylative Alkylation of Aroyl Chlorides

**DOI:** 10.1002/advs.202306923

**Published:** 2023-12-13

**Authors:** Tian‐Zhang Wang, Yu‐Qiu Guan, Tian‐Yu Zhang, Yu‐Feng Liang

**Affiliations:** ^1^ School of Chemistry and Chemical Engineering Shandong University Jinan 250100 China

**Keywords:** alkylation, aroyl chlorides, cross electrophiles coupling, decarbonylation, ligand relay, nickels

## Abstract

Transition metal‐catalyzed direct decarboxylative transformations of aromatic carboxylic acids usually require high temperatures, which limit the substrate's scope, especially for late‐stage applications. The development of the selective decarbonylative of carboxylic acid derivatives, especially the most fundamental aroyl chlorides, with stable and cheap electrophiles under mild conditions is highly desirable and meaningful, but remains challenging. Herein, a strategy of nickel‐catalyzed decarbonylative alkylation of aroyl chlorides via phosphine/nitrogen ligand relay is reported. The simple phosphine ligand is found essential for the decarbonylation step, while the nitrogen ligand promotes the cross‐electrophile coupling. Such a ligand relay system can effectively and orderly carry out the catalytic process at room temperature, utilizing easily available aroyl chlorides as an aryl electrophile for reductive alkylation. This discovery provides a new strategy for direct decarbonylative coupling, features operationally simple, mild conditions, and excellent functional group tolerance. The mild approach is applied to the late‐stage methylation of various pharmaceuticals. Extensive experiments are carried out to provide insights into the reaction pathway and support the ligand relay process.

## Graphical abstract

A nickel‐catalyzed decarbonylative cross electrophiles coupling of aroyl chlorides with alkyl halides/pseudohalides via phosphine/nitrogen ligand relay is demonstrated. The phosphine ligand is useful in decarbonylation process and the nitrogen ligand is essential for cross‐coupling step. Such a ligand relay system can effectively and orderly carry out the catalytic process at room temperature.

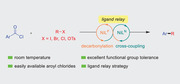

## 1 Introduction

Nickel‐catalyzed coupling reactions have been greatly developed in recent decades, mainly due to the fact that the metal element nickel has five oxidation states and is therefore good at transferring single‐electron, as well as its low price and widespread existence.^[^ ^1^ ^]^ The addition of suitable ligands can coordinate with nickel catalysts, thereby reducing the reaction activation energy barrier, improving catalytic activity and reaction efficiency, and broadening the types of reactions, so the discovery of ligands promotes the development of nickel‐catalyzed reactions.^[^ ^2^ ^]^ The selectivity of the reaction can be switched by changing the steric resistance and electrical properties of the ligand, which has become one of the common methods of organic chemistry, hence the development of new ligands is a very interesting and useful work.^[^ ^3^ ^]^ However, innovative ligands often require multi‐step synthesis and multiple structural adjustments, especially complex ligands with multiple coordination centers are difficult to prepare. The commercially available ligands can be obtained via simple approaches, providing more practicality for the catalytic reaction. Consequently, one of the simple and practical ways is to add two ligands to the catalytic system, in which the former ligand metal complex for promoting the first‐step process would undergo ligand exchange after adding the latter ligand to catalyze the subsequent second‐step reaction.^[^ ^4^ ^]^ Recently, these two ligands/single metal strategy has been applied to enhance the catalytic performance in the development of palladium,^[^ ^5^ ^]^ cobalt,^[^ ^6^ ^]^ and copper^[^ ^7^ ^]^ catalyzed new reactions. Weix,^[^ ^8^ ^]^ Fu,^[^ ^9^ ^]^ Wang,^[^ ^10^ ^]^ Zhu,^[^ ^11^ ^]^ and Shu^[^ ^12^ ^]^ observed the phosphine and nitrogen dual ligand effects in nickel‐catalyzed reductive coupling reactions. Recently, Sevov developed phosphine‐nitrogen ligand exchange for nickel‐catalyzed electroreductive coupling, revealing that the Ni^0^(phosphine) complex could undergo 2e^−^ oxidative addition with aryl electrophiles and Ni^II^(nitrogen) complex was crucial for 1e^−^ reaction with alkyl electrophiles.^[^ ^13^ ^]^ Despite the remarkable advances, the concept of two ligands/single metal is still in its infancy, and further potential is still to be disclosed.

**Scheme 1:**
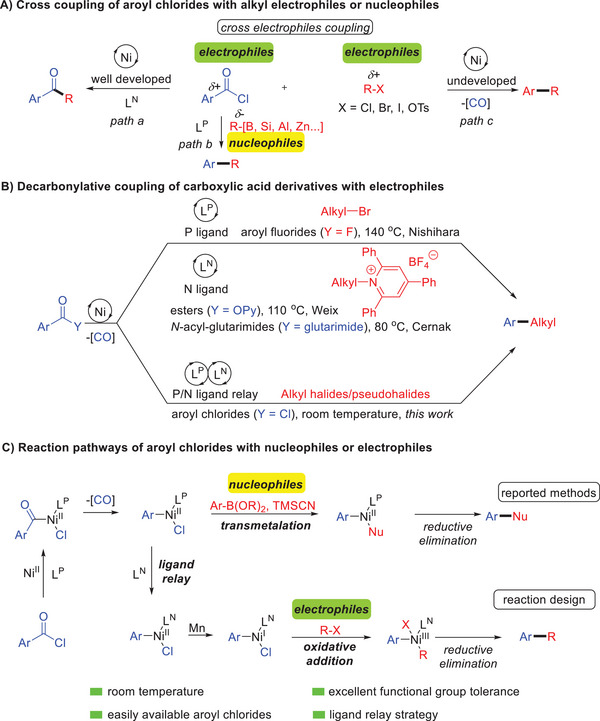
Ni‐catalyzed cross coupling of carboxylic acid derivatives.

Carboxylic acids and their derivatives have aroused wide interest in organic synthesis due to their ready availability, abundance, and environmental friendliness.^[^ ^14^ ^]^ The decarboxylative coupling of aliphatic carboxylic acids via the alkyl radical intermediate has been well demonstrated in the SET pathway,^[^ ^15^ ^]^ but the direct decarboxylative transformations of aromatic carboxylic acids usually performed under high temperature, resulting in narrow substrates scope.^[^ ^16^ ^]^ In this context, aroyl chlorides, one of the most widespread and fundamental carboxylic acid derivatives, have been developed as convenient surrogates for conventional coupling partners.^[^ ^17^, ^18^ ^]^ Cross electrophiles coupling, a catalytic method that couples two different readily accessible carbon electrophiles under reductive conditions that differs from the conventional nucleophiles/electrophiles coupling methods, has become a powerful tool in organic synthesis for selective construction of a diverse range of C─C bonds.^[^ ^19^, ^20^ ^]^ In this scenario, the Earth‐abundant nickel‐catalyzed nitrogen ligands promoted cross electrophiles coupling of aroyl halides with organic halides permitting access to ketones have recently been studied intensively by Weix,^[^ ^21^ ^]^ Gong,^[^ ^22^ ^]^ and Reisman^[^ ^23^ ^]^ (**Scheme** **1**A, path a). On the other hand, the decarbonylative coupling of aromatic carboxylic acids derivatives with nucleophiles,^[^ ^24^, ^25^ ^]^ such as organo‐boron, silicon, aluminum, and zinc compounds, have been developed by Sanford,^[^ ^26^ ^]^ Itami,^[^ ^27^ ^]^ Szostak,^[^ ^28^ ^]^ Rueping,^[^ ^29^ ^]^ and Nishihara^[^ ^30^ ^]^ (Scheme 1A, path b). Despite these reported advances, decarbonylative coupling of aroyl chlorides with electrophiles, such as easily available alkyl halides/pseudohalides, via the loss of CO is of great interest, but has been virtually unexplored (Scheme 1A, path c).

The formation of C(sp^2^)─C(sp^3^) bonds has been widely studied and become one of the most important processes in organic synthesis chemistry, and many reductive coupling reactions to construct C(sp^2^)─C(sp^3^) bonds have been developed with cheap transition metals, especially nickel.^[^ ^31^ ^]^ Nishihara disclosed a nickel‐catalyzed phosphine ligand promoted decarbonylative cross‐electrophile coupling of aroyl fluorides with alkyl bromides at 140 ^o^C.^[^ ^32^ ^]^ Recently, Weix^[^ ^33^ ^]^ and Cernak^[^ ^34^ ^]^ independently developed nickel‐catalyzed nitrogen ligand promoted decarbonylative coupling of 2‐pyridyl esters or *N*‐acyl‐glutarimides with *N*‐alkyl pyridinium salts at 80–110 °C, forming C(sp^2^)─C(sp^3^) bonds (Scheme 1B). Since the starting materials, aroyl fluorides, esters, and imides, in the above‐mentioned reactions are always prepared in an additional step, a mild protocol of decarbonylative alkylation of aroyl chlorides is highly desirable because aroyl chlorides are commercially available or could be easily prepared from the abundant carboxylic acids in one‐pot protocol. We noticed that aromatic carboxylic acid derivatives could be decarbonylated as an aryl electrophile^[^ ^25^, ^26^, ^27^, ^28^, ^29^ ^]^ in nickel‐catalyzed nucleophiles‐electrophiles coupling with phosphine ligands, while the nitrogen ligands failed to promote the reactions (Scheme 1C, up). By contrast, nitrogen ligands are critical in cross‐electrophile coupling reactions, but phosphine ligands always show low efficiency.^[^ ^19^, ^20^, ^21^, ^22^, ^23^ ^]^ Inspired by the above elegant outcomes, we envisioned that the use of phosphine ligands can effectively promote the decarbonylative step of aroyl chlorides, and the nitrogen ligand is conducive to the construction of chemical bonds in the following cross‐coupling, establishing an effective channel for decarbonylative cross electrophiles coupling (Scheme 1C, down). As a consequence of our efforts, we herein disclose a nickel‐catalyzed decarbonylative cross electrophiles coupling of aroyl chlorides with alkyl halides/pseudohalides by the synergistic relay of the phosphine/nitrogen ligands to construct C(sp^2^)─C(sp^3^) bonds. Salient features of our findings include; a) a novel ligand relay strategy utilizing two simple ligands, with the phosphine ligand for decarbonylation and the nitrogen ligand for cross‐coupling; b) the first decarbonylative alkylation of aroyl chlorides, readily available aroyl derivatives, with alkyl electrophiles; c) key mechanistic insights by experiments to support the proposed ligand relay mechanism; d) mild room temperature condition and excellent functional group tolerance, especially for complex molecules.

## 2 Results and Discussion

We commenced the study with 1‐naphthoyl chloride **1a** and (4‐bromobutyl) benzene **2a** as standard substrates and systematically screened the influence of reaction parameters (**Table** **1**). To our delight, after considerable optimization, the desired decarbonylative alkylation product **3aa** was obtained in an optimal 78% isolated yield when 1,6‐bis(diphenylphosphino)hexane (dpph) and 4,4′‐di‐*tert*‐butyl‐2,2′‐ bipyridine (dtbpy) were used as cooperative ligands along with a combination of NiBr_2_ at room temperature, and the acylation byproduct **4aa** was observed in trace amount (entry 1). The use of CoCl_2_ and Fe(OTf)_3_ proved unsuccessful (entry 2), and other nickel catalysts did not yield better results (entry 3). It was found that when nitrogen ligand was used in the absence of phosphine ligand, the yield of **3aa** decreased and the acylation product **4aa** increased significantly (entry 4). Meanwhile, most of the starting materials were recovered without nitrogen ligands because of the stop of cross electrophiles coupling step (entry 5), fully demonstrating the importance of both ligands in the yields and chemoselectivity. Next, we focused on the detailed screening of available and simple ligands, finding that dpph is the best phosphine ligand and dtbpy is the optimal nitrogen ligand choice. In addition, the use of Zn in lieu of Mn resulted in a lower yield (entry 6). Among many additives tested, NaI was found to be the best, while KI and LiCl were proved less effective (entries 7–9).^[^ ^35^ ^]^ The effects of solvents were next carefully examined. DMF and Dioxane as mixed solvents worked better than others, indicating that the choice of solvents is critical for this transformation (entries 10–11). Furthermore, the temperature turned out to have a crucial effect on the outcome of this transformation. The reaction at lower temperatures occurred slowly, and the byproduct from home‐coupling of both starting materials increased leading to inferior yields (entries 12–13). It should be pointed out that the metal catalyst, ligands, and reducing agent are all crucial in control experiments, with no reaction occurring in their absence (entry 14).

**Table 1:** Establishing the decarbonylative alkylation.

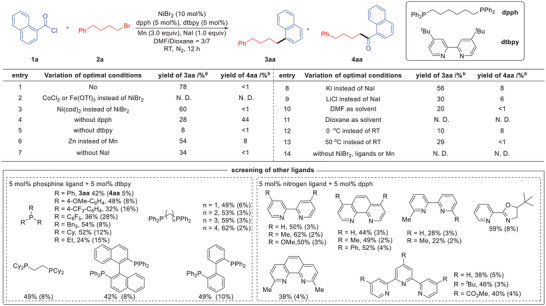							

^a)^ Reaction conditions: **1a** (0.2 mmol), **2a** (0.4 mmol), catalyst (10 mol %), P ligand (5 mol %), N ligand (5 mol %), reductant (3.0 equiv), additive (1.0 equiv) and solvent (1.0 mL), at room temperature for 12 h under N_2_;

^b)^ GC yields. dpph = 1,6‐bis(diphenylphosphino)hexane, dtbpy = 4,4′‐di‐*tert*‐butyl‐2,2′‐bipyridine, DMF = *N*,*N*‐dimethylformamide, Dioxane = 1,4‐dioxacyclohexane, OTf = Trifluoromethanesulfonate, cod = 1,5‐cyclooctadiene.

N. D. = not detected.

**Scheme 2:**
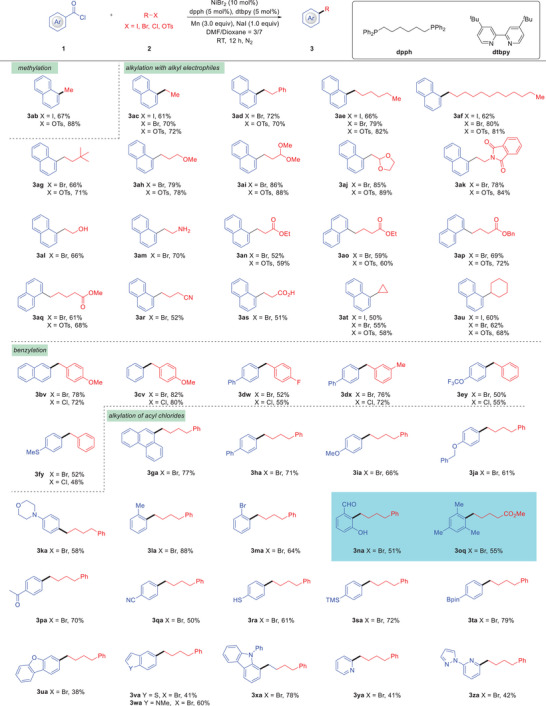
Scope of decarbonylative alkylation of acid chlorides with alkyl electrophiles.

After establishing the best reaction conditions, the substrate scope of this decarbonylative alkylation was then investigated (**Scheme** **2**). To our delight, not only alkyl bromides proceed smoothly, but also the alkyl iodides/chlorides/sulfonates are suitable substrates, delivering the corresponding alkylated products **3ab**‐**3za** in moderate to excellent yields. Significantly, the challenging methylation worked well with MeI or MeOTs as the reagent. In addition, a variety of electron‐neutral as well as electron‐rich and ‐poor alkyl electrophiles underwent conversion without affecting the substituents. Gratifyingly, a wide range of functional groups including ether, acetal, phthalimide, ester, and cyano were all readily accommodated. Significantly, the reaction can also occur smoothly with the functional group containing free hydrogen including ‐OH (**3al**), ‐NH_2_ (**3am**), and ‐CO_2_H (**3as**). Secondary alkyl bromides such as cyclopropyl bromide and cyclohexyl bromide are also compatible (**3at‐3au**). Moreover, benzyl halides were also suitable for the decarbonylative alkylation to afford the desired products (**3bv‐3fy**). A series of aroyl chlorides bearing electron‐donating and ‐withdrawing functional groups could be transformed into desired products (**3ga‐3za**) efficiently. Remarkably, sterically hindered aroyl chloride, *ortho* substituents were well tolerated regardless of the electronic nature of the substituents (**3la‐3ma**). Of particular note, the highly hindered aroyl chlorides, such as 2‐formyl‐6‐hydroxybenzoyl chloride and 2,4,6‐trimethylbenzoyl chloride, also worked well (**3na‐3oa**).^[^ ^33^, ^34^ ^]^ Notably, the unprotected group ‐SH (**3ra**) was compatible and valuable ‐TMS (**3sa**) and ‐Bpin (**3ta**) groups remained intact. Moreover, heteroatom‐containing aroyl chlorides such as furan, thiophene, indole, carbazole, pyridine, and imidazole were productive in the transformation (**3ua**‐**3za**). One limitation of this method was observed in the reactions employing aliphatic acid chlorides, which failed to deliver the desired products.

**Scheme 3:**
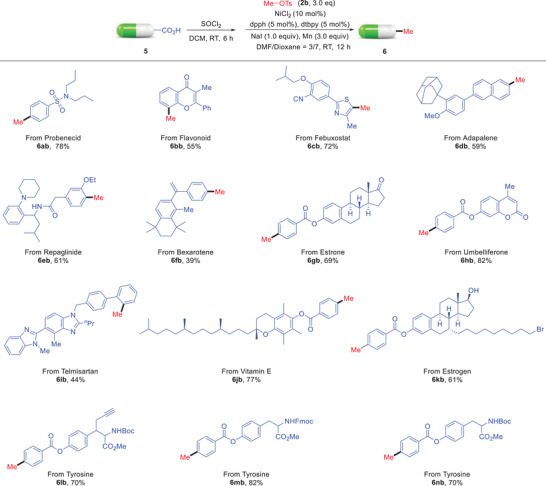
Decarbonylative methylation of complex molecules.

Encouraged by the above results, we next examined the late‐stage modification of the acid‐containing complex molecules with this robustness protocol (**Scheme** **3**). Gratifyingly, the valuable methylation^[^ ^36^ ^]^ of the aroyl chlorides, in situ prepared from corresponding structurally complex carboxylic acids, proceeded smoothly in all cases to give the corresponding products in moderate to good yields. Biologically active molecules such as the antimicrobial drug Probenecid (**6ab**), antioxidant Flavonoid (**6bb**), gout drug Febuxostat (**6cb**), dermatologic drug Adapalene (**6db**), oral antihyperglycemic agent Repaglulinide (**6eb**), antitumor drug Bexarotene (**6fb**), and antihypertensive drug Telmisartan (**6ib**) were identified as viable substrates. The complexes from Estrone (**6gb**), Umbelliferone (**6hb**), Vitamin E (**6jb**), Estrogen (**6kb**), and Tyrosine (**6lb**‐**6nb**) were all successfully converted into the methylation product, demonstrating good functional group tolerance and practicability of the present transformation.

**Figure 1:**
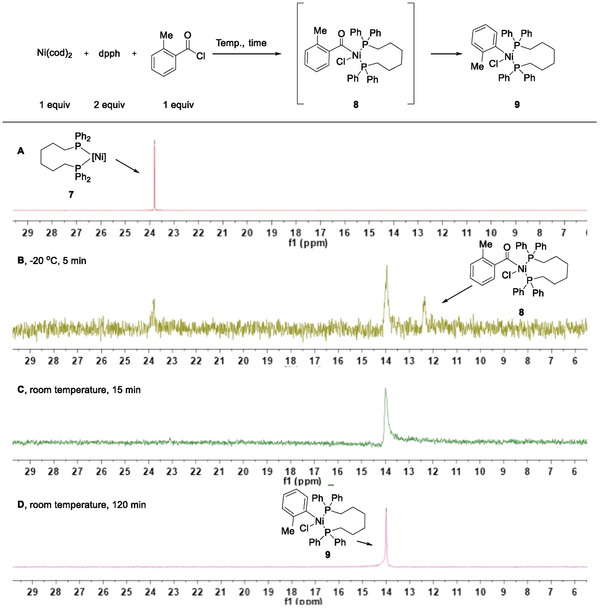
Stoichiometric reactions of Ni(cod)_2_, triphenylphosphine with *o*‐toluoyl chloride. The ^31^P NMR spectra of (A) [Ni^0^](dpph) **7**, (B) reaction at −20 °C for 5 min, (C) reaction at room temperature for 5 min, (D) aryl nickel specie **9**

To gain insights into the catalytic cycle, a series of mechanistic experiments were conducted to illuminate possible mechanisms. First, the stoichiometric reaction of Ni(cod)_2_, dpph with *o*‐toluoyl chloride in DMF was monitored by the ^31^P NMR (**Figure** **1**).^[^ ^30^ ^]^ The Ni^0^(dpph) complex **7**, prepared from Ni(cod)_2_ and dpph (Figure 1A), could be observed when the reaction was carried out at −20 °C for 5 min, as well as the oxidative addition acyl‐Ni^II^‐Cl intermediate **8** and decarbonylation aryl nickel specie **9** (Figure 1B). Other complexes almost disappeared after 15 min at room temperature (Figure 1C), and the amount of aryl nickel species **9** increased rapidly (Figure 1D).^[^ ^37^ ^]^ These results suggested that both oxidative addition and subsequent decarbonylation can readily occur rapidly with the assistance of phosphine ligands. The highly reactive intermediate **8** couldn't be isolated as pure compound at this moment even at low temperatures because of the rapid decarbonylation to give complex **9**, but when the phosphine ligand was changed to PEt_3_,^[^ ^30a^ ^]^ the corresponding acyl‐Ni^II^‐Cl intermediate could be obtained as a stable compound, which could undergo decarbonylation and further reaction with alkyl bromide (see Supporting Information for more details).

**Scheme 4:**
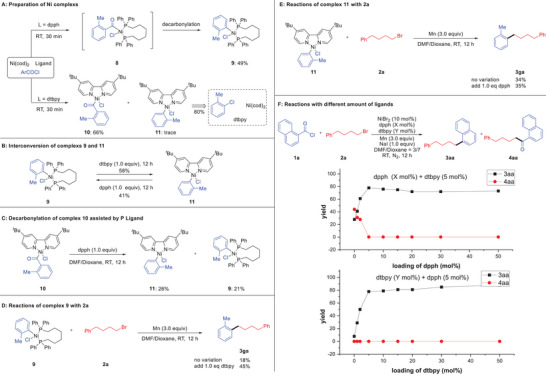
Studies of the influences of ligands.

The stoichiometric reaction of Ni(cod)_2_, *o*‐toluoyl chloride (**2l**) with dpph at room temperature for 30 min afforded the decarbonylation complex **9** in 49% yield, while the oxidative addition complex **10** could be observed in the reaction with dtbpy in 66% yield, along with trace amount of decarbonylation complex **11**, which could be prepared via the reaction with *o*‐tolylchloride as starting material (**Scheme** **4**A).^[^ ^38^ ^]^ On the other hand, complexes **9** and **11** could be transformed into each other via the reaction with the corresponding ligand, indicating that the phosphine and nitrogen ligands could easily undergo ligand exchange with the nickel catalyst (Scheme 4B). Additionally, the complex **10** could undergo decarbonylation through the reaction with dpph, generating complex **9** and **11** in 21% and 26% yield, respectively (Scheme 4C). Next, the reaction of complex **9** with **2a** afforded the product **3ga** an 18% yield, which could be improved to 45% through the addition of the dtbpy ligand (Scheme 4D). In stark contrast, the dpph ligand failed to increase the yield in the reaction of complex **11** with **2a** (Scheme 4E). These results clearly indicated that the phosphine ligand is responsible in the first decarbonylation step and useless for the second cross‐coupling step, while the nitrogen ligand is not useful for decarbonylation but crucial for the following cross‐coupling process. Moreover, the experiments with different loading of ligands suggested that the employment of phosphine ligand could significantly decrease the amount of undesired acylation product **4aa**, and with 5 mol% of dpph as the optimal choice and an excessive amount of ligand will not poison the nickel catalyst, further supporting that the phosphine could accelerate the decarbonylation process (Scheme 4F, up). On the other hand, the by‐product **4aa** could be suppressed in trace amounts with 5 mol% of dpph as ligand, and the addition of nitrogen ligand could drastically improve the efficiency for the formation of **3aa** (Scheme 4F, down).

**Scheme 5:**
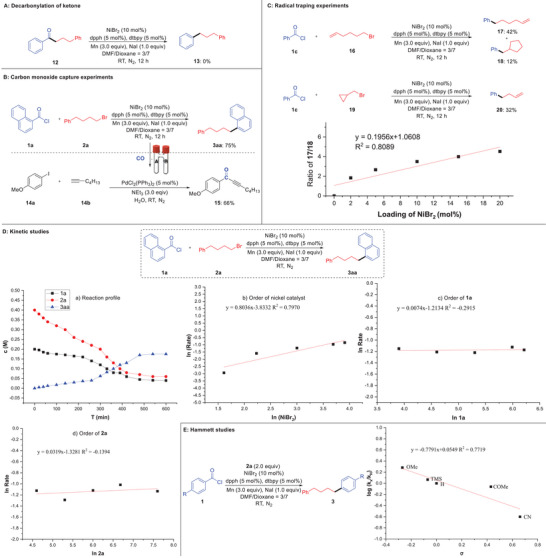
Mechanistic experiments.

The reaction of 1,4‐diphenylbutan‐1‐one **12** under standard conditions failed to afford decarbonylation product **13**, excluding a procedure of decarbonylation of acylation intermediate for this transformation (**Scheme** **5**A). To demonstrate the departure of CO, a carbon monoxide‐capture experiment was designed, and performed in a sealed two‐tube reactor (Scheme 5B). As depicted, the decarbonylative alkylation reaction was carried out in tube A, and the carbonylative alkynylation was conducted in tube B to consume the CO gas produced in tube A. Delightedly, the carbonylation product **15** was obtained in 66% yield, further indicating that this reaction involves a decarbonylation process.^[^ ^39^ ^]^ The reaction of 6‐bromo‐1‐ene **16** can produce the linear alkene product **17** along with ring‐closing product **18**. Additionally, a radical clock experiment reaction employing (bromomethyl) cyclopropane **19** was examined under the optimum reaction conditions, providing a ring‐opening product **20** exclusively (Scheme 5C). These outcomes suggested that an alkyl radical is involved in the reaction pathway. Furthermore, it was found that the ratio of **17** to **18** has a certain linear relationship with the loading of the nickel catalyst, which suggests that the free radical chain reaction mechanisms are more possible than cage radical mechanisms (Scheme 5C).^[^ ^40^ ^]^ Kinetic studies were then performed to explore the possible rate‐determining step (Scheme 5D). A first‐order dependence with respect to the concentration of nickel catalyst and zero‐order for both reagents were observed, indicating that an intermediate assembled from both reagents might be involved in the turnover‐limiting step. Finally, a negative *ρ* value was obtained from Hammett studies,^[^ ^41^ ^]^ suggesting that reductive elimination is the rate‐determining step, which is consistent with the findings of kinetic studies (Scheme 5E).

**Scheme 6:**
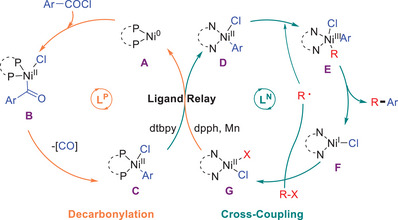
Proposed ligand relay mechanism.

Although further mechanistic insights are clearly warranted, on the basis of the above results and previous studies,^[^ ^13^, ^19^, ^20^, ^21^, ^22^, ^23^, ^24^, ^25^, ^26^, ^27^, ^28^, ^29^, ^30^, ^31^, ^32^, ^33^, ^34^ ^]^ a proposed mechanism is outlined (**Scheme** **6**). Initially, the active Ni(0) species **A** was formed from the reaction Ni(II) catalyst with manganese powder and phosphine ligand. Then intermediate acyl‐Ni(II)‐Cl **B** could be obtained via oxidative addition, followed by fast decarbonylation to give aryl‐Ni(II)‐Cl **C**.^[^ ^26^, ^27^, ^28^, ^29^, ^30^ ^]^ A key step of ligand exchange between phosphine and nitrogen ligands can generate Ni(II) complex **D**, which would react with a free alkyl radical to form Ni(III) complex **E**. Subsequently, the rate‐limiting reductive elimination would deliver the aryl‐alkyl product and Ni(I) complex **F**, which could react with the alkyl halides to regenerate an alkyl radical and intermediate **G**.^[^ ^13^ ^]^ Finally, the ligand exchange and reduction would regenerate complex **A**.

## 3 Conclusion

In conclusion, we have developed a nickel‐catalyzed decarbonylative alkylation through bidentate phosphine/nitrogen ligand relay, utilizing readily available aroyl chlorides as aryl electrophiles for the cross‐electrophile coupling with alkyl halides/pseudohalides. The transformations are performed at room temperature due to the cooperative phosphine/nitrogen system, constructing an aryl‐alkyl bond tolerating a wide range of functional groups, including free amine, alcohol, acid, phenol, and thiophenol. A phenomenon of ligand relay is found that phosphine ligand plays a key role in the decarbonylation process and nitrogen ligand is essential in the cross electrophile coupling. Ligand relay could offer an opportunity as a potentially powerful strategy for the conversion of an aroyl electrophile to an aryl electrophile in metal‐catalyzed cross‐electrophile coupling. Further studies on ligand relay‐guided cross‐electrophile coupling will be continuously explored in our group.

## Conflict of Interest

The authors declare no conflict of interest.

## Supporting information

Supporting Information

## Data Availability

The data that support the findings of this study are available from the corresponding author upon reasonable request.
