# Supplementary material for: Ligand Relay for Nickel‐Catalyzed Decarbonylative Alkylation of Aroyl Chlorides
Source: Adv Sci (Weinh). 2023 Dec 13;11(9):2306923. doi: 10.1002/advs.202306923 (PMC10916626; doi:10.1002/advs.202306923)
Supplement: Supplementary file 1 — Supporting Information [file ADVS-11-2306923-s001.pdf]

ADVANCED SCIENCE
Open Access

# Supporting Information

for *Adv. Sci.*, DOI 10.1002/advs.202306923

Ligand Relay for Nickel-Catalyzed Decarbonylative Alkylation of Aroyl Chlorides

*Tian-Zhang Wang, Yu-Qiu Guan, Tian-Yu Zhang and Yu-Feng Liang**

# *Supporting Information*

# Ligand Relay for Nickel-Catalyzed Decarbonylative Alkylation of Aroyl Chlorides

Tian-Zhang Wang, Yu-Qiu Guan, Tian-Yu Zhang and Yu-Feng Liang

School of Chemistry and Chemical Engineering, Shandong University, Jinan 250100, China

## Table of Contents

**1. General remarks ..........S2**

**2. General procedure ..........S2**

**3. Optimization of conditions..........S3**

**4. Characterization data..........S8**

**5. Mechanistic studies..........S41**

**6. References...........S63**

**7. NMR spectra for products ..........S65**

# 1. General remarks

$^1H$ NMR, $^{13}C$ NMR data were obtained on AVANCE III Bruker 500 MHz nuclear resonance spectrometers unless otherwise noted. Chemical shifts (in ppm) were referenced to tetramethylsilane (TMS) ($\delta$ = 0.00 ppm) in $CDCl_3$ or DMSO-$d_6$. The data of $^1H$ NMR was reported as follows: chemical shift, multiplicity (s = singlet, d = doublet, t = triplet, m = multiplet and br = broad), coupling constant (J values) in Hz and integration. $^{13}C$ NMR spectra were obtained by the same NMR spectrometers and were calibrated with $CDCl_3$ ($\delta$ = 77.16 ppm) or DMSO-$d_6$ ($\delta$ = 39.50 ppm). Flash chromatography was performed using 300-400 mesh silica gel with the indicated eluent according to standard techniques. Analytical thin-layer chromatography (TLC) was performed on pre-coated, glass-backed silica gel plates. Analysis of crude reaction mixture was done on an Agilent 7890 GC System with an Agilent 5975 Mass Selective Detector. Visualization of the developed chromatogram was performed by UV absorbance (254 nm) unless otherwise noted. High-resolution mass spectral (HRMS) data were recorded on Bruker APEX IV Fourier transform ion cyclotron resonance mass spectrometer using electrospray ionization (ESI) mode.

# 2. General procedure

**General procedure for the synthesis of acyl chlorides**[1]: A flame-dried flask was charged with a stirring bar, carboxylic acid (5.0 mmol) and dry DCM (20 mL) under $N_2$. The reaction was cooled to 0 ˚C and then treated with thionyl chloride (10.0 mmol, 2.0 equiv) and DMF (2-5 drops) over 5 min. The reaction was then allowed to gradually warm to room temperature for 3-8 h and then the reaction mixture was concentrated under reduced pressure to remove excess of thionyl chloride and DCM, affording the desired acyl chloride and were used without further purification.

**General procedure nickel-catalyzed decarbonylative alkylation of acid chlorides**: To a 10 ml Schlenk tube was added sequentially $NiBr_2$ (4.4 mg, 0.02 mmol), $(Ph_2P)_2C_4H_8$ (4.3 mg, 0.01 mmol), 4,4'-di-*tert*-butyl-2,2'-bipyridine (2.7 mg,

0.01 mmol), Mn power (32.9 mg, 0.6 mmol) and NaI (14.9 mg, 0.10 mmol), DMF (0.20 mL) was added via syringe and the mixture was stirred at room temperature for 10 min. The acyl chloride (0.20 mmol) and alkyl bromide (0.40 mmol) ware added, and then DMF (0.10 mL) and dioxane (0.70 mL) was subsequently added via syringe. The resulting solution was stirred at room temperature for 12 h under $N_2$. Then, the crude reaction mixture was diluted with ethyl acetate (10 mL) and washed with water (2.0 mL). The organic layer was dried over $Na_2SO_4$, filtered, and concentrated. The residue was purified by flash chromatography to give products.

## 3. Optimization of the reaction conditions

### 3.1 Table S1. Optimization of the catalysts

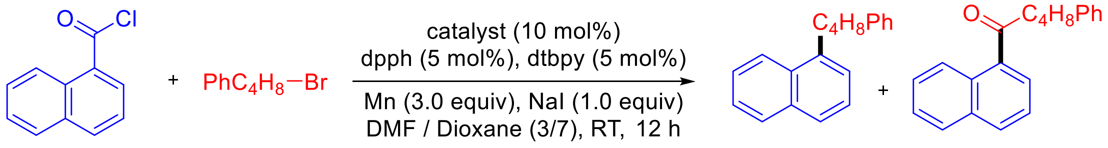


**1a** **2a** **3aa** **4aa**

| entry | catalyst | yield of 3aa /% | yield of 4aa /% |
|---|---|---|---|
| 1 | $NiI_2$ | 38 | <1 |
| 2 | $NiBr_2$ | 78 | <1 |
| 3 | $NiBr_2$·DME | 55 | <1 |
| 4 | $NiBr_2$·diglyme | 58 | <1 |
| 5 | $NiBr_2$·bpy | 32 | <1 |
| 6 | $NiCl_2$ | 41 | <1 |
| 7 | $NiCl_2$·DME | 49 | <1 |
| 8 | $NiCl_2(PPh_3)_2$ | 39 | <1 |
| 9 | $NiCl_2$(dppf) | 32 | <1 |
| 10 | $NiCl_2$(dppp) | 40 | <1 |
| 11 | $NiF_2$ | 38 | <1 |
| 12 | $Ni(acac)_2$ | 29 | <1 |
| 13 | $Ni(cod)_2$ | 60 | <1 |
| 14 | $CoCl_2$ | N. D. | N. D. |
| 15 | $Fe(OTf)_3$ | N. D. | N. D. |
| 16 | $CrCl_2$ | N. D. | N. D. |

Reaction conditions: **1a** (0.2 mmol), **2a** (0.4 mmol), catalyst (10 mol %), dpph (5 mol%), dtbpy (5 mol%), Mn (3.0 equiv), NaI (1.0 equiv) and DMF / Dioxane = 0.3 : 0.7 mL, at RT for 12 h under $N_2$. GC yields.

## 3.2 Table S2. Optimization of the phosphine ligand

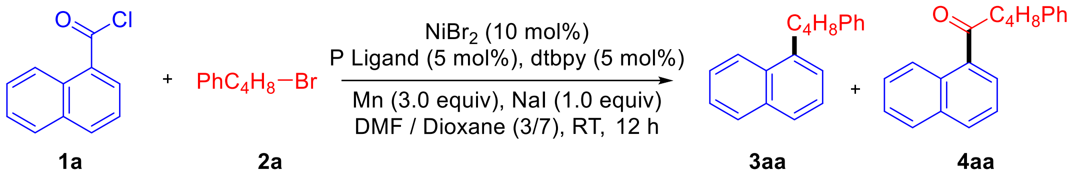


| entry | P Ligand | yield of 3aa /% | yield of 4aa /% |
|---|---|---|---|
| 1 | $PPh_3$ | 42 | 5 |
| 2 | $P(C_6F_5)_3$ | 36 | 28 |
| 3 | $P(4\text{-}CF_3\text{-}C_6H_4)_3$ | 32 | 16 |
| 4 | $P(4\text{-}Cl\text{-}C_6H_4)_3$ | 18 | 34 |
| 5 | $P(4\text{-}OMe\text{-}C_6H_4)_3$ | 48 | 8 |
| 6 | L1 | 49 | 10 |
| 7 | L2 | 42 | 8 |
| 8 | dppe | 48 | 6 |
| 9 | dppp | 53 | 3 |
| 10 | dppb | 59 | 3 |
| 11 | dppm | 62 | 2 |
| 12 | dpph | 78% | <1 |
| 13 | $PCy_3$ | 52 | 12 |
| 14 | $Ph_2PCy$ | 48 | 16 |
| 15 | $Ph_2P^tBu$ | 57 | 20 |
| 16 | $PBn_3$ | 54 | 8 |
| 17 | $P(Oct)_3$ | 58 | 18 |
| 18 | dcype | 49 | 8 |
| 19 | $P(NC_4H_8)_3$ | 21 | 38 |
| 20 | $P(NMe_2)_3$ | 26 | 42 |

L1

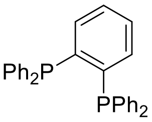


L2

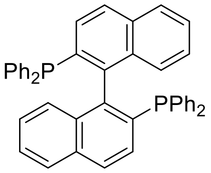


Reaction conditions: **1a** (0.2 mmol), **2a** (0.4 mmol), $NiBr_2$ (10 mol %), P Ligand (5 mol%), dtbpy (5 mol%), Mn (3.0 equiv), NaI (1.0 equiv) and DMF / Dioxane = 0.3 : 0.7 mL, at RT for 12 h under $N_2$. GC yields.

## 3.3 Table S3. Optimization of the nitrogen ligand

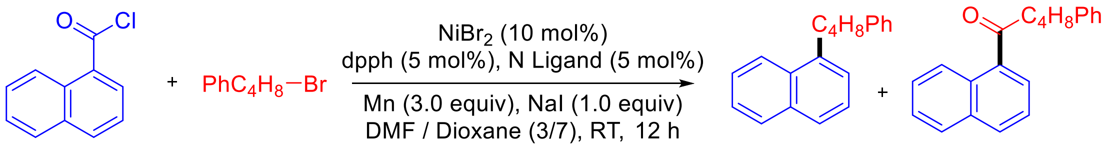


**1a** **2a** **3aa** **4aa**

| entry | N Ligand | yield of 3aa /% | yield of 4aa /% |
|---|---|---|---|
| 1 | L1 | 50 | 3 |
| 2 | L2 | 62 | 2 |
| 3 | L3 | 50 | 3 |
| 4 | L4 | 59 | 8 |
| 5 | L5 | 28 | 3 |
| 6 | L6 | 22 | 2 |
| 7 | L7 | 44 | 3 |
| 8 | L8 | 49 | 2 |
| 9 | L9 | 52 | 4 |
| 10 | L10 | 38 | 4 |
| 11 | L11 | 38 | 5 |
| 12 | L12 | 46 | 3 |
| 13 | L13 | 40 | 4 |

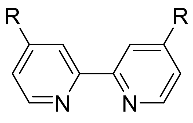


L1 R = H
L2 R = Me
L3 R = OMe

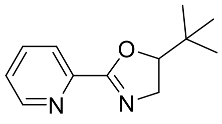


L4

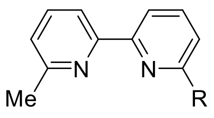


L5 R = H
L6 R = Me

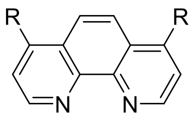


L7 R = H
L8 R = Me
L9 R = Ph

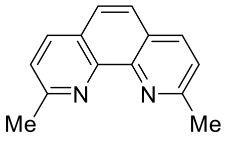


L10

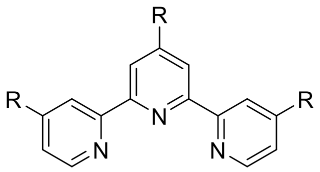


L11 R = H
L12 R = $^tBu$
L13 R = $CO_2Me$

Reaction conditions: **1a** (0.2 mmol), **2a** (0.4 mmol), $NiBr_2$ (10 mol %), dpph (5 mol%), N Ligand (5 mol%), Mn (3.0 equiv), NaI (1.0 equiv) and DMF / Dioxane = 0.3 : 0.7 mL, at RT for 12 h under $N_2$. GC yields.

## 3.4 Table S4. Optimization of additive

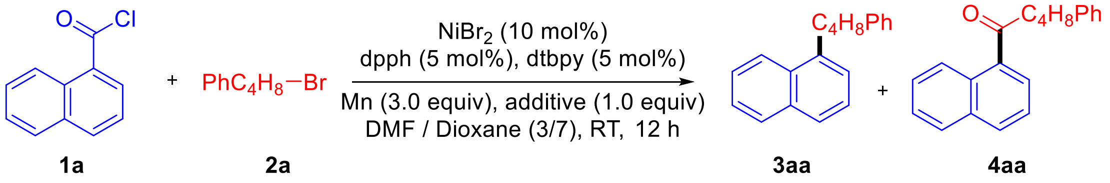


| entry | additive | yield of 3aa /% | yield of 4aa /% |
|---|---|---|---|
| 1 | LiI | 66 | <1 |
| 2 | LiBr | 28 | <1 |
| 3 | LiCl | 30 | 6 |
| 4 | LiF | 21 | <1 |
| 5 | NaI | 78 | <1 |
| 6 | $MgCl_2$ | 61 | 3 |
| 7 | Py | 44 | <1 |
| 8 | $NaBF_4$ | N. D. | N. D. |
| 9 | 1.0 eq NaI and 1.0 eq Py | 49 | <1 |
| 10 | 1.0 eq NaI and 1.0 eq $MgCl_2$ | 69 | <1 |

Reaction conditions: **1a** (0.2 mmol), **2a** (0.4 mmol), $NiBr_2$ (10 mol %), dpph (5 mol%), dtbpy (5 mol%), Mn (3.0 equiv), additive (1.0 equiv) and DMF / Dioxane = 0.3 : 0.7 mL, at RT for 12 h under $N_2$. GC yields.

## 3.5 Table S5. Optimization of NaI additive

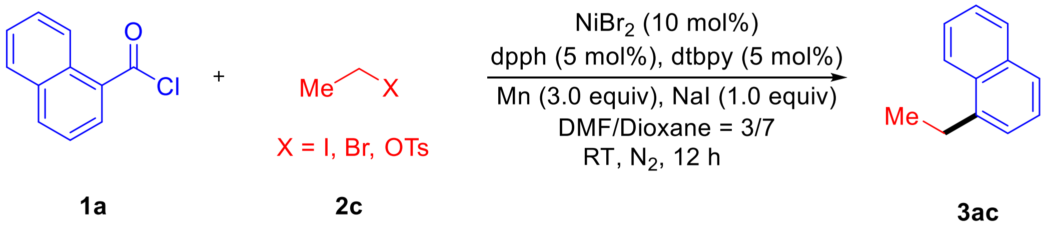


| entry | conditions | yield of 3ac /% |
|---|---|---|
| 1 | X = I | 61 |
| 2 | X = I, without NaI | 54 |
| 3 | X = Br | 70 |
| 4 | X = Br, without NaI | 49 |
| 5 | X = OTs | 72 |
| 6 | X = OTs, without NaI | 33 |

Reaction conditions: **1a** (0.2 mmol), **2** (0.4 mmol), $NiBr_2$ (10 mol %), dpph (5 mol%), dtbpy (5 mol%), Mn (3.0 equiv), NaI (1.0 equiv) and DMF / Dioxane = 0.3 : 0.7 mL, at RT for 12 h under $N_2$. GC yields.

## 3.6 Table S6. Optimization of solvent

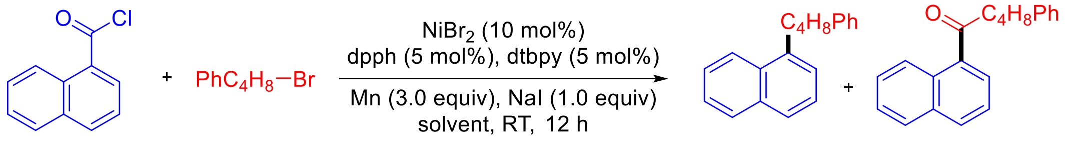


| entry | solvent | yield of 3aa /% | yield of 4aa /% |
|---|---|---|---|
| 1 | DMA | 20 | 8 |
| 2 | DMF | 20 | 1 |
| 3 | DMSO | 12 | 2 |
| 4 | THF | N.D. | N.D. |
| 5 | toluene | N.D. | N.D. |
| 6 | MeCN | N.D. | N.D. |
| 7 | Dioxane | N.D. | N.D. |
| 8 | DMF / THF = 0.3 : 0.7 | 8 | <1 |
| 9 | DMF / Toluene = 0.3 : 0.7 | 26 | <1 |
| 10 | DMF / MeCN = 0.3 : 0.7 | 55 | <1 |
| 11 | DMF / Dioxane = 0.3 : 0.7 | 78 | <1 |
| 12 | DMF / MeCN = 0.5 : 0.5 | 70 | <1 |
| 13 | DMF / MeCN = 0.7 : 0.3 | 61 | <1 |

Reaction conditions: **1a** (0.2 mmol), **2a** (0.4 mmol), $NiBr_2$ (10 mol %), dpph (5 mol%), dtbpy (5 mol%), Mn (3.0 equiv), NaI (1.0 equiv) and solvent (1 mL), at RT for 12 h under $N_2$. GC yields.

## 3.7 Table S7. Optimization of ratio and temperature

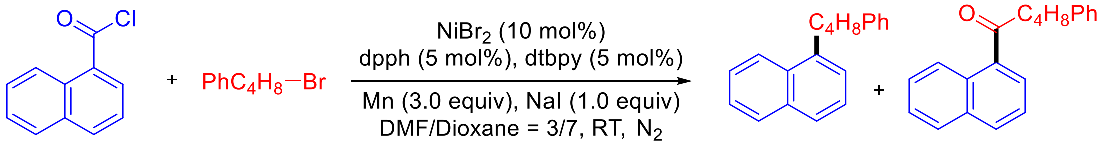


| entry | Variation of optimal conditions | yield of 3aa /% | yield of 4aa /% |
|---|---|---|---|
| 1 | No | 78 | <1 |
| 2 | **1a** : **2a** = 0.2 : 0.2 | 47 | 10 |
| 3 | **1a** : **2a** = 0.2 : 0.6 | 79 | <1 |
| 4 | **1a** : **2a** = 0.4 : 0.2 | 59 | 26 |
| 5 | 0 $^{o}$C, 12 h or 24 h | 10, 18 | 8, 39 |
| 6 | 10 $^{o}$C, 24 h | 66 | 3 |
| 7 | RT, 48 h | 79 | <1 |
| 8 | 50 $^{o}$C | 29 | <1 |
| 9 | 100 $^{o}$C | 19 | <1 |

Reaction conditions: **1a** (0.2 mmol), **2a** (0.4 mmol), $NiBr_2$ (10 mol %), dpph (5 mol%), dtbpy (5 mol%), Mn (3.0 equiv), NaI (1.0 equiv) and DMF / Dioxane = 0.3 : 0.7 mL, at RT for 12 h under $N_2$. GC yields.

## 4. Characterization data for products

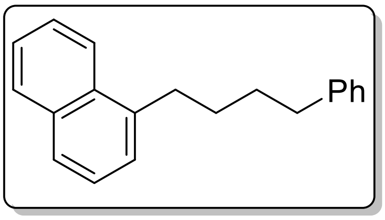

**1-(4-Phenylbutyl) naphthalene** (**3aa**).[1] The representative procedure was followed using 1-naphthoyl chloride (**1a**) (38.00 mg, 0.20 mmol) and (4-bromobutyl) benzene (**2a**) (84.8 mg, 0.40 mmol). Isolation by column chromatography (*n*-hexane) yielded **3aa** (40.56 mg, 78%) as a colorless oil; **$^1$H NMR (500 MHz, $CDCl_3$)** $\delta$ 7.90 (d, $J$ = 8.5 Hz, 1H), 7.72 (d, $J$ = 7.5 Hz, 1H), 7.58 (d, $J$ = 8.0 Hz, 1H), 7.39-7.33 (m, 2H), 7.26 (t, $J$ = 7.5 Hz, 1H), 7.19-7.15 (m, 3H), 7.08-7.06 (m, 3H), 2.98 (t, $J$ = 7.5 Hz, 2H), 2.55 (t, $J$ = 7.5 Hz, 2H), 1.70-1.62 (m, 4H); **$^{13}$C NMR (125 MHz, $CDCl_3$)** $\delta$ 142.5, 138.6, 133.8, 131.8, 128.7, 128.4, 128.2, 126.4, 125.9, 125.7, 125.6, 125.5, 125.3, 123.8, 35.8, 32.9, 31.5, 30.3; **MS** (EI) *m/z* (relative intensity): 260 ($M^+$, 50), 183 (70), 127 (100), 169 (40), 119(60).

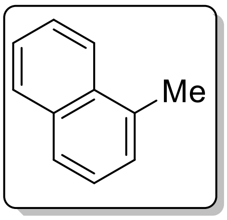

**1-Methylnaphthalene** (**3ab**).[1] The representative procedure was followed using 1-naphthoyl chloride (**1a**) (38.00 mg, 0.20 mmol) and methyl 4-methylbenzenesulfonate (**2b**) (196.8 mg, 0.60 mmol) or iodomethane (**2b'**) (84.6 mg, 0.60 mmol). Isolation by column chromatography (*n*-hexane) yielded **3ab** (18.18 mg, 67%; 24.99 mg, 88%) as a colorless oil; **$^1$H NMR (500 MHz, $CDCl_3$)** $\delta$ 8.14 (dd, $J$ = 7.5 Hz, 11.5 Hz, 1H), 8.00 (dd, $J$ = 8.0 Hz, 12.0 Hz, 1H), 7.87 (dd, $J$ = 8.5 Hz, 12.5 Hz, 1H), 7.72-7.61 (m, 2H), 7.60-7.52 (m, 1H), 7.50-7.46 (m, 1H), 2.86 (s, 3H); **$^{13}$C NMR (125 MHz, $CDCl_3$)** $\delta$ 134.2, 133.5, 132.6, 128.5, 126.5, 126.3, 125.7, 125.5, 124.0, 19.3; **MS** (EI) *m/z* (relative intensity): 142 ($M^+$, 80), 127 (100).

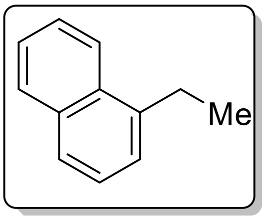


**1-Ethylnaphthalene** (**3ac**).[1] The representative procedure was followed using 1-naphthoyl chloride (**1a**) (38.00 mg, 0.20 mmol) and bromoethane (**2c**) (43.2 mg, 0.40 mmol) or iodoethane (**2c'**) (93.0 mg, 0.60 mmol) or methyl ethyl 4-methylbenzenesulfonate (**2c''**) (120.0 mg, 0.60 mmol). Isolation by column chromatography (*n*-hexane) yielded **3ac** (19.0 mg, 61%; 21.84 mg, 70%; 22.46 mg, 72%) as a colorless oil; **$^1$H NMR (500 MHz, CDCl$_3$)** $\delta$ 8.14 (d, $J$ = 8.0 Hz, 1H), 7.93 (d, $J$ = 8.0 Hz, 1H), 7.79 (d, $J$ = 8.0 Hz, 1H), 7.61-7.54 (m, 2H), 7.51-7.48 (m, 1H), 7.45-7.42 (m, 1H), 3.20 (q, $J$ = 7.5 Hz, 2H), 1.48 (t, $J$ = 7.5 Hz, 3H); **$^{13}$C NMR (125 MHz, CDCl$_3$)** $\delta$ 140.2, 133.8, 131.7, 128.7, 126.4, 125.6, 125.3, 124.8, 123.7, 25.9, 15.0; **MS** (EI) *m/z* (relative intensity): 156 ($M^+$, 80), 141 (100).

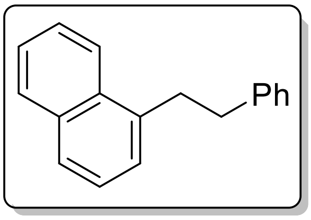


**1-Phenethylnaphthalene** (**3ad**).[2] The representative procedure was followed using 1-naphthoyl chloride (**1a**) (38.00 mg, 0.20 mmol) and (2-bromoethyl) benzene (**2d**) (74.0 mg, 0.40 mmol) or phenethyl 4-methylbenzenesulfonate (**2d'**) (165.6 mg, 0.60 mmol). Isolation by column chromatography (*n*-hexane) yielded **3ad** (33.4 mg, 72%; 32.5 mg, 70%) as a colorless oil; **$^1$H NMR (500 MHz, CDCl$_3$)** $\delta$ 7.98 (dd, $J$ = 2.0 Hz, 8.0 Hz, 1H), 7.74 (dd, $J$ = 2.0 Hz, 8.0 Hz, 1H), 7.60 (dd, $J$ = 2.0 Hz, 8.0 Hz, 1H), 7.42-7.36 (m, 2H), 7.28-7.24 (m, 1H), 7.24-7.21 (m, 3H), 7.14-7.12 (m, 2H), 7.06-7.04 (m, 1H), 3.27-3.24 (m, 2H), 2.96-2.93 (m, 2H); **$^{13}$C NMR (125 MHz, CDCl$_3$)** $\delta$ 141.9, 137.7, 133.8, 131.7, 128.8, 128.4, 128.2, 126.7, 125.98, 125.96, 125.8, 125.5, 125.4, 123.6, 37.0, 35.1; **MS** (EI) *m/z* (relative intensity): 232 ($M^+$, 80), 141 (100),

127 (100), 105 (60), 91 (40).

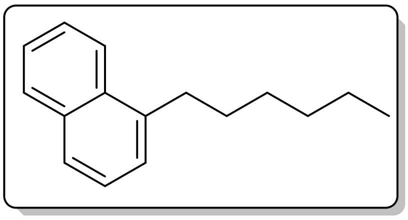

**1-Hexylnaphthalene** (**3ae**).[2] The representative procedure was followed using 1-naphthoyl chloride (**1a**) (38.00 mg, 0.20 mmol) and 1-iodohexane (**2e'**) (127.2 mg, 0.60 mmol) or 1-bromohexane (**2e**) (66.1 mg, 0.40 mmol) or hexyl 4-methylbenzenesulfonate (**2e''**) (153.6 mg, 0.60 mmol). Isolation by column chromatography (*n*-hexane) yielded **3ae** (27.98 mg, 66%; 33.5 mg, 79%; 34.8 mg, 82%) as a colorless oil; **$^1$H NMR (500 MHz, $CDCl_3$)** $\delta$ 8.08 (d, *J* = 8.5 Hz, 1H), 7.88 (d, *J* = 8.0 Hz, 1H), 7.74 (d, *J* = 8.0 Hz, 1H), 7.56-7.49 (m, 2H), 7.43 (t, *J* = 8.0 Hz, 1H), 7.35 (d, *J* = 7.0 Hz, 1H), 3.10 (d, *J* = 7.5 Hz, 1H), 1.82-1.76 (m, 2H), 1.51-1.45 (m, 2H), 1.40-1.34 (m, 4H), 0.94 (t, *J* = 7.0 Hz, 3H); **$^{13}$C NMR (125 MHz, $CDCl_3$)** $\delta$ 139.0, 133.8, 131.9, 128.7, 126.3, 125.8, 125.6, 125.5, 125.3, 123.9, 33.1, 31.7, 30.8, 29.5, 22.7, 14.1; **MS** (EI) *m/z* (relative intensity): 212 ($M^+$, 80), 197 (100), 169 (100), 141 (40).

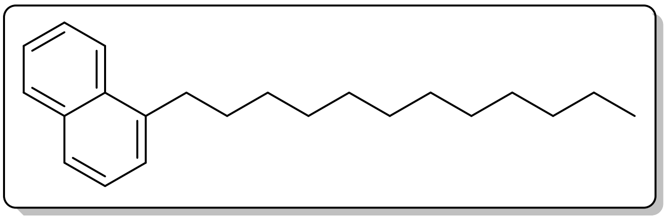

**1-Dodecylnaphthalene** (**3af**).[3] The representative procedure was followed using 1-naphthoyl chloride (**1a**) (38.00 mg, 0.20 mmol) and 1-bromododecane (**2f**) (99.6 mg, 0.40 mmol) or 1-iodododecane (**2f'**) (177.6 mg, 0.60 mmol) or dodecyl 4-methylbenzenesulfonate (**2f''**) (204.2 mg, 0.60 mmol). Isolation by column chromatography (*n*-hexane) yielded **3af** (27.98 mg, 66%; 33.5 mg, 79%; 34.8 mg, 82%) as a colorless oil; **$^1$H NMR (500 MHz, $CDCl_3$)** $\delta$ 7.73-7.68 (m, 1H), 7.53 (s, 1H), 7.38-7.31 (m, 2H), 7.25 (d, *J* = 8.5 Hz, 1H), 2.69 (t, *J* = 7.5 Hz, 1H), 1.65-1.59

(m, 2H), 1.34-1.26 (m, 6H), 1.22-1.16 (m, 12H), 0.80 (t, *J* = 6.5 Hz, 3H); **$^{13}$C NMR (125 MHz, $CDCl_3$)** *δ* 140.5, 133.6, 131.9, 137.7, 127.6, 127.44, 127.37, 126.2, 125.8, 124.9, 36.1, 31.9, 31.4, 29.7, 29.63, 29.59, 29.5, 29.4, 29.3, 29.2, 22.7, 14.1; **MS** (EI) *m/z* (relative intensity): 296 ($M^+$, 80), 211 (100), 141 (40).

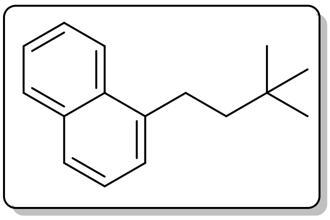

**1-(3,3-Dimethylbutyl) naphthalene** (**3ag**). The representative procedure was followed using 1-naphthoyl chloride (**1a**) (38.00 mg, 0.20 mmol) and 1-bromo-3,3-dimethylbutane (**2g**) (66.0 mg, 0.40 mmol) or 3,3-dimethylbutyl 4-methylbenzenesulfonate (**2g'**) (153.6 mg, 0.60 mmol). Isolation by column chromatography (*n*-hexane) yielded **3ag** (27.98 mg, 66%; 30.1 mg, 71%) as a colorless oil; **$^1$H NMR (500 MHz, $CDCl_3$)** *δ* 8.00 (d, *J* = 8.5 Hz, 1H), 7.81 (d, *J* = 8.0 Hz, 1H), 7.66 (d, *J* = 8.0 Hz, 1H), 7.49-7.46 (m, 1H), 7.44-7.41 (m, 1H), 7.37-7.34 (m, 1H), 7.29 (d, *J* = 7.0 Hz, 1H), 3.03-3.00 (m, 2H), 1.62-1.59 (m, 2H), 1.03 (s, 9H); **$^{13}$C NMR (125 MHz, $CDCl_3$)** *δ* 139.6, 133.9, 131.8, 128.8, 126.3, 125.7, 125.6, 125.3, 123.7, 45.6, 30.8, 29.3, 28.3; **HR-MS** (ESI+): *m/z* calcd. for [Chemical Formula: $C_{16}H_{20}$ + H]$^+$ 213.1638, found 213.1637.

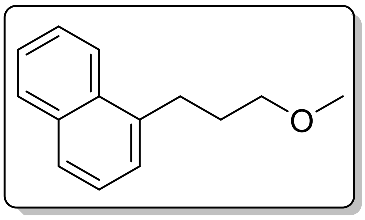


**1-(3-Methoxypropyl) naphthalene** (**3ah**). The representative procedure was followed using 1-naphthoyl chloride (**1a**) (38.00 mg, 0.20 mmol) and 1-bromo-3-methoxypropane (**2h**) (61.2 mg, 0.40 mmol) or 3-methoxypropyl 4-methylbenzenesulfonate (**2h'**) (146.4 mg, 0.60 mmol). Isolation by column chromatography (*n*-hexane : EtOAc = 100 : 1) yielded **3ah** (31.76 mg, 79%; 31.36

mg, 78%) as a colorless oil; **$^1$H NMR (500 MHz, $CDCl_3$)** $\delta$ 8.08 (d, $J$ = 8.5 Hz, 1H), 7.86 (d, $J$ = 8.0 Hz, 1H), 7.73 (d, $J$ = 8.0 Hz, 1H), 7.54-7.51 (m, 1H), 7.50-7.47 (m, 1H), 7.42 (t, $J$ = 8.0 Hz, 1H), 7.36 (d, $J$ = 6.5 Hz, 1H), 3.47 (t, $J$ = 6.5 Hz, 2H), 3.40 (s, 3H), 3.18 (t, $J$ = 8.0 Hz, 2H), 2.07-2.02 (m, 2H); **$^{13}$C NMR (125 MHz, $CDCl_3$)** $\delta$ 138.1, 133.8, 131.9, 128.7, 126.6, 126.0, 125.7, 125.,5 125.4, 123.8, 72.9, 58.6, 30.6, 29.4; **HR-MS** (ESI+): $m/z$ calcd. for [Chemical Formula: $C_{14}H_{16}O$ + H]$^+$ 201.1274, found 201.1274.

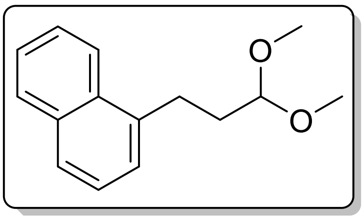

**1-(3,3-Dimethoxypropyl) naphthalene** (**3ai**). The representative procedure was followed using 1-naphthoyl chloride (**1a**) (38.00 mg, 0.20 mmol) and 3-bromo-1,1-dimethoxypropane (**2i**) (73.22 mg, 0.40 mmol) or 3,3-dimethoxypropyl 4-methylbenzenesulfonate (**2i'**) (164.4 mg, 0.60 mmol). Isolation by column chromatography ($n$-hexane : EtOAc = 30 : 1) yielded **3ai** (39.56 mg, 86%; 40.48 mg, 88%) as a white solid ; **$^1$H NMR (500 MHz, $CDCl_3$)** $\delta$ 8.05 (d, $J$ = 8.5 Hz, 1H), 7.83 (d, $J$ = 8.0 Hz, 1H), 7.70 (d, $J$ = 8.0 Hz, 1H), 7.52-7.49 (m, 1H), 7.47-7.45 (m, 1H), 7.40-7.37 (m, 1H), 7.33 (d, $J$ = 7.0 Hz, 1H), 4.45 (t, $J$ = 5.5 Hz, 1H), 3.35 (s, 6H), 3.13 (t, $J$ = 8.0 Hz, 2H), 2.07-2.03 (m, 2H); **$^{13}$C NMR (125 MHz, $CDCl_3$)** $\delta$ 1337.7, 133.8, 131.8, 128.7, 126.7, 125.9, 125.8, 125.5, 125.4, 123.7, 104.0, 52.8, 33.4, 27.9; **HR-MS** (ESI+): $m/z$ calcd. for [Chemical Formula: $C_{15}H_{18}O_2$ + H]$^+$ 231.1380, found 231.1379.

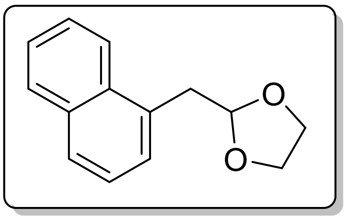

**2-(Naphthalen-1-ylmethyl)-1,3-dioxolane** (**3aj**).[4] The representative procedure was

followed using 1-naphthoyl chloride (**1a**) (38.00 mg, 0.20 mmol) and 2-(bromomethyl)-1,3-dioxolane (**2j**) (66.80 mg, 0.40 mmol) or (1,3-dioxolan-2-yl)methyl 4-methylbenzenesulfonate (**2j'**) (154.80 mg, 0.60 mmol). Isolation by column chromatography (*n*-hexane : EtOAc = 30 : 1) yielded **3aj** (36.38 mg, 85%; 38.09 mg, 89%) as a white solid ; **$^1$H NMR (500 MHz, CDCl$_3$)** $\delta$ 8.00 (d, $J$ = 8.0 Hz, 1H), 7.74 (d, $J$ = 7.5 Hz, 1H), 7.65 (d, $J$ = 7.5 Hz, 1H), 7.43-7.31 (m, 4H), 5.12 (t, $J$ = 5.0 Hz, 1H), 3.87-3.82 (m, 2H), 3.74-3.69 (m, 2H), 3.34 (d, $J$ = 5.0 Hz, 2H); **$^{13}$C NMR (125 MHz, CDCl$_3$)** $\delta$ 133.7, 132.4, 132.2, 128.6, 127.8, 127.4, 125.8, 125.4, 123.9, 104.2, 64.9, 37.7; **MS** (EI) *m/z* (relative intensity): 214 ($M^+$, 80), 141 (100).

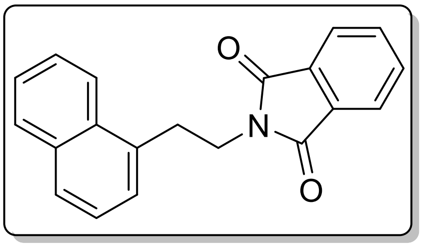

**2-[2-(Naphthalen-1-yl) ethyl] isoindoline-1,3-dione** (**3ak**).[5] The representative procedure was followed using 1-naphthoyl chloride (**1a**) (38.00 mg, 0.20 mmol) and 2-(2-bromoethyl) isoindoline-1,3-dione (**2k**) (101.60 mg, 0.40 mmol) or 2-(1,3-dioxoisoindolin-2-yl) ethyl 4-methylbenzenesulfonate (**2k'**) (207.00 mg, 0.60 mmol). Isolation by column chromatography (*n*-hexane : EtOAc = 30 : 1) yielded **3ak** (46.96 mg, 78%; 50.57 mg, 84%) as a white solid ; **$^1$H NMR (500 MHz, CDCl$_3$)** $\delta$ 8.22 (d, $J$ = 3.5 Hz, 1H), 7.78-7.76 (m, 3H), 7.67 (d, $J$ = 7.5 Hz, 1H), 7.64-7.62 (m, 2H), 7.52-7.49 (m, 1H), 7.41 (t, $J$ = 7.5 Hz, 1H), 7.34-7.29 (m, 2H), 3.95 (t, $J$ = 8.0 Hz, 2H), 3.34 (t, $J$ = 7.5 Hz, 2H); **$^{13}$C NMR (125 MHz, CDCl$_3$)** $\delta$ 168.2, 134.1, 133.9, 133.8, 132.1, 131.9, 128.7, 127.5, 127.0, 126.4, 125.7, 125.5, 123.7, 123.2, 38.7, 32.2; **MS** (EI) *m/z* (relative intensity): 301 ($M^+$, 60), 174 (100), 160 (50), 141 (80) .

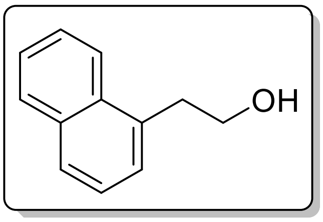

**2-(Naphthalen-1-yl) ethan-1-ol** (**3al**).[6] The representative procedure was followed using 1-naphthoyl chloride (**1a**) (38.00 mg, 0.20 mmol) and 2-bromoethan-1-ol (**2l**) (49.61 mg, 0.40 mmol). Isolation by column chromatography (*n*-hexane : EtOAc = 20 : 1) yielded **3al** (36.38 mg, 66%) as a colorless oil ; **$^1$H NMR (500 MHz, $CDCl_3$)** $\delta$ 8.00 (d, *J* = 8.5 Hz, 1H), 7.81 (d, *J* = 8.0 Hz, 1H), 7.70 (d, *J* = 8.0 Hz, 1H), 7.50-7.43 (m, 2H), 7.37 (t, *J* = 7.5 Hz, 1H), 7.31 (d, *J* = 6.5 Hz, 1H), 3.90 (t, *J* = 6.5 Hz, 2H), 3.28 (t, *J* = 6.5 Hz, 2H); **$^{13}$C NMR (125 MHz, $CDCl_3$)** $\delta$ 134.3, 133.8, 131.9, 128.7, 127.2, 127.0, 125.9, 125.4, 123.5, 62.8, 36.0; **MS** (EI) *m/z* (relative intensity): 172 ($M^+$, 60), 141 (100) .

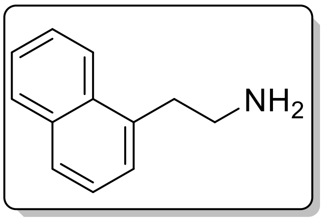

**2-(Naphthalen-1-yl) ethan-1-amine** (**3am**).[7] The representative procedure was followed using 1-naphthoyl chloride (**1a**) (38.00 mg, 0.20 mmol) and 2-bromoethan-1-amine (**2m**) (49.20 mg, 0.40 mmol). Isolation by column chromatography (*n*-hexane : EtOAc = 20 : 1) yielded **3am** (23.94 mg, 70%) as a colorless oil ; **$^1$H NMR (500 MHz, $CDCl_3$)** $\delta$ 8.05 (d, *J* = 8.0 Hz, 1H), 7.86 (d, *J* = 8.0 Hz, 1H), 7.74 (d, *J* = 8.0 Hz, 1H), 7.54-7.47 (m, 2H), 7.42 (t, *J* = 8.0 Hz, 1H), 7.34 (d, *J* = 7.0 Hz, 1H), 3.23 (t, *J* = 7.0 Hz, 2H), 3.10 (t, *J* = 7.0 Hz, 2H), 1.58 (brs, 2H); **$^{13}$C NMR (125 MHz, $CDCl_3$)** $\delta$ 135.6, 133.9, 131.9, 128.7, 126.9, 126.7, 125.8, 125.5, 125.4, 123.6, 42.7, 37.1; **MS** (EI) *m/z* (relative intensity): 171 ($M^+$, 60), 141 (100) .

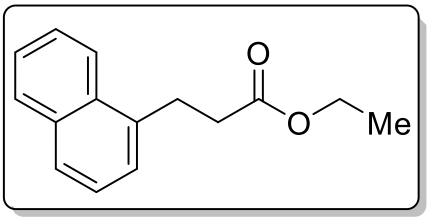


**Ethyl 3-(naphthalen-1-yl) propanoate** (**3an**).[8] The representative procedure was followed using 1-naphthoyl chloride (**1a**) (38.00 mg, 0.20 mmol) and ethyl 3-bromopropanoate (**2n**) (72.40 mg, 0.40 mmol) or ethyl 3-(tosyloxy) propanoate (**2n"**) (163.20 mg, 0.60 mmol). Isolation by column chromatography (n-hexane : EtOAc = 50 : 1) yielded **3an** (23.71 mg, 52%; 26.90 mg, 59%) as a colorless oil ; **$^{1}$H NMR (500 MHz, $CDCl_3$)** $\delta$ 8.04 (d, $J$ = 8.5 Hz, 1H), 7.86 (d, $J$ = 7.5 Hz, 1H), 7.74 (d, $J$ = 8.0 Hz, 1H), 7.55-7.48 (m, 2H), 7.42-7.35 (m, 2H), 4.19-4.14 (m, 2H), 3.43 (t, $J$ = 8.0 Hz, 2H), 2.76 (t, $J$ = 7.5 Hz, 2H), 1.26 (t, $J$ = 7.0 Hz, 3H); **$^{13}$C NMR (125 MHz, $CDCl_3$)** $\delta$ 173.0, 136.5, 133.8, 131.6, 128.8, 127.1, 126.0, 125.9, 125.6, 125.5, 123.4, 60.5, 35.2, 28.1, 14.2; **MS** (EI) *m/z* (relative intensity): 228 ($M^+$, 60), 213 (50), 199 (100), 155 (70), 141 (60) .

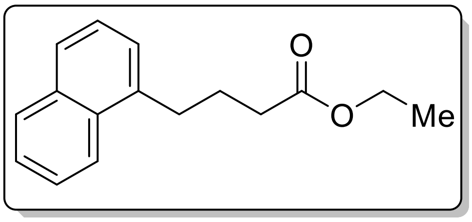


**Ethyl 4-(naphthalen-1-yl) butanoate** (**3ao**).[8] The representative procedure was followed using 1-naphthoyl chloride (**1a**) (38.00 mg, 0.20 mmol) and ethyl 4-bromobutanoate (**2o**) (78.00 mg, 0.40 mmol) or ethyl 4-(tosyloxy) butanoate (**2o'**) (171.60 mg, 0.60 mmol). Isolation by column chromatography (*n*-hexane : EtOAc = 50 : 1) yielded **3ao** (25.56 mg, 59%; 29.04 mg, 60%) as a colorless oil ; **$^{1}$H NMR (500 MHz, $CDCl_3$)** $\delta$ 8.07 (d, $J$ = 8.5 Hz, 1H), 7.86 (d, $J$ = 7.5 Hz, 1H), 7.72 (d, $J$ = 8.5 Hz, 1H), 7.54-7.46 (m, 2H), 7.40 (t, $J$ = 7.5 Hz, 1H), 7.32 (d, $J$ = 7.0 Hz, 1H), 4.15 (q, $J$ = 8.5 Hz, 2H), 3.13 (t, $J$ = 7.5 Hz, 2H), 2.42 (t, $J$ = 7.5 Hz, 2H), 2.13-2.07 (m, 2H), 1.27 (t, $J$ = 7.0 Hz, 3H); **$^{13}$C NMR (125 MHz, $CDCl_3$)** $\delta$ 173.5, 137.6, 133.9, 131.8, 128.7, 126.8, 126.2, 125.8, 125.5, 123.8, 60.3, 34.0, 32.3, 25.8, 14.2; **MS** (EI)

*m/z* (relative intensity): 242 ($M^+$, 60), 227 (50), 213 (100), 169 (70), 141 (60) .

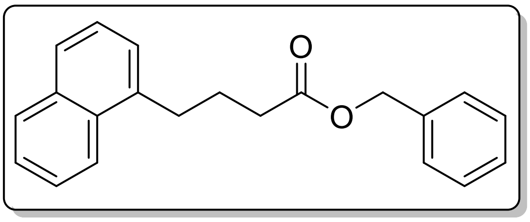


**Benzyl 4-(naphthalen-1-yl) butanoate** (**3ap**).[8] The representative procedure was followed using 1-naphthoyl chloride (**1a**) (38.00 mg, 0.20 mmol) and benzyl 4-(tosyloxy)butanoate (**2p**) (78.00 mg, 0.40 mmol) or ethyl 4-(tosyloxy) butanoate (**2p'**) (208.80 mg, 0.60 mmol). Isolation by column chromatography (*n*-hexane : EtOAc = 50 : 1) yielded **3ap** (41.96 mg, 69%; 43.78 mg, 72%) as a colorless oil ; **$^1$H NMR (500 MHz, $CDCl_3$)** $\delta$ 8.06 (d, *J* = 7.5 Hz, 1H), 7.87 (d, *J* = 9.0 Hz, 1H), 7.74 (d, *J* = 8.0 Hz, 1H), 7.54-7.48 (m, 2H), 7.42-7.39 (m, 4H), 7.38-7.34 (m, 2H), 7.31 (d, *J* = 7.0 Hz, 1H), 5.16 (s, 2H), 3.14 (t, *J* = 7.5 Hz, 2H), 2.50 (t, *J* = 7.0 Hz, 2H), 2.17-2.11 (m, 2H); **$^{13}$C NMR (125 MHz, $CDCl_3$)** $\delta$ 173.2, 137.4, 135.9, 133.8, 131.7, 128.7, 128.5, 128.3, 128.2, 126.8, 126.2, 125.8, 125.5, 125.4, 123.7, 66.2, 33.9, 32.3, 25.8; **MS** (EI) *m/z* (relative intensity): 304 ($M^+$, 60), 213 (100), 169 (80), 163 (70), 91 (60) .

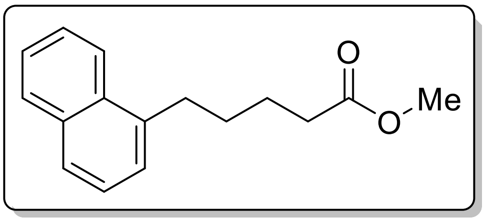


**Methyl 5-(naphthalen-1-yl) pentanoate** (**3aq**).[8] The representative procedure was followed using 1-naphthoyl chloride (**1a**) (38.00 mg, 0.20 mmol) and methyl 5-bromopentanoate (**2q**) (78.00 mg, 0.40 mmol) or methyl 5-(tosyloxy) pentanoate (**2q'**) (171.60 mg, 0.60 mmol). Isolation by column chromatography (*n*-hexane : EtOAc = 50 : 1) yielded **3aq** (29.52 mg, 61%; 32.91 mg, 68%) as a colorless oil ; **$^1$H NMR (500 MHz, $CDCl_3$)** $\delta$ 8.00 (d, *J* = 8.0 Hz, 1H), 7.82 (d, *J* = 8.0 Hz, 1H), 7.68 (d, *J* = 8.5 Hz, 1H), 7.39-7.43 (m, 2H), 7.37 (t, *J* = 8.0 Hz, 1H), 7.28 (d, *J* = 7.0 Hz, 1H), 4.09 (t, *J* = 6.5 Hz, 2H), 3.07 (t, *J* = 8.0 Hz, 2H), 2.01 (s, 3H), 1.81-1.78 (m, 2H),

1.75-1.71 (m, 2H); **$^{13}$C NMR (125 MHz, $CDCl_3$)** $\delta$ 171.0, 138.0, 133.8, 131.7, 128.7, 126.6, 125.7, 125.4, 125.3, 123.6, 64.2, 32.4, 28.5, 26.9, 20.9; **MS** (EI) *m/z* (relative intensity): 242 ($M^+$, 60), 227 (100), 183 (80), 141 (60) .

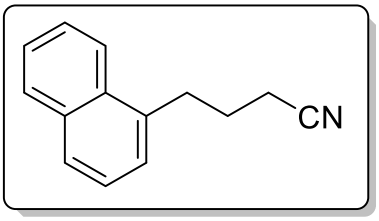


**4-(Naphthalen-1-yl) butanenitrile** (**3ar**). The representative procedure was followed using 1-naphthoyl chloride (**1a**) (38.00 mg, 0.20 mmol) and 4-bromobutanenitrile (**2r**) (58.40 mg, 0.40 mmol). Isolation by column chromatography (*n*-hexane : EtOAc = 20 : 1) yielded **3ar** (20.28 mg, 52%) as a colorless oil ; **$^1$H NMR (500 MHz, $CDCl_3$)** $\delta$ 7.91 (d, *J* = 8.0 Hz, 1H), 7.82 (d, *J* = 8.0 Hz, 1H), 7.67 (d, *J* = 8.0 Hz, 1H), 7.48-7.44 (m, 1H), 7.44-7.40 (m, 1H), 7.33 (t, *J* = 7.5 Hz, 1H), 7.25 (d, *J* = 6.5 Hz, 1H), 3.16 (t, *J* = 7.5 Hz, 2H), 2.28 (t, *J* = 7.0 Hz, 2H), 2.06-2.00 (m, 2H); **$^{13}$C NMR (125 MHz, $CDCl_3$)** $\delta$ 135.6, 133.9, 131.5, 128.9, 127.4, 126.5, 126.2, 125.7, 125.5, 123.3, 119.5, 31.5, 26.1, 16.7; **HR-MS** (ESI+): *m/z* calcd. for [Chemical Formula: $C_{14}H_{13}N$ + H]$^+$ 196.1121, found 196.1120.

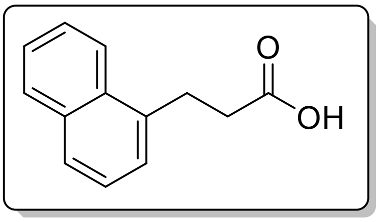


**3-(Naphthalen-1-yl) propanoic acid** (**3as**).[9] The representative procedure was followed using 1-naphthoyl chloride (**1a**) (38.00 mg, 0.20 mmol) and 3-bromopropanoic acid (**2s**) (60.80 mg, 0.40 mmol). Isolation by column chromatography (*n*-hexane : EtOAc = 10 : 1) yielded **3as** (20.40 mg, 51%) as a colorless oil ; **$^1$H NMR (500 MHz, $CDCl_3$)** $\delta$ 8.03 (d, *J* = 8.5 Hz, 1H), 7.87 (d, *J* = 8.0 Hz, 1H), 7.75 (d, *J* = 8.0 Hz, 1H), 7.56-7.53 (m, 1H), 7.51-7.49 (m, 1H), 7.43-7.37 (m, 2H), 3.45 (t, *J* = 7.5 Hz, 2H), 2.84 (t, *J* = 7.5 Hz, 2H); **$^{13}$C NMR (125 MHz,**

**$CDCl_3$)** $\delta$ 174.0, 136.1, 133.9, 131.5, 128.9, 127.3, 126.2, 125.9, 125.7, 125.6, 123.3, 34.7, 27.8; **MS** (EI) *m/z* (relative intensity): 200 ($M^+$, 60), 141 (100) .

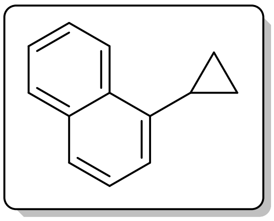

**1-Cyclopropylnaphthalene** (**3at**).[10] The representative procedure was followed using 1-naphthoyl chloride (**1a**) (38.00 mg, 0.20 mmol) and iodocyclopropane (**2t'**) (100.20 mg, 0.60 mmol) or bromocyclopropane (**2t**) (48.00 mg, 0.40 mmol) or cyclopropyl 4-methylbenzenesulfonate (**2t''**) (127.20 mg, 0.60 mmol). Isolation by column chromatography (n-hexane) yielded **3at** (16.80 mg, 50%; 18.48 mg, 55%; 19.49 mg, 58%) as a colorless oil ; **$^1$H NMR (500 MHz, $CDCl_3$)** $\delta$ 8.49 (d, *J* = 3.5 Hz, 1H), 7.92 (d, *J* = 8.0 Hz, 1H), 7.78 (d, *J* = 8.0 Hz, 1H), 7.63 (t, *J* = 8.0 Hz, 1H), 7.57 (t, *J* = 7.5 Hz, 1H), 7.46 (t, *J* = 7.5 Hz, 1H), 7.35 (d, *J* = 7.0 Hz, 1H), 2.45-2.39 (m, 1H), 1.16-1.12 (m, 2H), 0.87-0.84 (m, 2H); **$^{13}$C NMR (125 MHz, $CDCl_3$)** $\delta$ 139.1, 133.49, 133.47, 128.4, 126.5, 125.7, 125.5, 125.4, 124.4, 123.7, 13.2, 6.4; **MS** (EI) *m/z* (relative intensity): 168 ($M^+$, 70), 127 (100) .

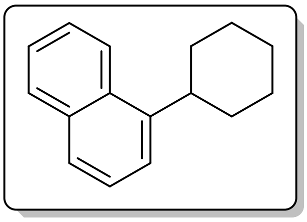

**1-Cyclohexylnaphthalene** (**3au**).[11] The representative procedure was followed using 1-naphthoyl chloride (**1a**) (38.00 mg, 0.20 mmol) and iodocyclohexane (**2u'**) (126.0 mg, 0.60 mmol) or bromocyclohexane (**2u**) (65.20 mg, 0.40 mmol) or cyclohexyl 4-methylbenzenesulfonate (**2u''**) (152.40 mg, 0.60 mmol). Isolation by column chromatography (*n*-hexane) yielded **3au** (25.20 mg, 60%; 26.00 mg, 62%; 28.56 mg, 68%) as a colorless oil ; **$^1$H NMR (500 MHz, $CDCl_3$)** $\delta$ 8.13 (d, *J* = 8.5 Hz, 1H), 7.87 (d, *J* = 8.0 Hz, 1H), 7.70 (d, *J* = 8.0 Hz, 1H), 7.54-7.51 (m, 1H), 7.49-7.44 (m, 2H),

7.41 (d, $J$ = 8.0 Hz, 1H), 3.36-3.33 (m, 1H), 2.05 (d, $J$ = 7.5 Hz, 2H), 1.93 (d, $J$ = 5.0 Hz, 2H), 1.62-1.53 (m, 4H), 1.50-1.27 (m, 2H); **$^{13}$C NMR (125 MHz, $CDCl_3$)** $\delta$ 143.8, 133.9, 131.3, 128.9, 126.2, 125.64, 125.56, 125.2, 123.2, 122.2, 39.2, 34.2, 27.3, 26.5; **MS** (EI) *m/z* (relative intensity): 210 ($M^+$, 70), 127 (100) .

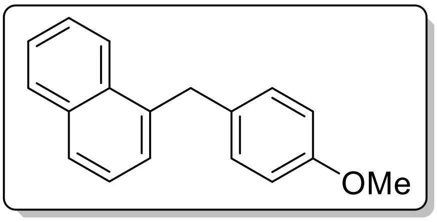


**1-(4-Methoxybenzyl) naphthalene** (**3bv**).[12] The representative procedure was followed using 1-naphthoyl chloride (**1b**) (38.00 mg, 0.20 mmol) and 1-(bromomethyl)-4-methoxybenzene (**2v**) (80.40 mg, 0.40 mmol) or 1-(chloromethyl)-4-methoxybenzene (**2v'**) (93.6 mg, 0.60 mmol). Isolation by column chromatography (*n*-hexane : EtOAc = 100 : 1) yielded **3bv** (38.69 mg, 78%; 35.72 mg, 72%) as a colorless oil ; **$^1$H NMR (500 MHz, $CDCl_3$)** $\delta$ 7.81-7.75 (m, 3H), 7.62 (s, 1H), 7.46-7.42 (m, 2H), 7.31 (d, $J$ = 8.5 Hz, 1H), 7.15 (d, $J$ = 8.5 Hz, 2H), 6.84 (d, $J$ = 8.5 Hz, 2H), 4.09 (s, 2H), 3.79 (s, 3H); **$^{13}$C NMR (125 MHz, $CDCl_3$)** $\delta$ 158.0, 139.0, 133.6, 133.1, 132.0, 129.9, 128.0, 127.59, 127.56, 127.5, 126.9, 125.9, 125.3, 113.9, 55.2, 41.2; **MS** (EI) *m/z* (relative intensity): 248 ($M^+$, 70), 217 (100), 141 (60), 121 (60).

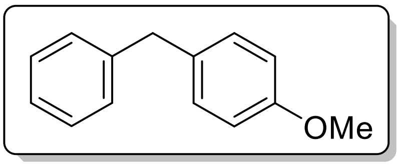


**1-Benzyl-4-methoxybenzene** (**3cv**).[12] The representative procedure was followed using benzoyl chloride (**1c**) (28.00 mg, 0.20 mmol) and 1-(bromomethyl)-4-methoxybenzene (**2v**) (80.40 mg, 0.40 mmol) or 1-(chloromethyl)-4-methoxybenzene (**2v'**) (93.6 mg, 0.60 mmol). Isolation by column chromatography (*n*-hexane : EtOAc = 100 : 1) yielded **3cv** (32.47 mg, 82%; 31.68 mg, 80%) as a colorless oil ; **$^1$H NMR (500 MHz, $CDCl_3$)** $\delta$ 7.20-7.17 (m, 2H), 7.11-7.07 (m, 3H), 7.01 (d, $J$ = 8.5 Hz, 2H),

6.75-6.72 (m, 2H), 3.83 (s, 2H), 3.67 (s, 3H); **$^{13}$C NMR (125 MHz, CDCl$_3$)** $\delta$ 158.0, 141.5, 133.2, 129.8, 128.8, 128.4, 125.9, 113.8, 55.2, 41.0; **MS** (EI) *m/z* (relative intensity): 198 (M$^+$, 70), 167 (100), 121 (60), 91 (60).

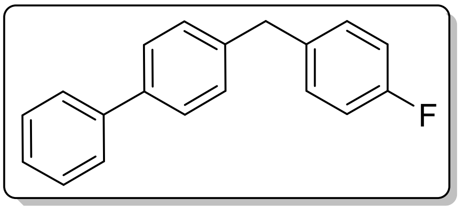


**4-(4-Fluorobenzyl)-1,1'-biphenyl** (**3dw**).[12] The representative procedure was followed using [1,1'-biphenyl]-4-carbonyl chloride (**1d'**) (43.20 mg, 0.20 mmol) and 1-(bromomethyl)-4-fluorobenzene (**2w**) (75.60 mg, 0.40 mmol) or 1-(chloromethyl)-4-fluorobenzene (**2w'**) (86.40 mg, 0.60 mmol). Isolation by column chromatography (*n*-hexane) yielded **3dw** (27.25 mg, 52%; 28.82 mg, 55%) as a colorless oil ; **$^1$H NMR (500 MHz, CDCl$_3$)** $\delta$ 7.57-7.55 (m, 2H), 7.52-7.50 (m, 2H), 7.41 (t, *J* = 7.5 Hz, 2H), 7.33-7.30 (m, 1H), 7.22 (d, *J* = 8.5 Hz, 2H), 7.17-7.15 (m, 2H), 6.97 (t, *J* = 8.5 Hz, 2H), 3.97 (s, 2H); **$^{13}$C NMR (125 MHz, CDCl$_3$)** $\delta$ 161.4 (d, *J* = 242.5 Hz), 140.9, 140.0, 139.2, 136.6 (d, *J* = 3.75 Hz), 130.3 (d, *J* = 7.5 Hz), 129.2, 128.7, 127.2, 127.1, 127.0, 115.2 (d, *J* = 21.25 Hz), 40.7; **$^{19}$F NMR (300 MHz, CDCl3)** $\delta$ -32.67; **MS** (EI) *m/z* (relative intensity): 262 (M$^+$, 70), 185 (60), 167 (100), 153 (60).

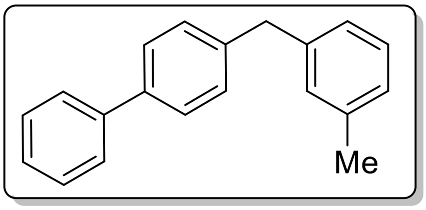


**4-(3-Methylbenzyl)-1,1'-biphenyl (3dx)**.[12] The representative procedure was followed using [1,1'-biphenyl]-4-carbonyl chloride (**1d'**) (43.20 mg, 0.20 mmol) and 1-(bromomethyl)-3-methylbenzene (**2x**) (74.00 mg, 0.40 mmol) or 1-(chloromethyl)-3-methylbenzene (**2x'**) (86.00 mg, 0.60 mmol). Isolation by column chromatography (*n*-hexane) yielded **3dx** (39.22 mg, 76%; 37.15 mg, 72%) as a colorless oil ; **$^1$H NMR (500 MHz, CDCl$_3$)** $\delta$ 7.76 (d, *J* = 7.5 Hz, 2H), 7.72 (d, *J* = 8.0 Hz, 2H), 7.61 (t, *J* =

8.0 Hz, 2H), 7.52 (d, *J* = 7.5 Hz, 1H), 7.45 (d, *J* = 8.0 Hz, 2H), 7.33-7.31 (m, 4H), 4.18 (s, 2H), 2.54 (s, 3H); **$^{13}$C NMR (125 MHz, $CDCl_3$)** $\delta$ 141.0, 140.5, 138.9, 137.9, 135.5, 129.20, 129.16, 129.0, 128.8, 128.6, 128.3, 127.1, 127.0, 126.9, 41.1, 21.0; **MS** (EI) *m/z* (relative intensity): 258 ($M^+$, 70), 243 (60), 167 (100), 105 (60).

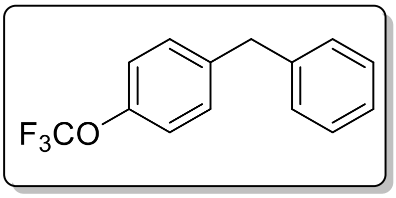


**1-Benzyl-4-(trifluoromethoxy) benzene (3ey)**.[12] The representative procedure was followed using 4-(trifluoromethoxy) benzoyl chloride (**1e**) (44.80 mg, 0.20 mmol) and (bromomethyl) benzene (**2y**) (68.40 mg, 0.40 mmol) or (chloromethyl) benzene (**2y'**) (75.60 mg, 0.60 mmol). Isolation by column chromatography (*n*-hexane : EtOAc = 200 : 1) yielded **3ey** (39.22 mg, 50%; 37.15 mg, 55%) as a colorless oil ; **$^1$H NMR (500 MHz, $CDCl_3$)** $\delta$ 7.26 (d, *J* = 7.5 Hz, 2H), 7.18 (d, *J* = 7.5 Hz, 1H), 7.15-7.12 (m, 4H), 7.07 (d, *J* = 8.0 Hz, 2H), 3.91 (s, 2H); **$^{13}$C NMR (125 MHz, $CDCl_3$)** $\delta$ 147.6 (d, *J* = 2.5 Hz), 140.2 (d, *J* = 66.25 Hz), 130.1, 128.9, 128.6, 126.4, 121.0, 120.7 (q, *J* = 225.0 Hz), 41.2; **$^{19}$F NMR (300 MHz, CDCl3)** $\delta$ -57.80; **MS** (EI) *m/z* (relative intensity): 252 ($M^+$, 70), 175 (60), 167 (100).

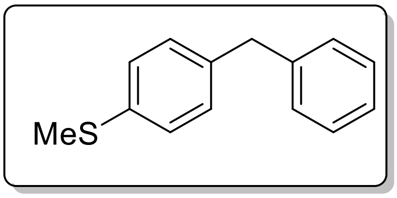


**(4-Benzylphenyl) (methyl) sulfane** (**3fy**).[12] The representative procedure was followed using 4-(methylthio) benzoyl chloride (**1f**) (37.20 mg, 0.20 mmol) and (bromomethyl) benzene (**2y**) (68.40 mg, 0.40 mmol) or (chloromethyl) benzene (**2y'**) (75.60 mg, 0.60 mmol). Isolation by column chromatography (*n*-hexane : EtOAc = 100 : 1) yielded **3fy** (22.26 mg, 52%; 20.54 mg, 48%) as a colorless oil ; **$^1$H NMR (500 MHz, $CDCl_3$)** $\delta$ 7.22-7.17 (m, 2H), 7.13-7.11 (m, 3H), 7.10-7.09 (m, 2H), 7.03 (d, *J* = 8.5 Hz, 2H), 3.86 (s, 2H), 2.38 (s, 3H); **$^{13}$C NMR (125 MHz, $CDCl_3$)** $\delta$ 141.0,

138.2, 135.7, 129.4, 128.8, 128.5, 127.1, 126.1, 41.3, 16.2; **MS** (EI) *m/z* (relative intensity): 214 ($M^+$, 70), 167 (100), 137 (60).

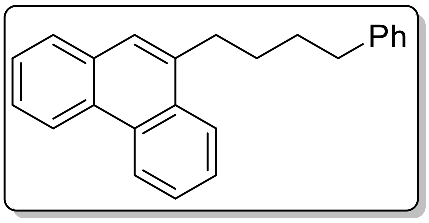

**9-(4-Phenylbutyl) phenanthrene** (**3ga**). The representative procedure was followed using phenanthrene-9-carbonyl chloride (**1g**) (48.00 mg, 0.20 mmol) and (4-bromobutyl) benzene (**2a**) (84.8 mg, 0.40 mmol). Isolation by column chromatography (*n*-hexane) yielded **3ga** (40.56 mg, 78%) as a colorless oil; **$^1$H NMR (500 MHz, $CDCl_3$)** $\delta$ 8.64 (d, *J* = 7.5 Hz, 1H), 8.56 (d, *J* = 8.0 Hz, 1H), 7.99 (d, *J* = 7.5 Hz, 1H), 7.72 (d, *J* = 7.5 Hz, 1H), 7.58-7.53 (m, 2H), 7.52-7.46 (m, 3H), 7.21-7.18 (m, 2H), 7.15-7.09 (m, 3H), 3.05 (t, *J* = 7.5 Hz, 2H), 2.61 (t, *J* = 7.5 Hz, 2H), 1.83-1.76 (m, 2H), 1.75-1.71 (m, 2H); **$^{13}$C NMR (125 MHz, $CDCl_3$)** $\delta$ 142.5, 136.6, 131.9, 131.2, 130.7, 129.6, 128.4, 128.3, 128.0, 126.5, 126.4, 126.1, 126.0, 125.9, 125.7, 124.4, 123.2, 122.4, 33.8, 33.3, 31.6, 29.8; **HR-MS** (ESI+): *m*/*z* calcd. for [Chemical Formula: $C_{24}H_{22}$ + H]$^+$ 311.1794, found 311.1793.

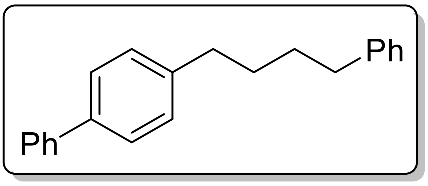

**4-(4-Phenylbutyl)-1,1'-biphenyl** (**3ha**).[13] The representative procedure was followed using [1,1'-biphenyl]-4-carbonyl chloride (**1h**) (43.20 mg, 0.20 mmol) and (4-bromobutyl) benzene (**2a**) (84.8 mg, 0.40 mmol). Isolation by column chromatography (*n*-hexane) yielded 3ha (40.56 mg, 78%) as a colorless oil; **$^1$H NMR (500 MHz, $CDCl_3$)** $\delta$ 7.50 (d, *J* = 7.5 Hz, 2H), 7.43 (d, *J* = 8.0 Hz, 2H), 7.35 (t, *J* = 7.5 Hz, 2H), 7.24 (t, *J* = 7.5 Hz, 1H), 7.20 (t, *J* = 7.5 Hz, 2H), 7.17-7.15 (m, 2H), 7.11-7.10 (m, 3H), 2.60-2.58 (m, 4H), 1.64-1.61 (m, 4H); **$^{13}$C NMR (125 MHz,**

**$CDCl_3$)** $\delta$ 142.5, 141.7, 141.1, 138.6, 128.8, 128.7, 128.4, 128.2, 126.99, 126.97, 126.95, 125.7, 35.8, 35.4, 31.1, 31.0; **MS** (EI) *m/z* (relative intensity): 286 ($M^+$, 50), 209 (100), 181 (60), 133 (40), 91 (60).

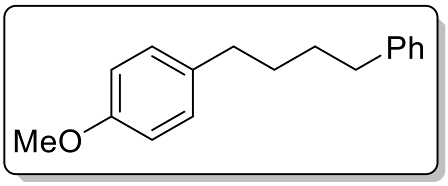


**1-Methoxy-4-(4-phenylbutyl) benzene** (**3ia**).[1] The representative procedure was followed using [1,1'-biphenyl]-4-carbonyl chloride (**1i**) (43.20 mg, 0.20 mmol) and (4-bromobutyl) benzene (**2a**) (84.8 mg, 0.40 mmol). Isolation by column chromatography (*n*-hexane : EtOAc = 100 : 1) yielded **3ia** (31.68 mg, 66%) as a colorless oil; **$^1$H NMR (500 MHz, $CDCl_3$)** $\delta$ 7.27-7.24 (m, 2H), 7.17-7.14 (m, 3H), 7.07 (d, $J$ = 8.0 Hz, 2H), 6.80 (d, $J$ = 8.0 Hz, 2H), 3.76 (s, 3H), 2.65-2.60 (m, 2H), 2.58-2.55 (m, 2H), 1.65-1.58 (m, 4H); **$^{13}$C NMR (125 MHz, $CDCl_3$)** $\delta$ 157.6, 142.6, 134.6, 129.2, 128.4, 128.2, 125.6, 113.6, 55.2, 35.8, 34.8, 31.3, 31.0; **MS** (EI) *m/z* (relative intensity): 240 ($M^+$, 50), 209 (100), 149 (60), 119 (40), 91 (60).

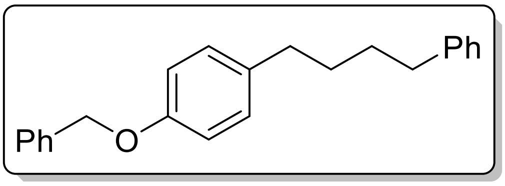


**1-(Benzyloxy)-4-(4-phenylbutyl) benzene** (**3ja**). The representative procedure was followed using 4-(benzyloxy) benzoyl chloride (**1j**) (49.20 mg, 0.20 mmol) and (4-bromobutyl) benzene (**2a**) (84.8 mg, 0.40 mmol). Isolation by column chromatography (*n*-hexane : EtOAc = 100 : 1) yielded 3ja (38.55 mg, 61%) as a colorless oil; **$^1$H NMR (500 MHz, $CDCl_3$)** $\delta$ 7.35 (d, $J$ = 7.5 Hz, 2H), 7.30 (t, $J$ = 7.5 Hz, 2H), 7.27-7.23 (m, 2H), 7.21-7.17 (m, 2H), 7.11-7.08 (s, 2H), 7.00 (d, $J$ = 8.0 Hz, 2H), 6.81 (d, $J$ = 8.0 Hz, 2H), 4.96 (s, 2H), 2.57-2.54 (m, 2H), 2.51-2.49 (m, 2H), 1.61-1.51 (m, 4H); **$^{13}$C NMR (125 MHz, $CDCl_3$)** $\delta$ 156.9, 142.6, 137.2, 134.9, 129.3, 128.5, 128.4, 128.2, 127.5, 125.6, 114.6, 99.9, 70.0, 35.8, 34.9, 31.3, 31.0; **HR-MS**

(ESI+): *m*/*z* calcd. for [Chemical Formula: $C_{23}H_{24}O + H]^+$ 317.1900, found 317.1899.

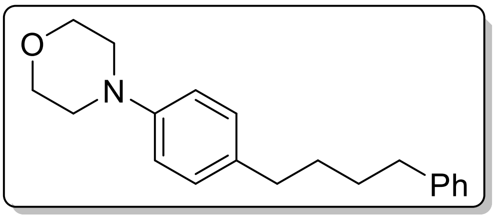


**4-[4-(4-Phenylbutyl) phenyl] morpholine** (**3ka**). The representative procedure was followed using 4-morpholinobenzoyl chloride (**1k**) (45.00 mg, 0.20 mmol) and (4-bromobutyl) benzene (**2a**) (84.8 mg, 0.40 mmol). Isolation by column chromatography (*n*-hexane : EtOAc = 50 : 1) yielded **3ka** (34.22 mg, 58%) as a colorless oil; **$^1$H NMR (500 MHz, $CDCl_3$)** $\delta$ 7.19-7.16 (m, 2H), 7.11-7.06 (m, 3H), 6.99 (d, *J* = 8.5 Hz, 2H), 6.74 (d, *J* = 8.5 Hz, 2H), 3.76 (t, *J* = 5.0 Hz, 4H), 3.01 (t, *J* = 5.0.0 Hz, 4H), 2.55-2.52 (m, 2H), 2.49-2.46 (m, 2H), 1.60-1.52 (m, 4H); **$^{13}$C NMR (125 MHz, $CDCl_3$)** $\delta$ 149.3, 142.5, 134.2, 129.0, 128.3, 128.2, 125.5, 115.8, 66.9, 49.7, 35.8, 34.8, 31.2, 31.0; **HR-MS** (ESI+): *m*/*z* calcd. for [Chemical Formula: $C_{20}H_{25}NO + H]^+$ 296.2009, found 296.2009.

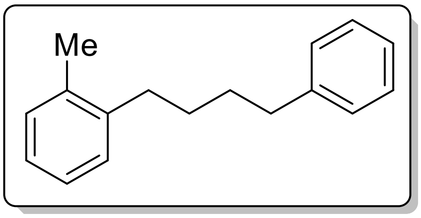


**1-Methyl-2-(4-phenylbutyl) benzene** (**3la**).[13] The representative procedure was followed using 2-methylbenzoyl chloride (**1l**) (30.80 mg, 0.20 mmol) and (4-bromobutyl) benzene (**2a**) (84.8 mg, 0.40 mmol). Isolation by column chromatography (*n*-hexane) yielded **3la** (39.42 mg, 88%) as a colorless oil; **$^1$H NMR (500 MHz, $CDCl_3$)** $\delta$ 7.27-7.22 (m, 2H), 7.19-7.14 (m, 3H), 7.12-7.07 (m, 4H), 2.65-2.59 (m, 4H), 2.27 (s, 3H), 1.73-1.67 (m, 2H), 1.63-1.57 (m, 2H); **$^{13}$C NMR (125 MHz, $CDCl_3$)** $\delta$ 142.5, 140.7, 135.8, 130.1, 128.8, 128.4, 128.2, 125.82, 125.76, 125.6, 35.8, 33.1, 31.4, 29.8, 19.3; **MS** (EI) *m/z* (relative intensity): 224 ($M^+$, 60), 119 (60), 113 (100), 119 (60), 105 (30).

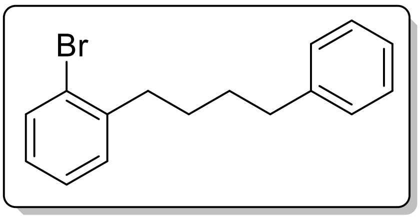


**1-Bromo-2-(4-phenylbutyl) benzene (3ma).**[14] The representative procedure was followed using 2-bromobenzoyl chloride (**1m**) (43.80 mg, 0.20 mmol) and (4-bromobutyl) benzene (**2a**) (84.8 mg, 0.40 mmol). Isolation by column chromatography (*n*-hexane) yielded **3ma** (36.86 mg, 64%) as a colorless oil; **$^1$H NMR (500 MHz, $CDCl_3$)** $\delta$ 8.04 (d, *J* = 7.5 Hz, 1H), 7.45 (t, *J* = 7.5 Hz, 1H), 7.29-7.24 (m, 4H), 7.18-7.14 (m, 3H), 3.08-3.05 (m, 2H), 2.66-2.64 (m, 2H), 1.74-1.67 (m, 4H); **$^{13}$C NMR (125 MHz, $CDCl_3$)** $\delta$ 146.5, 142.6, 133.8, 132.8, 131.7, 131.2, 128.4, 123.2, 125.9, 125.6, 35.7, 34.4, 31.4, 31.3; **MS** (EI) *m/z* (relative intensity): 290 ($M^+$, 60), 288 (60), 209 (100), 196 (30), 168 (30).

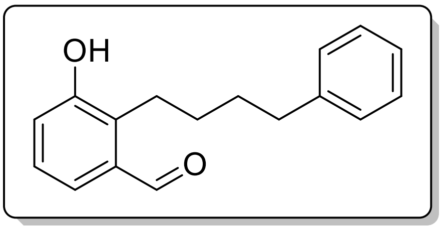


**3-Hydroxy-2-(4-phenylbutyl) benzaldehyde** (**3na**). The representative procedure was followed using 2-formyl-6-hydroxybenzoyl chloride (**1n**) (36.80 mg, 0.20 mmol) and (4-bromobutyl) benzene (**2a**) (84.8 mg, 0.40 mmol). Isolation by column chromatography (*n*-hexane : EtOAc = 10 : 1) yielded **3na** (25.91 mg, 51%) as a colorless oil; **$^1$H NMR (500 MHz, $CDCl_3$)** $\delta$ 9.98-9.96 (m, 1H), 7.44-7.41 (m, 2H), 7.38-7.35 (m, 1H), 7.38-7.35 (m, 1H), 7.30-7.26 (m, 2H), 7.21-7.16 (m, 3H), 4.03-4.01 (m, 2H), 2.73-2.68 (m, 2H), 1.87-1.81 (m, 4H); **$^{13}$C NMR (125 MHz, $CDCl_3$)** $\delta$ 192.2, 159.6, 142.0, 137.7, 130.0, 128.4, 128.3, 123.4, 123.4, 121.9, 112.6, 68.0, 35.5, 28.7, 27.7; **HR-MS** (ESI+): *m*/*z* calcd. for [Chemical Formula: $C_{17}H_{18}O_2$ + H]$^+$ 255.1380, found 255.1384.

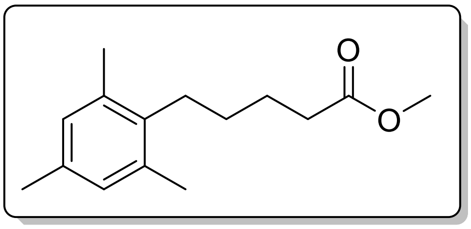

**Methyl 5-mesitylpentanoate** (**3oq**). The representative procedure was followed using 2,4,6-trimethylbenzoyl chloride (**1o**) (36.50 mg, 0.20 mmol) and methyl 5-bromopentanoate (**2q**) (78.00 mg, 0.40 mmol). Isolation by column chromatography (*n*-hexane : EtOAc = 100 : 1) yielded **3oq** (25.70 mg, 55%) as a white solid; **$^1$H NMR (500 MHz, $CDCl_3$)** $\delta$ 6.84 (s, 2H), 4.12 (t, *J* = 6.5 Hz, 2H), 2.61 (t, *J* = 8.5 Hz, 2H), 2.29 (s, 6H), 2.27 (s, 3H), 2.05 (s, 3H), 1.79-1.73 (m, 2H), 1.56-1.49 (m, 2H); **$^{13}$C NMR (125 MHz, $CDCl_3$)** $\delta$ 171.2, 135.9, 135.8, 135.0, 128.9, 64.2, 28.9, 28.8, 25.5, 21.0, 20.7, 19.7; **HR-MS** (ESI+): *m/z* calcd. for [Chemical Formula: $C_{17}H_{18}O_2$ + H]$^+$ 235.1692, found 235.1691.

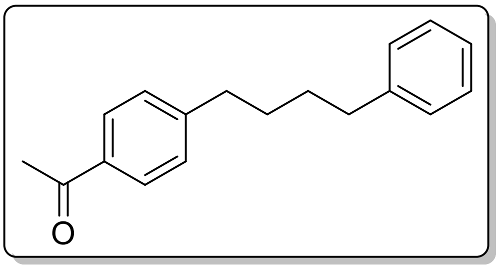

**1-[4-(4-Phenylbutyl) phenyl] ethan-1-one**[1] (**3pa**). The representative procedure was followed using 4-acetylbenzoyl chloride (**1p**) (36.40 mg, 0.20 mmol) and (4-bromobutyl) benzene (**2a**) (84.8 mg, 0.40 mmol). Isolation by column chromatography (*n*-hexane : EtOAc = 30 : 1) yielded **3pa** (35.28 mg, 70%) as a colorless oil; **$^1$H NMR (500 MHz, $CDCl_3$)** $\delta$ 7.79 (d, *J* = 7.0 Hz, 2H), 7.21-7.20 (m, 1H), 7.19-7.17 (m, 2H), 7.14-7.10 (m, 2H), 7.09-7.08 (m, 2H), 2.63-2.60 (m, 2H), 2.57-2.55 (m, 2H), 2.50 (s, 3H), 1.63-1.57 (m, 4H); **$^{13}$C NMR (125 MHz, $CDCl_3$)** $\delta$ 197.87, 148.4, 142.3, 134.9, 128.6, 128.5, 128.4, 128.3, 125.7, 35.8, 35.7, 31.0, 30.6, 26.5; **MS** (EI) *m/z* (relative intensity): 252 ($M^+$, 50), 237 (100), 209 (40), 133 (30).

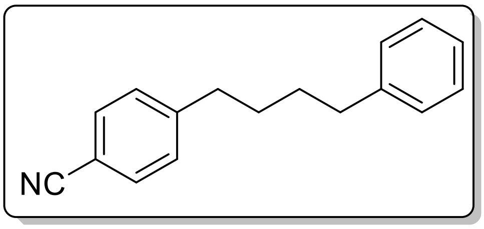

**4-(4-Phenylbutyl) benzonitrile** (**3qa**).[1] The representative procedure was followed using 4-cyanobenzoyl chloride (**1q**) (32.80 mg, 0.20 mmol) and (4-bromobutyl) benzene (**2a**) (84.8 mg, 0.40 mmol). Isolation by column chromatography (*n*-hexane : EtOAc = 10 : 1) yielded **3qa** (23.50 mg, 50%) as a colorless oil; **$^1$H NMR (500 MHz, $CDCl_3$)** $\delta$ 7.46 (d, $J$ = 8.0 Hz, 2H), 7.21-7.16 (m, 4H), 7.12-7.07 (m, 3H), 2.60 (t, $J$ = 7.0 Hz, 2H), 2.55 (t, $J$ = 6.5 Hz, 2H), 1.61-1.55 (m, 4H); **$^{13}$C NMR (125 MHz, $CDCl_3$)** $\delta$ 148.1, 142.1, 133.3, 132.6, 132.1, 129.1, 128.32, 128.29, 125.8, 119.1, 109.5, 35.9, 35.6, 30.8, 30.4; **MS** (EI) *m/z* (relative intensity): 235 ($M^+$, 50), 209 (100), 119 (40), 116 (50), 91 (30).

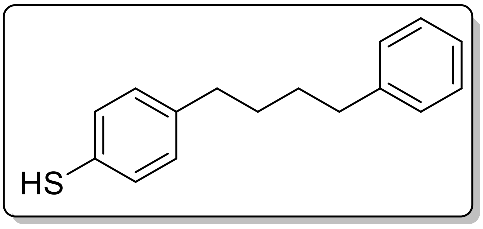

**4-(4-Phenylbutyl) benzenethiol** (**3ra**). The representative procedure was followed using 4-cyanobenzoyl chloride (**1r**) (32.80 mg, 0.20 mmol) and (4-bromobutyl) benzene (**2a**) (84.8 mg, 0.40 mmol). Isolation by column chromatography (*n*-hexane : EtOAc = 10 : 1) yielded **3ra** (29.52 mg, 61%) as a colorless oil; **$^1$H NMR (500 MHz, $CDCl_3$)** $\delta$ 7.25-7.22 (m, 2H), 7.15 (t, $J$ = 6.5 Hz, 2H), 7.08-7.04 (m, 2H), 7.03-7.00 (m, 3H), 2.75 (t, $J$ = 6.5 Hz, 2H), 2.49 (t, $J$ = 6.5 Hz, 2H), 1.65-1.59 (m, 2H), 1.56-1.51 (m, 2H); **$^{13}$C NMR (125 MHz, $CDCl_3$)** $\delta$ 141.8, 136.0, 131.7, 130.3, 128.24, 128.22, 125.7, 119.3, 35.2, 33.3, 30.3, 28.3; **HR-MS** (ESI+): *m*/*z* calcd. for [Chemical Formula: $C_{16}H_{18}S$ + H]$^+$ 243.1202, found 243.1206.

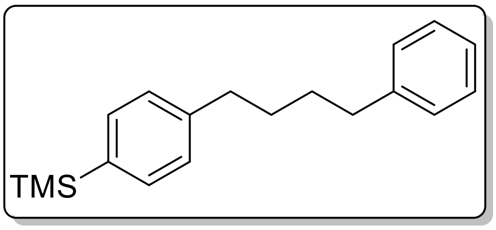

**Trimethyl[4-(4-phenylbutyl) phenyl] silane** (**3sa**).[1] The representative procedure was followed using 4-(trimethylsilyl) benzoyl chloride (**1s**) (42.40 mg, 0.20 mmol) and (4-bromobutyl) benzene (**2a**) (84.8 mg, 0.40 mmol). Isolation by column chromatography (*n*-hexane : EtOAc = 100 : 1) yielded **3sa** (40.61 mg, 72%) as a colorless oil; **$^{1}$H NMR (500 MHz, CDCl$_3$)** $\delta$ 7.52-7.50 (m, 1H), 7.36-7.34 (m, 2H), 7.20-7.17 (m, 2H), 7.08 (d, $J$ = 7.5 Hz, 4H), 2.59-2.51 (m, 4H), 1.62-1.56 (m, 4H), 0.17 (s, 9H); **$^{13}$C NMR (125 MHz, CDCl$_3$)** $\delta$ 143.2, 142.5,5 137.2, 133.8, 133.3, 128.4, 128.2, 127.9, 126.5, 125.6, 35.8, 35.78, 31.2, 31.0, 1.1; **MS** (EI) *m/z* (relative intensity): 282 ($M^+$, 50), 267 (100), 209 (30), 163 (40), 119 (50).

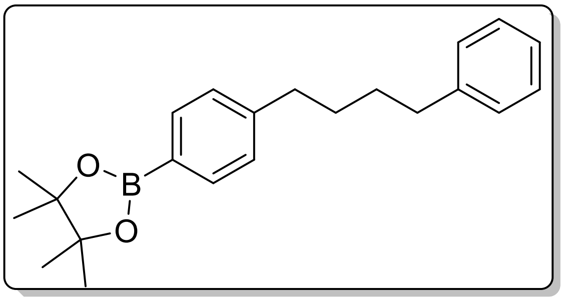

**4,4,5,5-Tetramethyl-2-[4-(4-phenylbutyl) phenyl]-1,3,2-dioxaborolane** (**3ta**).[15] The representative procedure was followed using 4-(4,4,5,5-tetramethyl-1,3,2-dioxaborolan-2-yl) benzoyl chloride (**1t**) (53.20 mg, 0.20 mmol) and (4-bromobutyl) benzene (**2a**) (84.8 mg, 0.40 mmol). Isolation by column chromatography (*n*-hexane : EtOAc = 30 : 1) yielded **3ta** (53.09 mg, 79%) as a colorless oil; **$^{1}$H NMR (500 MHz, CDCl$_3$)** $\delta$ 7.64 (d, $J$ = 8.0 Hz, 2H), 7.18 (t, $J$ = 7.5 Hz, 2H), 7.15-7.09 (m, 3H), 7.07 (d, $J$ = 8.0 Hz, 2H), 2.58-2.53 (m, 4H), 1.62-1.55 (m, 4H), 1.25 (s, 12H); **$^{13}$C NMR (125 MHz, CDCl$_3$)** $\delta$ 146.0, 142.5, 134.8, 128.4, 128.2, 127.9, 125.6, 83.6, 36.0, 35.8, 30.9, 24.8; **MS** (EI) *m/z* (relative intensity): 336 ($M^+$, 50), 321 (50), 306 (30), 209 (100), 119 (50).

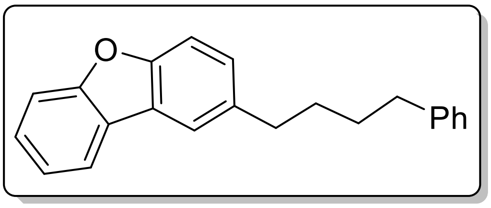


**2-(4-Phenylbutyl) dibenzo[*b,d*]furan** (**3ua**). The representative procedure was followed using dibenzo[*b*,*d*]furan-2-carbonyl chloride (**1u**) (46.00 mg, 0.20 mmol) and (4-bromobutyl) benzene (**2a**) (84.8 mg, 0.40 mmol). Isolation by column chromatography (*n*-hexane : EtOAc = 20 : 1) yielded **3ua** (22.80 mg, 38%) as a colorless oil; **$^{1}$H NMR (500 MHz, $CDCl_3$)** $\delta$ 7.90 (d, *J* = 7.5 Hz, 1H), 7.72 (s, 1H), 7.53 (d, *J* = 8.5 Hz, 1H), 7.46-7.40 (m, 2H), 7.31 (t, *J* = 7.5 Hz, 1H), 2.28-7.22 (m, 3H), 7.18-7.15 (m, 3H), 2.78 (t, *J* = 7.5 Hz, 2H), 2.65 (t, *J* = 7.5 Hz, 2H), 1.76-1.67 (m, 4H); **$^{13}$C NMR (125 MHz, $CDCl_3$)** $\delta$ 156.4, 154.6, 142. 5, 137.0, 128.4, 128.3, 127.6, 126.9, 124.2, 124.1, 122.5, 120.5, 120.0, 111.6, 111.2, 35.8, 35.7, 31.7, 31.0; **HR-MS** (ESI+): *m*/*z* calcd. for [Chemical Formula: $C_{22}H_{20}O + H]^+$ 301.1587, found 301.1588.

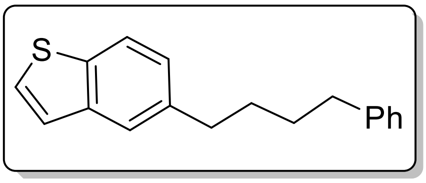


**5-(4-Phenylbutyl) benzo[*b*]thiophene** (**3va**). The representative procedure was followed using benzo[*b*]thiophene-5-carbonyl chloride (**1v**) (39.20 mg, 0.20 mmol) and (4-bromobutyl) benzene (**2a**) (84.8 mg, 0.40 mmol). Isolation by column chromatography (*n*-hexane : EtOAc = 20 : 1) yielded **3va** (22.80 mg, 41%) as a colorless oil; **$^{1}$H NMR (500 MHz, $CDCl_3$)** $\delta$ 7.76 (d, *J* = 8.0 Hz, 1H), 7.59 (s, 1H), 7.38 (d, *J* = 5.0 Hz, 1H), 7.38-7.24 (m, 3H), 7.18-7.16 (m, 2H), 7.15-7.14 (m, 2H), 2.74 (t, *J* = 7.5 Hz, 2H), 2.63 (t, *J* = 7.5 Hz, 2H), 1.74-1.64 (m, 4H); **$^{13}$C NMR (125 MHz, $CDCl_3$)** $\delta$ 142.5, 139.9, 138.6, 137.1, 128.4, 128.2, 126.4, 125.6, 125.4, 123.6, 122.9, 122.1, 35.8, 35.7, 31.4, 31.0; **HR-MS** (ESI+): *m*/*z* calcd. for [Chemical

Formula: $C_{18}H_{18}S + H]^+$ 267.1202, found 267.1204.

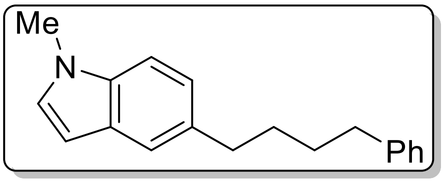


**1-Methyl-5-(4-phenylbutyl)-1*H*-indole** (**3wa**). The representative procedure was followed using 1-methyl-1*H*-indole-5-carbonyl chloride (**1w**) (38.60 mg, 0.20 mmol) and (4-bromobutyl) benzene (**2a**) (84.8 mg, 0.40 mmol). Isolation by column chromatography (*n*-hexane : EtOAc = 30 : 1) yielded **3wa** (31.56 mg, 60%) as a colorless oil; **$^1$H NMR (500 MHz, CDCl$_3$)** $\delta$ 7.33 (s, 1H), 7.20-7.14 (m, 3H), 7.10-7.07 (m, 3H), 6.96 (d, *J* = 8.5 Hz, 1H), 6.92 (d, *J* = 3.0 Hz, 1H), 6.32 (d, *J* = 2.5 Hz, 1H), 3.67 (s, 3H), 2.65 (t, *J* = 7.0 Hz, 2H), 2.56 (t, *J* = 7.5 Hz, 2H), 1.66-1.59 (m, 4H); **$^{13}$C NMR (125 MHz, CDCl$_3$)** $\delta$ 142.8, 135.3, 133.4, 128.8, 128.6, 128.4, 128.2, 125.5, 122.5, 119.9, 108.9, 100.3, 35.9, 35.8, 32.8, 32.0, 31.1; **HR-MS** (ESI+): *m*/*z* calcd. for [Chemical Formula: $C_{19}H_{21}N + H]^+$ 264.1747, found 264.1749.

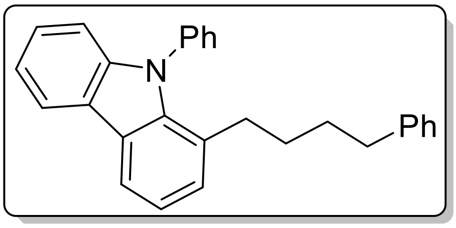


**9-Phenyl-1-(4-phenylbutyl)-9*H*-carbazole** (**3xa**). The representative procedure was followed using 9-phenyl-9*H*-carbazole-1-carbonyl chloride (**1x**) (61.00 mg, 0.20 mmol) and (4-bromobutyl) benzene (**2a**) (84.8 mg, 0.40 mmol). Isolation by column chromatography (*n*-hexane : EtOAc = 30 : 1) yielded **3xa** (58.50 mg, 78%) as a colorless oil; **$^1$H NMR (500 MHz, CDCl$_3$)** $\delta$ 7.99 (d, *J* = 8.0 Hz, 1H), 7.82 (s, 1H), 7.48-7.46 (m, 1H), 7.45-7.43 (m, 3H), 7.33-7.27 (m, 3H), 7.21 (d, *J* = 8.5 Hz, 1H), 7.18-7.14 (m, 3H), 7.10 (d, *J* = 8.0 Hz, 1H), 7.08-7.05 (m, 3H), 2.72 (t, *J* = 7.0 Hz, 2H), 2.56 (t, *J* = 7.0 Hz, 2H), 1.71-1.59 (m, 4H); **$^{13}$C NMR (125 MHz, CDCl$_3$)** $\delta$ 142.6, 141.0, 139.3, 137.8, 134.2, 129.8, 128.4, 128.2, 127.2, 126.9, 126.6, 125.7,

125.6, 123.4, 123.2, 120.2, 119.7, 119.6, 109.7 ,109.5, 35.9, 35.8, 31.9, 31.1; **HR-MS** (ESI+): *m*/*z* calcd. for [Chemical Formula: $C_{28}H_{25}N$ + H]$^+$ 376.2060, found 376.2064.

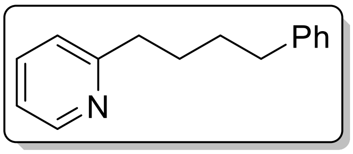

**2-(4-Phenylbutyl) pyridine** (**3ya**). The representative procedure was followed using picolinoyl chloride (**1y**) (28.20 mg, 0.20 mmol) and (4-bromobutyl) benzene (**2a**) (84.8 mg, 0.40 mmol). Isolation by column chromatography (*n*-hexane : EtOAc = 30 : 1) yielded **3ya** (17.30 mg, 41%) as a colorless oil; **$^1$H NMR (500 MHz, CDCl$_3$)** $\delta$ 8.51 (d, *J* = 4.5 Hz, 1H), 7.58-7.55 (m, 1H), 7.28-7.25 (m, 2H), 7.17-7.16 (m, 3H), 7.12-7.07 (m, 2H), 2.81 (t, *J* = 7.5 Hz, 2H), 2.65 (t, *J* = 7.5 Hz, 2H), 1.82-1.76 (m, 2H), 1.72-1.66 (m, 2H); **$^{13}$C NMR (125 MHz, CDCl$_3$)** $\delta$ 162.1, 149.1, 142.4, 136.8, 136.2, 128.3, 128.2, 125.6, 123.6, 122.6, 121.0, 120.8, 38.2, 35.7, 31.1, 29.4; **MS** (EI) *m/z* (relative intensity): 211 (M$^+$, 50), 120 (100), 119 (30).

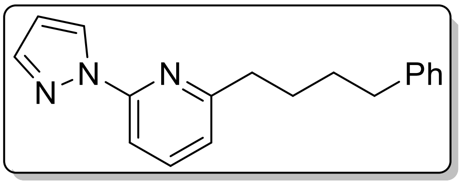

**2-(4-Phenylbutyl)-6-(1*H*-pyrazol-1-yl)pyridine** (**3za**).[16] The representative procedure was followed using 6-(1*H*-pyrazol-1-yl) picolinoyl chloride (**1z**) (41.40 mg, 0.20 mmol) and (4-bromobutyl) benzene (**2a**) (84.8 mg, 0.40 mmol). Isolation by column chromatography (*n*-hexane : EtOAc = 30 : 1) yielded **3za** (23.27 mg, 42%) as a colorless oil; **$^1$H NMR (500 MHz, CDCl$_3$)** $\delta$ 8.57 (d, *J* = 2.0 Hz, 1H), 7.77-7.67 (m, 3H), 7.27 (t, *J* = 8.0 Hz, 2H), 7.17 (d, *J* = 6.0 Hz, 3H), 7.00 (d, *J* = 7.5 Hz, 1H), 6.47-6.43 (m, 1H), 2.81 (t, *J* = 7.5 Hz, 2H), 2.67 (t, *J* = 7.5 Hz, 2H), 1.86-1.79 (m, 2H), 1.74-1.68 (m, 2H); **$^{13}$C NMR (125 MHz, CDCl$_3$)** $\delta$ 161.0, 151.0, 142.4, 141.8, 138.8, 128.4, 128.3, 127.0, 125.7, 120.3, 109.3, 107.3, 37.6, 35.8, 31.0, 28.9; **HR-MS** (ESI+): *m*/*z* calcd. for [Chemical Formula: $C_{18}H_{19}N_3$ + H]$^+$ 278.1652, found 278.1655.

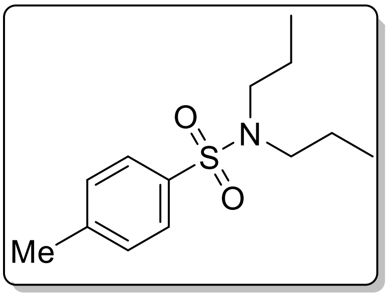


**4-Methyl-*N*,*N*-dipropylbenzenesulfonamide** (**6ab**).[17] The representative procedure was followed using 4-(*N*,*N*-dipropylsulfamoyl) benzoyl chloride (**5a**) (60.60 mg, 0.20 mmol) and methyl 4-methylbenzenesulfonate (**2b**) (196.8 mg, 0.60 mmol). Isolation by column chromatography (*n*-hexane : EtOAc = 2 : 1) yielded **6ab** (39.95 mg, 78%) as a colorless oil; **$^{1}$H NMR (500 MHz, $CDCl_3$)** $\delta$ 7.80 (dd, *J* = 1.5 Hz, 8.5 Hz, 1H), 7.68 (d, *J* = 8.5 Hz, 1H), 7.57-7.50 (m, 1H), 7.28 (d, *J* = 8.5 Hz, 1H), 3.10-3.04 (m, 4H), 2.41 (s, 3H), 1.58-1.52 (m, 4H), 0.87 (t, *J* = 7.5 Hz, 6H); **$^{13}$C NMR (125 MHz, $CDCl_3$)** $\delta$ 142.8, 140.1, 137.1, 132.2, 129.5, 128.9, 127.02, 126.96, 49.99, 49.95, 21.98, 21.95, 21.4, 11.13, 11.11; **HR-MS** (ESI+): *m*/*z* calcd. for [Chemical Formula: $C_{13}H_{21}NO_2S$ + H]$^+$ 256.1366, found 256.1369.

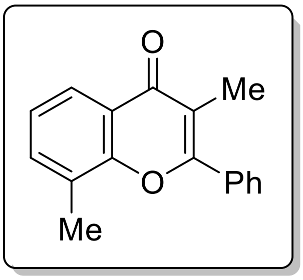


**3,8-Dimethyl-2-phenyl-4*H*-chromen-4-one** (**6bb**). The representative procedure was followed using 3-methyl-4-oxo-2-phenyl-4*H*-chromene-8-carbonyl chloride (**5b**) (59.60 mg, 0.20 mmol) and methyl 4-methylbenzenesulfonate (**2b**) (196.8 mg, 0.60 mmol). Isolation by column chromatography (*n*-hexane : EtOAc = 1 : 1) yielded **6bb** (27.62 mg, 55%) as a colorless oil; **$^{1}$H NMR (500 MHz, $CDCl_3$)** $\delta$ 8.09 (d, *J* = 9.0 Hz, 1H), 7.68-7.67 (m, 2H), 7.53-7.46 (m, 4H), 7.26 (t, *J* = 7.5 Hz, 1H), 2.46 (s, 3H), 2.19 (s, 3H); **$^{13}$C NMR (125 MHz, $CDCl_3$)** $\delta$ 179.2, 160.5, 154.5, 134.1, 133.5, 130.1, 128.9, 128.4, 127.1, 124.2, 123.3, 122.2, 117.1, 15.5, 11.7 ; **HR-MS** (ESI+): *m*/*z* calcd. for [Chemical Formula: $C_{17}H_{14}O_2$ + H]$^+$ 251.1067, found 251.1066.

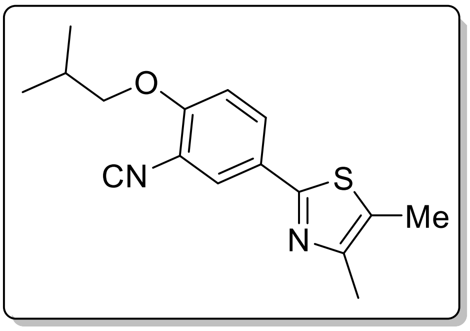


**5-(4,5-Dimethylthiazol-2-yl)-2-isobutoxybenzonitrile** (**6cb**).[17] The representative procedure was followed using 2-(3-cyano-4-isobutoxyphenyl)-4-methylthiazole-5-carbonyl chloride (**5c**) (66.80 mg, 0.20 mmol) and methyl 4-methylbenzenesulfonate (**2b**) (196.8 mg, 0.60 mmol). Isolation by column chromatography (*n*-hexane : EtOAc = 3 : 1) yielded **6cb** (41.32 mg, 72%) as a colorless oil; **$^{1}$H NMR (500 MHz, $CDCl_3$)** $\delta$ 8.07-7.92 (m, 2H), 6.96-6.84 (m, 1H), 7.53-7.46 (m, 4H), 3.84 (t, *J* = 7.0 Hz, 2H), 2.45 (s, 3H), 2.33 (d, *J* = 10.0 Hz, 3H), 2.19-2.12 (m, 1H), 1.21 (s, 3H), 1.05 (dd, *J* = 2.5 Hz, 6.5 Hz, 3H); **$^{13}$C NMR (125 MHz, $CDCl_3$)** $\delta$ 164.8, 161.5, 161.2, 160.4, 153.8, 149.3, 132.0, 131.7, 131.4, 131.0, 126.9, 126.73, 126.66, 115.7, 115.6, 113.4, 112.4, 112.3, 102.5, 102.4, 75.4, 28.0, 26.9, 18.9, 17.0, 14.6, 11.3; **HR-MS** (ESI+): *m*/*z* calcd. for [Chemical Formula: $C_{16}H_{18}N_2OS$ + H]$^+$ 287.1213, found 287.1211.

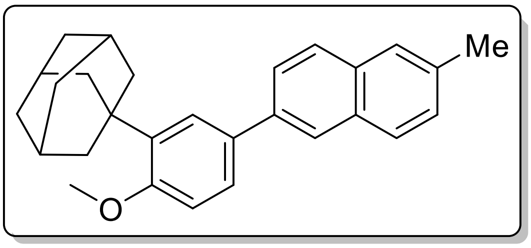


**1-(2-Methoxy-5-(6-methylnaphthalen-2-yl)phenyl)adamantane** (**6db**).[17] The representative procedure was followed using 6-(3-(adamantan-1-yl)-4-methoxyphenyl)-2-naphthoyl chloride (**5d**) (88.60 mg, 0.20 mmol) and methyl 4-methylbenzenesulfonate (**2b**) (196.8 mg, 0.60 mmol). Isolation by column chromatography (*n*-hexane : EtOAc = 20 : 1) yielded **6db** (45.19 mg, 59%) as a colorless oil; **$^{1}$H NMR (500 MHz, $CDCl_3$)** $\delta$ 7.98 (s, 2H), 7.87-7.82 (m, 3H), 7.72 (d, *J* = 8.5 Hz, 1H), 7.59 (d, *J* = 2.0 Hz, 1H), 7.52-7.43 (m, 2H), 6.97-6.95 (m, 1H), 3.87

(s, 3H), 2.28-2.16 (m, 8H), 2.10 (s, 3H), 1.91-1.72 (m, 7H); **$^{13}$C NMR (125 MHz, $CDCl_3$)** $\delta$ 158.5, 138.9, 138.8, 133.7, 133.2, 132.2, 128.2, 128.0, 127.6, 126.1, 125.9, 125.7, 125.5, 125.0, 112.0, 55.1, 40.6, 37.2, 37.1, 29.1; **HR-MS** (ESI+): *m*/*z* calcd. for [Chemical Formula: $C_{28}H_{30}O + H]^+$ 383.2369, found 383.2371.

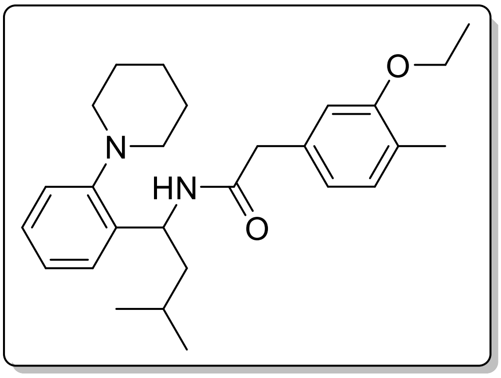

**2-(3-Ethoxy-4-methylphenyl)-*N*-{3-methyl-1-[2-(piperidin-1-yl) phenyl] butyl} acetamide** (**6eb**). The representative procedure was followed using 2-(3-cyano-4-isobutoxyphenyl)-4-methylthiazole-5-carbonyl chloride (**5e**) (94.0 mg, 0.20 mmol) and methyl 4-methylbenzenesulfonate (**2b**) (196.8 mg, 0.60 mmol). Isolation by column chromatography (*n*-hexane : EtOAc = 2 : 1) yielded **6eb** (51.49 mg, 61%) as a colorless oil; **$^1$H NMR (500 MHz, $CDCl_3$)** $\delta$ 7.53-7.15 (m, 3H), 7.08-7.03 (m, 2H), 6.82-6.77 (m, 2H), 6.61-6.59 (m, 1H), 5.40 (t, *J* = 7.5 Hz, 1H), 3.98-3.90 (m, 2H), 3.54-3.47 (m, 2H), 3.05-2.90 (m, 2H), 2.67-2.55 (m, 2H), 2.20 (s, 3H), 1.78-1.65 (m, 3H), 1.63-1.49 (m, 6H), 1.38 (s, 3H), 0.99-0.83 (m, 6H); **$^{13}$C NMR (125 MHz, $CDCl_3$)** $\delta$ 170.1, 156.4, 152.2, 138.9, 133.7, 130.8, 127.7, 127.4, 124.8, 122.5, 121.4, 120.9, 115.1, 113.5, 111.8, 63.4, 49.3, 46.6, 44.0, 26.6, 25.3, 24.2, 22.8, 22.5, 15.9, 14.8; **HR-MS** (ESI+): *m*/*z* calcd. for [Chemical Formula: $C_{27}H_{38}N_2O_2 + H]^+$ 423.3006, found 423.3008.

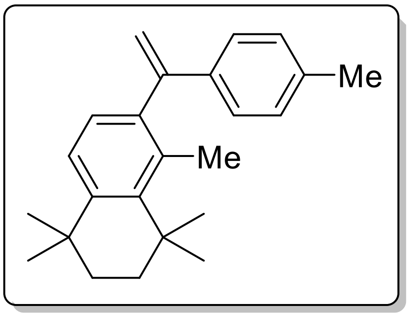


**1,1,4,4,5-Pentamethyl-6-[1-(*p*-tolyl)vinyl]-1,2,3,4-tetrahydronaphthalene** (**6fb**). The representative procedure was followed using 4-(1-(1,5,5,8,8-pentamethyl-5,6,7,8-tetrahydronaphthalen-2-yl)vinyl)benzoyl chloride (**5f**) (73.2 mg, 0.20 mmol) and methyl 4-methylbenzenesulfonate (**2b**) (196.8 mg, 0.60 mmol). Isolation by column chromatography (*n*-hexane : EtOAc = 100 : 1) yielded **6fb** (24.88 mg, 39%) as a colorless oil; **$^1$H NMR (500 MHz, $CDCl_3$)** $\delta$ 7.28-7.24 (m, 4H), 7.13 (s, 1H), 7.06 (s, 1H), 5.71 (d, $J$ = 1.5 Hz, 1H), 5.20 (d, $J$ = 1.5 Hz, 1H), 1.96 (s, 3H), 1.69 (s, 4H), 1.30 (s, 6H), 1.27 (s, 6H), 1.26-1.24 (m, 3H); **$^{13}$C NMR (125 MHz, $CDCl_3$)** $\delta$ 149.8, 143.9, 142.1, 141.0, 138.7, 121.8, 128.2, 128.0, 127.8, 127.4, 126.6, 114.6, 35.2, 34.0, 33.9, 31.91, 31.89, 19.9, 14.2; **HR-MS** (ESI+): *m*/*z* calcd. for [Chemical Formula: $C_{24}H_{30}$ + H]$^+$ 319.2420, found 319.2419.

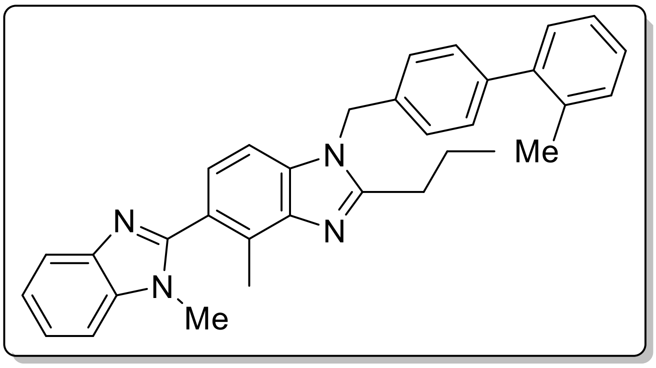


**1,4'-Dimethyl-1'-((2'-methyl-[1,1'-biphenyl]-4-yl)methyl)-2'-propyl-1*H*,1'*H*-2,5'-bibenzo[*d*]imidazole** (**6gb**). The representative procedure was followed using 4'-((1,4'-dimethyl-2'-propyl-1*H*,1'*H*-[2,5'-bibenzo[*d*]imidazol]-1'-yl)methyl)-[1,1'-biphenyl]-2-carbonyl chloride (**5g**) (106.4 mg, 0.20 mmol) and methyl 4-methylbenzenesulfonate (**2b**) (196.8 mg, 0.60 mmol). Isolation by column chromatography (*n*-hexane : EtOAc = 5 : 1) yielded **6gb** (42.70 mg, 44%) as a

colorless oil; **$^1$H NMR (500 MHz, $CDCl_3$)** $\delta$ 7.82 (dd, *J* = 1.5 Hz, 6.5 Hz, 1H), 7.49 (s, 1H), 7.42 (s, 1H), 7.32-7.28 (m, 2H), 7.26-7.25 (m, 3H), 7.24-7.18 (m, 3H), 7.14 (d, *J* = 7.0 Hz, 1H), 7.09 (d, *J* = 8.0 Hz, 2H), 5.42 (s, 2H), 3.71 (s, 3H), 2.93 (t, *J* = 7.5 Hz, 2H), 2.77 (s, 3H), 2.20 (s, 3H), 1.87-1.80 (m, 2H), 1.03 (t, *J* = 7.5 Hz, 3H); **$^{13}$C NMR (125 MHz, $CDCl_3$)** $\delta$ 156.5, 154.4, 142.7, 142.5, 141.5, 140.9, 136.4, 135.1, 134.8, 134.2, 130.2, 129.7, 129.5, 129.2, 127.3, 125.9, 125.7, 123.8, 124.6, 122.4, 122.3, 119.3, 109.4, 108.9, 46.9, 31.6, 29.5, 27.2, 21.7, 20.3, 18.3, 16.8, 13.9; **HR-MS** (ESI+): *m*/*z* calcd. for [Chemical Formula: $C_{33}H_{32}N_4$ + H]$^+$ 485.2700, found 485.2703.

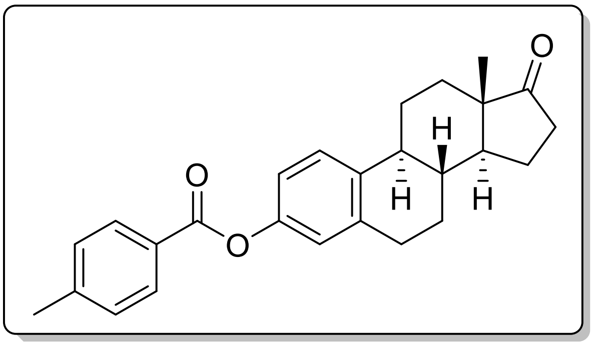


**(8*R*,9*S*,13*S*,14*S*)-13-Methyl-17-oxo-7,8,9,11,12,13,14,15,16,17-decahydro-6*H*-cyclopenta[*a*]phenanthren-3-yl 4-methylbenzoate** (**6hb**). The representative procedure was followed using (8*R*,9*S*,13*S*,14*S*)-13-methyl-17-oxo-7,8,9,11,12,13,14,15,16,17-decahydro-6*H*-cyclopenta[*a*]phenanthren-3-yl 4-(chlorocarbonyl) benzoate (**5h**) (87.20 mg, 0.20 mmol) and methyl 4-methylbenzenesulfonate (**2b**) (196.8 mg, 0.60 mmol). Isolation by column chromatography (*n*-hexane : EtOAc = 10 : 1) yielded **6hb** (53.54 mg, 69%) as a colorless oil; **$^1$H NMR (500 MHz, $CDCl_3$)** $\delta$ 8.07 (d, *J* = 8.5 Hz, 2H), 7.34-7.29 (m, 3H), 6.97 (dd, *J* = 2.5 Hz, 8.5 Hz, 1H), 6.94 (d, *J* = 2.0 Hz, 1H), 2.96-2.93 (m, 2H), 2.54-2.48 (m, 1H), 2.45 (s, 3H), 2.34-2.28 (m, 1H), 2.19-2.11 (m, 1H), 2.04-1.96 (m, 4H), 1.66-1.61 (m, 2H), 1.56-1.42 (m, 4H), 0.93 (s, 3H); **$^{13}$C NMR (125 MHz, $CDCl_3$)** $\delta$ 165.4, 148.8, 144.2, 137.9, 137.2, 130.1, 129.2, 126.8, 126.3, 121.7, 118.8, 50.4, 47.9, 44.1, 37.9, 35.8, 31.5, 29.4, 26.3, 25.7, 21.6, 21.5, 13.8; **HR-MS** (ESI+): *m*/*z* calcd. for [Chemical Formula: $C_{26}H_{28}O_3$ + H]$^+$ 389.2112, found 389.2111.

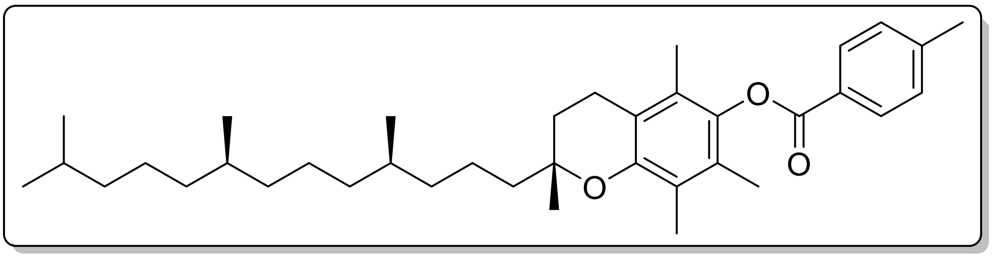

**(*R*)-2,5,7,8-Tetramethyl-2-[(4R,8R)-4,8,12-trimethyltridecyl] chroman-6-yl 4-methylbenzoate** (**6ib**). The representative procedure was followed using (*R*)-2,5,7,8-tetramethyl-2-((4*R*,8*R*)-4,8,12-trimethyltridecyl) chroman-6-yl 4-(chlorocarbonyl) benzoate (**5i**) (119.40 mg, 0.20 mmol) and methyl 4-methylbenzenesulfonate (**2b**) (196.8 mg, 0.60 mmol). Isolation by column chromatography (*n*-hexane : EtOAc = 20 : 1) yielded **6ib** (84.39 mg, 77%) as a white solid; **$^1$H NMR (500 MHz, $CDCl_3$)** $\delta$ 8.13 (d, *J* = 8.0 Hz, 2H), 7.27 (d, *J* = 8.0 Hz, 2H), 2.60 (t, *J* = 6.5 Hz, 2H), 2.42 (s, 3H), 2.12 (s, 3H), 2.05 (s, 3H), 2.01 (s, 3H), 1.82-1.75 (m, 2H), 1.60-1.53 (m, 2H), 1.54-1.43 (m, 4H), 1.40-1.29 (m, 6H), 1.27-1.23 (m, 3H), 1.16-1.12 (m, 2H), 1.10-1.05 (m, 2H), 0.88 (s, 6H), 0.86 (s, 6H), 0.85 (s, 3H); **$^{13}$C NMR (125 MHz, $CDCl_3$)** $\delta$ 165.2, 149.5, 144.1, 140.8, 130.3, 129.4, 127.0, 125.2, 123.1, 117.5, 39.5, 37.6, 37.4, 32.9, 28.1, 24.9, 24.6, 22.7, 21.8, 20.7, 19.9, 13.1, 12.2, 11.9; **HR-MS** (ESI+): *m*/*z* calcd. for [Chemical Formula: $C_{37}H_{56}O_3$ + H]$^+$ 549.4300, found 549.4302.

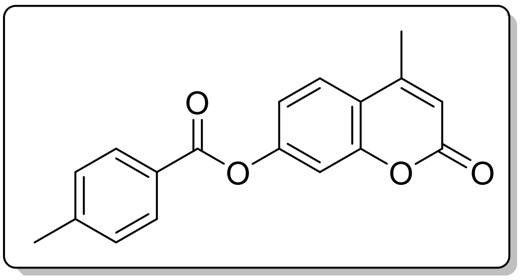

**4-Methyl-2-*oxo*-2*H*-chromen-7-yl 4-methylbenzoate** (**6jb**). The representative procedure was followed using 4-methyl-2-*oxo*-2*H*-chromen-7-yl 4-(chlorocarbonyl) benzoate (**5j**) (68.40 mg, 0.20 mmol) and methyl 4-methylbenzenesulfonate (**2b**) (196.8 mg, 0.60 mmol). Isolation by column chromatography (*n*-hexane : EtOAc = 10 : 1) yielded **6jb** (48.22 mg, 82%) as a colorless oil; **$^1$H NMR (500 MHz, $CDCl_3$)** $\delta$ 8.06 (d, *J* = 8.0 Hz, 2H), 7.63 (d, *J* = 8.5 Hz, 1H), 7.30 (d, *J* = 8.0 Hz, 2H), 7.22-7.18

(m, 2H), 6.26 (s, 1H), 2.45 (s, 3H), 2.43 (s, 3H); **$^{13}$C NMR (125 MHz, $CDCl_3$)** $\delta$ 164.5, 160.4, 154.1, 153.4, 151.9, 144.9, 130.3, 129.3, 126.0, 118.2, 117.7, 114.4, 110.5, 21.7, 18.6; **HR-MS** (ESI+): *m*/*z* calcd. for [Chemical Formula: $C_{18}H_{14}O_4$ + H]$^+$ 295.0964, found 295.09645.

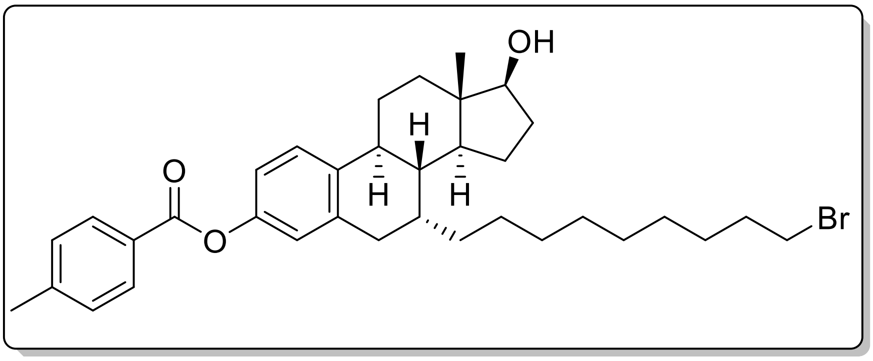


**(7*R*,8*R*,9*S*,13*S*,14*S*,17*S*)-7-(9-Bromononyl)-17-hydroxy-13-methyl-7,8,9,11,12,13,14,15,16,17-decahydro-6*H*-cyclopenta[*a*]phenanthren-3-yl 4-methylbenzoate** (**6kb**). The representative procedure was followed using (7*R*,8*R*,9*S*,13*S*,14*S*,17*S*)-7-(9-bromononyl)-17-hydroxy-13-methyl-7,8,9,11,12,13,14,15,16,17-decahydro-6*H*-cyclopenta[*a*]phenanthren-3-yl 4-(chlorocarbonyl) benzoate (**5k**) (128.80 mg, 0.20 mmol) and methyl 4-methylbenzenesulfonate (**2b**) (196.8 mg, 0.60 mmol). Isolation by column chromatography (*n*-hexane : EtOAc = 5 : 1) yielded **6kb** (72.48 mg, 61%) as a white solid; **$^1$H NMR (500 MHz, $CDCl_3$)** $\delta$ 8.06 (d, *J* = 8.0 Hz, 2H), 7.33-7.25 (m, 3H), 6.96 (dd, *J* = 2.0 Hz, 8.5 Hz, 1H), 6.91 (d, *J* = 2.0 Hz, 1H), 3.74 (t, *J* = 8.5 Hz, 1H), 3.38 (t, *J* = 6.5 Hz, 2H), 2.92 (dd, *J* = 5.0 Hz, 17.0 Hz, 1H), 2.77 (d, *J* = 16.5 Hz, 1H), 2.44 (s, 3H), 2.38-2.34 (m, 2H), 2.16-2.09 (m, 1H), 1.92 (d, *J* = 12.5 Hz, 1H), 1.86-1.80 (m, 2H), 1.76-1.73 (m, 2H), 1.68-1.59 (m, 2H), 1.52-1.48 (m, 2H), 1.47-1.36 (m, 6H), 1.29-1.25 (m, 8H), 1.20-1.19 (m, 2H), 0.78 (s, 3H); **$^{13}$C NMR (125 MHz, $CDCl_3$)** $\delta$ 165.4, 148.7, 144.2, 137.1, 137.07, 130.1, 129.2, 126.9, 122.4, 118.7, 81.8, 46.4, 43.3, 41.7, 38.2, 36.8, 34.5, 33.9,v33.1, 32.7, 30.5, 29.8, 29.5, 29.3, 28.6, 28.1, 27.1, 25.6, 22.6, 21.7, 11.0; **HR-MS** (ESI+): *m*/*z* calcd. for [Chemical Formula: $C_{35}H_{47}BrO_3$ + H]$^+$ 595.2780, found 595.2781.

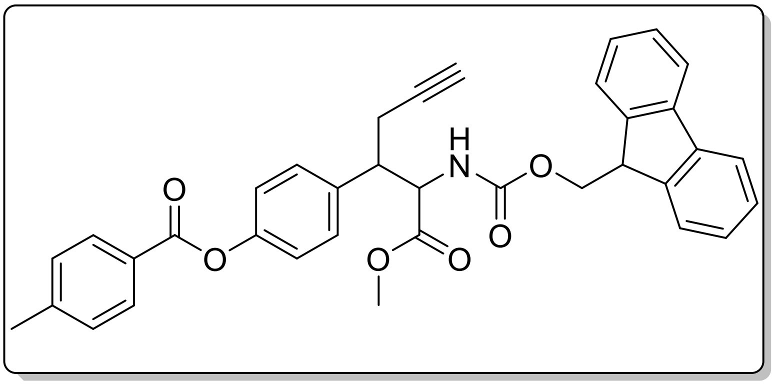

**4-(2-((((9*H*-Fluoren-9-yl)methoxy)carbonyl)amino)-1-methoxy-1-oxohex-5-yn-3-yl)phenyl 4-methylbenzoate** (**6lb**). The representative procedure was followed using 4-(2-((((9H-fluoren-9-yl)methoxy)carbonyl)amino)-1-methoxy-1-oxohex-5-yn-3-yl)phenyl 4-(chlorocarbonyl)benzoate (**5l**) (124.20 mg, 0.20 mmol) and methyl 4-methylbenzenesulfonate (**2b**) (196.8 mg, 0.60 mmol). Isolation by column chromatography (*n*-hexane : EtOAc = 2 : 1) yielded **6lb** (80.12 mg, 70%) as a white solid; **$^1$H NMR (500 MHz, CDCl$_3$)** $\delta$ 7.99 (d, *J* = 8.0 Hz, 2H), 7.67 (d, *J* = 7.5 Hz, 2H), 7.56 (d, *J* = 5.0 Hz, 2H), 7.31 (t, *J* = 7.5 Hz, 2H), 7.24-7.20 (m, 4H), 7.13 (d, *J* = 7.0 Hz, 2H), 7.04(d, *J* = 7.5 Hz, 2H), 5.98 (brs, 1H), 4.87 (q, *J* = 1.5 Hz, 1H), 4.36-4.33 (m, 2H), 4.17 (t, *J* = 7.0 Hz, 1H), 3.64 (s, 3H), 3.15-3.11 (m, 1H), 3.06-3.02 (m, 1H), 2.38 (s, 3H); **$^{13}$C NMR (125 MHz, CDCl$_3$)** $\delta$ 171.2, 169.5, 165.1, 150.0, 144.3, 143.6, 141.1, 133.1, 130.2, 130.0, 129.2, 127.6, 126.9, 124.9, 121.8, 119.9, 79.0, 72.9, 67.2, 53.3, 52.3, 37.0, 21.6; **HR-MS** (ESI+): *m/z* calcd. for [Chemical Formula: $C_{36}H_{31}NO_6$ + H]$^+$ 574.2224, found 574.2230.

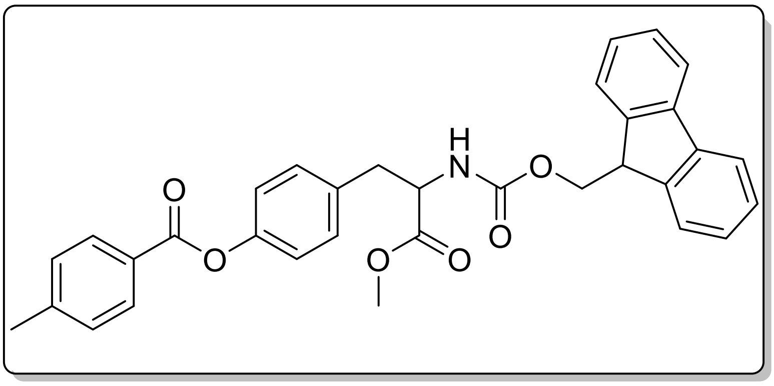

**4-(2-((((9*H*-Fluoren-9-yl)methoxy)carbonyl)amino)-3-methoxy-3-**

**oxopropyl)phenyl 4-methylbenzoate** (**6mb**). The representative procedure was followed using 4-(2-((((9*H*-fluoren-9-yl)methoxy)carbonyl)amino)-3-methoxy-3-oxopropyl)phenyl 4-(chlorocarbonyl)benzoate (**5m**) (116.80 mg, 0.20 mmol) and methyl 4-methylbenzenesulfonate (**2b**) (196.8 mg, 0.60 mmol). Isolation by column chromatography (*n*-hexane : EtOAc = 2 : 1) yielded **6mb** (86.12 mg, 82%) as a white solid; **$^1$H NMR (500 MHz, CDCl$_3$)** $\delta$ 7.99 (d, *J* = 8.0 Hz, 2H), 7.67 (d, *J* = 7.5 Hz, 2H), 7.56 (d, *J* = 5.0 Hz, 2H), 7.31 (t, *J* = 7.5 Hz, 2H), 7.24-7.20 (m, 4H), 7.13 (d, *J* = 7.0 Hz, 2H), 7.04(d, *J* = 7.5 Hz, 2H), 5.98 (brs, 1H), 4.87 (q, *J* = 1.5 Hz, 1H), 4.36-4.33 (m, 2H), 4.17 (t, *J* = 7.0 Hz, 1H), 3.64 (s, 3H), 3.15-3.11 (m, 1H), 3.06-3.02 (m, 1H), 2.38 (s, 3H); **$^{13}$C NMR (125 MHz, CDCl$_3$)** $\delta$ 171.5, 169.1, 165.0, 156.5, 149.8, 144.2, 143.6, 144.2, 143.6, 140.1, 141.0, 133.2, 130.1, 129.9, 129.1, 126.8, 126.4, 124.9, 121.6, 119.7, 67.0, 52.1, 46.8, 44.1, 38.3, 36.9, 21.5; **HR-MS** (ESI+): *m*/*z* calcd. for [Chemical Formula: $C_{33}H_{29}NO_6$ + H]$^+$ 536.2067, found 536.2069.

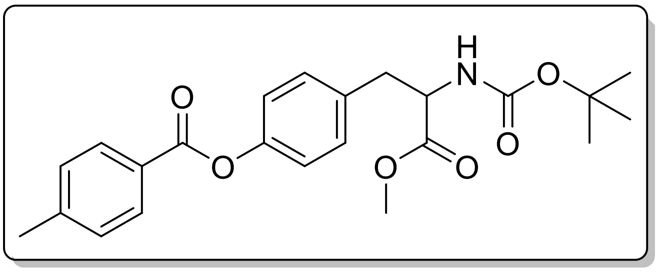

**4-{2-[(*tert*-Butoxycarbonyl) amino]-3-methoxy-3-oxopropyl} phenyl 4-methylbenzoate** (**6nb**). The representative procedure was followed using 4-(2-((*tert*-butoxycarbonyl) amino)-3-methoxy-3-oxopropyl) phenyl 4-(chlorocarbonyl)benzoate (**5n**) (92.20 mg, 0.20 mmol) and methyl 4-methylbenzenesulfonate (**2b**) (196.8 mg, 0.60 mmol). Isolation by column chromatography (*n*-hexane : EtOAc = 1 : 1) yielded **6nb** (57.82 mg, 70%) as a white solid; **$^1$H NMR (500 MHz, CDCl$_3$)** $\delta$ 7.95-7.93 (m, 2H), 7.16-7.14 (m, 2H), 7.06 (d, *J* = 8.0 Hz, 2H), 7.0.3-7.01 (m, 2H), 5.20-5.13 (m, 1H), 4.47-4.45 (m, 1H), 3.56 (s, 3H), 3.03-3.00 (m, 1H), 2.94-2.92 (m, 1H), 2.29 (s, 3H), 1.30 (s, 9H); **$^{13}$C NMR (125 MHz, CDCl$_3$)** $\delta$ 171.9, 164.7, 154.8, 148.7, 144.0, 133.5, 129.9, 129.8, 128.9, 126.4, 121.4, 59.9, 54.2, 51.8, 37.3, 27.9, 21.3, 20.5, 13.8; **HR-MS** (ESI+): *m*/*z* calcd. for [Chemical Formula: $C_{23}H_{27}NO_6$ + H]$^+$ 414.1911, found

414.1915.

# 5. Mechanistic studies

5.1 Synthesis of key nickel intermediates

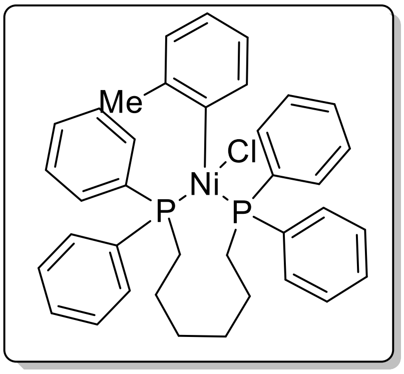


**Ni(dpph) (*o*-tolyl)Cl (9).**[19] Bis(1,5-cyclooctadiene) nickel (55.0 mg, 0.20 mmol) and 1,6-bis(diphenylphosphino)hexane (90.9 mg, 0.20 mmol) was added to an oven-dried 10 mL round bottom flask equipped with a magnetic stir-bar and purged with $N_2$. Toluene (2 mL) was added via syringe and the reaction mixture was stirred at room temperature for 3 minutes. *o*-Toluoyl chloride (29 μL, 1.0 M in toluene, 0.22 mmol) was then added dropwise via syringe. The reaction mixture was stirred for 2 min at 0˚C and an additional 30 min at room temperature and then was quenched with *n*-hexane (15 mL). The solvent was removed under vacuum and residue was redissolved in minimal volume of $CHCl_3$ (~3 mL) followed by the addition of *n*-hexane (5 mL). This solution was allowed to stand at room temperature for 12 h, during which time yellow crystals formed. The supernatant was decanted, and the crystals were dried in vacuo to afford complex as a yellow solid (62.6 mg, 49% yield). **$^1$H NMR (500 MHz, $CDCl_3$)** $\delta$ 7.70-7.50 (m, 6H), 7.40-7.25 (m, 7H), 7.20-7.11 (m, 6H), 7.10-7.00 (m, 2H), 6.33-6.10 (m, 3H), 2.38 (s, 3H), 1.43-1.36 (m, 4H), 1.19 (s, 2H), 0.89-0.64 (m, 6H); **$^{13}$C NMR (125 MHz, $CDCl_3$)** $\delta$ 137.4, 136.0, 134.1, 133.7, 132.0, 131.3, 130.7, 127.7, 123.2, 122.0, 30.4, 25.4, 23.7; **$^{31}$P NMR (121 MHz, $CDCl_3$)** $\delta$ 14.27; **HR-MS** (ESI+): *m*/*z* calcd. for [Chemical Formula: $C_{37}H_{39}ClNiP_2$ + H]$^+$ 639.1642, found 639.1450.

# Display Report

**Analysis Info**

| | | | |
|---|---|---|---|
| Analysis Name | D:\data\fenglei\TZ-3_BE5_01_22678.d | Acquisition Date | 9/22/2023 3:12:33 PM |
| Method | 1008-ms.m | Operator | Demo User |
| Sample Name | TZ-3 | Instrument | impact II 1825265.10256 |
| Comment | | | |

**Acquisition Parameter**

| | | | | | |
|---|---|---|---|---|---|
| Source Type | ESI | Ion Polarity | Positive | Set Nebulizer | 0.4 Bar |
| Focus | Not active | Set Capillary | 3500 V | Set Dry Heater | 180 °C |
| Scan Begin | 50 m/z | Set End Plate Offset | -500 V | Set Dry Gas | 4.0 l/min |
| Scan End | 10000 m/z | Set Charging Voltage | 2000 V | Set Divert Valve | Source |
| | | Set Corona | 0 nA | Set APCI Heater | 0 °C |

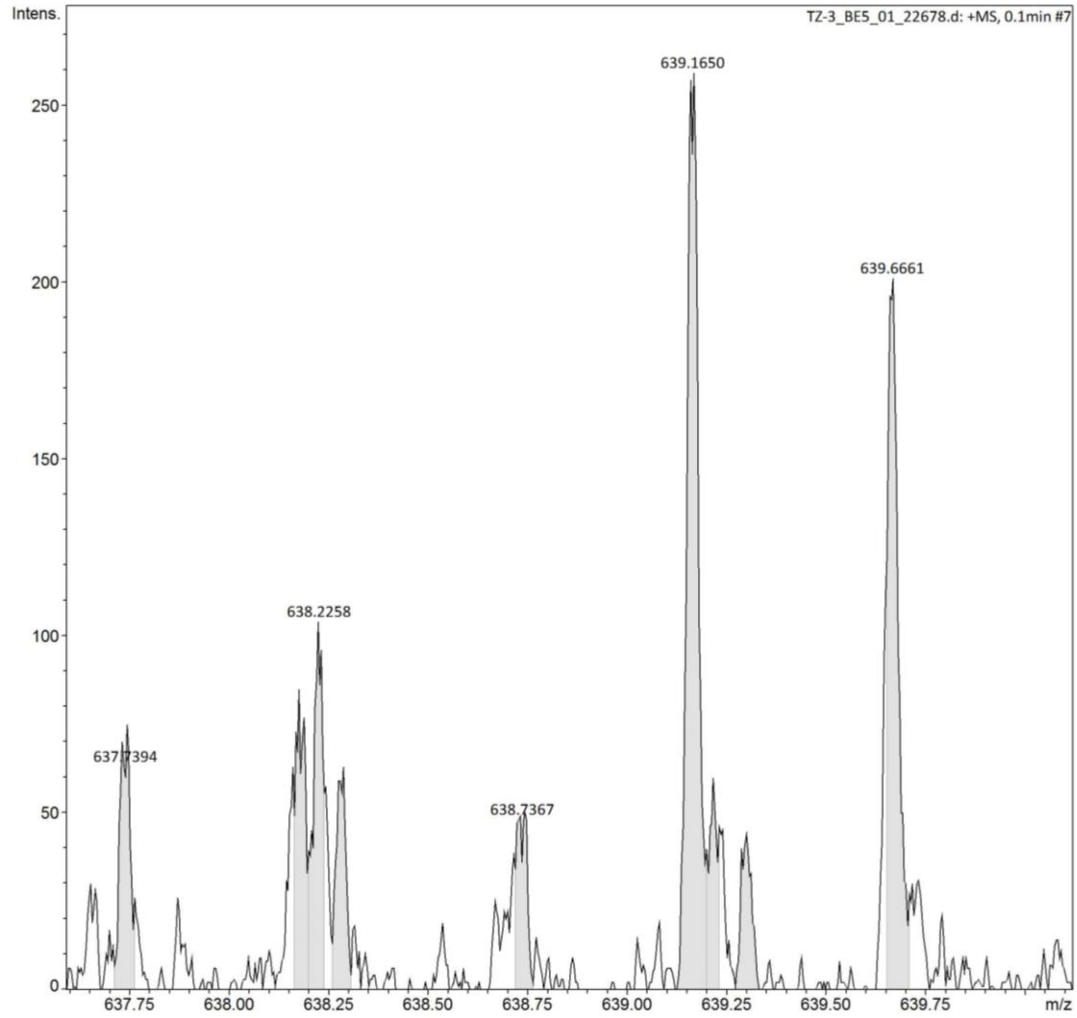


TZ-3_BE5_01_22678.d

Bruker Compass DataAnalysis 4.4 printed: 9/22/2023 4:01:53 PM by: demo Page 1 of 1

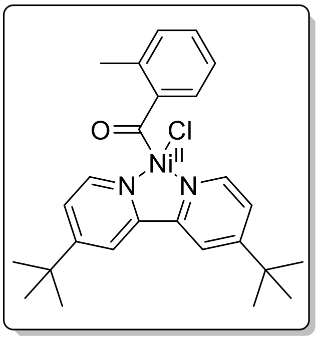


**Ni(dtbpy) (*o*-formal) Cl (10).**[21] Bis(1,5-cyclooctadiene) nickel (55.0 mg, 0.20 mmol) and 4,4'-di-*tert*-butyl-2,2'-bipyridine (53.7 mg, 0.44 mmol) was added to an oven-dried 10 mL round bottom flask equipped with a magnetic stir-bar and purged with $N_2$. THF (3.0 mL) was added via syringe and the reaction mixture was stirred at room temperature for 12 h and then *o*-toluoyl chloride (40 μL, 0.30 mmol) was added. The reaction mixture was stirred for 30 min at room temperature. The obtained compound in 66% yield and data is consistent with the literature. After washing with *n*-hexane 3-5 times and it can be used directly for the next step. Although crystals could be obtained, attempts to characterize this product by X-ray diffraction have suffered from desolvation, even at low temperature. **$^1$H NMR (500 MHz, $CDCl_3$)** *δ* 9.52 (s, 1H), 8.92 (s, 2H), 8.12-8.06 (m, 2H), 7.52 (s, 2H), 7.41-7.34 (m, 3H), 2.72 (s, 3H), 1.31 (s, 9H), 1.28 (s, 9H); **$^{13}$C NMR (125 MHz, $CDCl_3$)** *δ* 216.2, 163.0, 161.8, 155.7, 152.2, 150.6, 149.2, 144.1, 141.2, 132.3, 123.1, 117.0, 116.1, 30.3, 30.1, 24.9, 20.5.

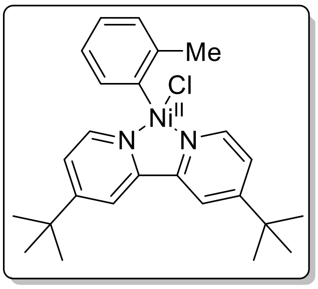


**Ni(dtbpy)(*o*-tolyl)Cl (11).**[20] Bis(1,5-cyclooctadiene) nickel (55.0 mg, 0.20 mmol) and 4,4'-di-*tert*-butyl-2,2'-bipyridine (53.7 mg, 0.44 mmol) was added to an oven-dried 10 mL round bottom flask equipped with a magnetic stir-bar and purged with $N_2$. THF (3.0 mL) was added via syringe and the reaction mixture was stirred at room

temperature for 30 minutes and then 2-chlorotoluene (35 μL, 0.30 mmol) was added. The reaction mixture was stirred for 2 h at room temperature. The obtained compound in 71% yield and data is consistent with the literature. After washing with *n*-hexane 3-5 times and it can be used directly for the next step. **$^{1}$H NMR (500 MHz, $CDCl_3$)** $\delta$ 9.13 (d, *J* = 5.5 Hz, 1H), 7.81 (s, 1H), 7.75 (s, 1H), 7.77 (d, *J* = 6.5 Hz, 1H), 7.61 (d, *J* = 4.5 Hz, 1H), 7.47 (d, *J* = 4.5 Hz, 1H), 7.19 (d, *J* = 6.0 Hz, 1H), 7.09 (d, *J* = 5.0 Hz, 1H), 6.85-6.80 (m, 2H), 3.12 (s, 3H), 1.43 (s, 9H), 1.36 (s, 9H); **$^{13}$C NMR (125 MHz, $CDCl_3$)** $\delta$ 162.9, 161.8, 155.4, 152.0, 150.8, 149.2, 149.0, 141.6, 134.9, 127.0, 123.1, 122.8, 122.6, 122.2, 116.7, 115.9, 34.9, 29.7, 24.7, 17.9.

5.2 Influences of Ligands

**Scheme SI-1** Interconversion of Complexs **9** and **11**

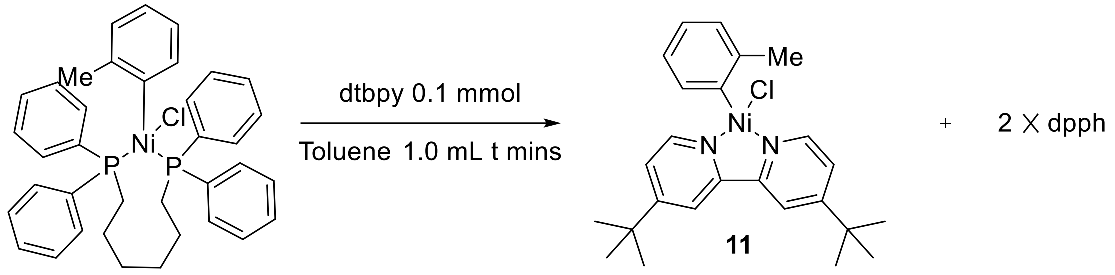


**9**, 0.1 mmol

| | | |
|---|---|---|
| 10 min | 21% | 8% |
| 30 min | 33% | 30% |
| 120 min | 35% | 33% |
| 12 h | 55% | 54% |

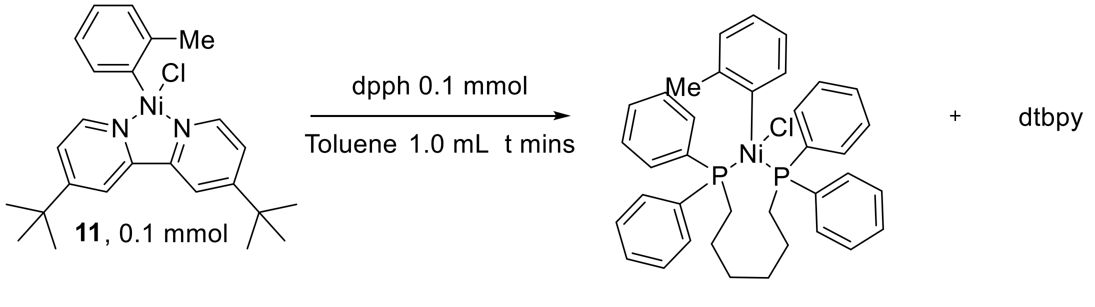


**9**

| | | |
|---|---|---|
| 10 min | 15% | 18% |
| 30 min | 29% | 31% |
| 120 min | 33% | 35% |
| 12 h | 41% | 39% |

To a 10 ml Schlenk tube was added sequentially nickel intermediate **9** (70.8 mg, 0.10 mmol) and 4,4’-di-*tert*-butyl-2,2’-bipyridine (26.80 mg, 0.10 mmol). Then dry toluene (1.00 mL) was added via syringe. The resulting solution was stirred at room temperature under $N_2$. The reaction was then detected at 10 min, 30 min, 2 h or 12 h. The amount of dpph produced by GC-MS detection and nickel intermediate **11** was converted by 4,4’-di-*tert*-butyl-2,2’-bipyridine consumption. Using a similar method with intermediate **11** as a reaction product, the yield of 4,4’-di-*tert*-butyl-2,2’-bipyridine and intermediate **9** can be detected.

**Scheme SI-2** Decarbonlyation of Comples **10** Assisted by P Ligand

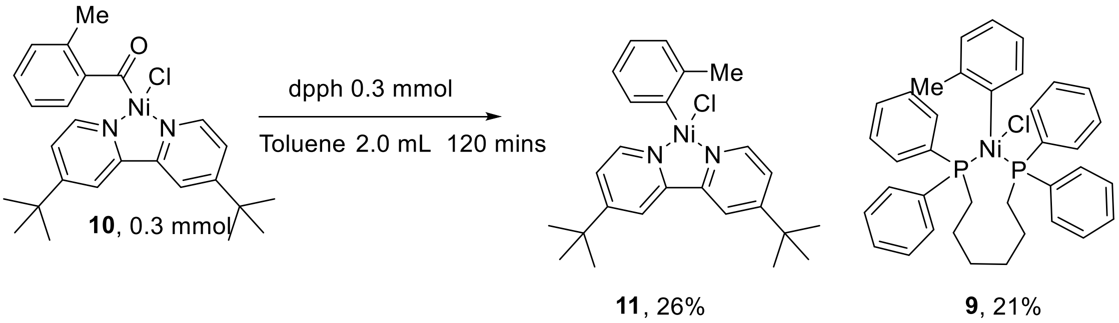


To a 10 ml Schlenk tube was added sequentially nickel intermediate **10** (96.00 mg, 0.2 mmol), dpph (136.2 mg, 0.3 mmol) and toluene (2.0 mL) was added via syringe and the mixture was stirred at room temperature. The resulting solution was stirred for 2 h at room temperature under $N_2$. After washing with *n*-hexane 3-5 times, the yellow crystals formed.

**Scheme SI-3** Effect of ligands on the reaction

**Effect of phosphine ligand on the reaction**

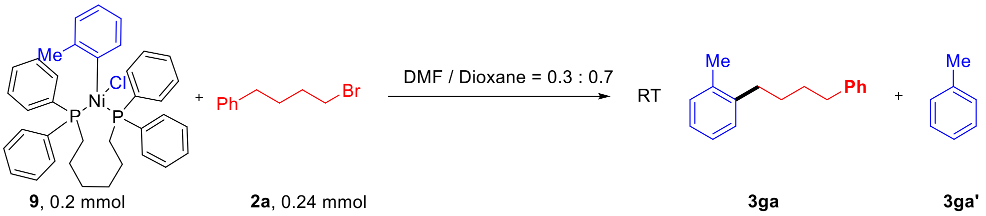


| entry | Variation of optimal conditions | yield of 3ga | yield of 3ga' |
|---|---|---|---|
| 1 | No | 18% | 50% |
| 2 | dtbpy 1.0 eq | 52% | 42% |
| 3 | Mn 3.0 eq | 18% | 80% |
| 4 | dtbpy 1.0 eq, Mn 3.0 eq | 45% | 30% |

**Effect of nitrogen ligand on the reaction**

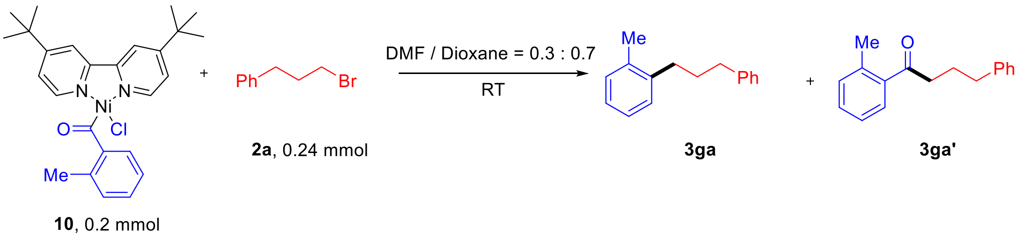


| entry | Variation of optimal conditions | yield of 3ga | yield of 3ga' |
|---|---|---|---|
| 5 | No | 16% | 34% |
| 6 | dpph 1.0 eq | 18% | 28% |
| 7 | Mn 3.0 eq | 21% | 46% |
| 8 | dpph 1.0 eq, Mn 3.0 eq | 22% | 47% |

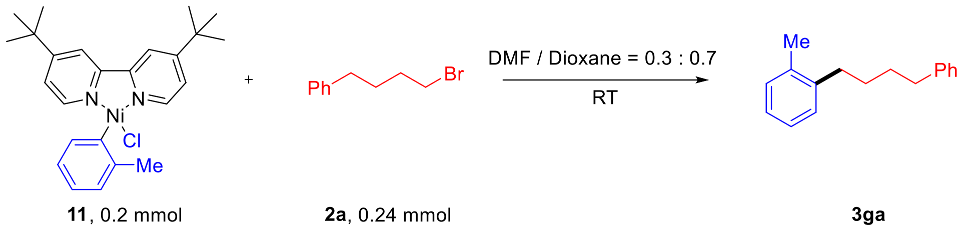


| entry | Variation of optimal conditions | yield of 3ga |
|---|---|---|
| 9 | No | 29% |
| 10 | dpph 1.0 eq | 31% |
| 11 | Mn 3.0 eq | 34% |
| 12 | dpph 1.0 eq, Mn 3.0 eq | 35% |

To a 10 ml Schlenk tube was added sequentially nickel intermediate (0.02 mmol), (4-bromobutyl) benzene (2a) (84.8 mg, 0.40 mmol) and other reagents if needed, then DMF and dioxane (0.30 mL : 0.70 mL) was added via syringe and the mixture was stirred at room temperature. The resulting solution was stirred for 12 h at room temperature under $N_2$. After this time, the crude reaction mixture was diluted with ethyl acetate (10 mL) and washed with water (2.0 mL × 3). The organic layer was dried over $Na_2SO_4$, filtered, and concentrated. The residue was purified by flash chromatography.

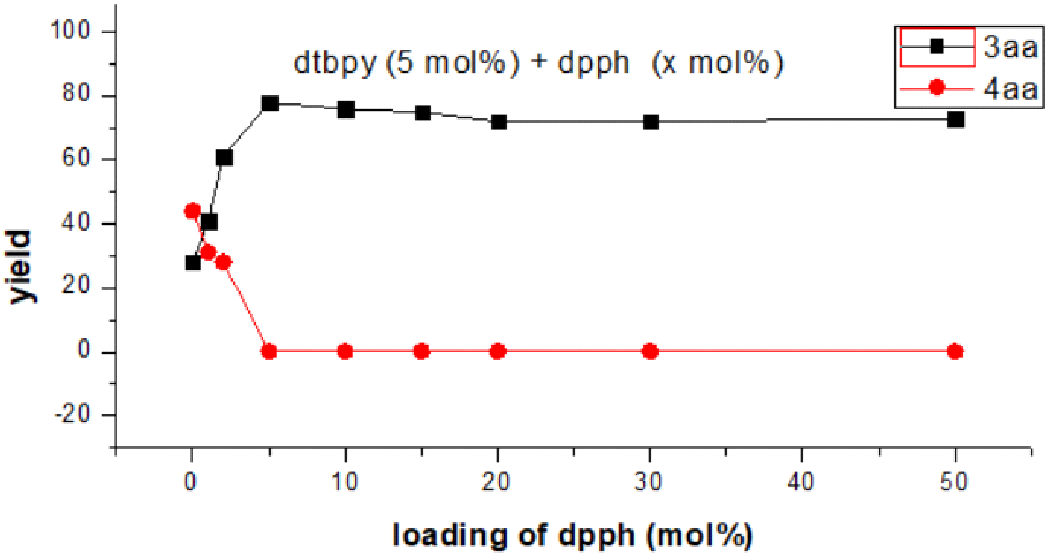


**Figure S1.** The influence of dtbpy under 5 mol% dpph.

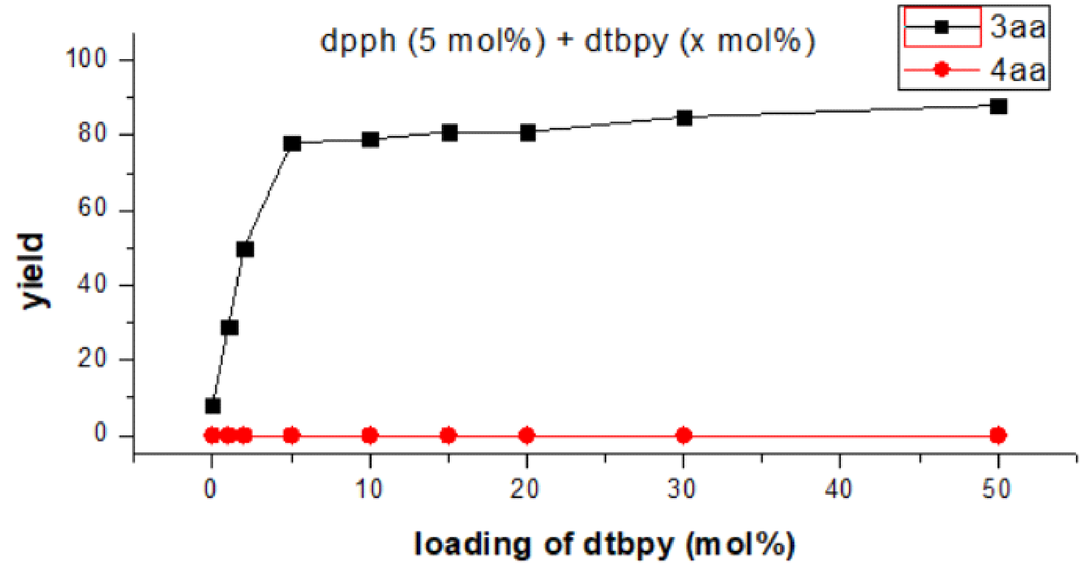


**Figure S2.** The influence of dpph under 5 mol% dtbpy.

**Scheme SI-4** The influence of other ligands

**A: Preparation of Ni complexs**

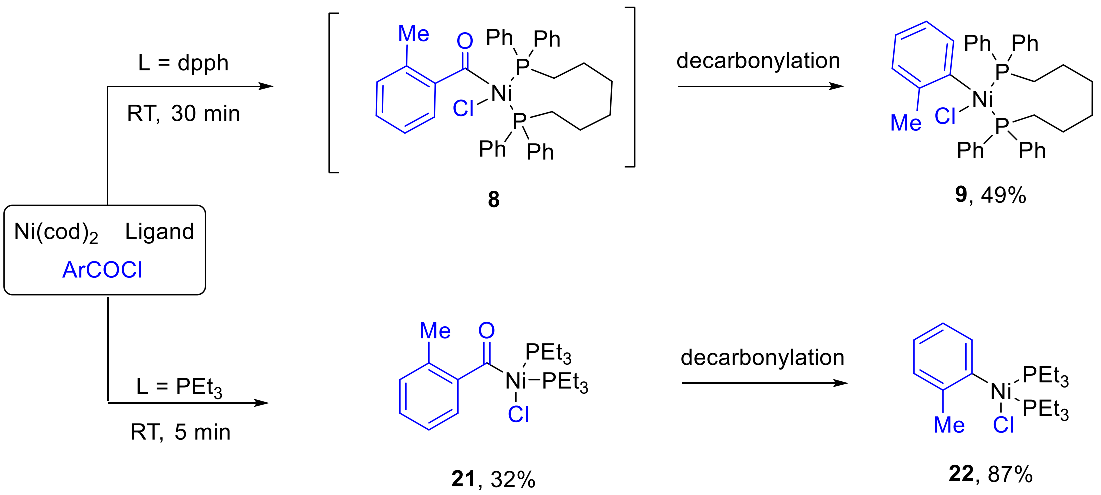


**B: Reactions of complex 8a with 2a**

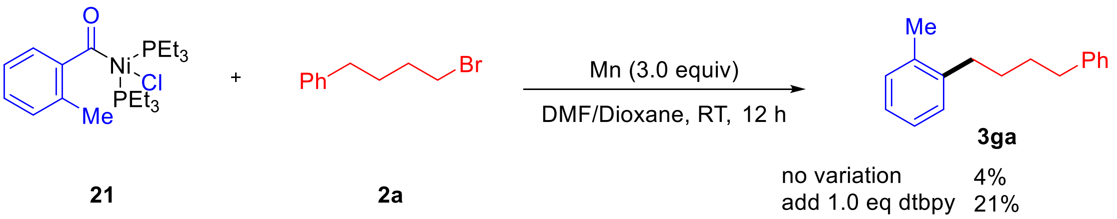


**C: Reactions of complex 8a with 2a**

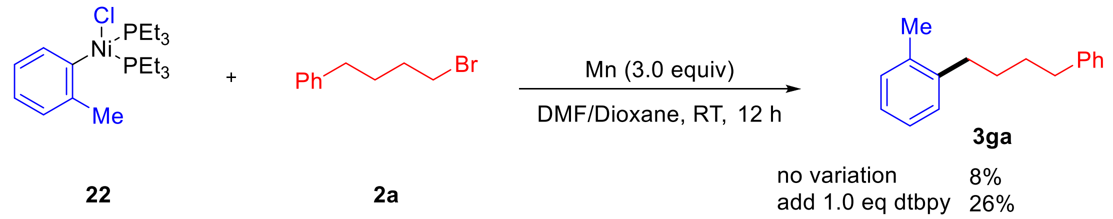


**D: Carbon monoxide capture experiments**

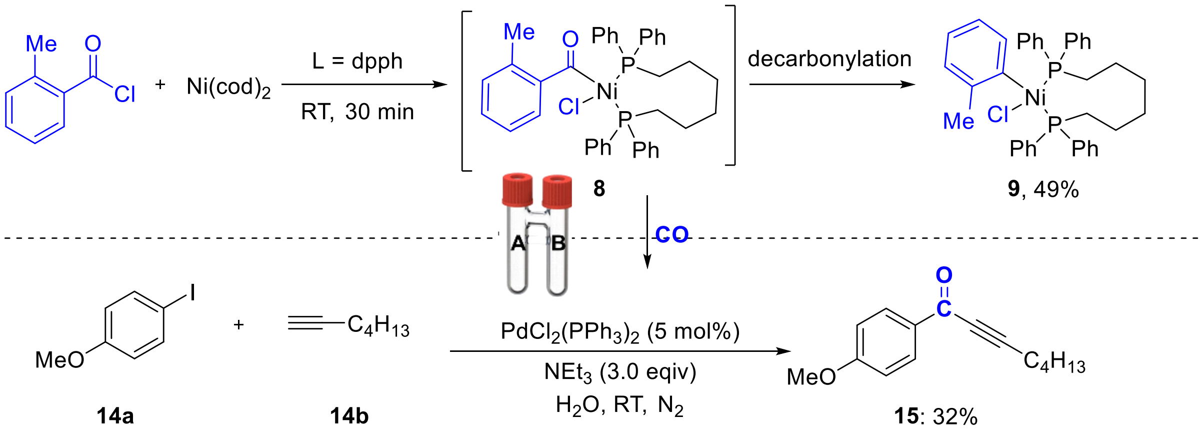

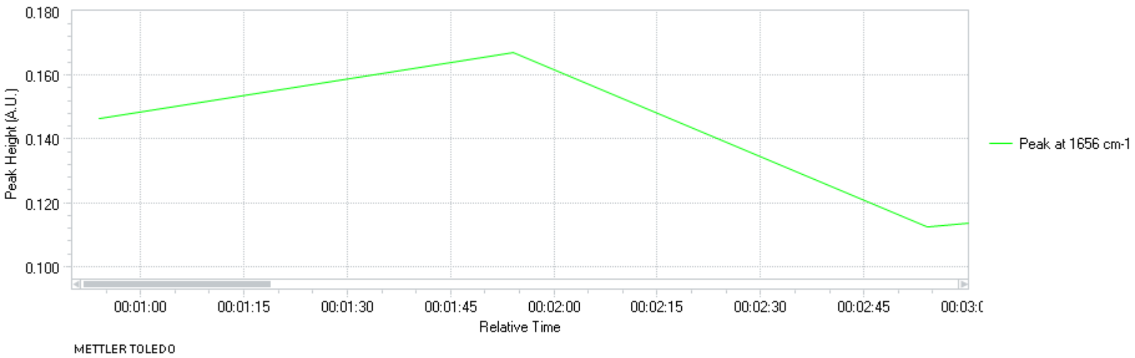


**Figure S3.** In-situ IR data converted from complex **8** to complex **9**.

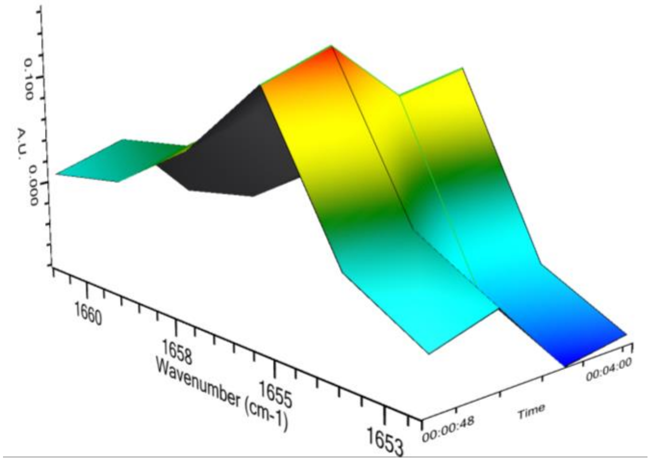


**Figure S4.** In-situ IR plot of converted from complex **8** to complex **9**. Carbonyl signal disappears rapidly in three minutes

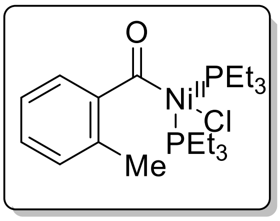


**Ni(PEt$_3$)$_2$ (*o*-formal) Cl** (**21**). Bis(1,5-cyclooctadiene) nickel (55.0 mg, 0.20 mmol) and PEt$_3$ (52.0 mg, 0.44 mmol) was added to an oven-dried 10 mL round bottom flask equipped with a magnetic stir-bar and purged with N$_2$. THF (3.0 mL) was added via syringe and the reaction mixture was stirred at room temperature for 5 minutes and then *o*-Toluoyl chloride (35 μL, 0.30 mmol) was added. The reaction mixture was stirred for 5 min at room temperature. The supernatant was decanted, and the crystals were dried in vacuo to afford complex as a yellow solid (28.6 mg, 32% yield). **$^1$H**

**NMR (500 MHz, $CDCl_3$)** $\delta$ 9.13 (d, $J$ = 5.5 Hz, 1H), 7.81 (s, 1H), 7.75 (s, 1H), 7.77 (d, $J$ = 6.5 Hz, 1H), 7.61 (d, $J$ = 4.5 Hz, 1H), 7.47 (d, $J$ = 4.5 Hz, 1H), 7.19 (d, $J$ = 6.0 Hz, 1H), 7.09 (d, $J$ = 5.0 Hz, 1H), 6.85-6.80 (m, 2H), 3.12 (s, 3H), 1.43 (s, 9H), 1.36 (s, 9H); **$^{13}$C NMR (125 MHz, $CDCl_3$)** $\delta$ 162.9, 161.8, 155.4, 152.0, 150.8, 149.2, 149.0, 141.6, 134.9, 127.0, 123.1, 122.8, 122.6, 122.2, 116.7, 115.9, 34.9, 29.7, 24.7, 17.9. **$^{31}$P NMR (121 MHz, $CDCl_3$)** $\delta$ 7.8, 6.9;

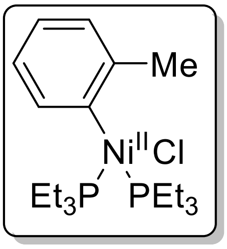


**$Ni(PEt_3)_2$ (*o*-toluoyl) Cl (22)**. $Ni(PEt_3)_2$ (*o*-formal) Cl (22) (44.8 mg, 0.10 mmol) was added to an oven-dried 10 mL round bottom flask equipped with a magnetic stir-bar and purged with $N_2$. THF (3.0 mL) was added via syringe and the reaction mixture was stirred at room temperature for 12 h. The supernatant was decanted, and the crystals were dried in vacuo to afford complex as a yellow solid (36.5 mg, 87% yield). **$^{1}$H NMR (500 MHz, $CDCl_3$)** $\delta$ 6.72-6.67 (m, 3H), 2.80 (s, 3H), 1.32-1.36 (m, 12H), 1.13-1.11 (m, 18H); **$^{13}$C NMR (125 MHz, $CDCl_3$)** $\delta$ 151.3, 140.8, 135.2, 125.9, 123.4, 120.3, 25.9, 12.9, 12.8, 1.3. **$^{31}$P NMR (121 MHz, $CDCl_3$)** $\delta$ 10.1;

5.3 Mechanistic experiments of Decarbonylation.

**Scheme SI-5** Ketones decarbonylated experiment directly

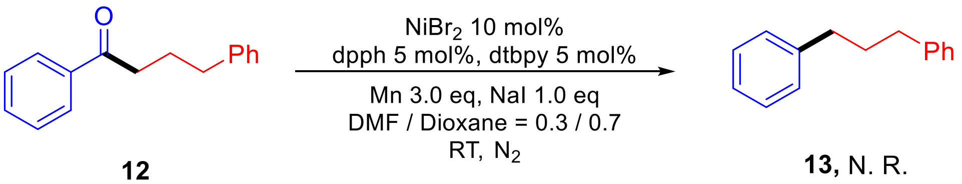


**12** **13,** N. R.

To a 10 ml Schlenk tube was added sequentially $NiBr_2$ (4.37 mg, 0.02 mmol), $(Ph_2P)_2C_4H_8$ (4.25 mg, 0.01 mmol), 4,4'-di-*tert*-butyl-2,2'-bipyridine (2.68 mg, 0.01 mmol), Mn power (32.90 mg, 0.6 mmol) and NaI (14.9 mg, 0.10 mmol). DMF (0.20 mL) was added via syringe and the mixture was stirred at room temperature for 10

min. The Compound 12 (44.82 mg, 0.20 mmol) was added, and then DMF (0.30 mL) was subsequently added via syringe. The resulting solution was stirred for 12 h at room temperature under $N_2$, and this reaction can not be performed.

**Scheme SI-6** Carbon monoxide-capture experiment

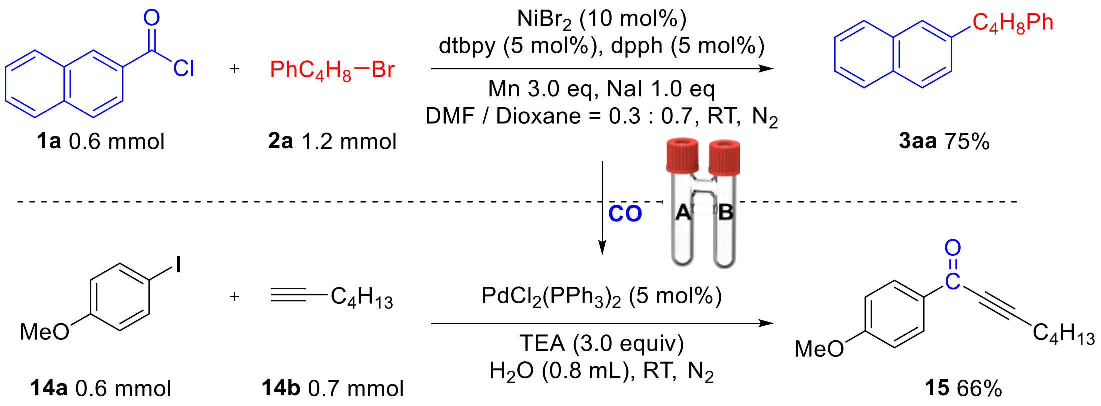


Under an atmosphere of argon, to chamber A of a two-chamber reactor was added $NiBr_2$ (13.11 mg, 0.06 mmol), $(Ph_2P)_2C_4H_8$ (12.75 mg, 0.03 mmol), 4,4'-di-*tert*-butyl-2,2'-bipyridine (8.04 mg, 0.03 mmol), Mn power (98.70 mg, 1.8 mmol) and NaI (44.7 mg, 0.30 mmol). DMF (0.50 mL) was added via syringe and the mixture was stirred at room temperature for 10 min. The 1-naphthoyl chloride (**1a**) (114.00 mg, 0.60 mmol) and (4-bromobutyl) benzene (**2a**) (254.40 mg, 1.20 mmol) was added, and then DMF /Dioxnane (0.4/2.1 mL). According to a literature procedure, to chamber B was added $PdCl_2(PPh_3)_2$ (21.06 mg, 0.03 mmol), 4-iodoanisole (**14a**) (140.42 mg, 0.60 mmol), 1-hexyne (**14b**) (57.50 mg, 0.70 mmol), triethylamine (250 μL, 1.8 mmol) and $H_2O$ (0.8 mL) were subsequently added. The two-chamber reactor was sealed and the reaction was stirred at room temperature for 12 hours. The crude mixture of chamber A and B was transferred to a flask. The volatile materials were evaporated under reduced pressure, and the residue was purified by column chromatography to afford **3aa** in 75%yield and **15** in 66% yield.[23]

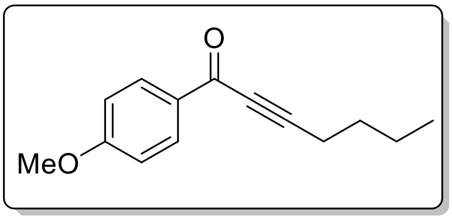


**1-(4-methoxyphenyl)hept-2-yn-1-one** (**15**).[23] **$^1$H NMR (500 MHz, CDCl$_3$)** $\delta$ 7.98 (d, $J$ = 9.0 Hz, 2H), 6.82 (d, $J$ = 9.0 Hz, 2H), 3.75 (s, 3H), 2.37 (t, $J$ = 7.0 Hz, 2H), 1.56-1.50 (m, 2H), 1.42-1.35 (m, 2H), 0.85 (t, $J$ = 7.5 Hz, 3H); **$^{13}$C NMR (125 MHz, CDCl$_3$)** $\delta$ 176.6, 164.0, 131.6, 130.0, 113.5, 95.6, 79.4, 55.3, 29.6, 18.6, 13.3; **MS** (EI) $m/z$ (relative intensity): 216 (M$^+$, 50), 187 (100), 159 (80), 135 (60).

**Radical test experiments**

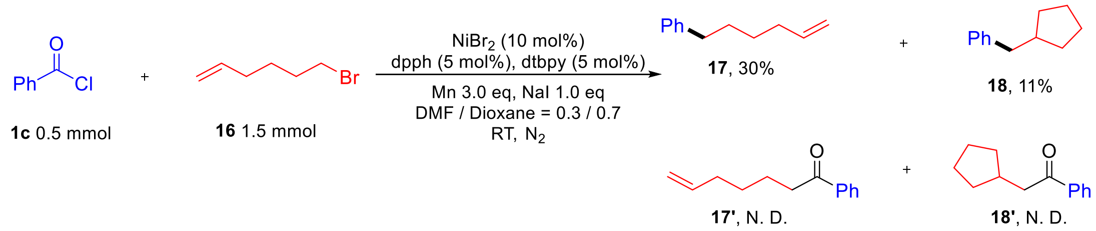


| Entry | Loading of NiBr$_2$ / mol% | yield of 17 /% | yield of 18 /% |
|---|---|---|---|
| 1 | 0 | 0 | 0 |
| 2 | 2 | 16 | 6 |
| 3 | 5 | 30 | 11 |
| 4 | 10 | 42 | 12 |
| 5 | 15 | 52 | 13 |
| 6 | 20 | 68 | 15 |

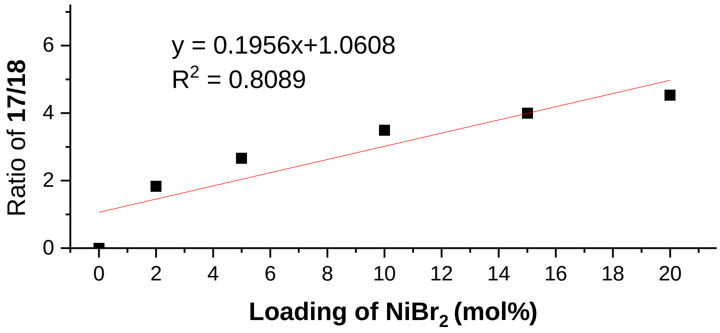


**Figure S5.** Dependence of the ratio of (**17**/**18**) on catalyst loading using a radical clock.

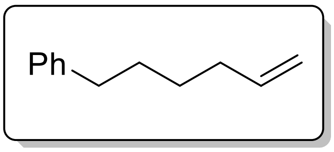


**Hex-5-en-1-ylbenzene** (**17**).[24] The representative procedure was followed using benzoyl chloride (1c) (70.00 mg, 0.50 mmol) and 6-bromo-1-hexene (**16**) (244.5 mg, 1.5 mmol). Isolation by column chromatography (*n*-hexane : EtOAc = 200 : 1) yielded **17** as a colorless oil; **$^{1}$H NMR (500 MHz, CDCl$_3$)** $\delta$ 7.29-7.26 (m, 2H), 7.19-7.17 (m, 3H), 5.83-5.76 (m, 1H), 5.00 (d, *J* = 17.5 Hz, 1H), 4.93 (d, *J* = 10.0 Hz, 1H), 2.61 (t, *J* = 7.5 Hz, 2H), 2.10-2.06 (m, 2H), 1.66-1.61 (m, 2H), 1.47-1.42 (m, 2H); **$^{13}$C NMR (125 MHz, CDCl$_3$)** $\delta$ 142.7, 138.9, 128.4, 128.2, 125.6, 114.4, 35.8, 33.6, 30.9, 28.5; **MS** (EI) *m/z* (relative intensity): 160 (M$^+$, 50), 133 (100), 119 (80), 91 (30).

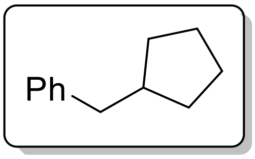


**(Cyclopentylmethyl)benzene** (**18**).[24] The representative procedure was followed using benzoyl chloride (**1c**) (28.00 mg, 0.20 mmol) and 6-bromo-1-hexene (**16**) (244.5 mg, 1.5 mmol). Isolation by column chromatography (*n*-hexane : EtOAc = 200 : 1) yielded **18** as a colorless soild; **$^{1}$H NMR (500 MHz, CDCl$_3$)** $\delta$ 7.26-7.23 (m, 2H), 7.17-7.14 (m, 1H), 7.12-7.08 (m, 2H), 2.37 (t, *J* = 8.0 Hz, 1H), 1.84-1.80 (m, 3H), 1.55-1.40 (m, 8H); **$^{13}$C NMR (125 MHz, CDCl$_3$)** $\delta$ 135.6, 129.4, 128.6, 127.5, 126.8, 125.5, 55.4, 44.6, 31.7, 24.5; **MS** (EI) *m/z* (relative intensity): 160 (M$^+$, 100), 91 (70).

**Scheme SI-7** Radical cyclization experiment

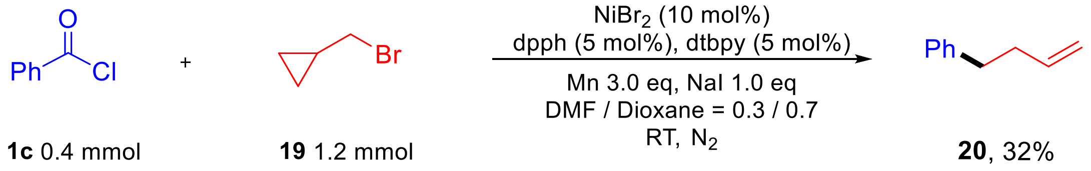

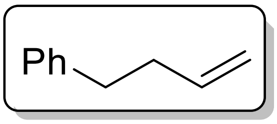

**But-3-en-1-ylbenzene** (**20**).[23] The representative procedure was followed using benzoyl chloride (**1c**) (56.00 mg, 0.40 mmol) and (bromomethyl)cyclopropane (**19**) (162.0 mg, 01.20 mmol). Isolation by column chromatography (*n*-hexane : EtOAc = 200 : 1) yielded **20** (16.89 mg, 32%) as a colorless soild; **$^1$H NMR (500 MHz, CDCl$_3$)** $\delta$ 7.29-7.24 (m, 2H), 7.20-7.14 (m, 3H), 5.89-5.81 (m, 1H), 5.08-5.01 (m, 1H), 5.00-4.95 (m, 1H), 2.74-2.66 (m, 2H), 2.740-2.32 (m, 2H); **$^{13}$C NMR (125 MHz, CDCl$_3$)** $\delta$ 141.8, 138.0, 128.38, 128.37, 128.2, 125.8, 114.9, 35.5, 35.3; **MS** (EI) *m/z* (relative intensity): 132 ($M^+$, 100), 91 (70).

**Kinetic experiments**

**a) Order in NiBr$_2$**

The order in **NiBr$_2$** was determined by obtaining the initial rate of the **3aa** formation at differing amount of $NiBr_2$, $(Ph_2P)_2C_4H_8$ and 4,4'-di-*tert*-butyl-2,2'-bipyridine with a fixed ratio of 10:5:5. The concentration of **NiBr$_2$** was calculated according to the amount of $NiBr_2$.

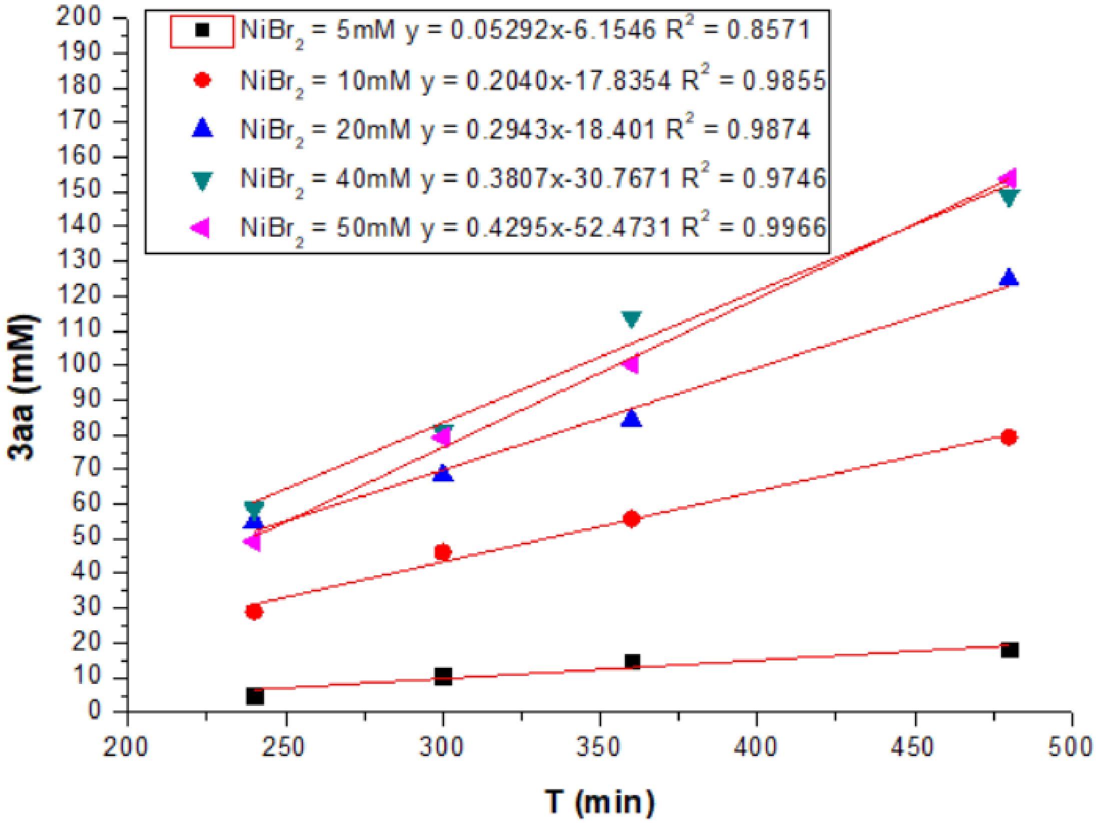


**Figure S6.** Initial rates of **3aa** formation with different concentrations of **$NiBr_2$**

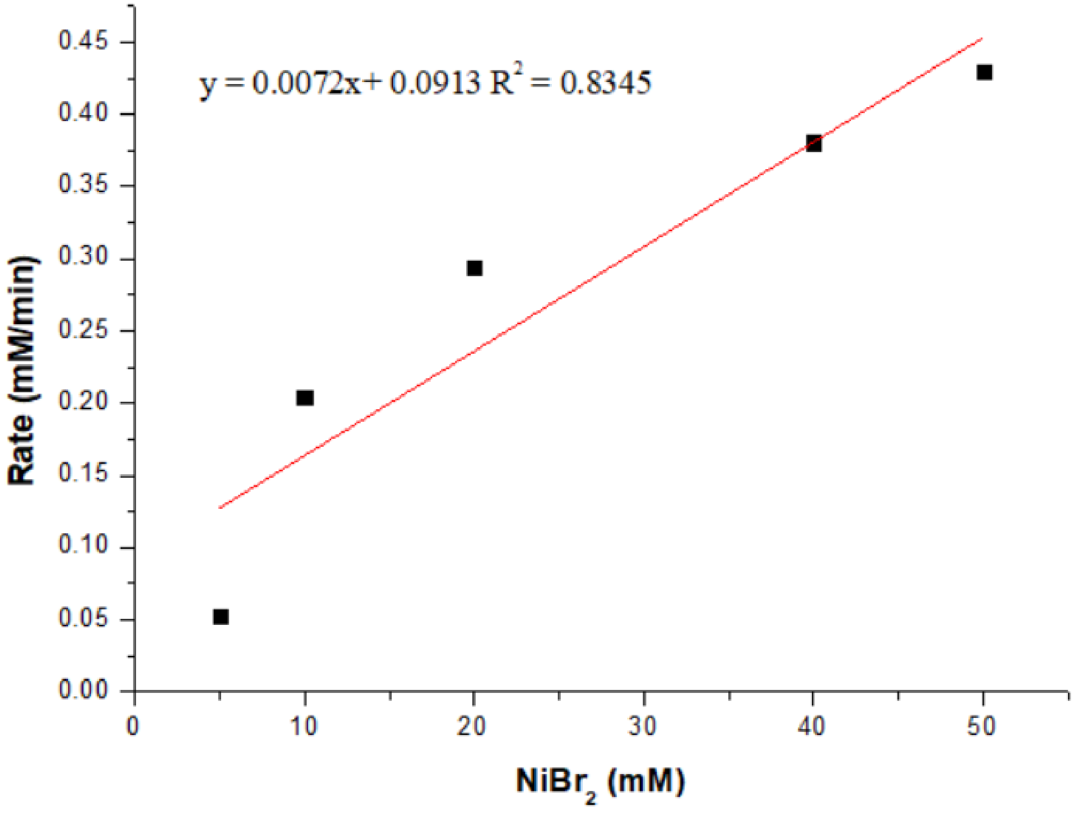


**Figure S7.** Initial rates of **3aa** formation with different concentrations of **$NiBr_2$**

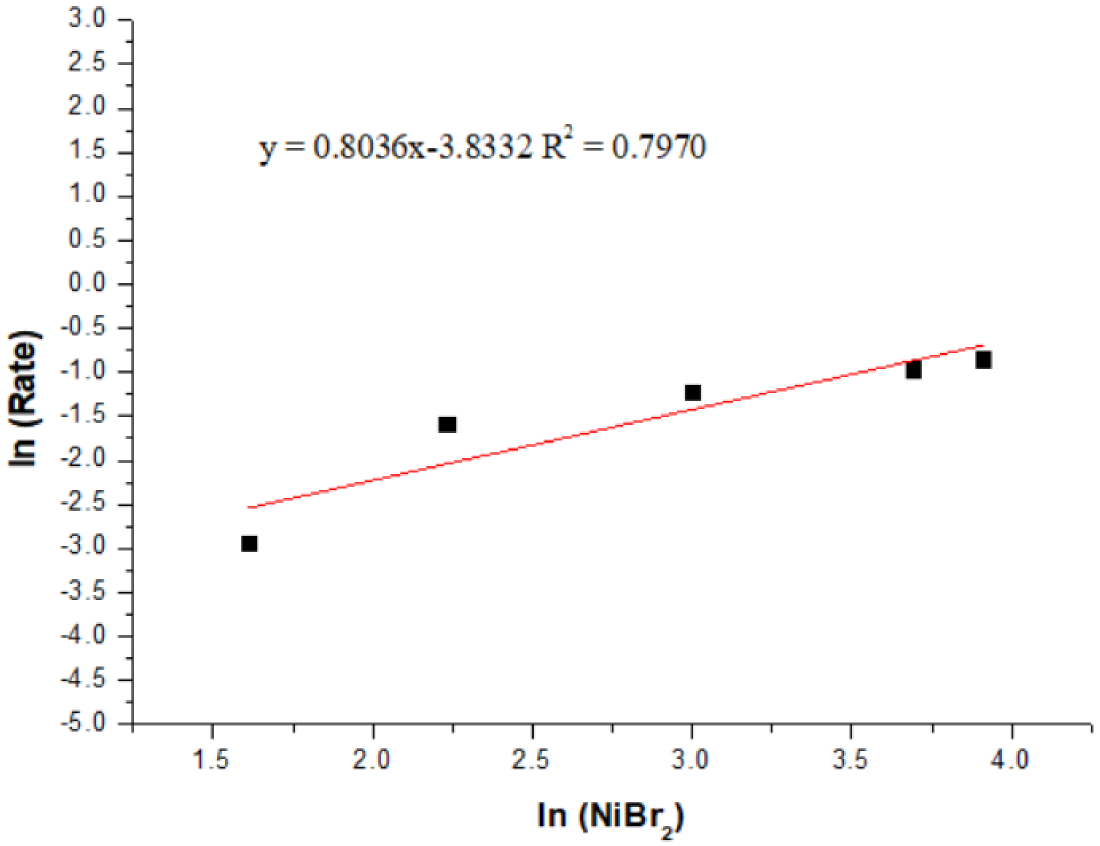


**Figure S8.** Plot of initial rates with varying **1aa**

**b) Order in 1a**

The order in **1a** was determined by obtaining the initial rate of the **3aa** formation at differing amount of **1a**.

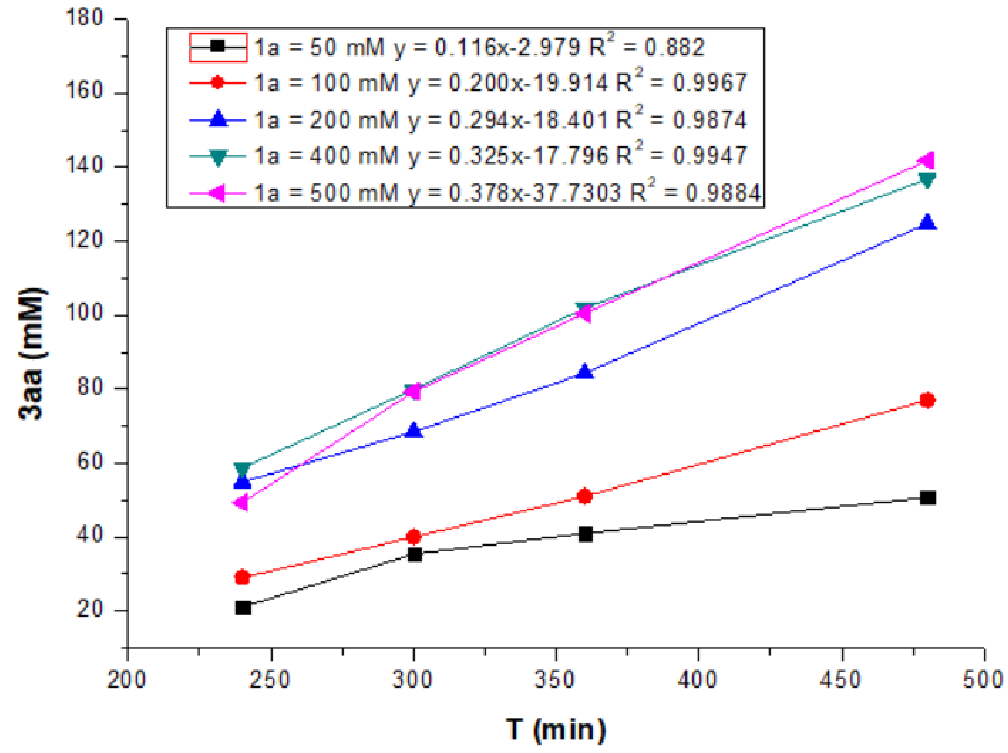


**Figure S9.** Initial rates of **3aa** formation with different concentrations of **1a**

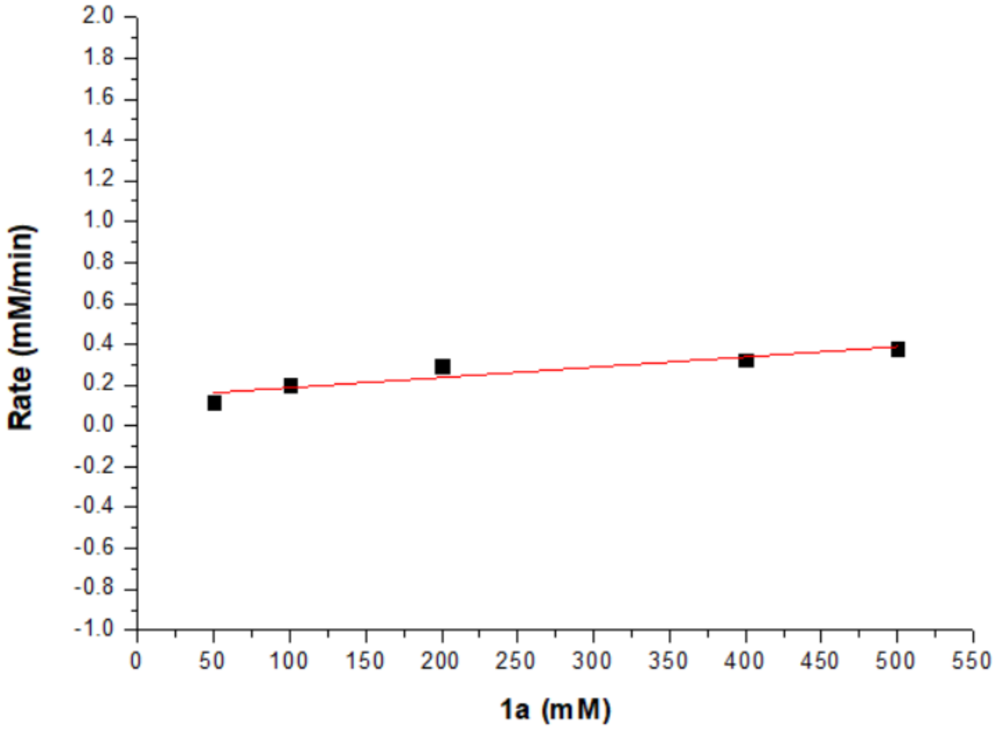


**Figure S10.** Initial rates of **3aa** formation with different concentrations of **1a**

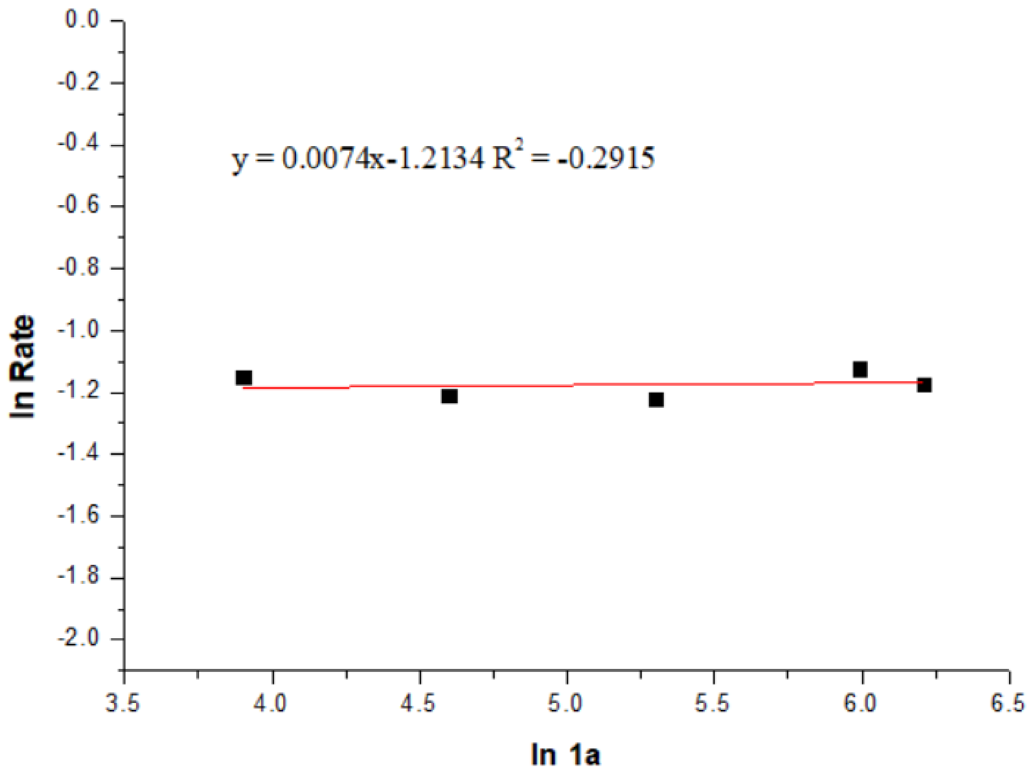


**Figure S11.** Plot of initial rates with varying **1a**

**c) Order in 2a**

The order in **2a** was determined by obtaining the initial rate of the **3aa** formation at differing amount of **2a**.

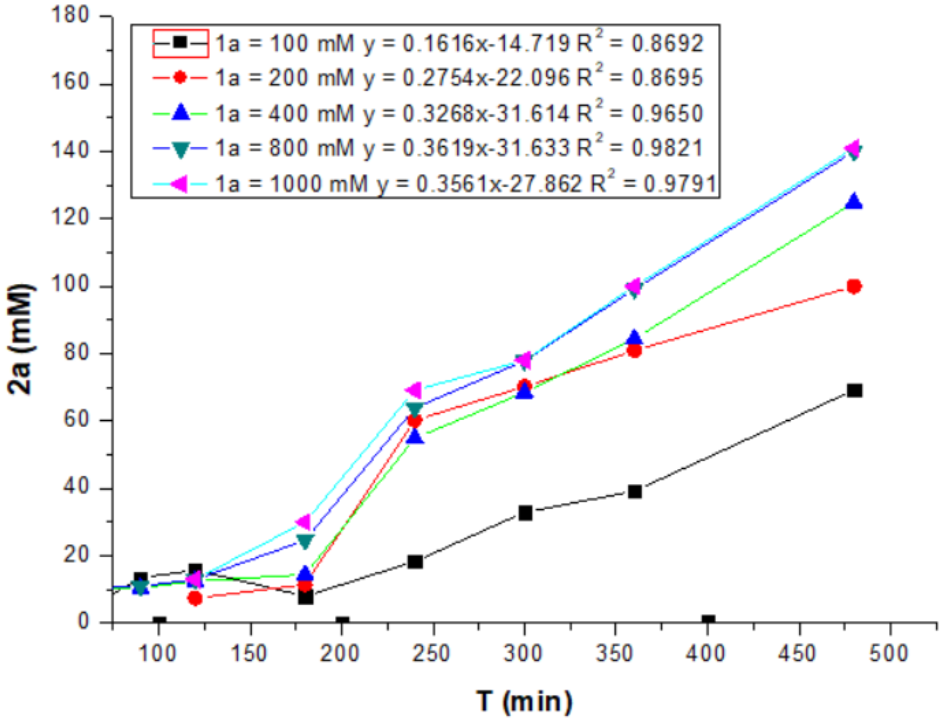


**Figure S12.** Initial rates of **3aa** formation with different concentrations of **2a**

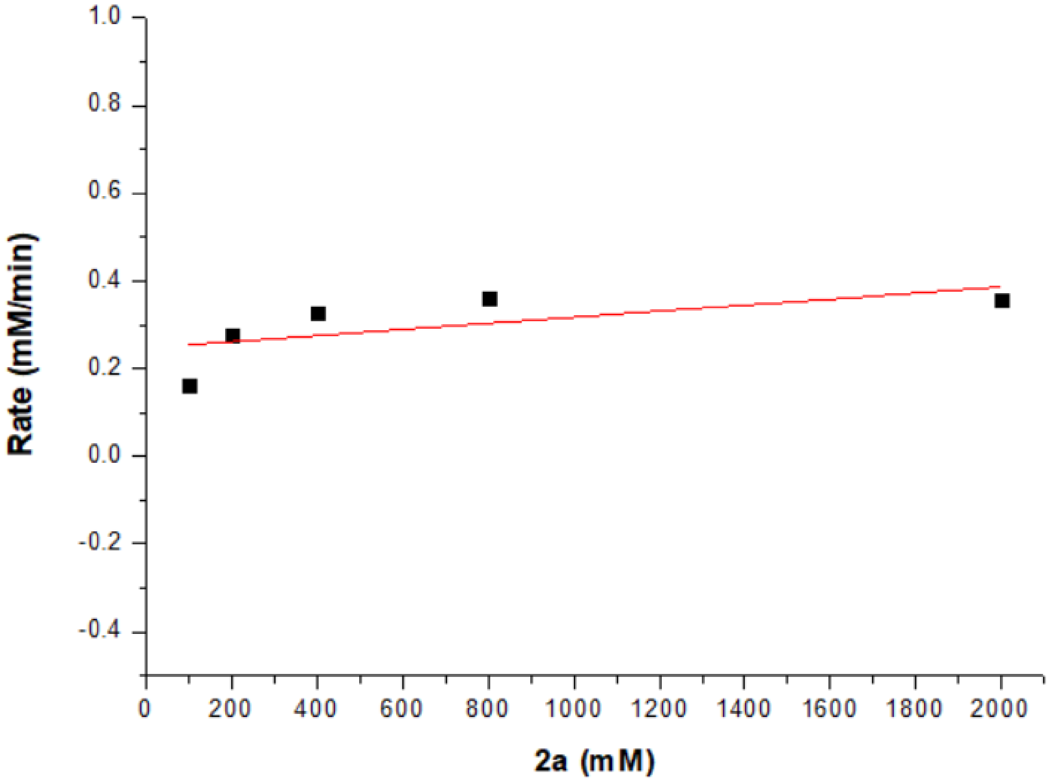


**Figure S13.** Initial rates of **3aa** formation with different concentrations of **3a**

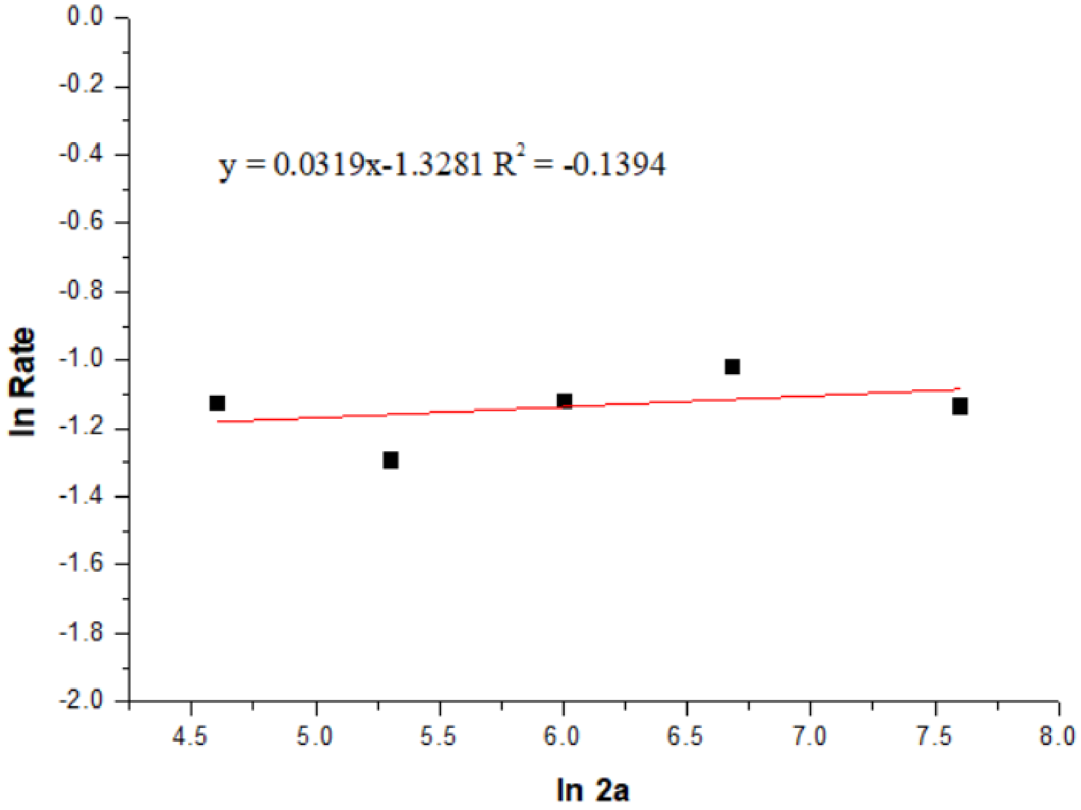


**Figure S14.** Plot of initial rates with varying **2a**

## d) Hammett plot

The Hammett plot was determined by obtaining the initial rate of the formation of corresponding **3** with different substrate **1**.

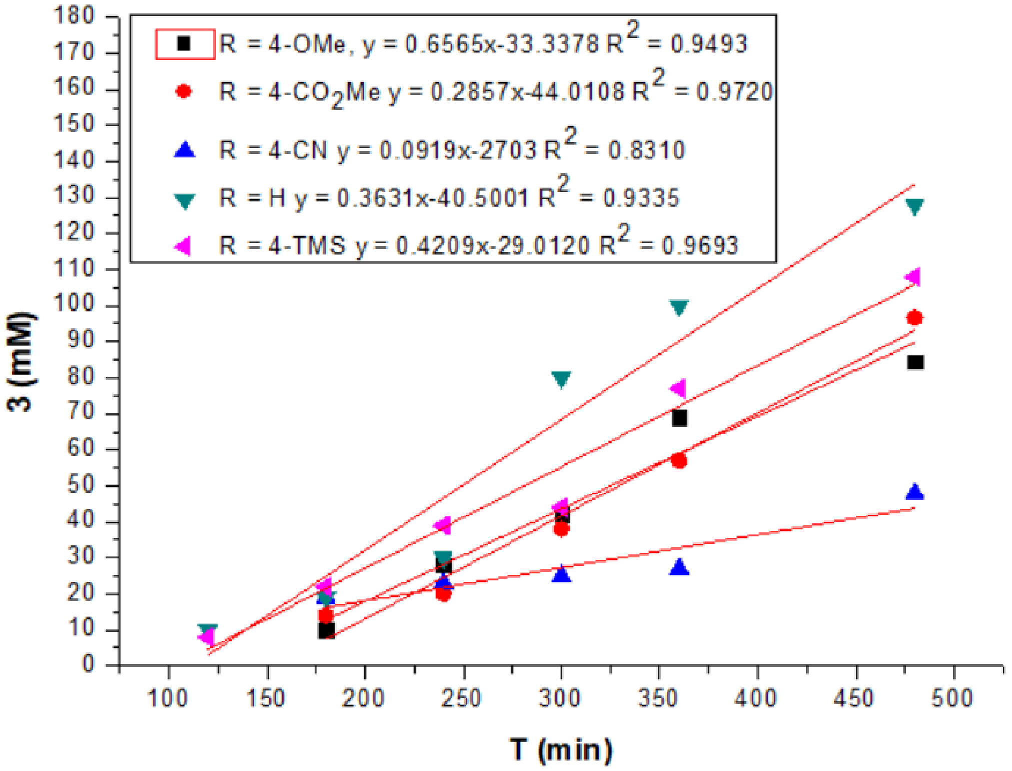


**Figure S15.** Plot of initial rates with varying **1**

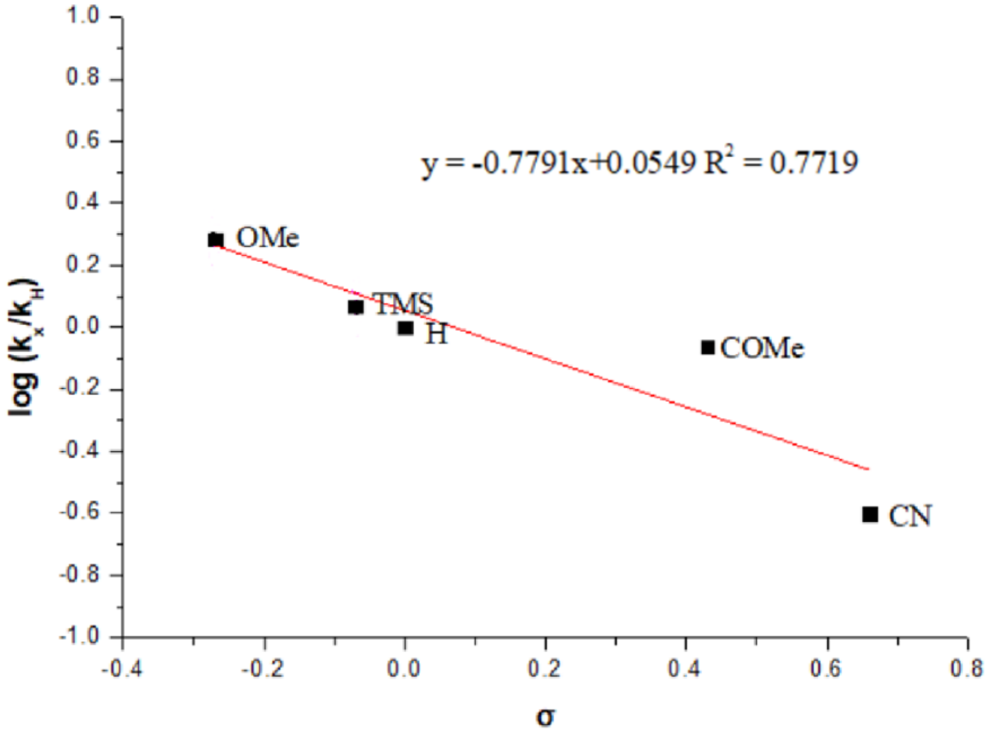


**Figure S16.** Hammett plot of **1**.

**e) Reaction profile**

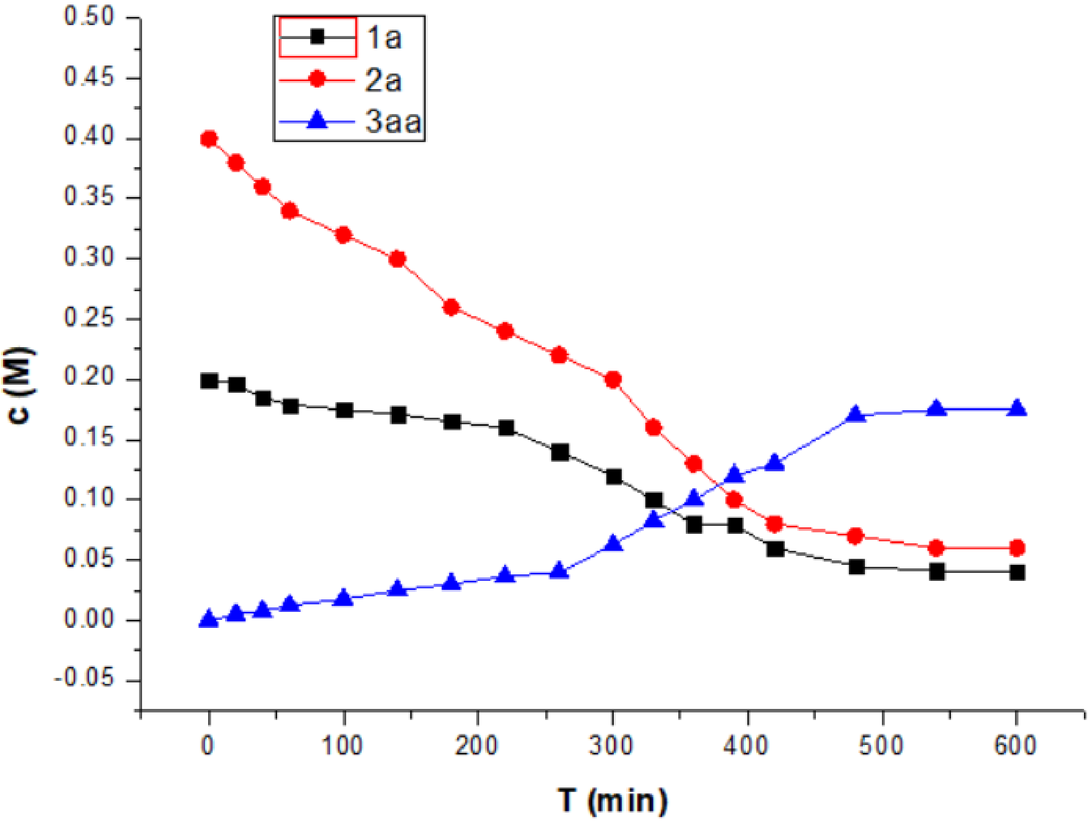


**Figure S17.** Initial rates of **1aa**, **2aa** or **3aa** formation with different concentrations of time.

## 6. References


1) Jiang, B.; Shi, S.-L. *Chin. J. Chem*. **2022**, *40*, 1813–1820.

2) Dubbaka, R.; Kienle, M.; Mayr, H.; Knochel, P. *Angew. Chem., Int. Ed.* **2007**, *46*, 9093–9096.

3) Czaplik, W. M.; Mayer, M.; von Wangelin, A. J. *Angew. Chem., Int. Ed.* **2009**, *48*, 607–610.

4) Kondolff, I.; Doucet, H.; Santelli, M. *Eur. J. Org. Chem*. **2006**, 765–774.

5) Zhang, W.; Wang, F.; McCann, S. D.; Wang, D.; Chen, P.; Stahl, S. S.; Liu, G. *Science* **2016**, *353*, 1014–1018.

6) Cui, X.; Li, Y.; Topf, C.; Junge, K.; Beller, M. *Angew. Chem., Int. Ed*. **2015**, *54*, 10596–10599.

7) Betori, R. C.; May, C. M.; Scheidt, K. A. *Angew. Chem., Int. Ed*. **2019**, *58*, 16490–16494.

8) Shen, Z.-L.; Goh, K. K.; Wong, C. H.; Yang, Y.-S.; Lai, Y.-C.; Cheong, H.-L.; Loh, T.-P. *Chem. Commun.* **2011**, *47*, 4778–4780.

9) Wu, M.; Han, Z.; Li, K.; Wu, J.; Ding, K.; Lu, Y. *J. Am. Chem. Soc*. **2019**, *141*, 16362–16373.

10) Petzold, D.; Singh, P.; Almqvist, F.; Koenig, B. *Angew. Chem., Int. Ed.* **2019**, *58*, 8577–8580.

11) Wei, X.-J.; Abdiaj, I.; Sambiagio, C.; Li, C.; Zysman-Colman, E.; Alcazar, J.; Noel, T. *Angew. Chem., Int. Ed*. **2019**, *58*, 13030–13034.

12) He, Z.; Song, F.; Sun, H.; Huang, Y. *J. Am. Chem. Soc*. **2018**, *140*, 2693–2699.

13) Yamada, T.; Saito, K.; Akiyama, T. *Adv. Synth. Catal*. **2016**, *358*, 62–66.

14) Askey, H. E.; Grayson, J. D.; Tibbetts, J. D.; Turner-Dore, J. C.; Holmes, J. M.; Kociok-Kohn, G.; Wrigley, G.L.; Cresswell, A. J. *J. Am. Chem. Soc*. **2021**, *143*, 15936–15945.

15) Rasina, D.; Otikovs, M.; Leitans, J.; Recacha, R.; Borysov, O. V.; Kanepe-Lapsa, I.; Domraceva, I.; Pantelejevs, T.; Tars, K.; Blackman, M. J.; Jaudzems, K.; Jirgensons, A. *J. Med. Chem.* **2016**, *59*, 374–387.

16) Liu, T.; Yang, Y.; Wang, C. *Angew. Chem., Int. Ed.* **2020**, *59*, 14256–14260.

17) Bronstein, H. A.; Luscombe, C. K. *J. Am. Chem. Soc.* **2009**, *131*, 12894–12895.

18) Feng, B.; Zhang, G.; Feng, X.; Chen, Y. *Org. Chem. Front.* **2022**, *9*, 1085–1089.

19) Maciejewski, H.; Sydor, A.; Marciniec, B.; Kubicki, M.; Hitch-cock, P. B. *Inorg. Chim. Acta*. **2006**, *359*, 2989–2997.

20) Biswas, S.; Weix, D. J. Mechanism and Selectivity in Nickel-Catalyzed Cross-Electrophile

Coupling of Aryl Halides with Alkyl Halides. *J. Am. Chem. Soc.* **2013**, *135*, 16192–16197.

21) Zhou, M.; Zhao, H.-Y.; Zhang, S.; Zhang, Y.; Zhang, X. Nickel-Catalyzed Four-Component Carbocarbonylation of Alkenes under 1 atm of CO. *J. Am. Chem. Soc*. **2020**, *142*, 18191–18199.

22) Zhuo, J.; Zhang, Y.; Li, Z.; Li, C. *ACS Catal*. **2020**, *10*, 3895–3903.

23) Liu, X.; Liu, B.; Liu, Q. *Angew. Chem., Int. Ed.* **2020**, *59*, 6750–6755.

24) Zhu, D.; Lv, L.; Qiu, Z.; Li, C.-J. *J. Org. Chem.* **2019**, *84*, 6312–6322.

# 7. NMR Spectrum

**1-(4-Phenylbutyl) naphthalene (3aa)**

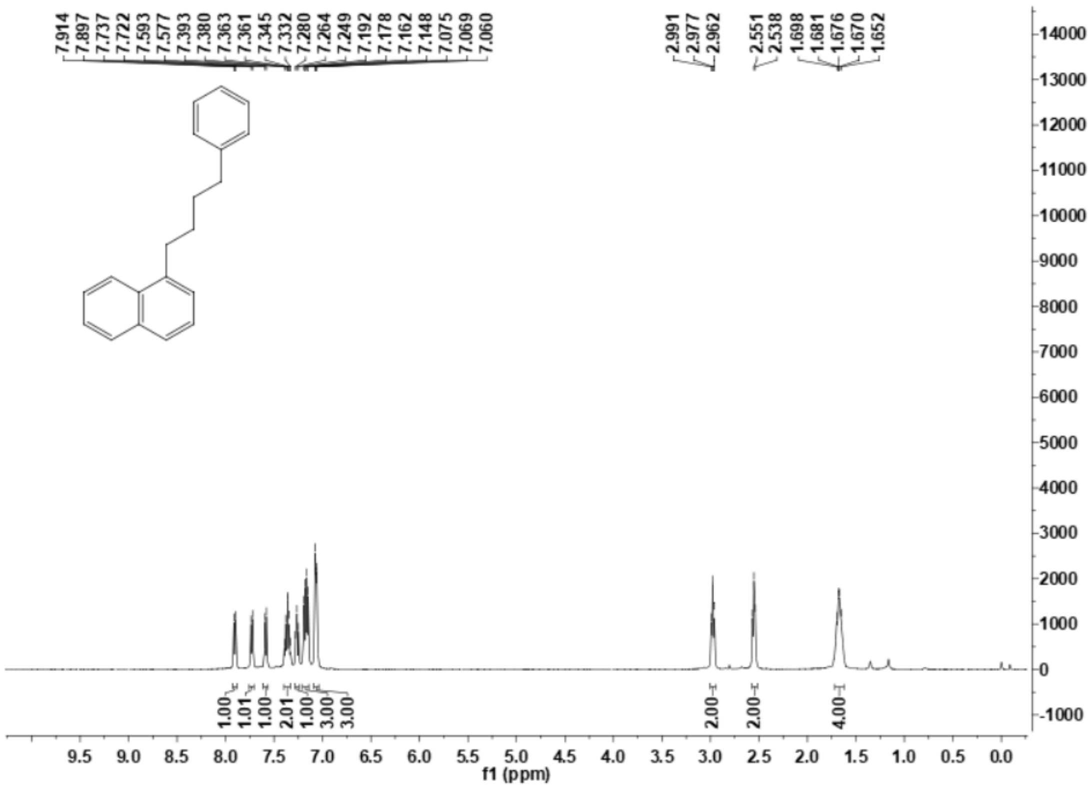


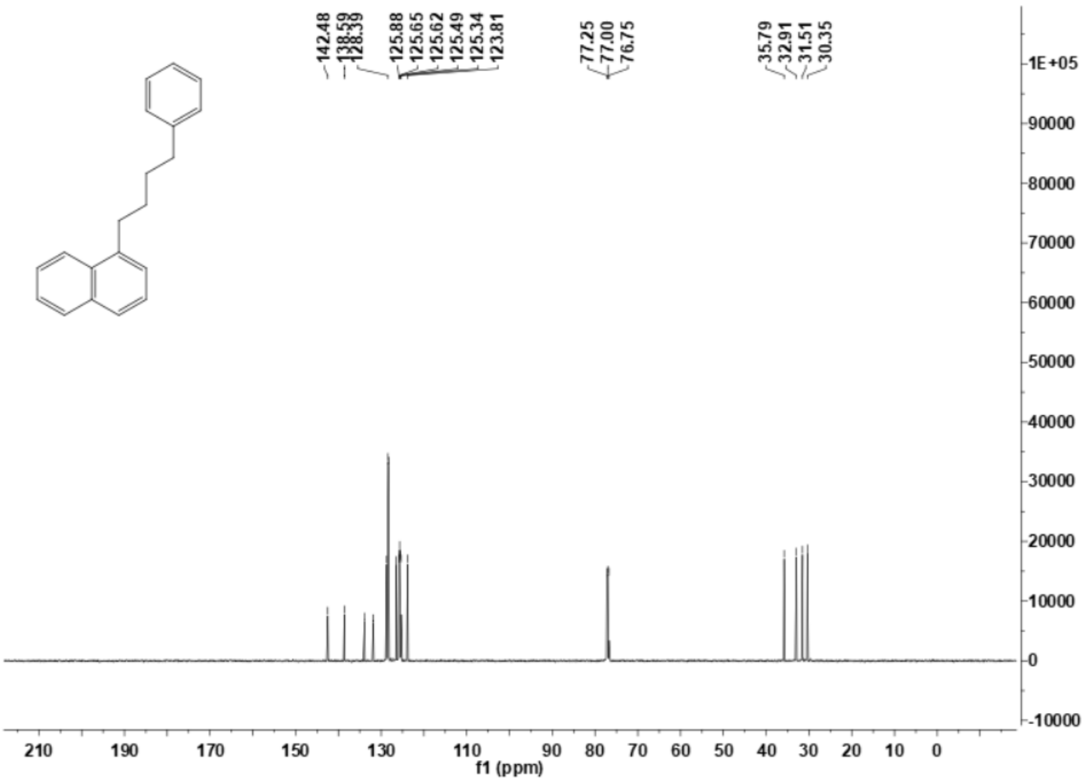

## 1-Methylnaphthalene (3ab)

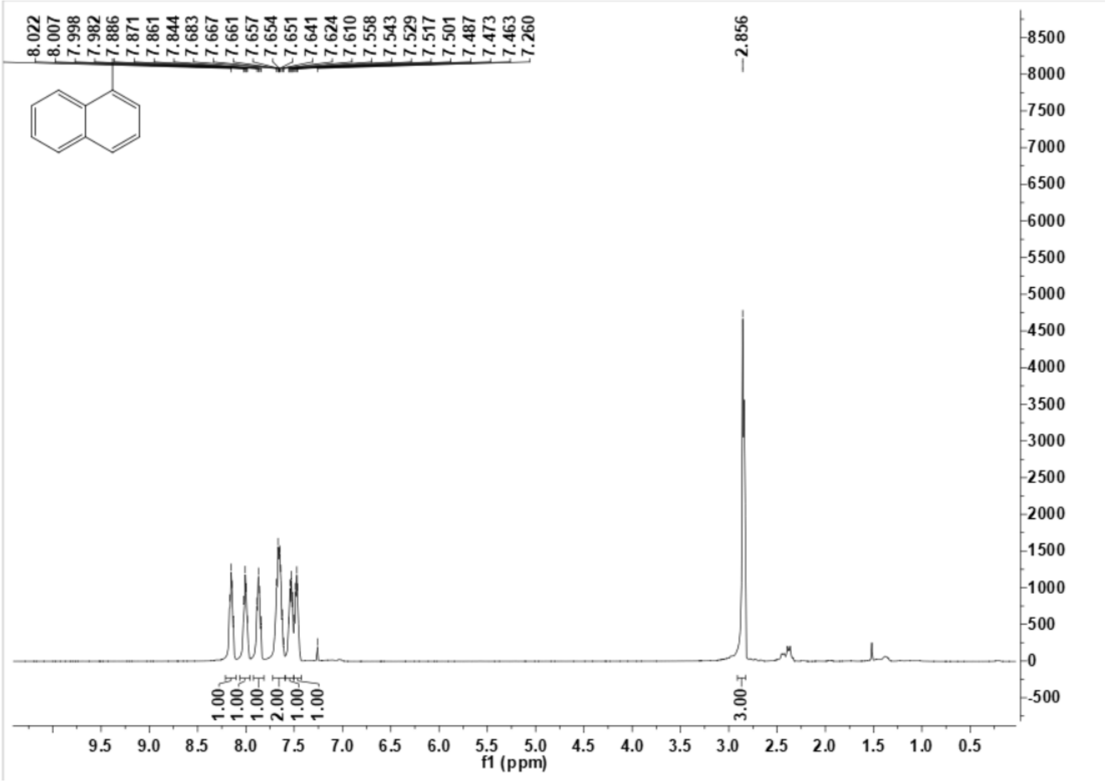


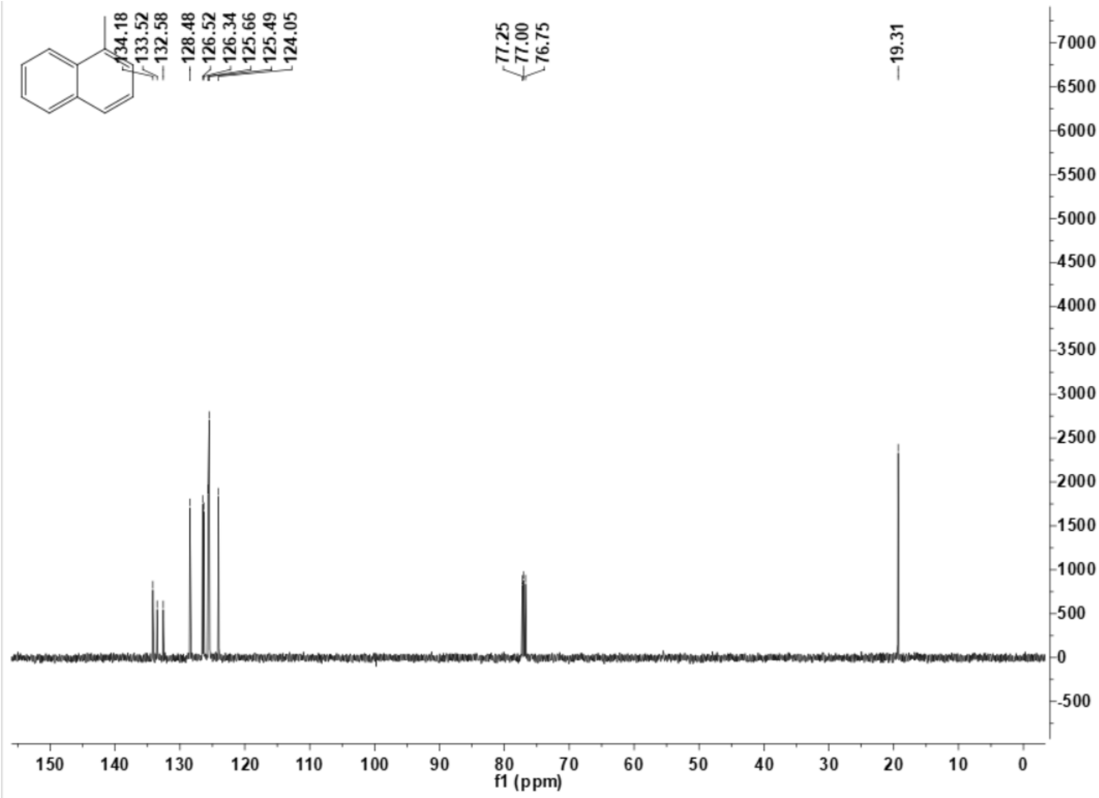

**1-Ethylnaphthalene (3ac)**

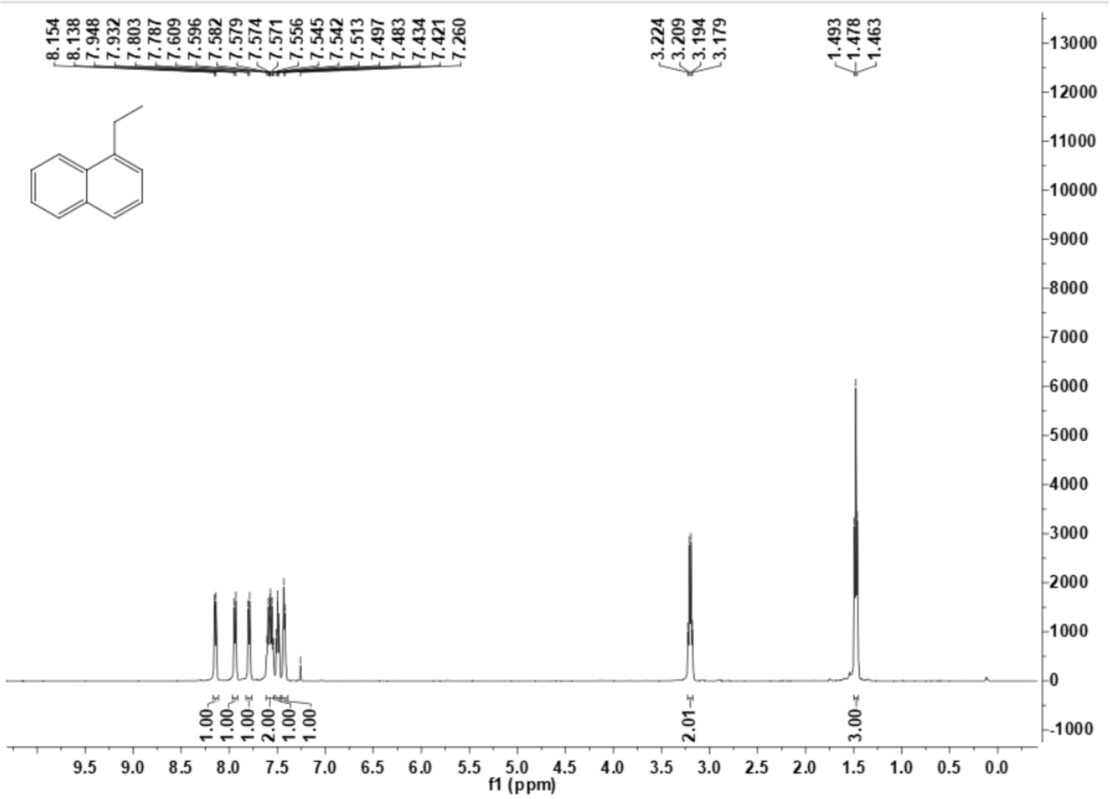

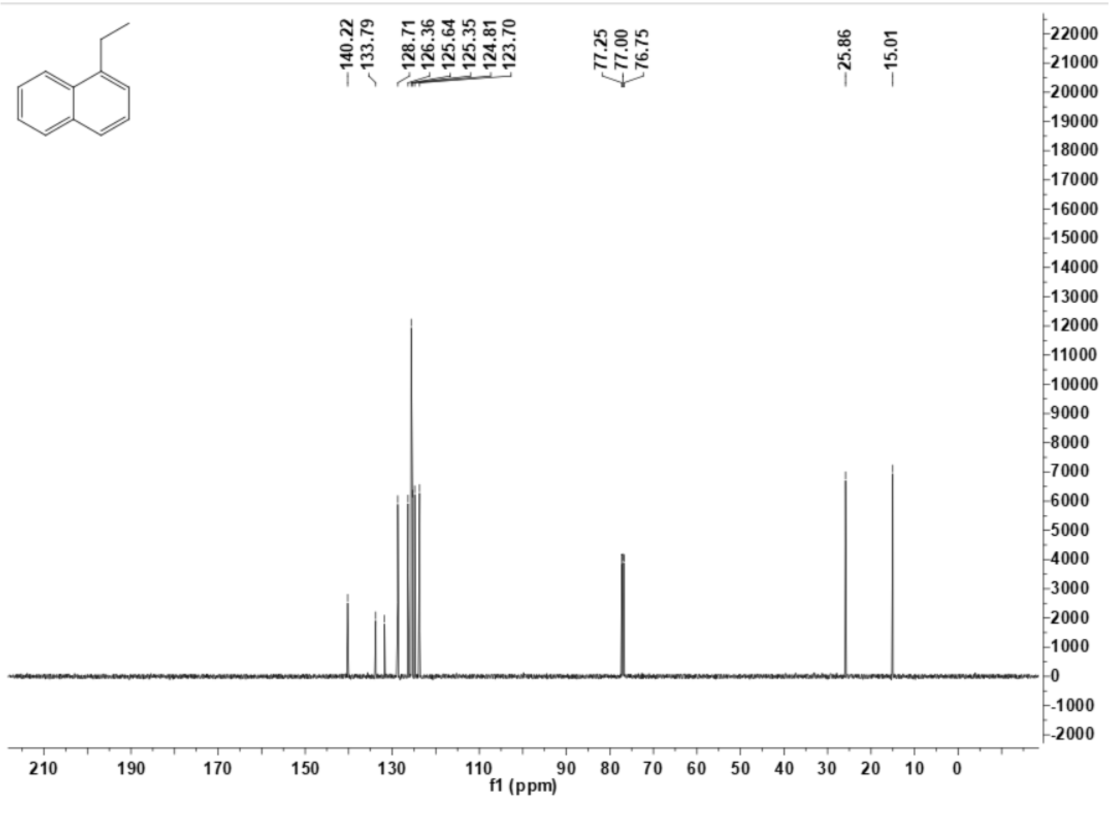

# 1-Phenethylnaphthalene (3ad)

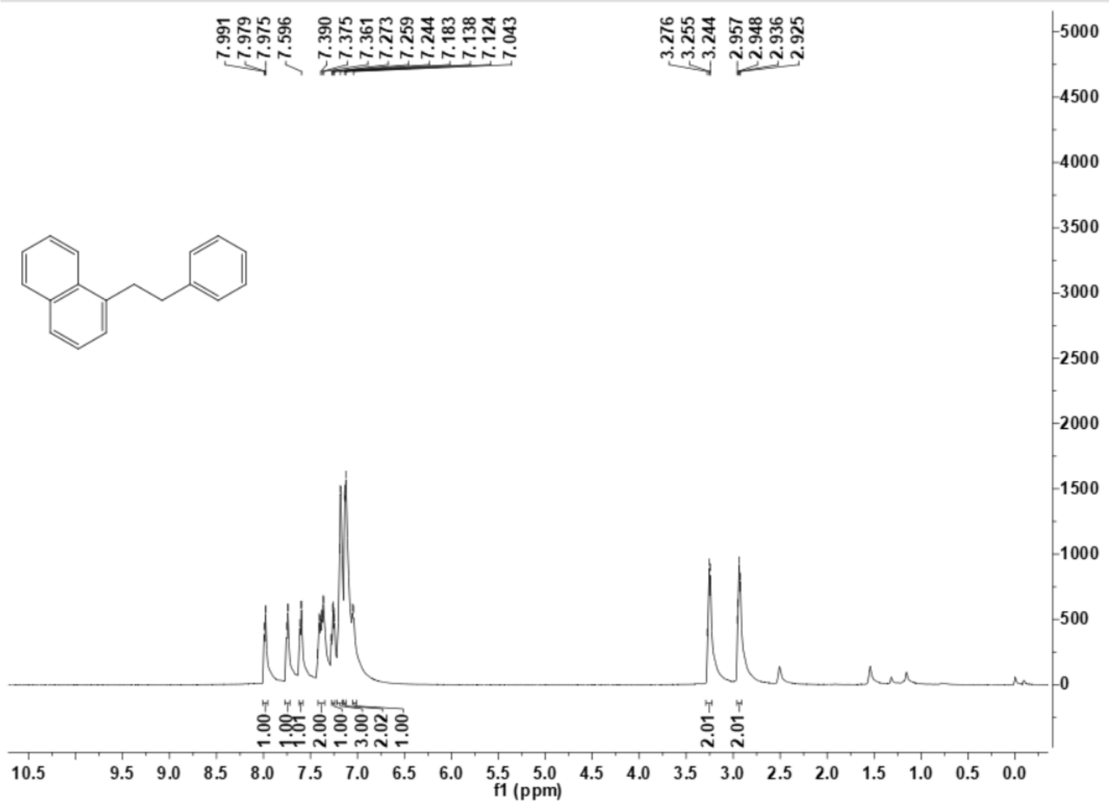


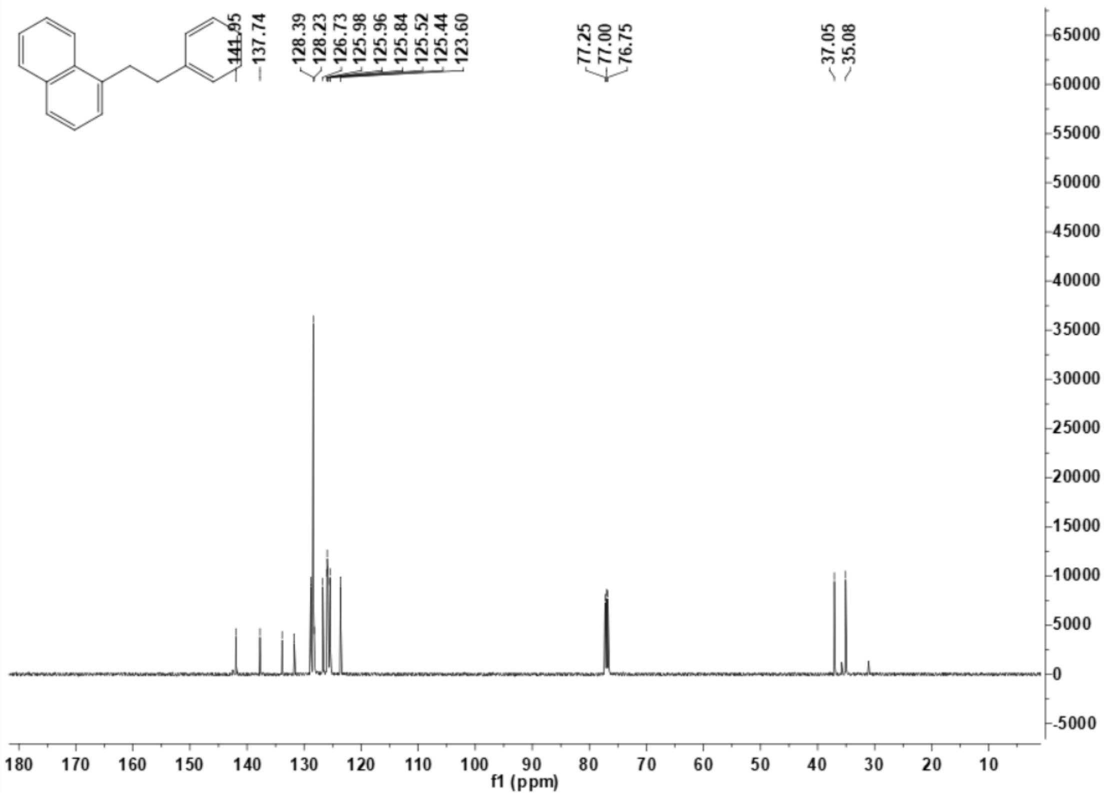

# 1-Hexylnaphthalene (3ae)

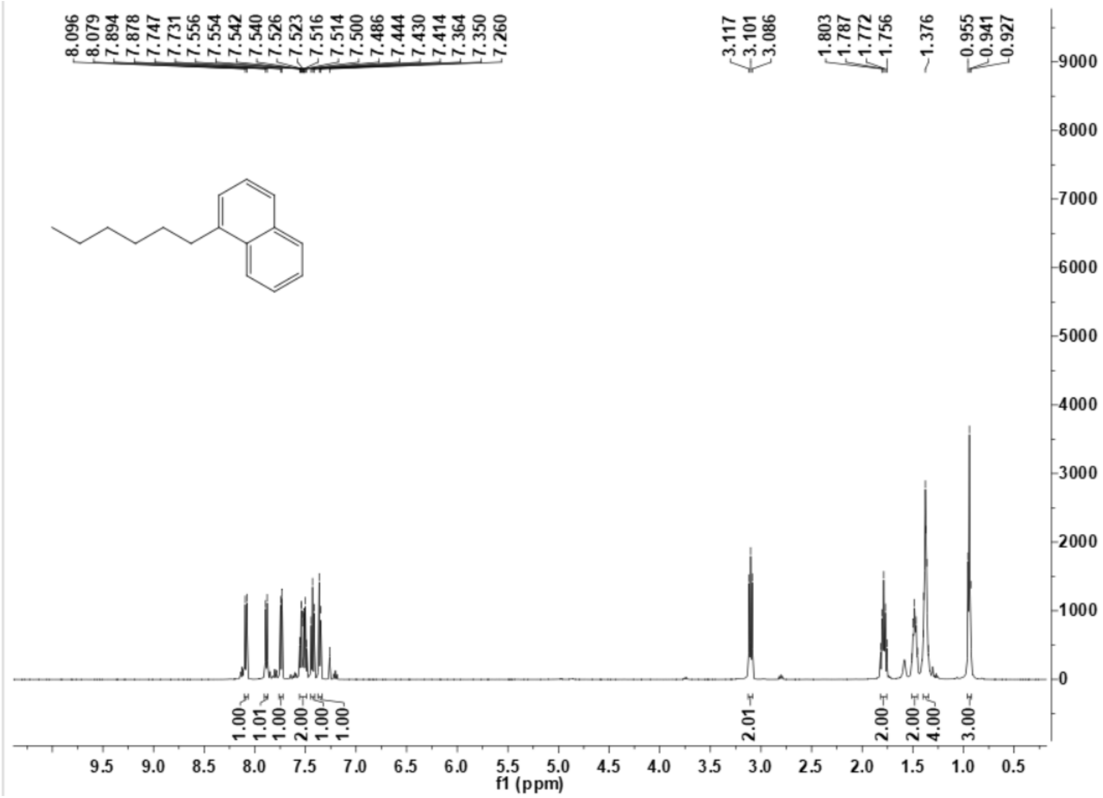

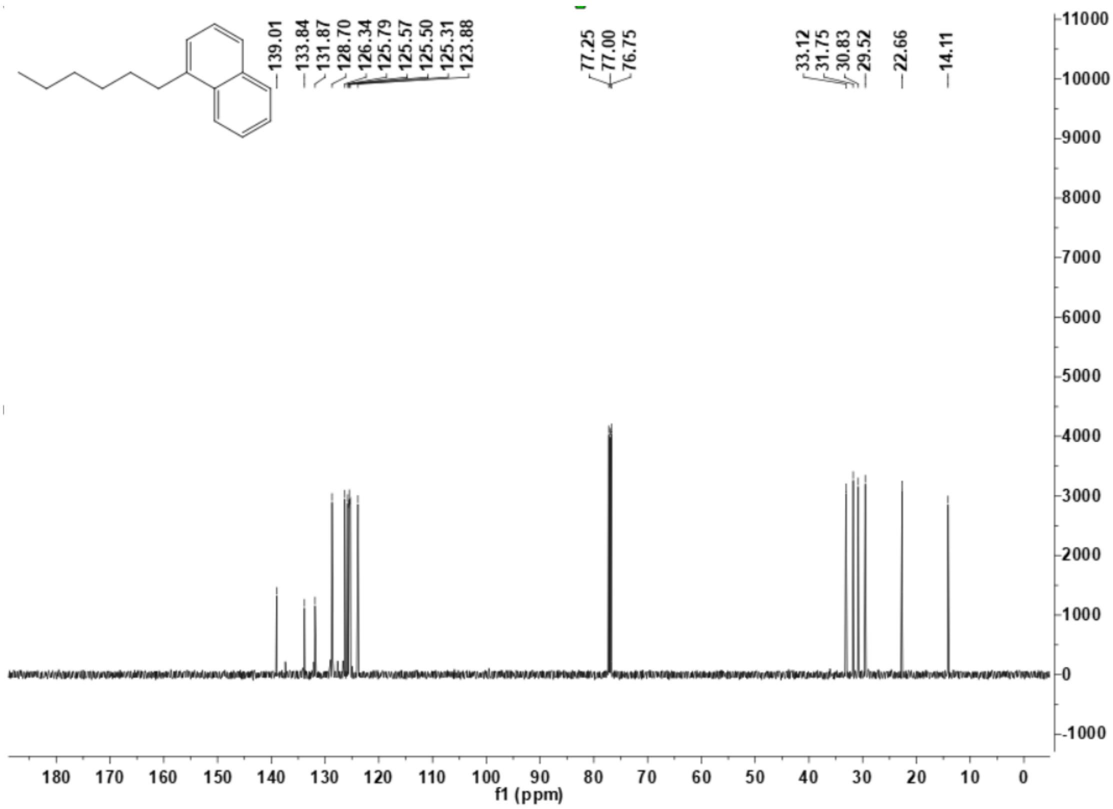

**1-Dodecylnaphthalene (3af)**

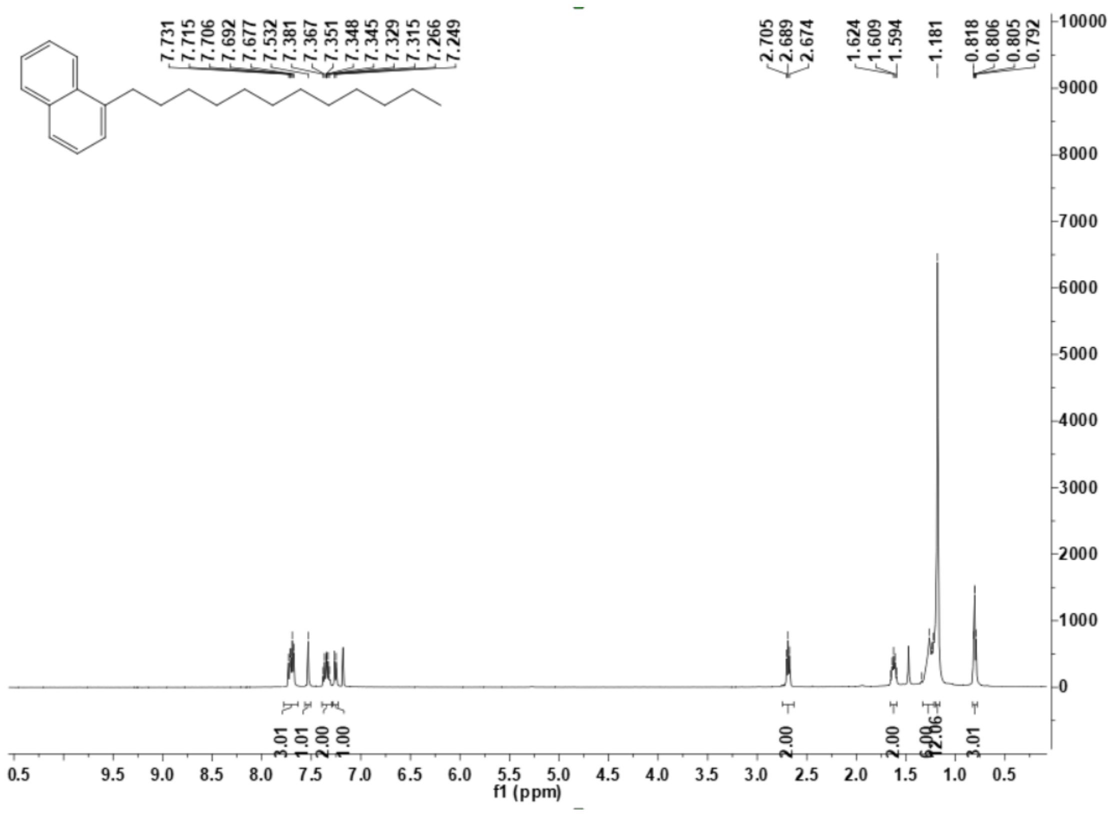

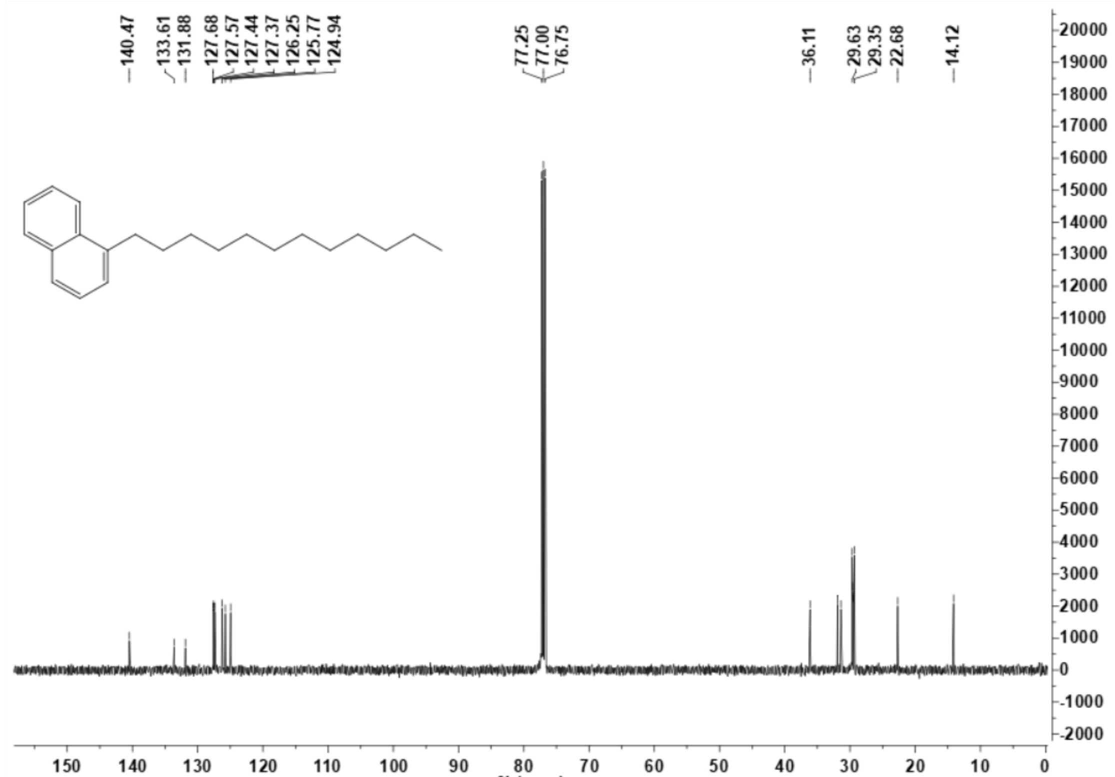

# 1-(3,3-dimethylbutyl) naphthalene (3ag)

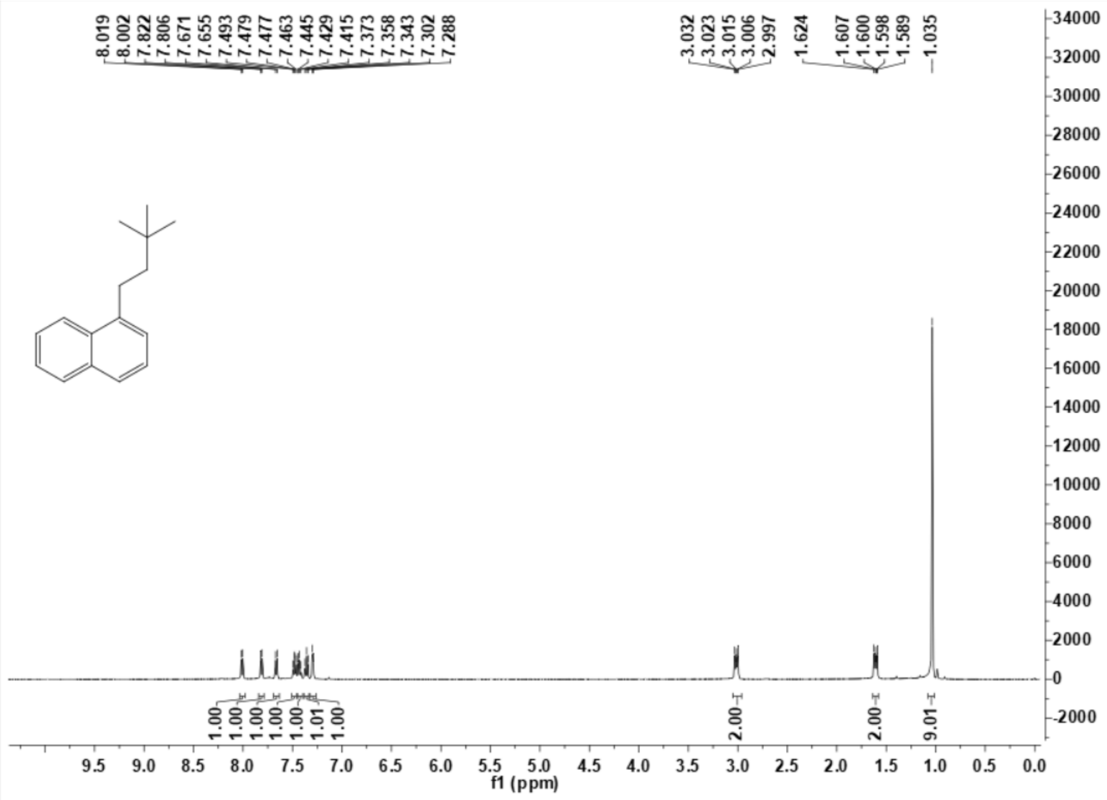


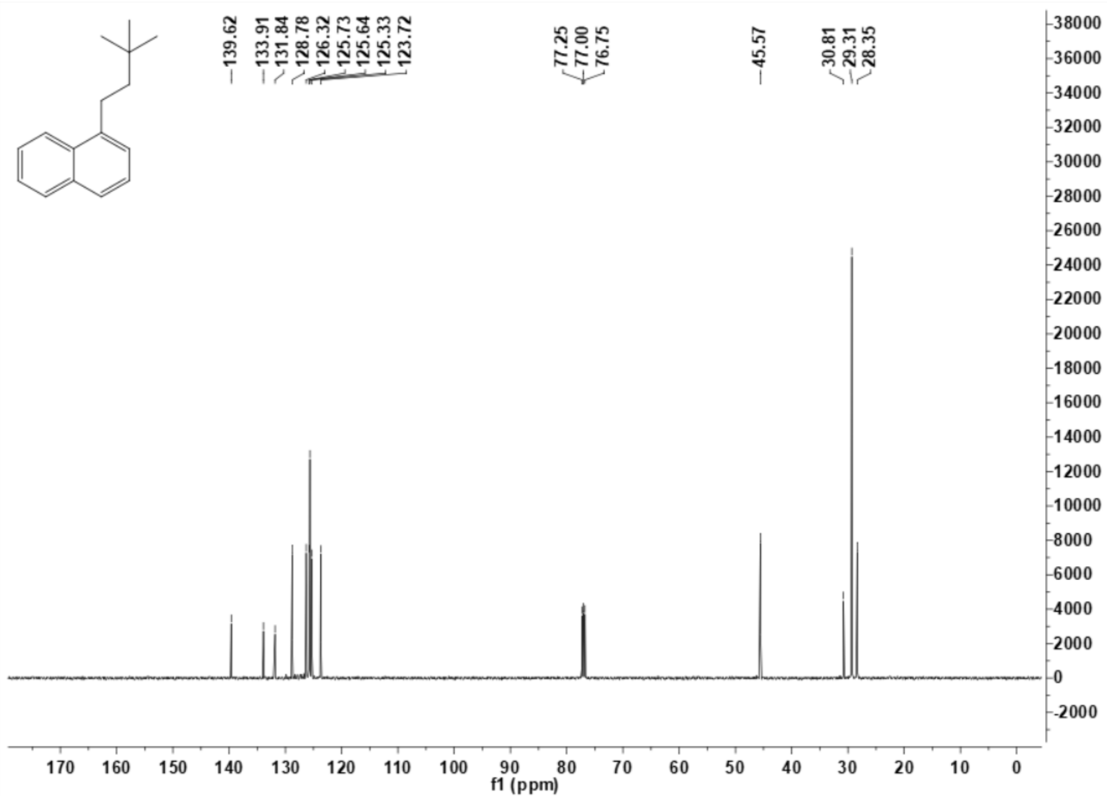

**1-(3-methoxypropyl) naphthalene (3ah)**

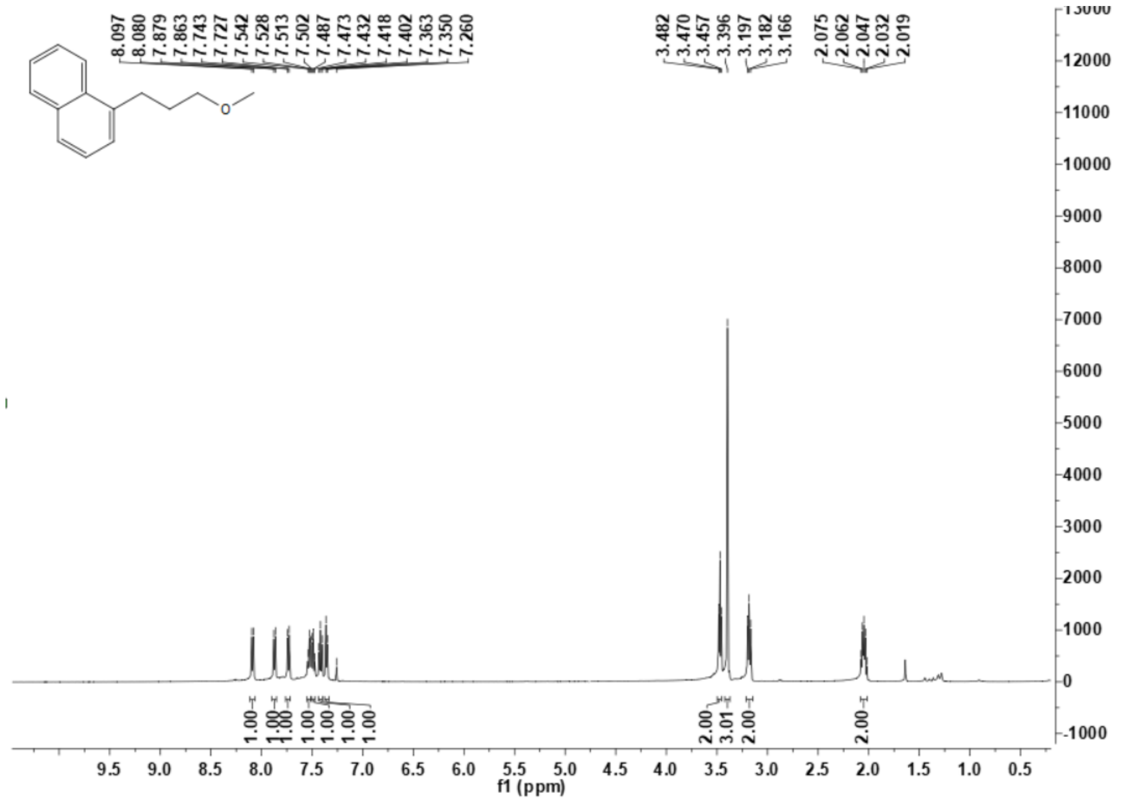

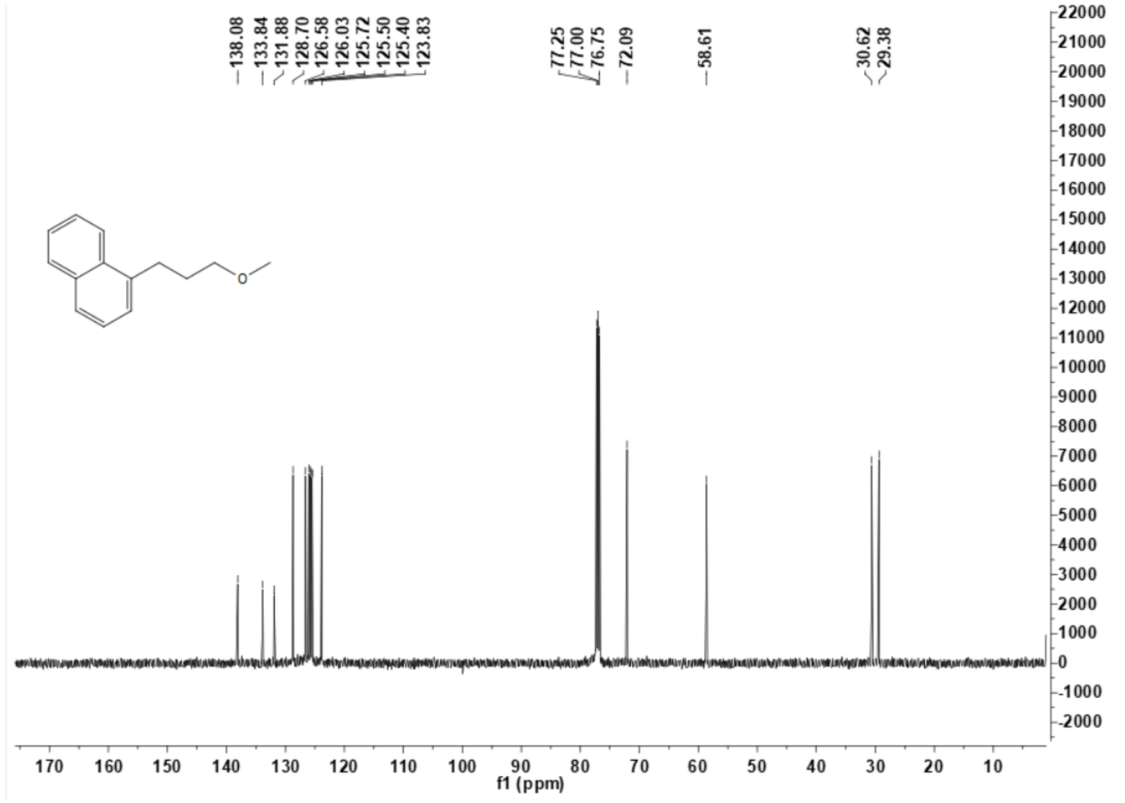

**1-(3,3-dimethoxypropyl) naphthalene (3ai)**

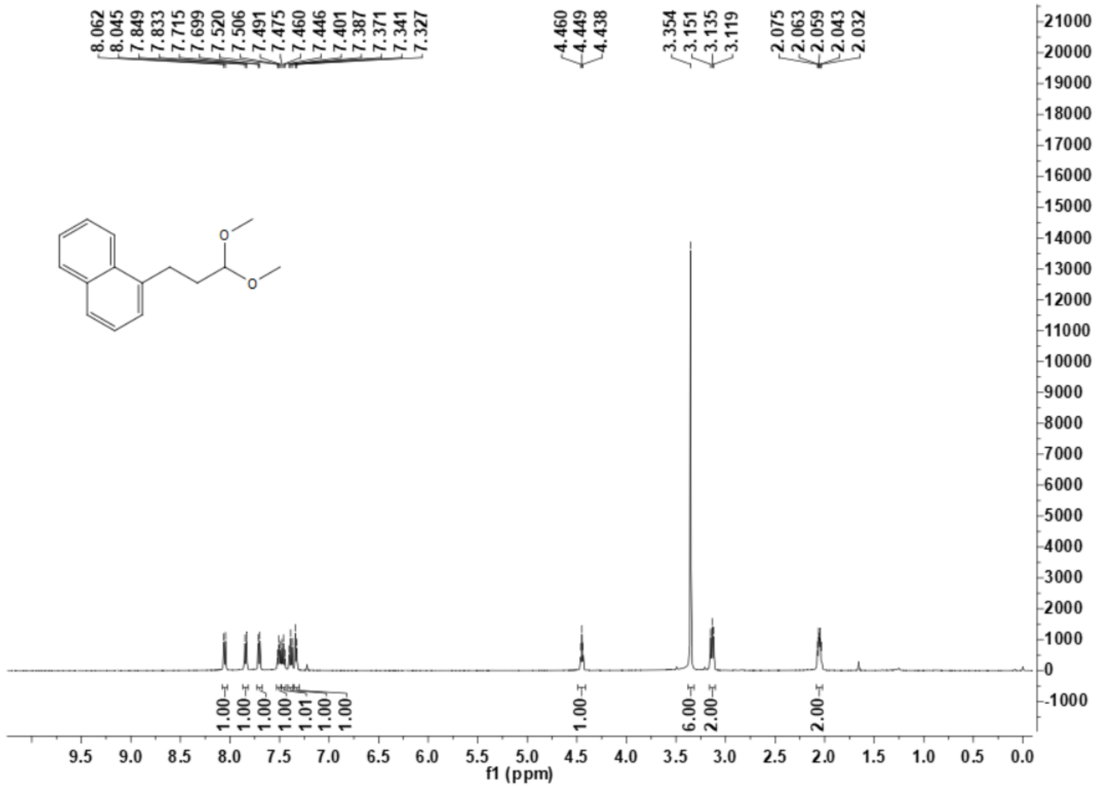


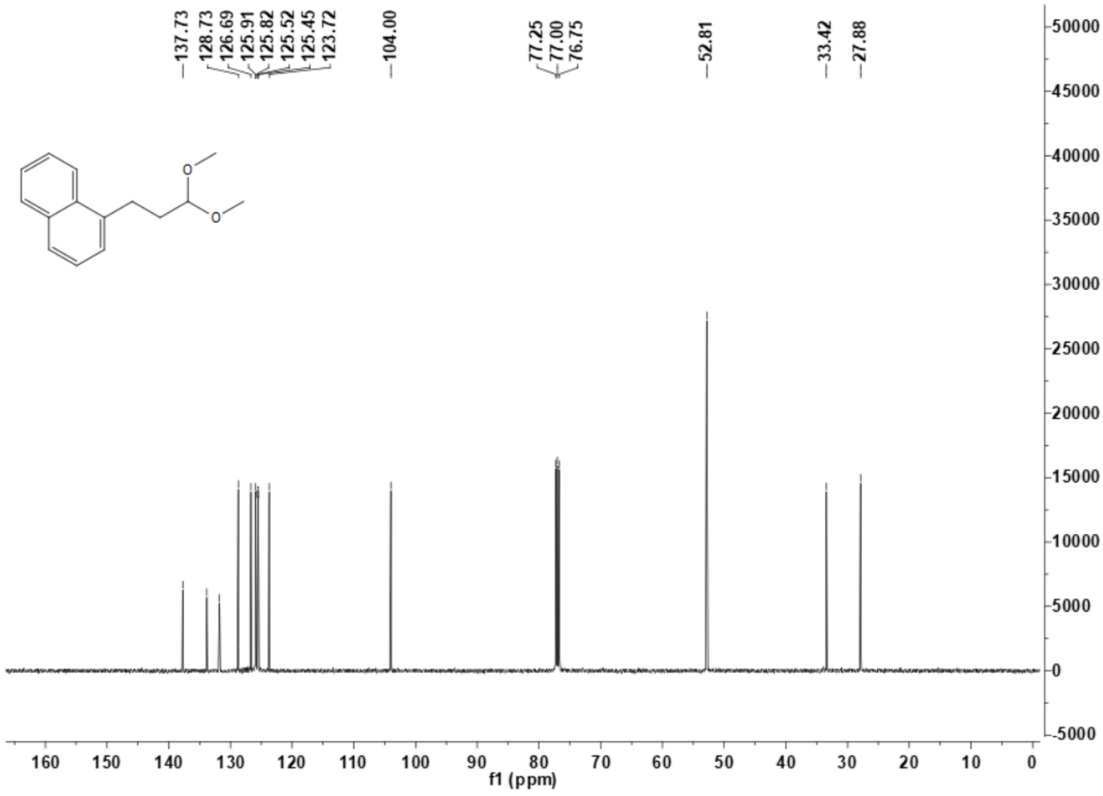

**2-(naphthalen-1-ylmethyl)-1,3-dioxolane (3aj)**

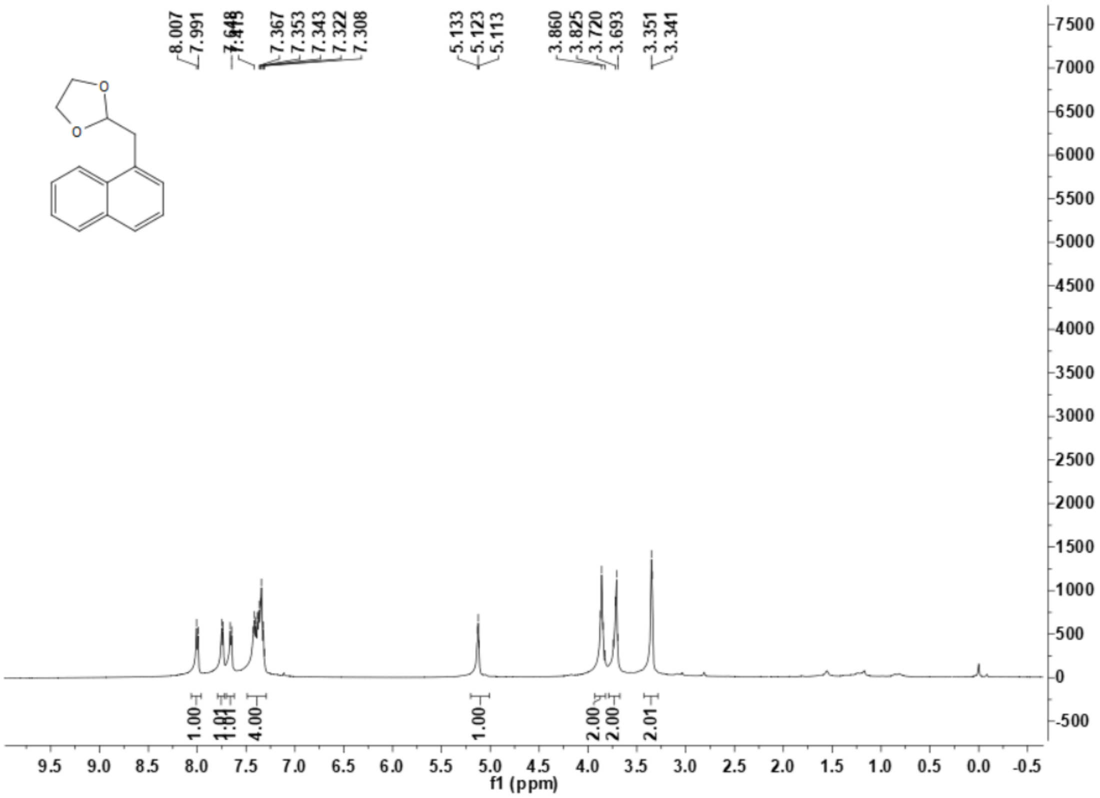


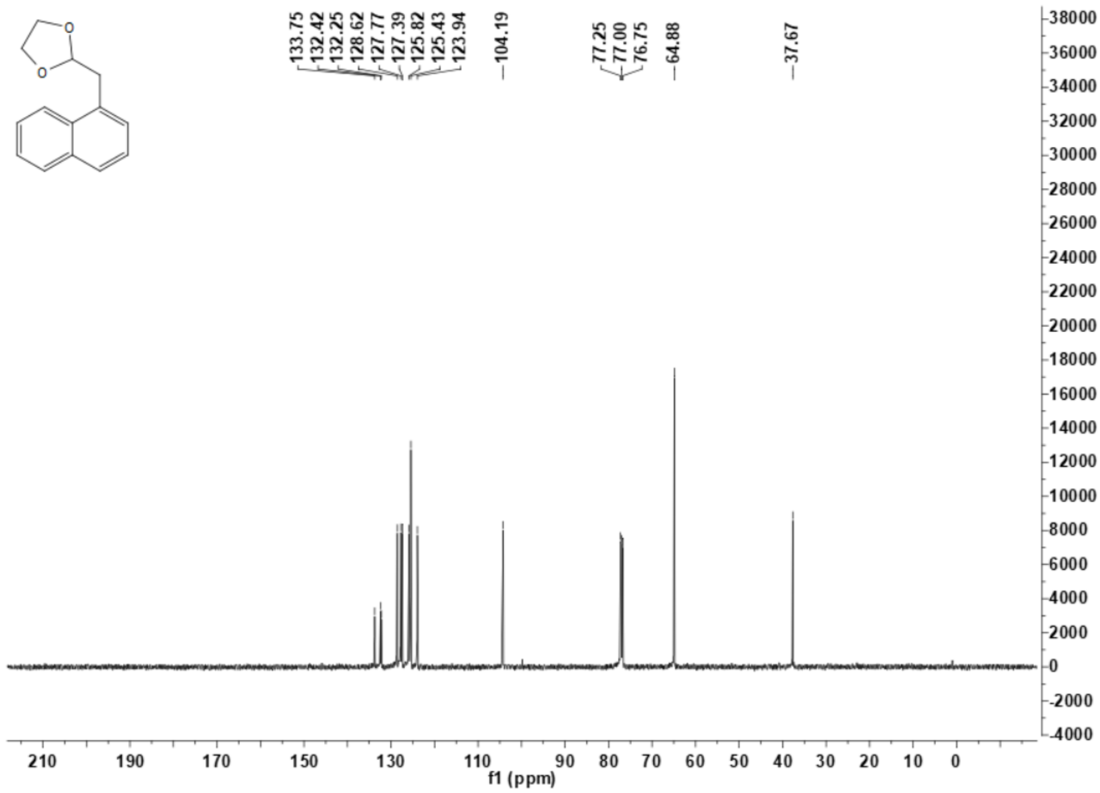

**2-(2-(naphthalen-1-yl) ethyl) isoindoline-1,3-dione (3ak)**

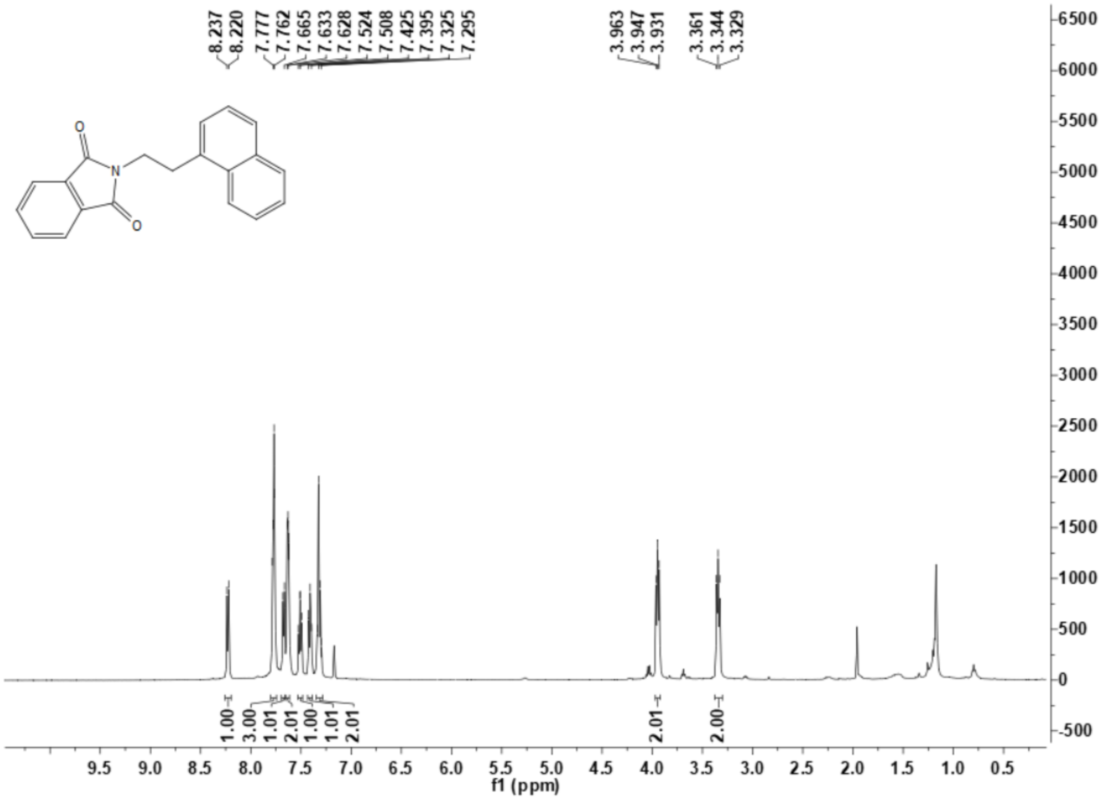


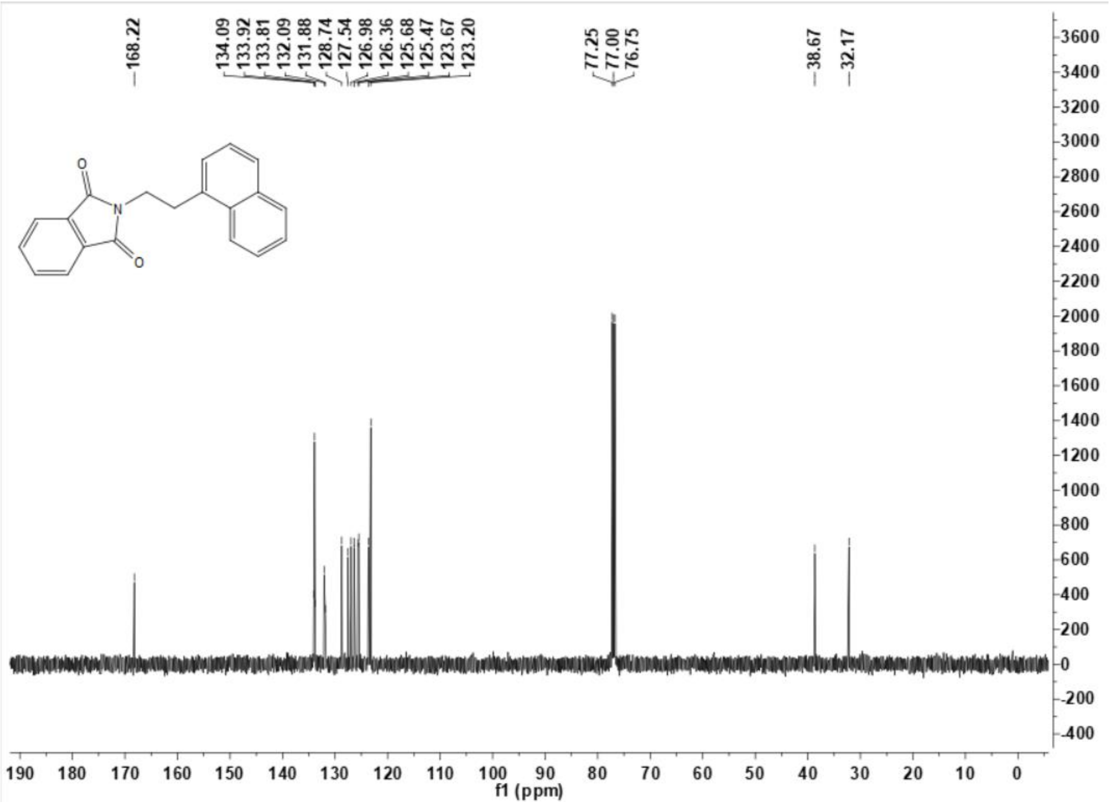

**2-(naphthalen-1-yl) ethan-1-ol (3al)**

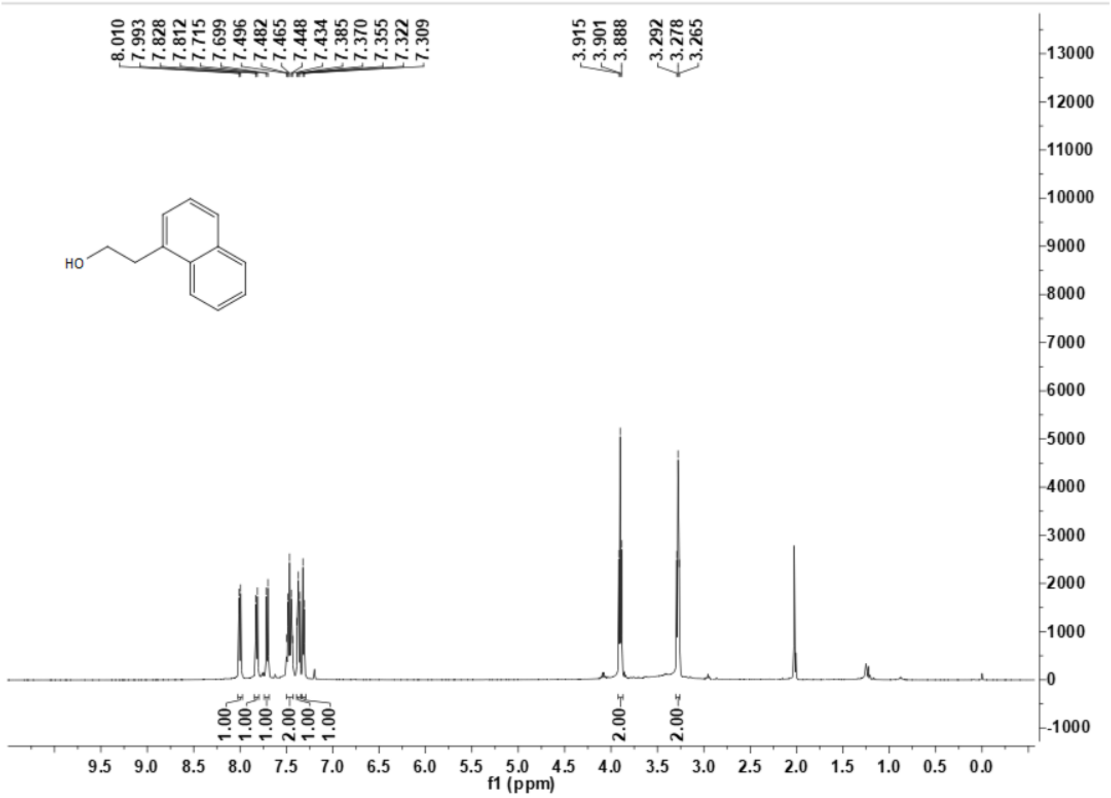


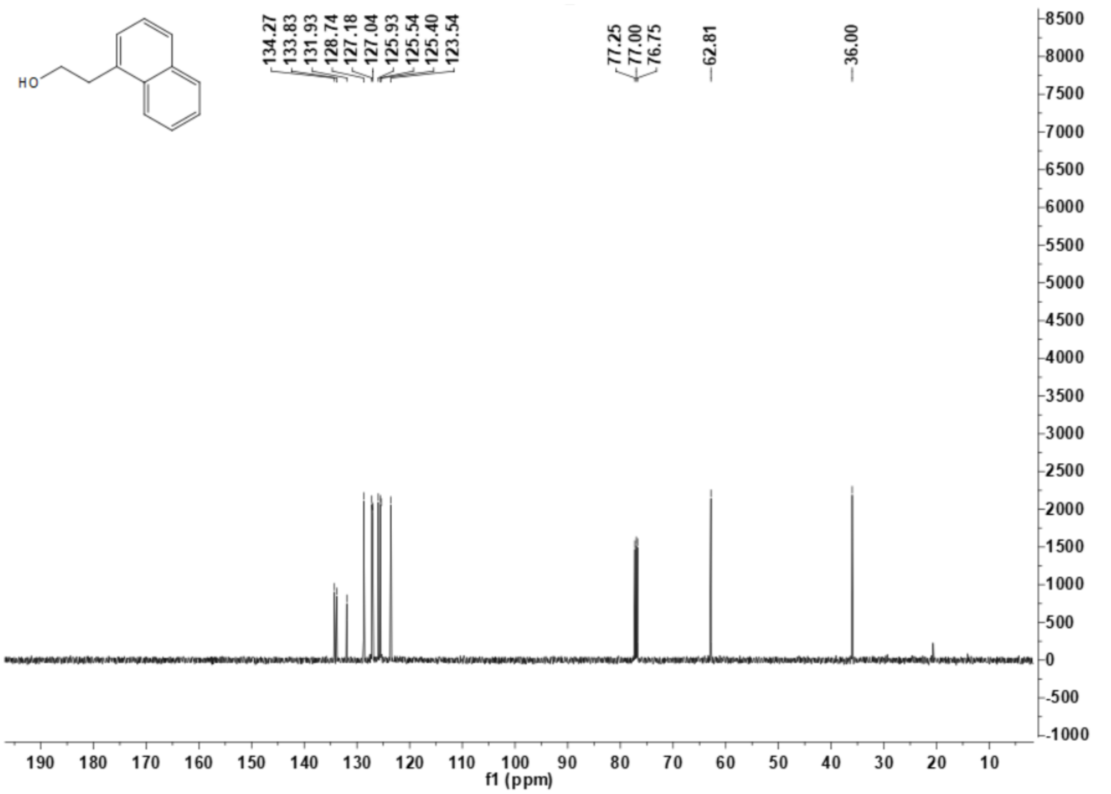

# 2-(naphthalen-1-yl) ethan-1-amine (3am)

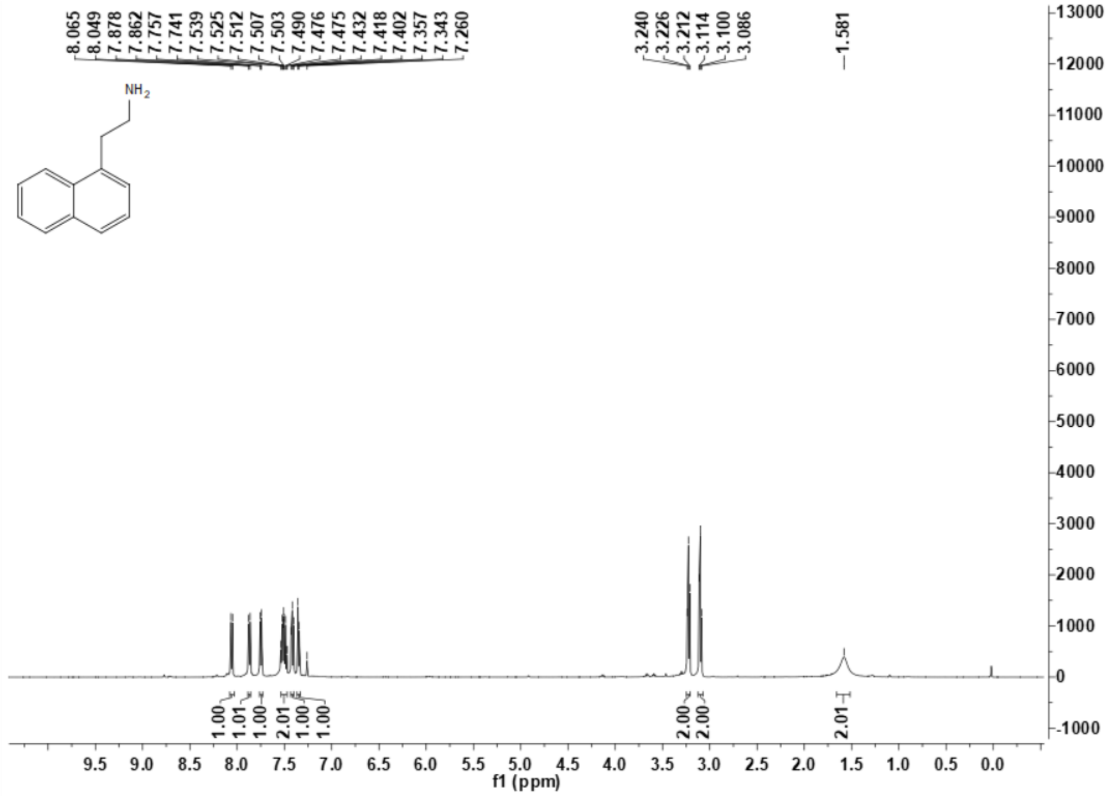


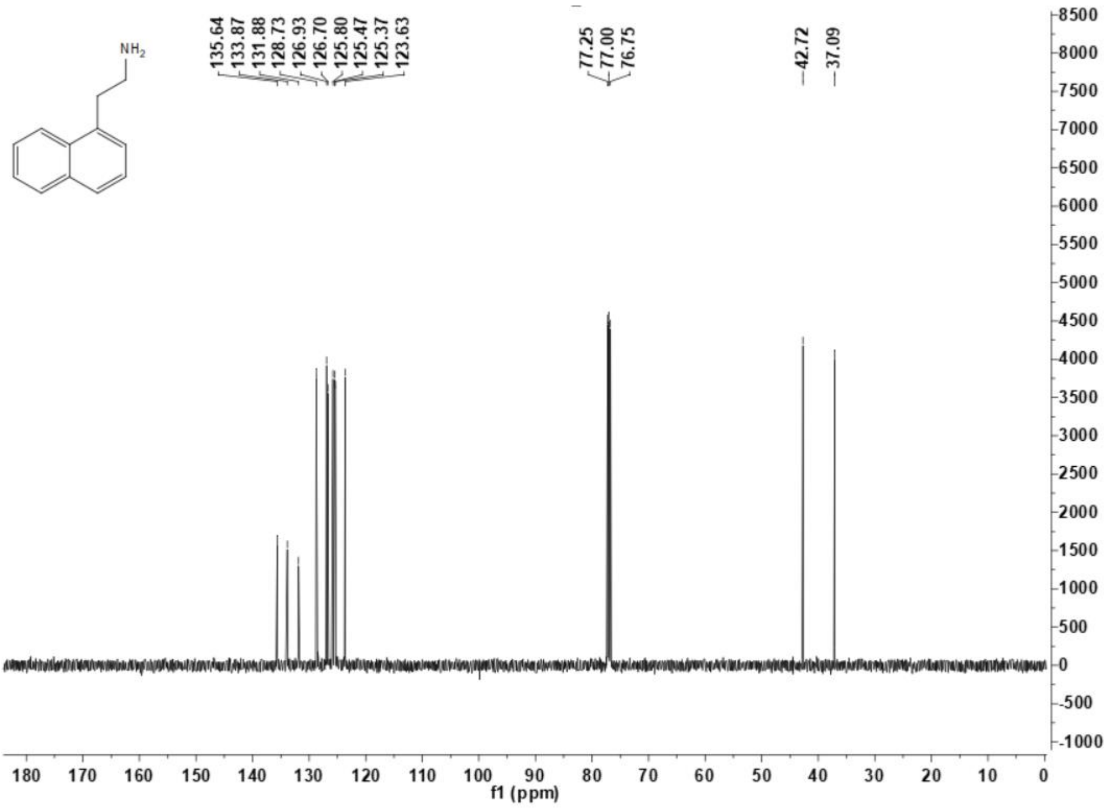

**Ethyl 3-(naphthalen-1-yl) propanoate (3an)**

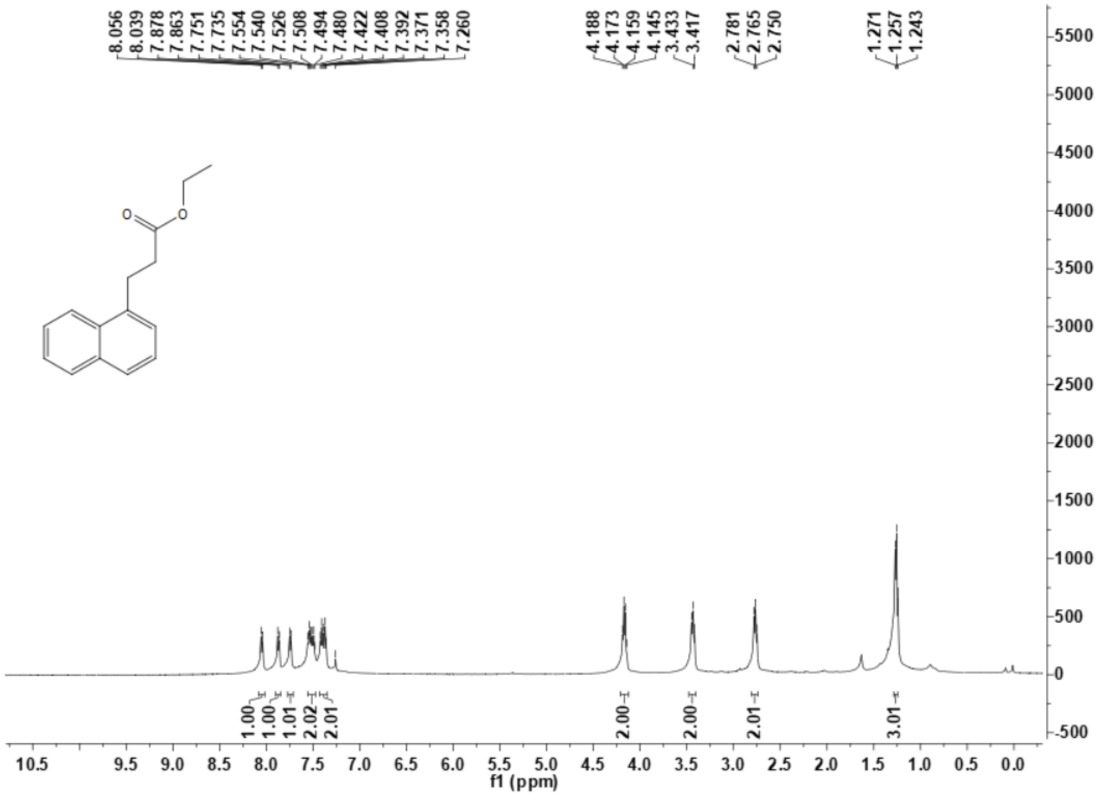


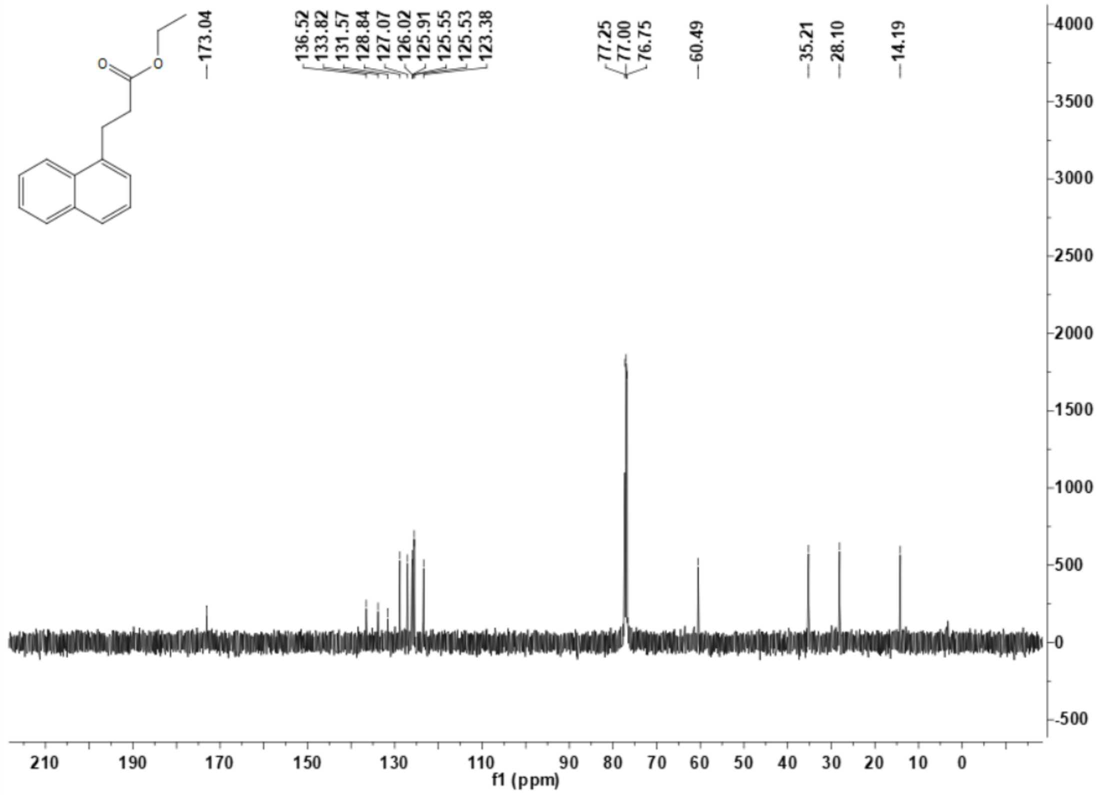

## Ethyl 4-(naphthalen-1-yl) butanoate (3ao)

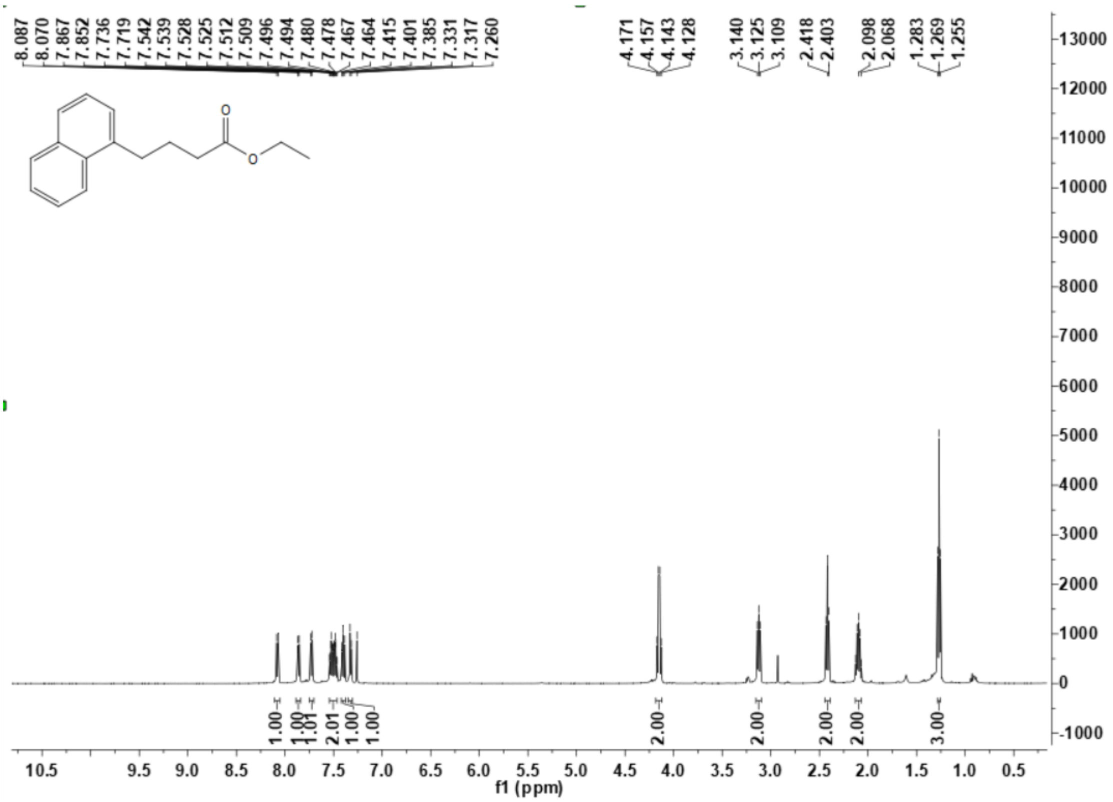

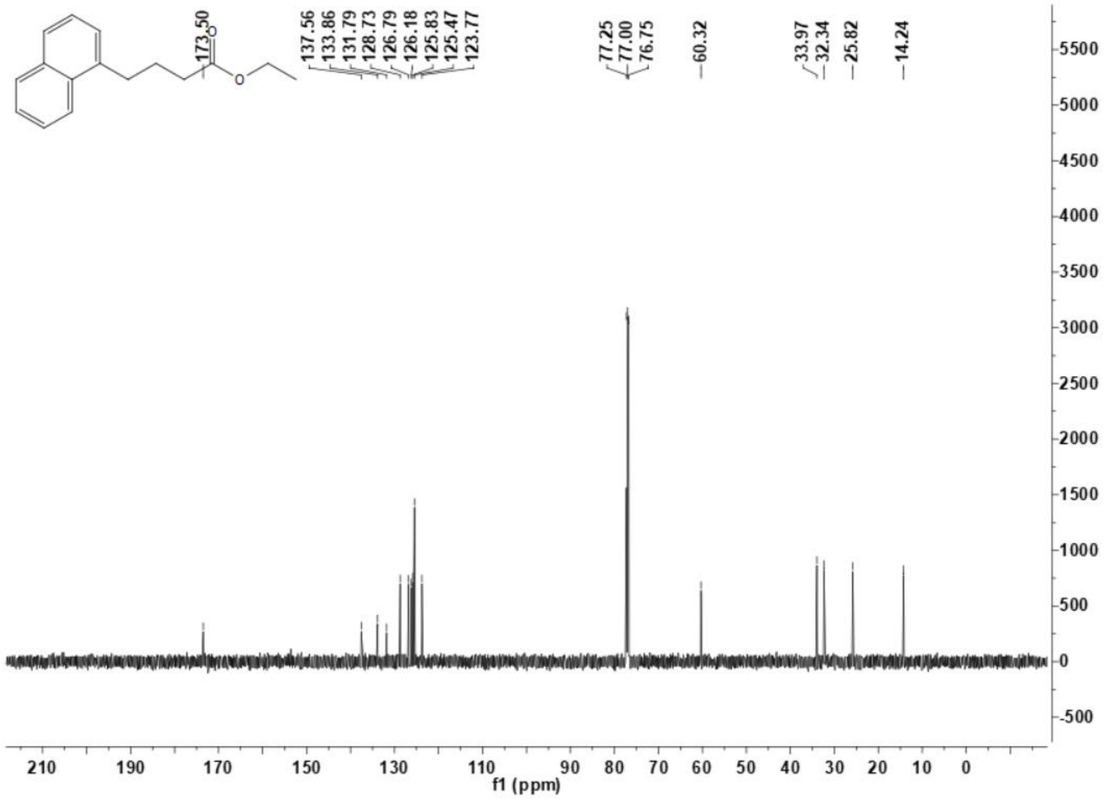

**Benzyl 4-(naphthalen-1-yl) butanoate (3ap)**

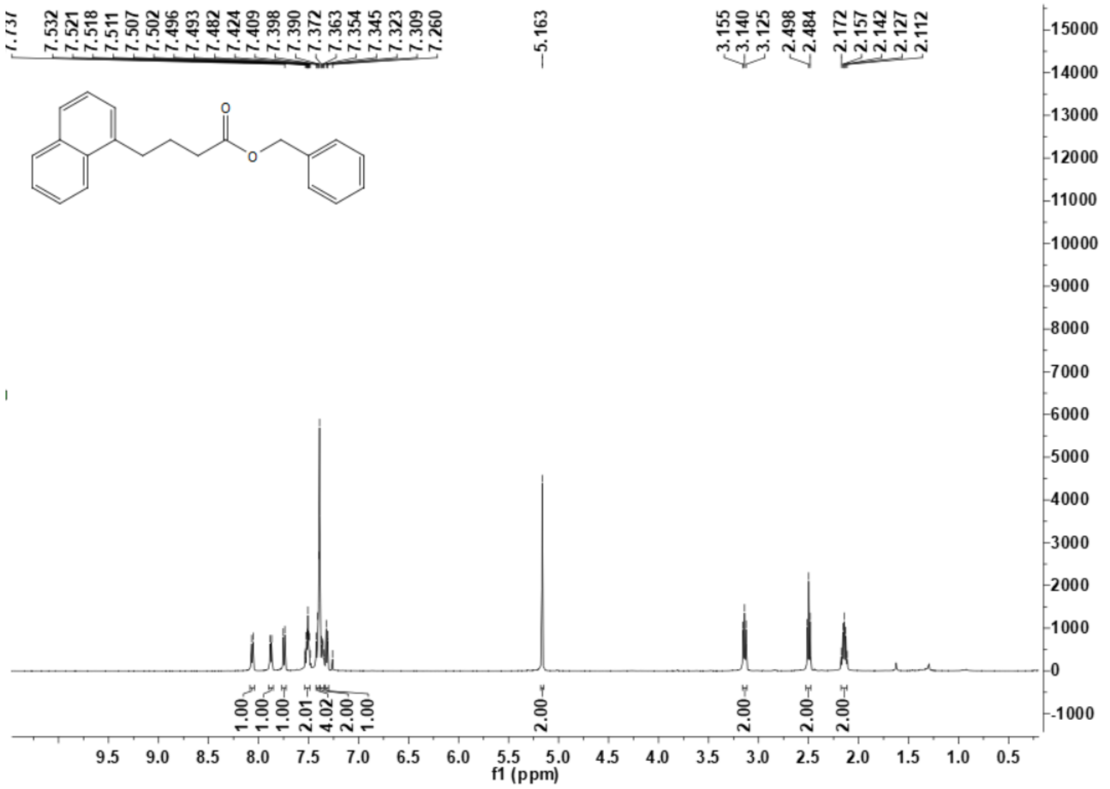

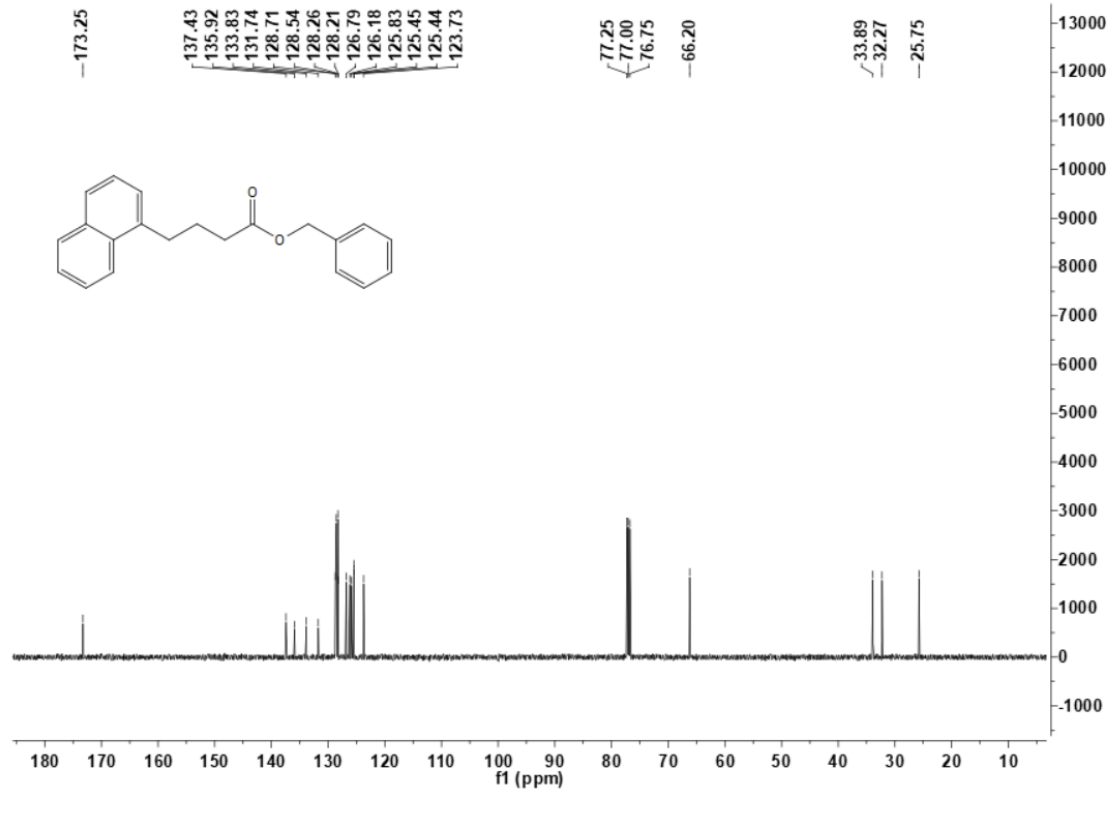

**Methyl 5-(naphthalen-1-yl) pentanoate (3aq)**

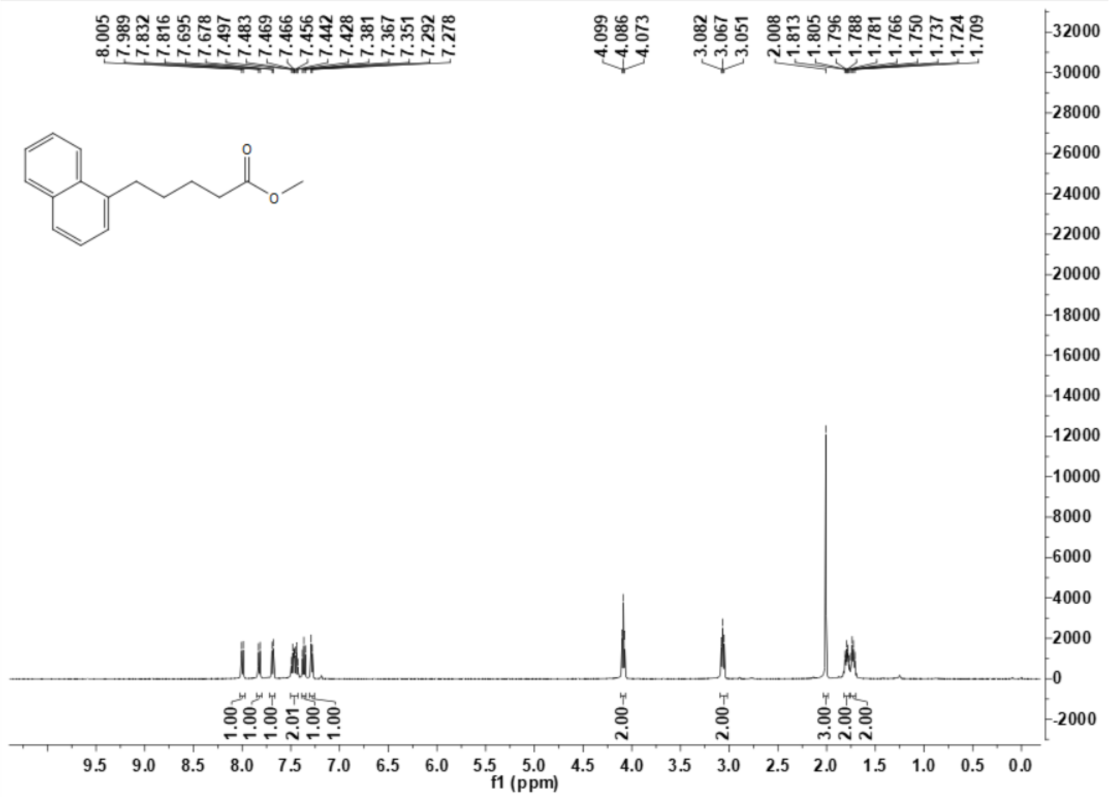


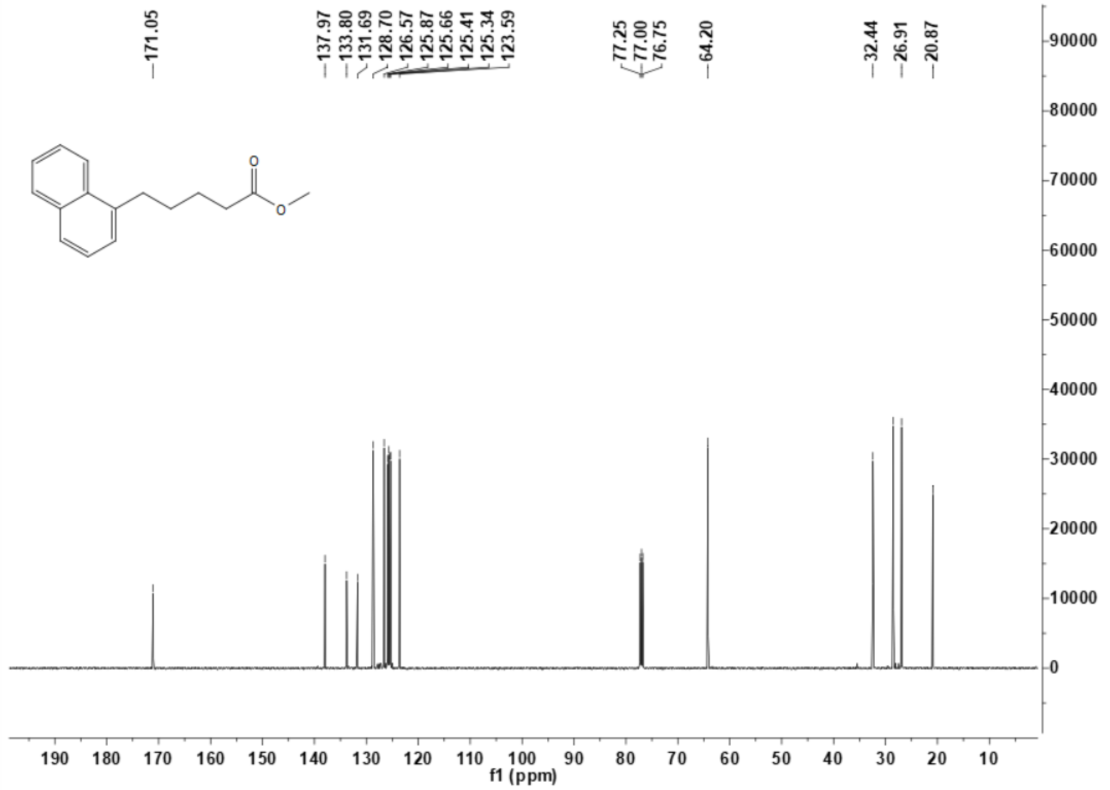

**4-(naphthalen-1-yl) butanenitrile (3ar)**

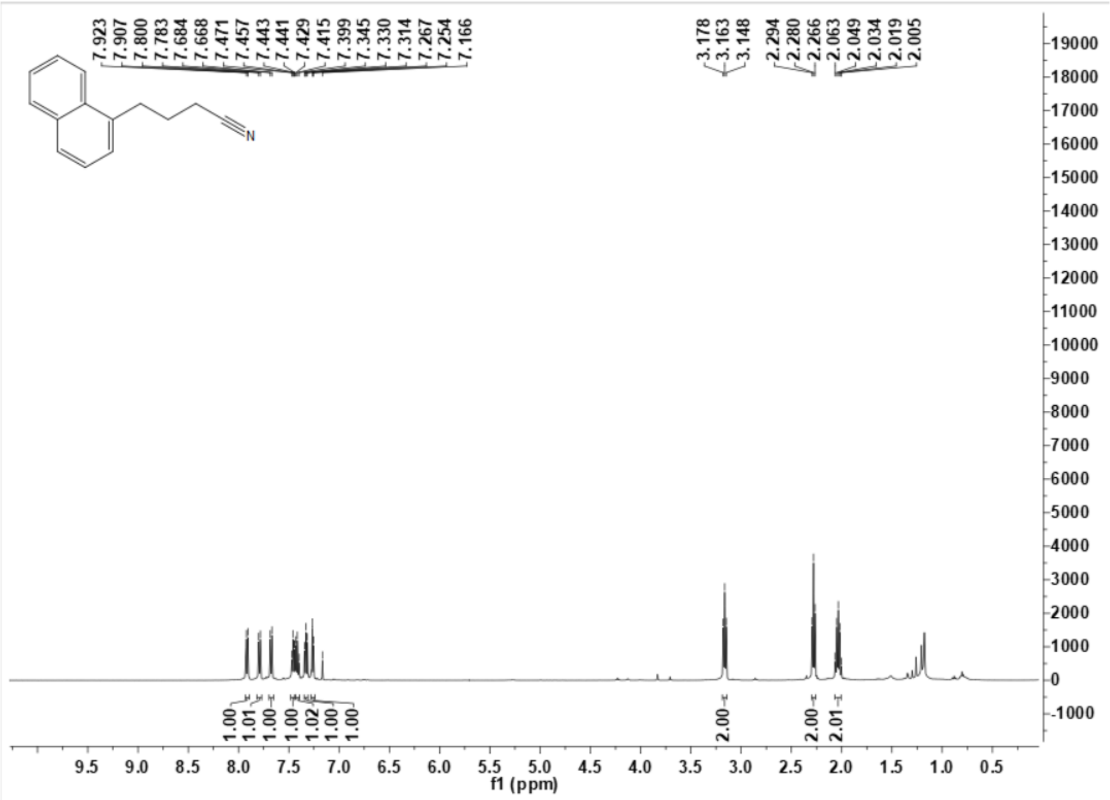


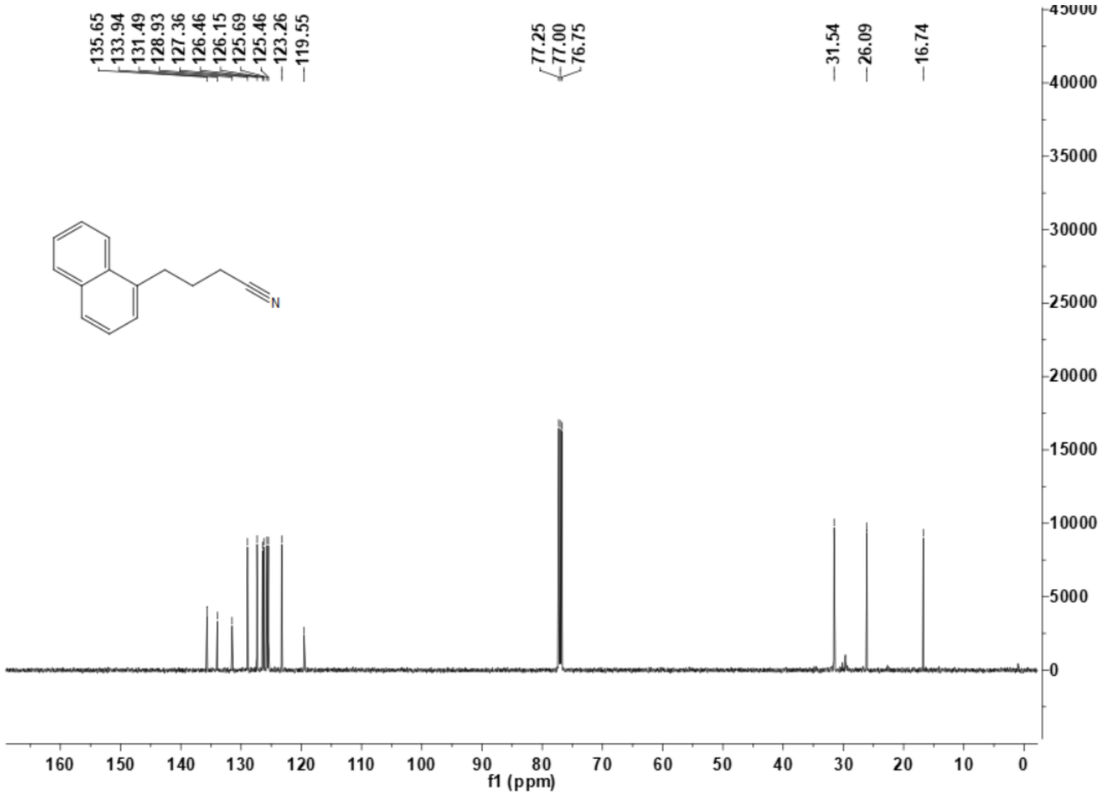

**3-(naphthalen-1-yl)propanoic acid (3as)**

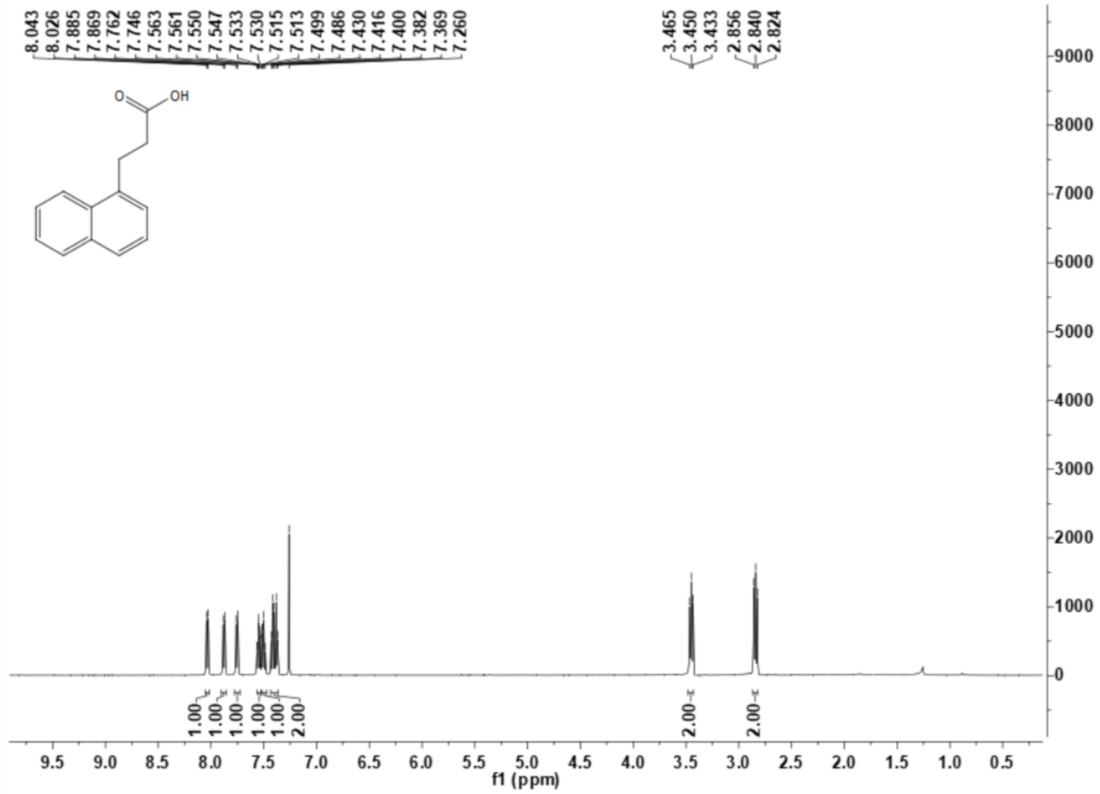


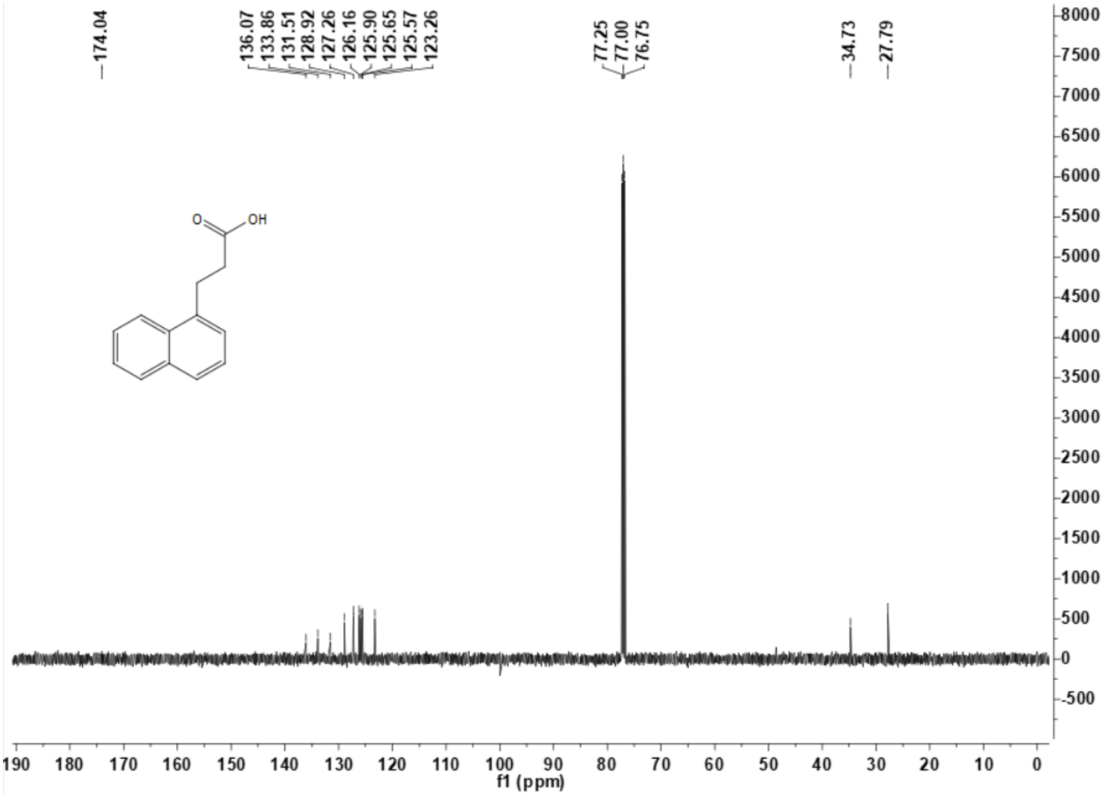

**1-cyclopropylnaphthalene (3at)**

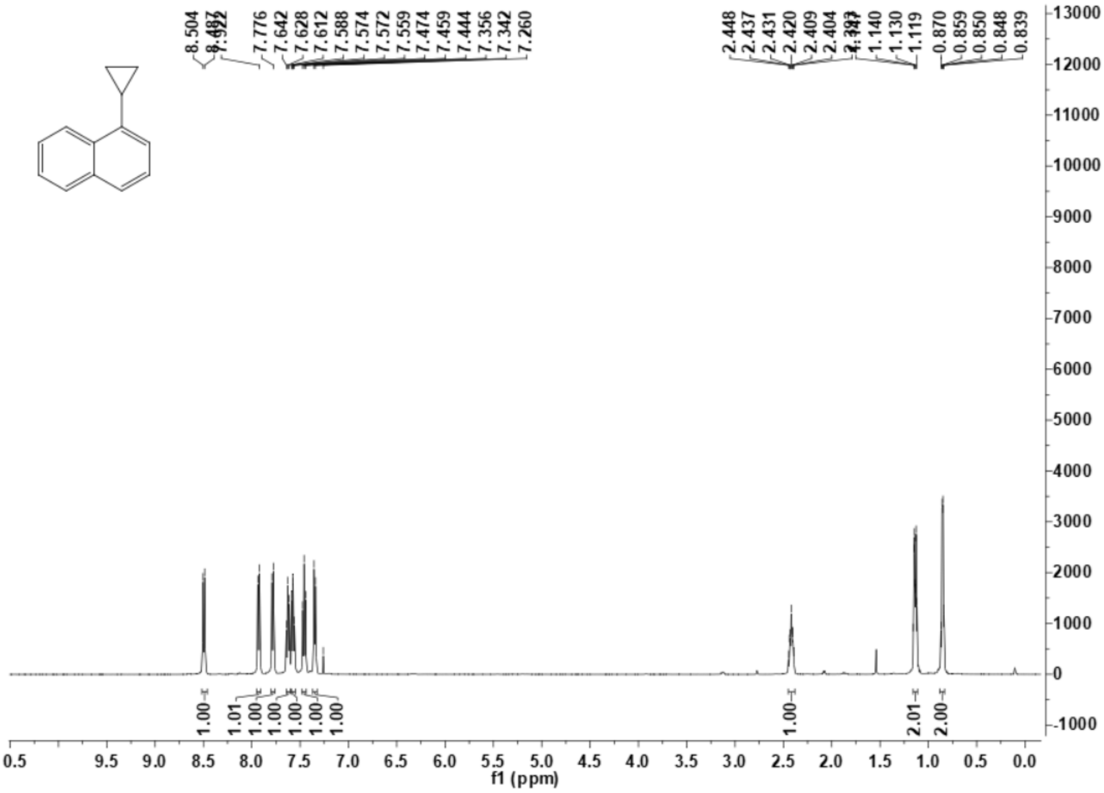


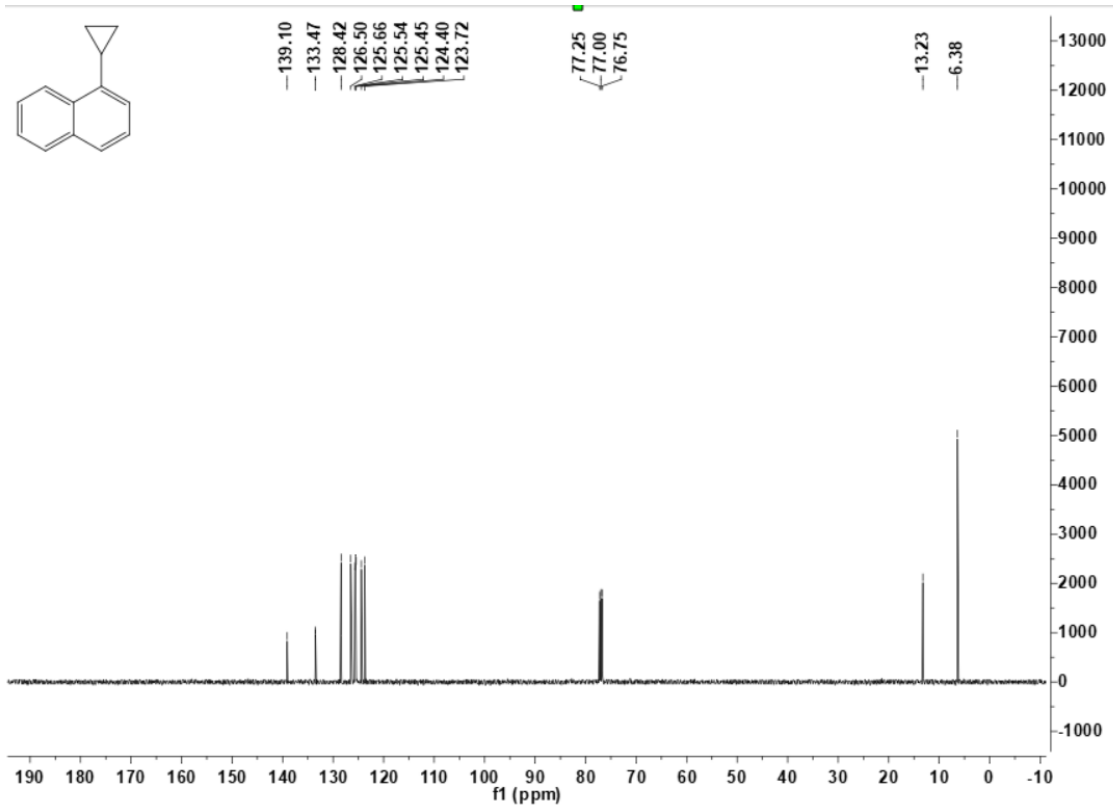

## 1-cyclohexylnaphthalene (3au)

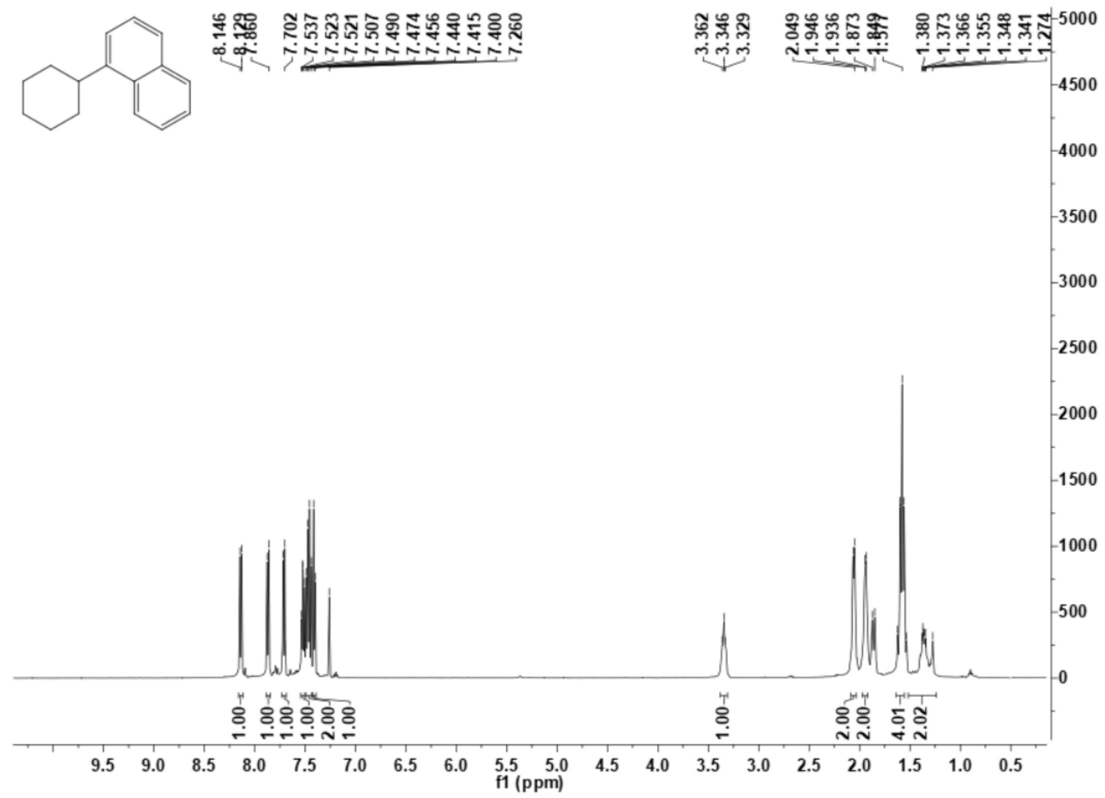

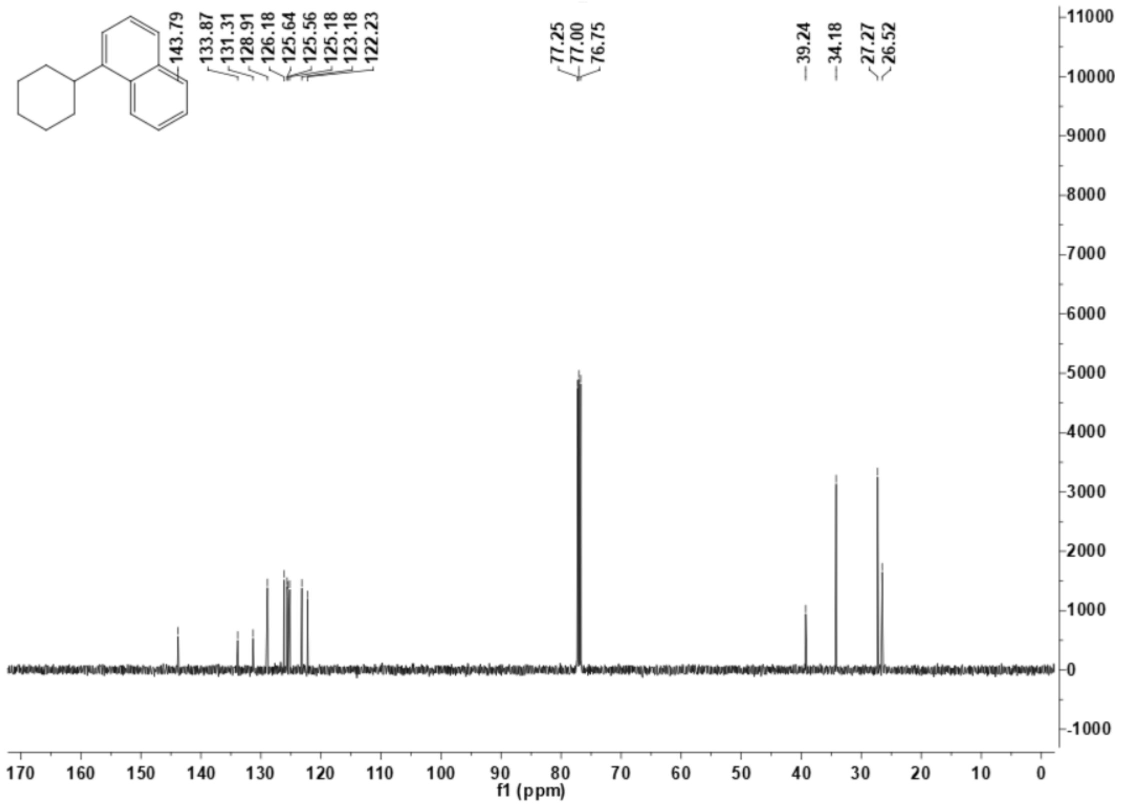

**1-(4-methoxybenzyl) naphthalene (3bv)**

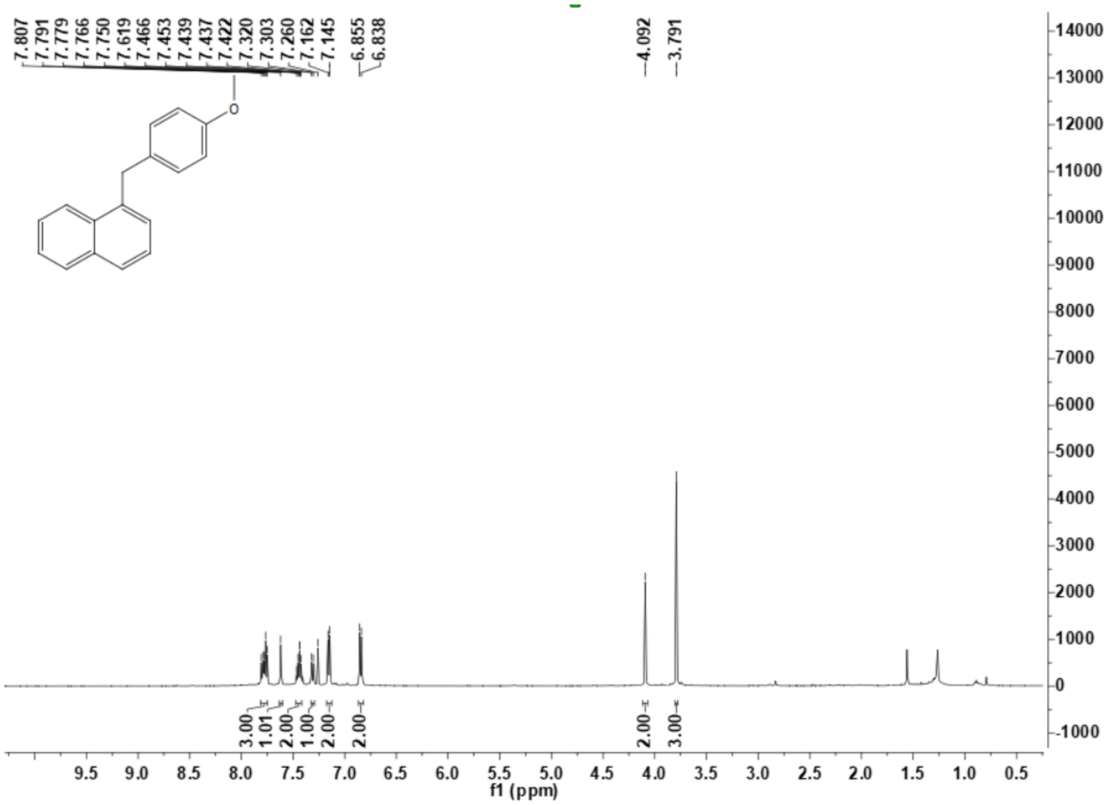


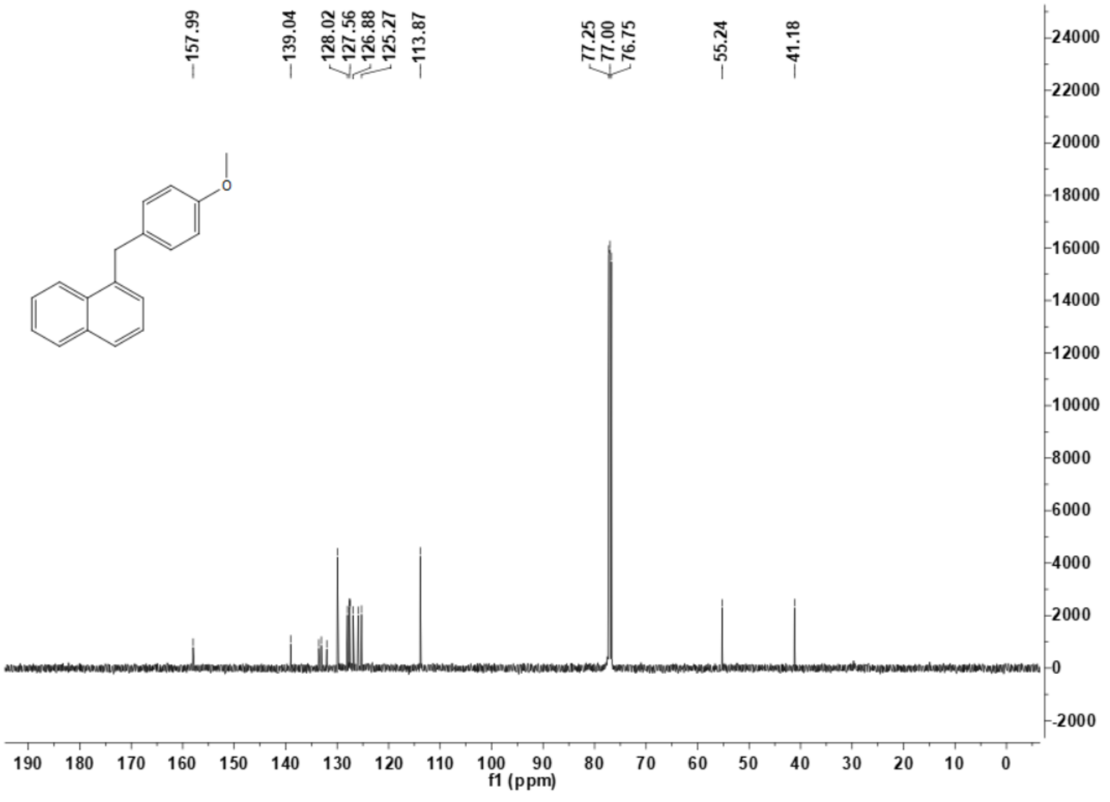

# 1-benzyl-4-methoxybenzene (3cv)

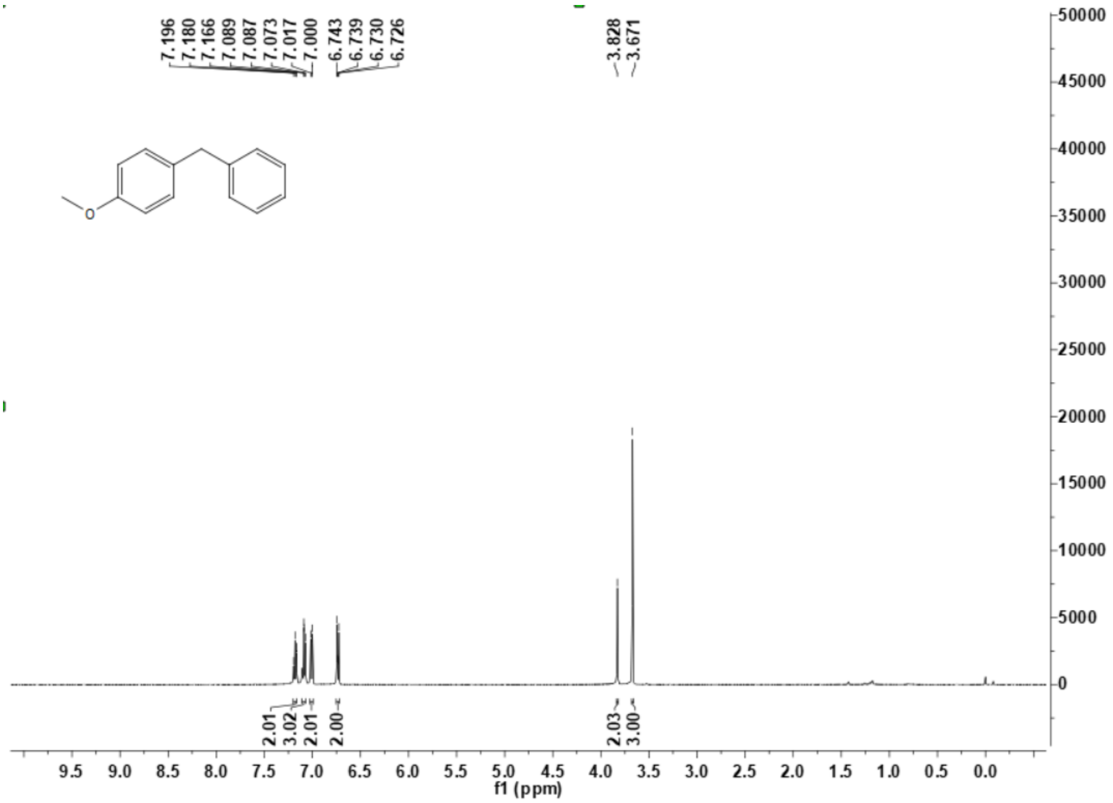


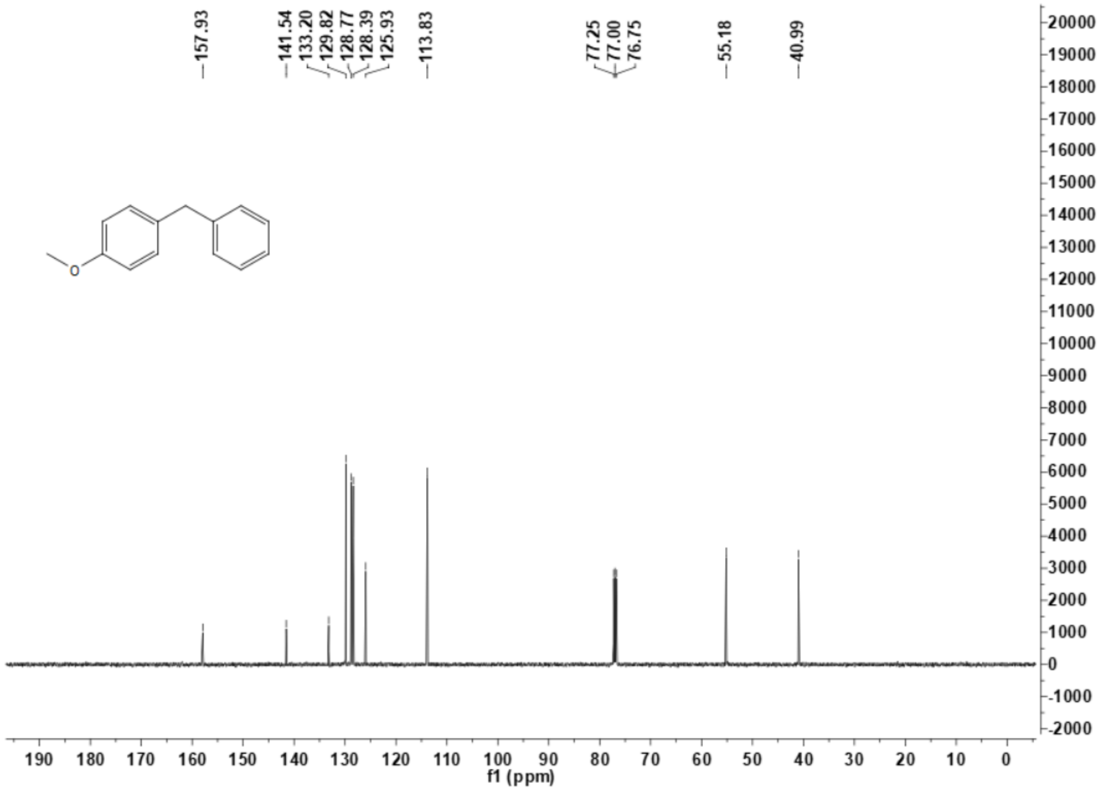

**4-(4-fluorobenzyl)-1,1'-biphenyl (3dw)**

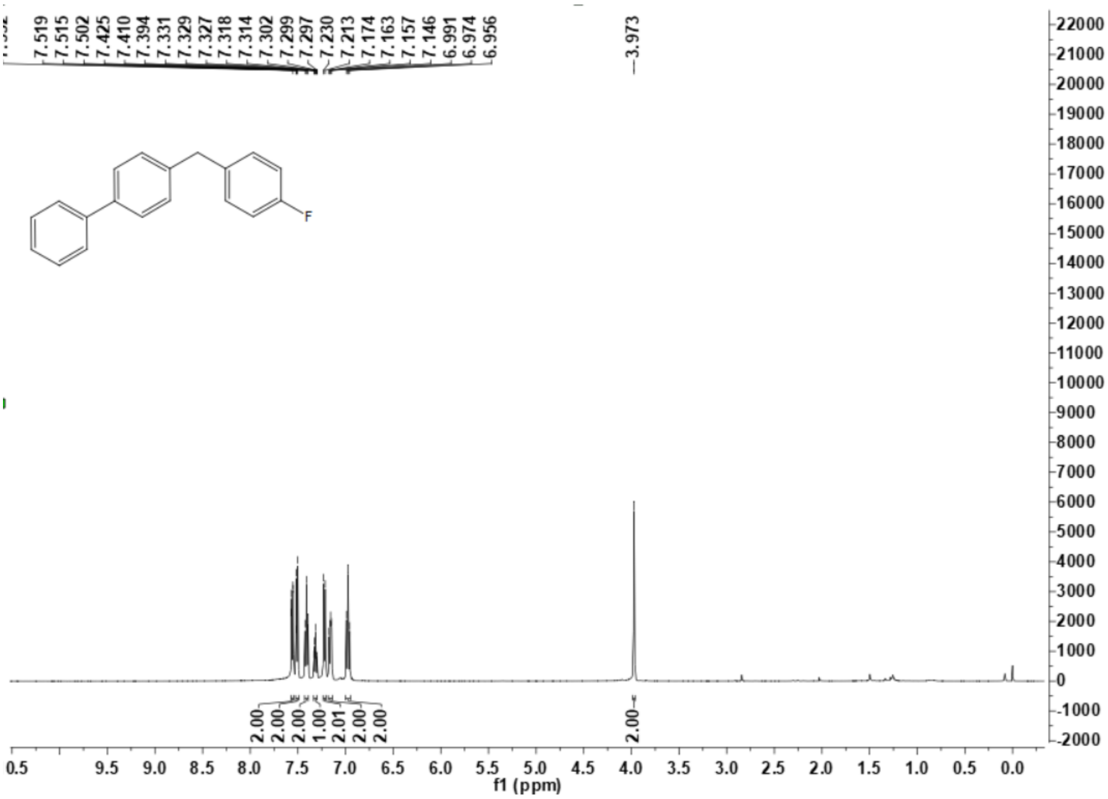


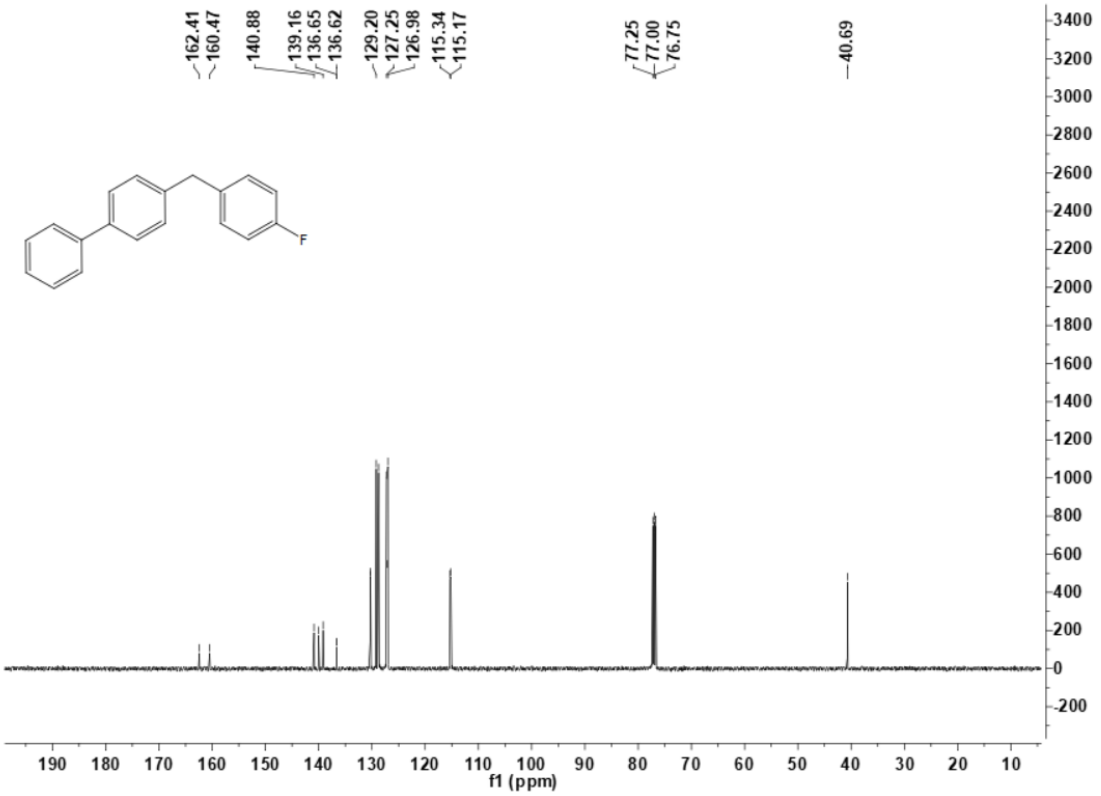

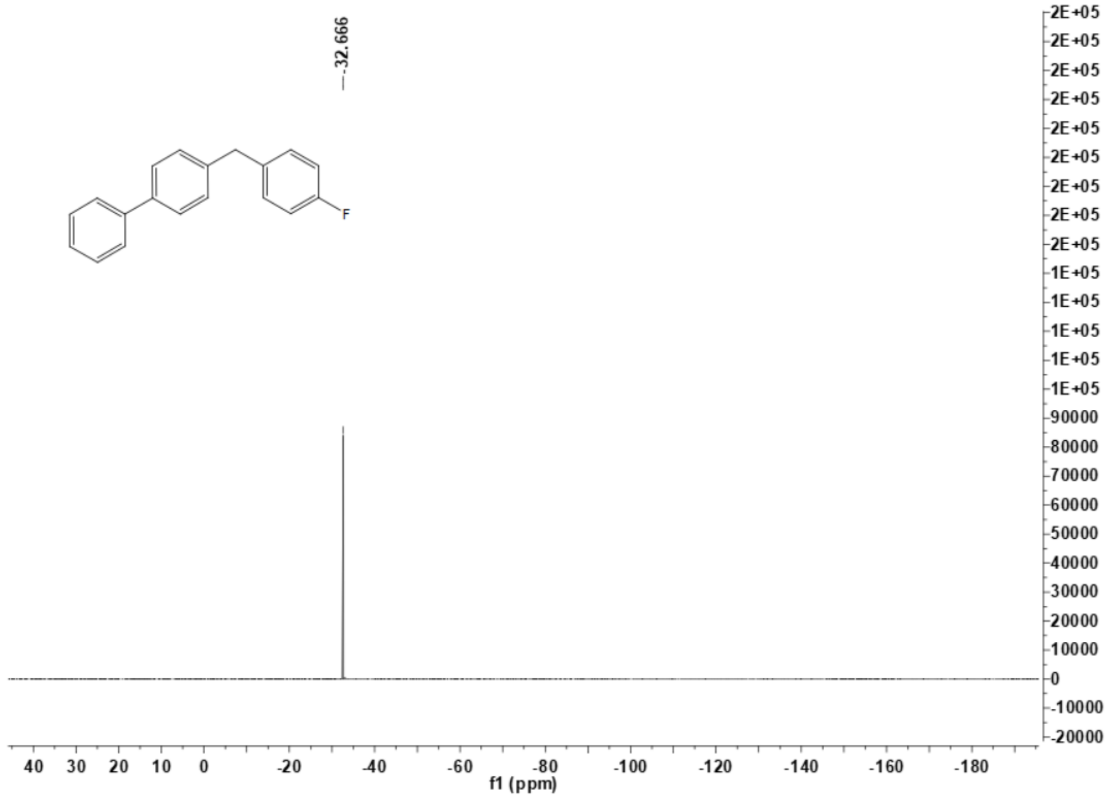


**4-(3-methylbenzyl)-1,1'-biphenyl (3dx)**

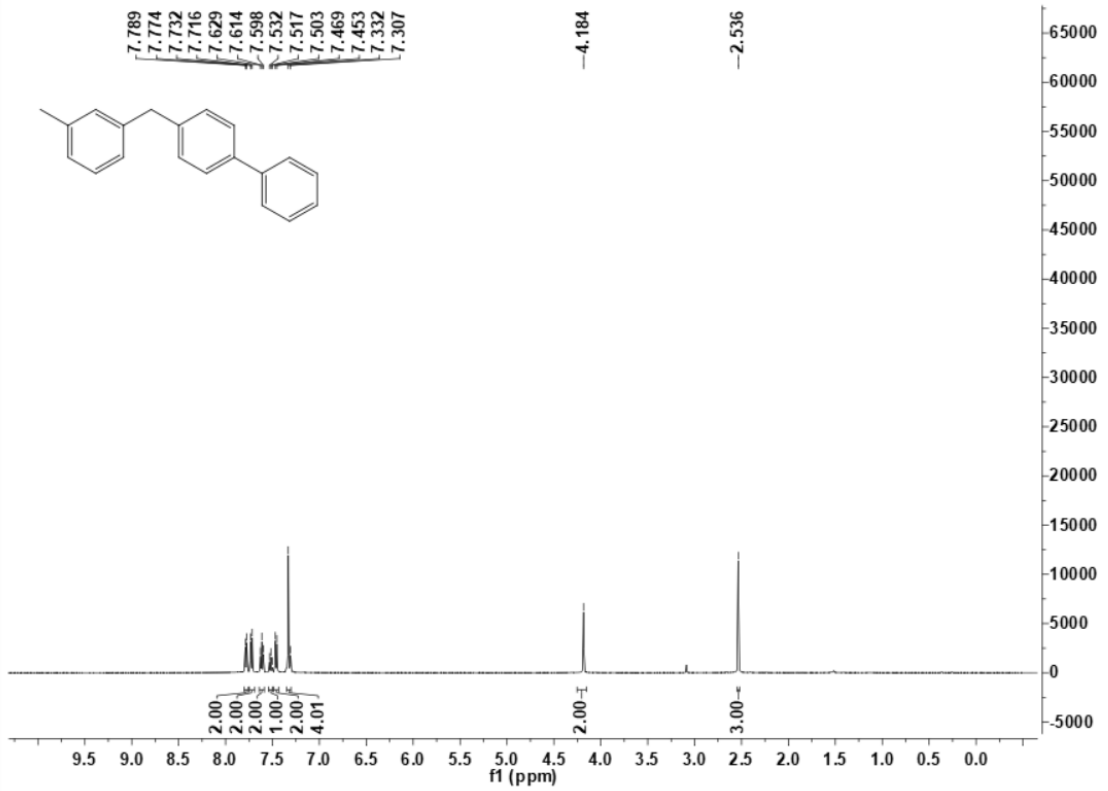

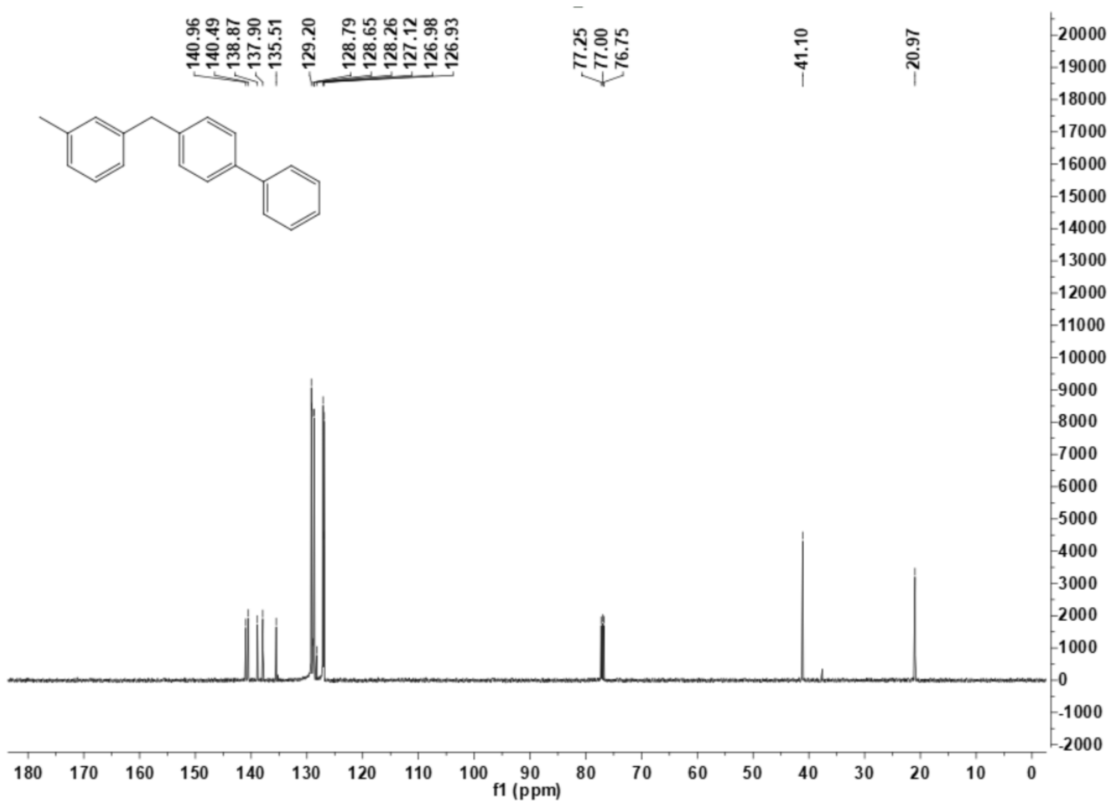

**1-benzyl-4-(trifluoromethoxy) benzene (3ey)**

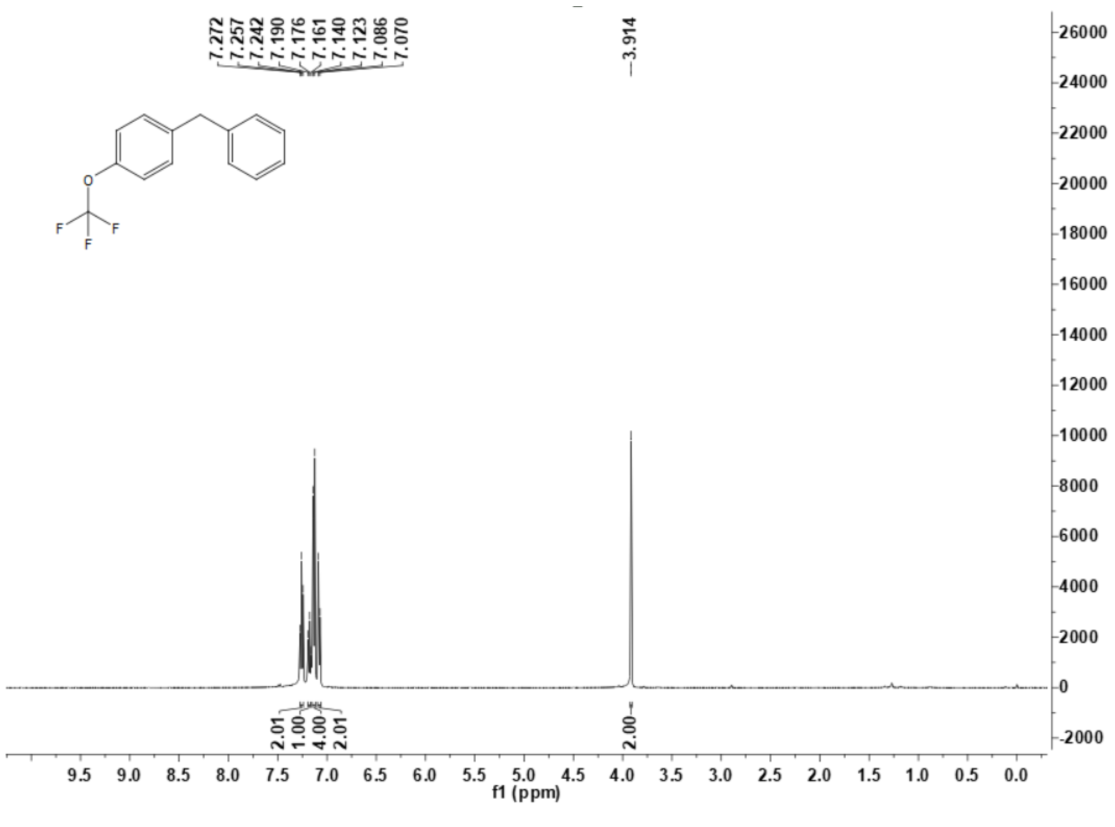

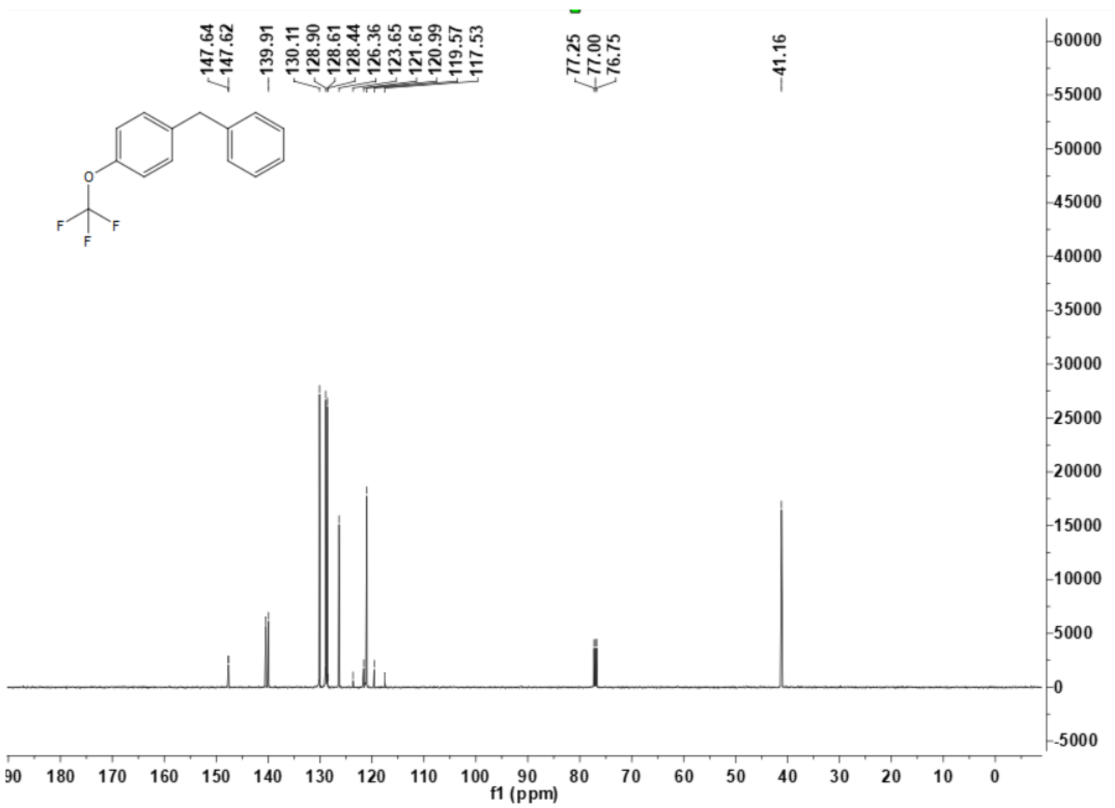
147.64
147.62
139.91
130.11
128.90
128.61
128.44
126.36
123.65
121.61
120.99
119.57
117.53
77.25
77.00
76.75
41.16
F
F
F
O
f1 (ppm)

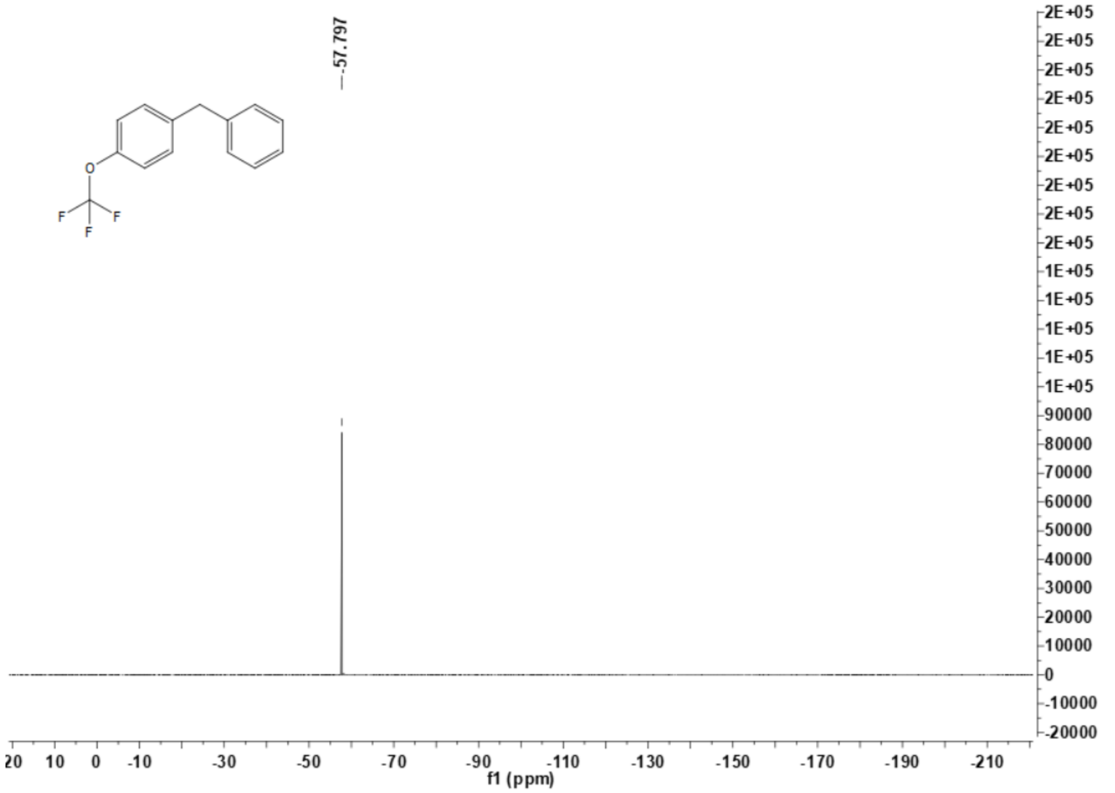
-57.797
F
F
F
O
f1 (ppm)

**(4-benzylphenyl) (methyl) sulfane (3fy)**

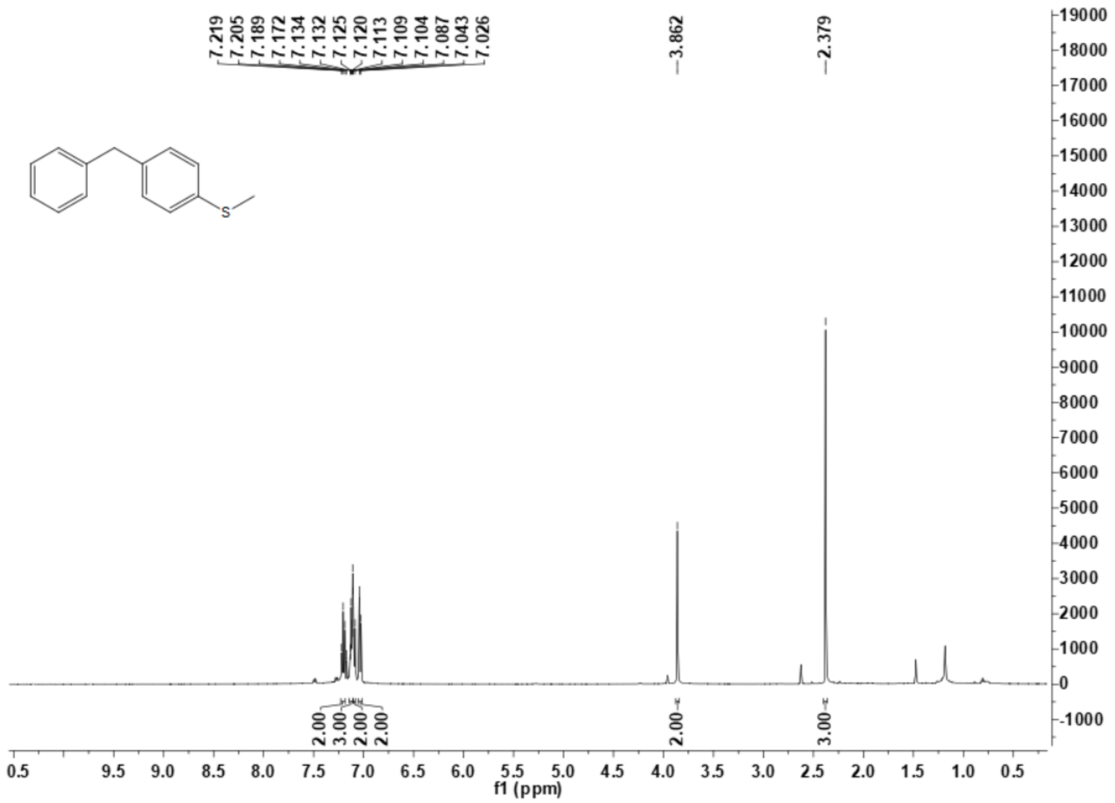

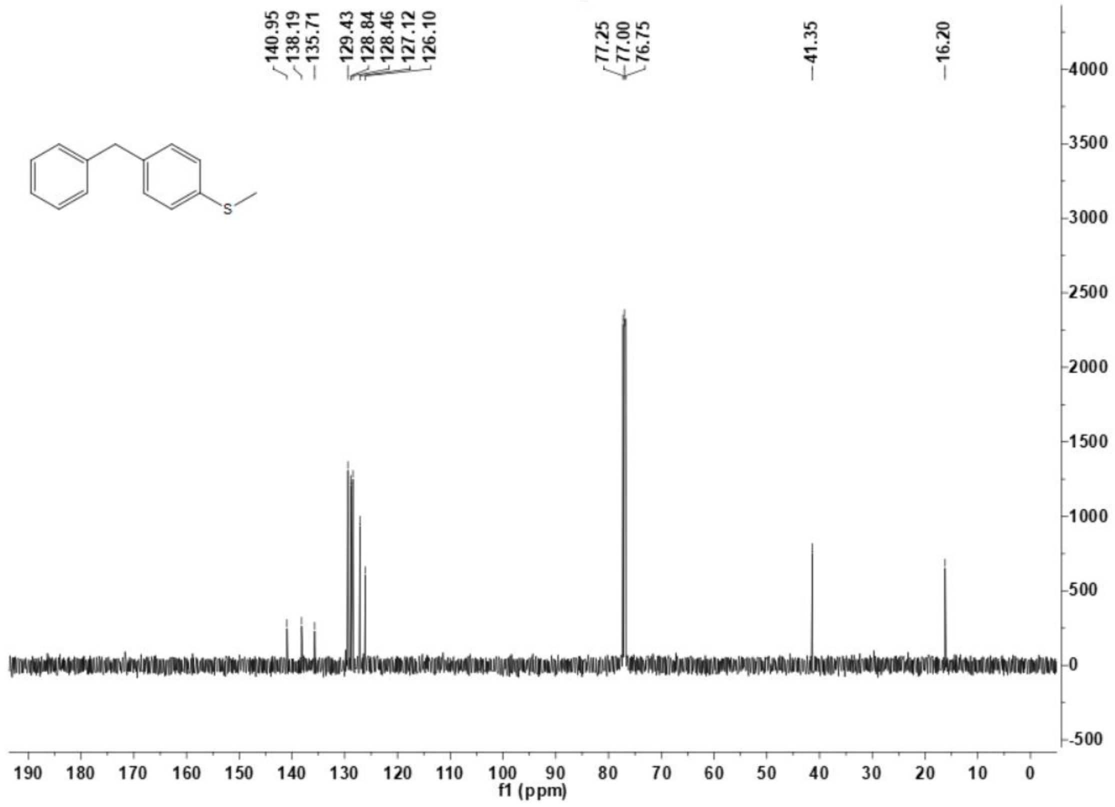

**9-(4-phenylbutyl) phenanthrene (3ga)**

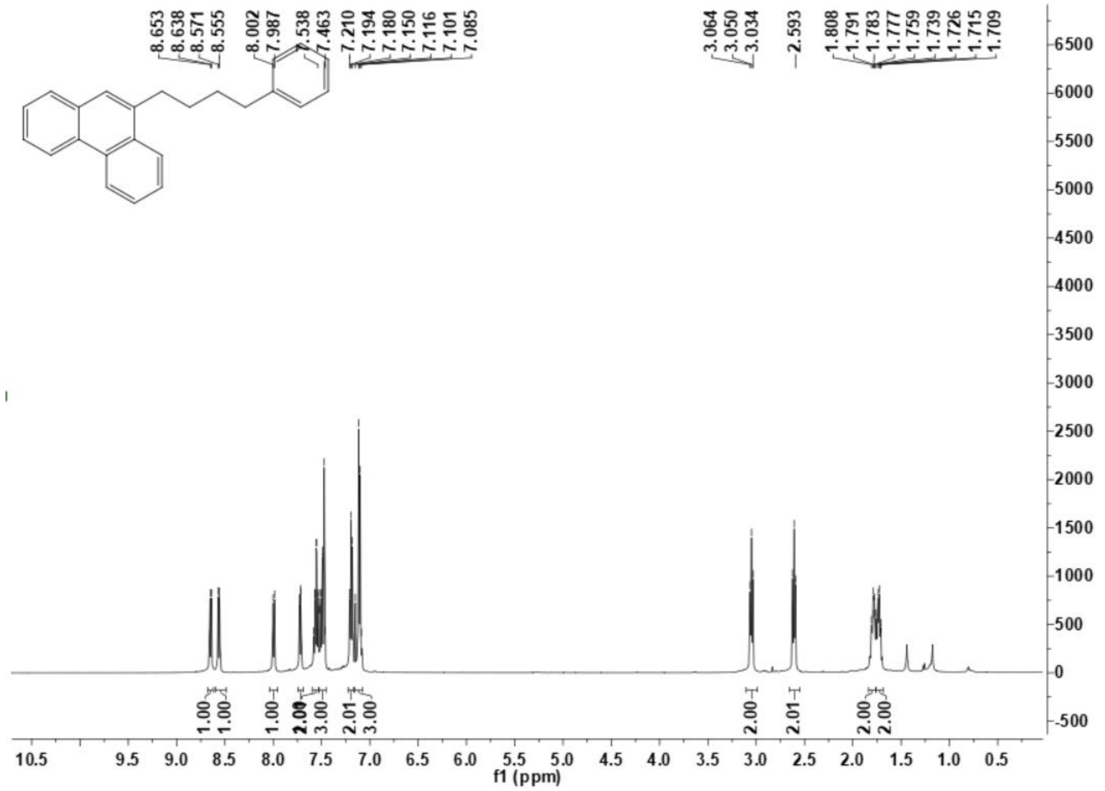


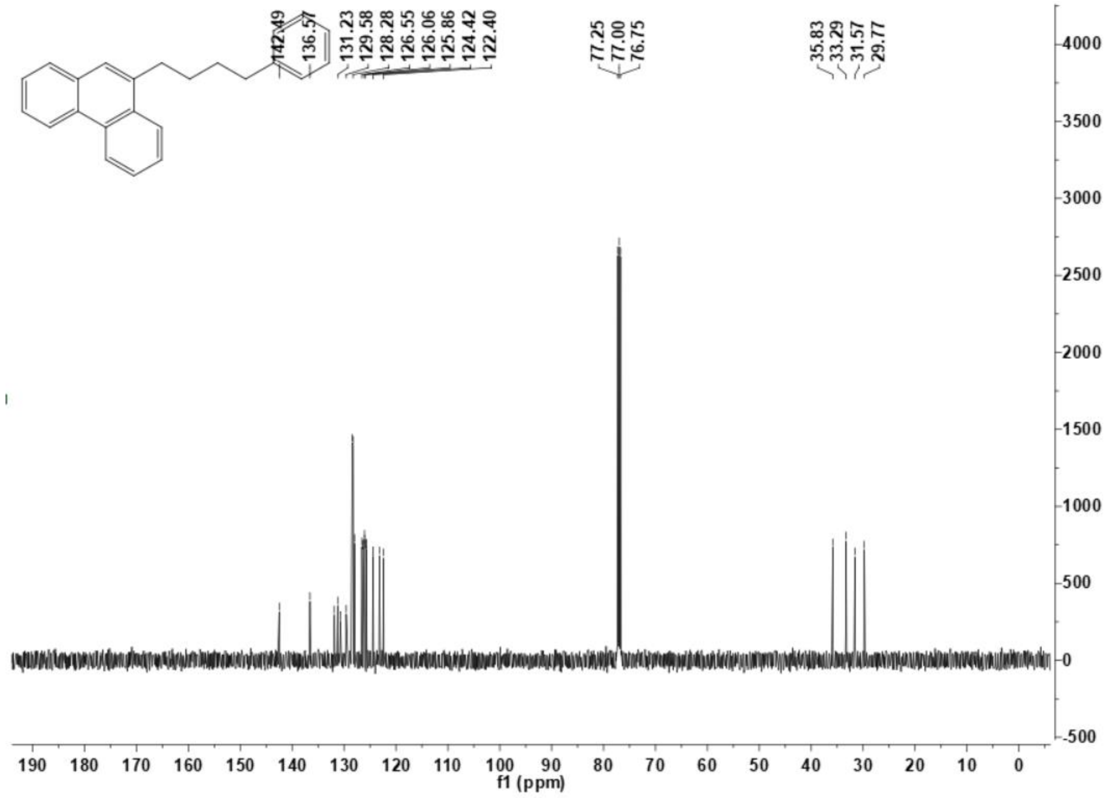

**4-(4-phenylbutyl)-1,1'-biphenyl (3ha)**

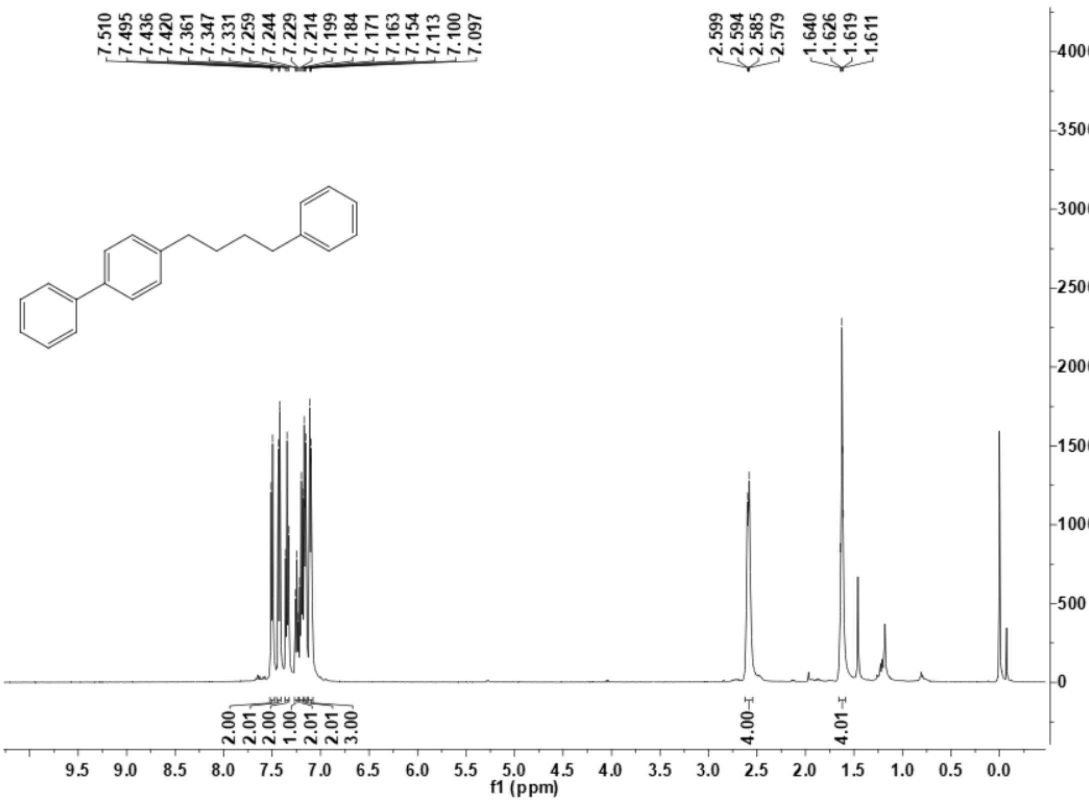


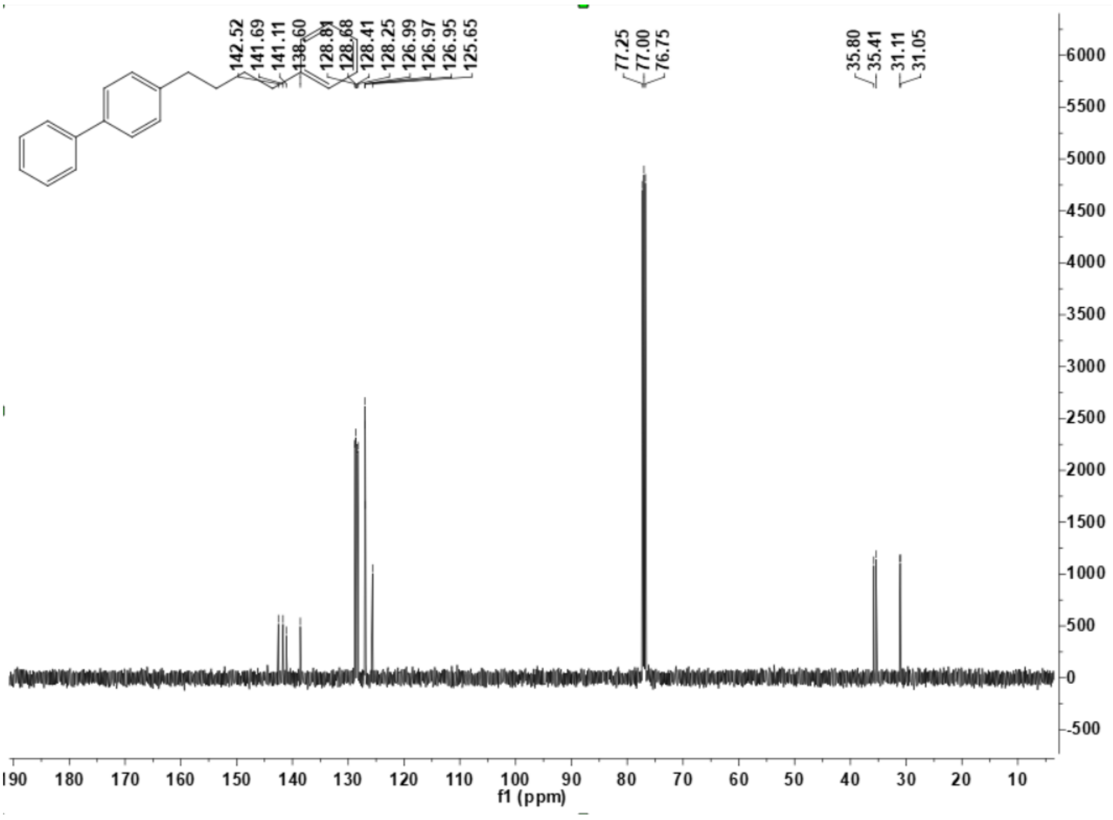

**1-methoxy-4-(4-phenylbutyl) benzene (3ia)**

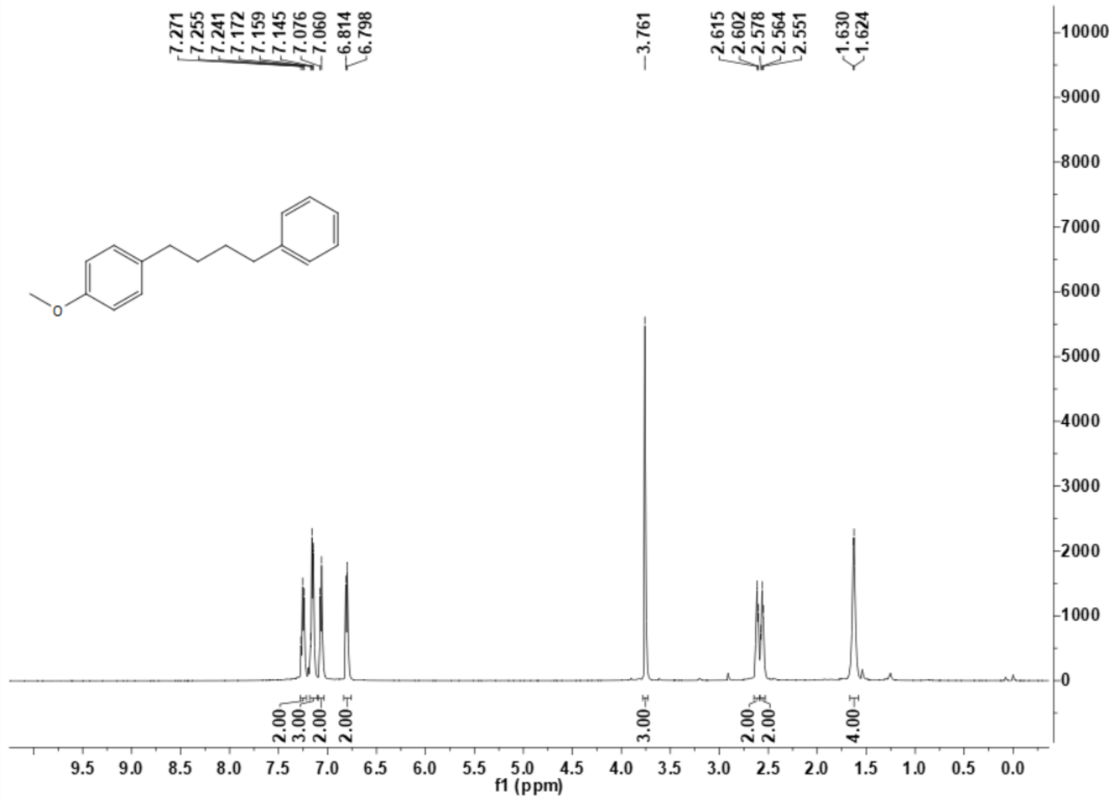


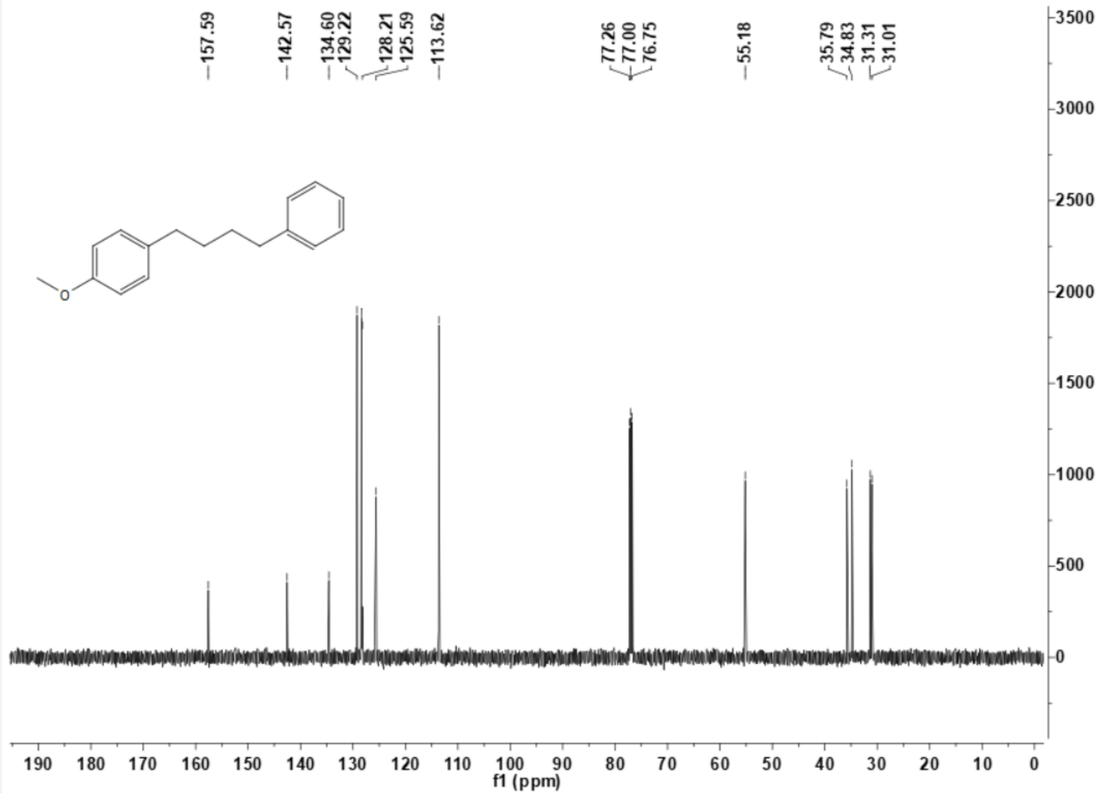

**1-(benzyloxy)-4-(4-phenylbutyl) benzene (3ja)**

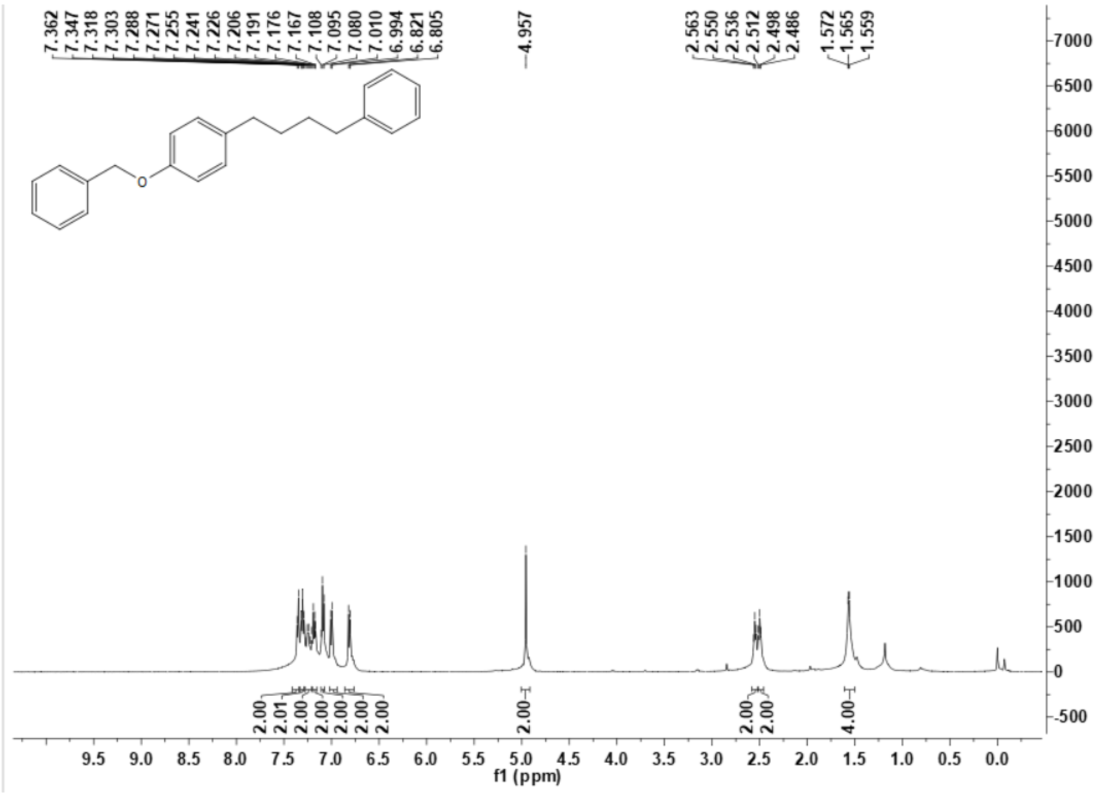


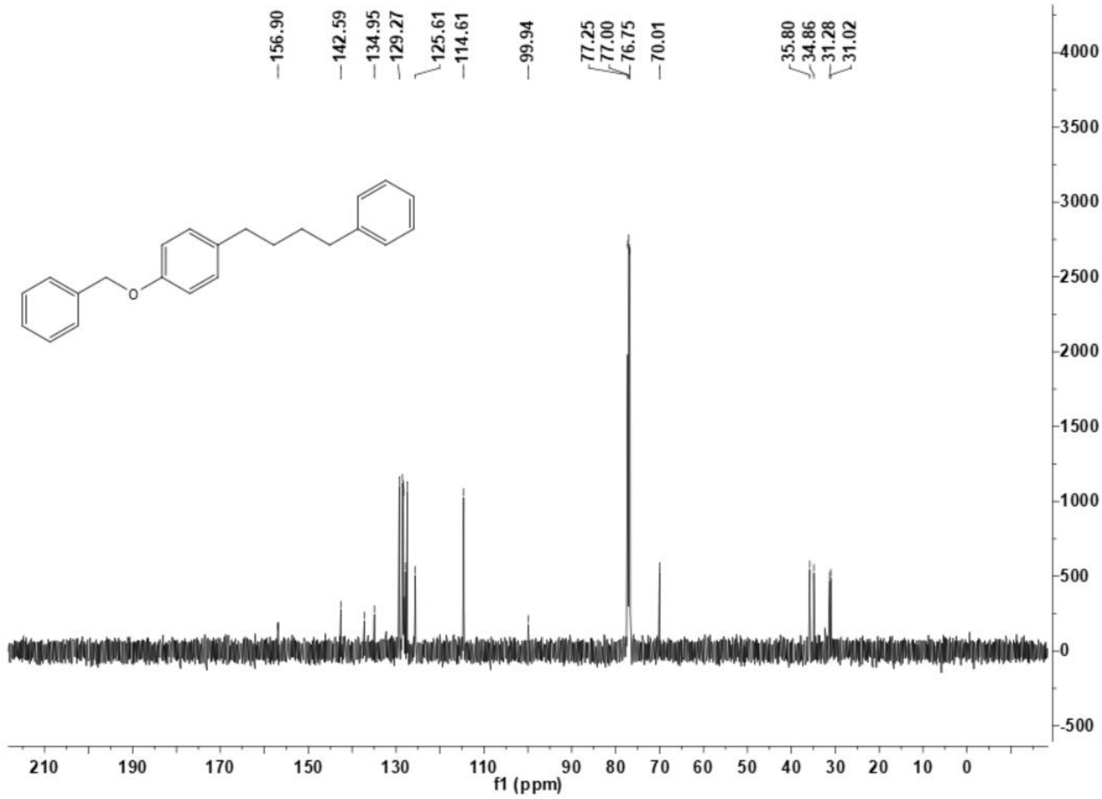

**4-(4-(4-phenylbutyl) phenyl) morpholine (3ka)**

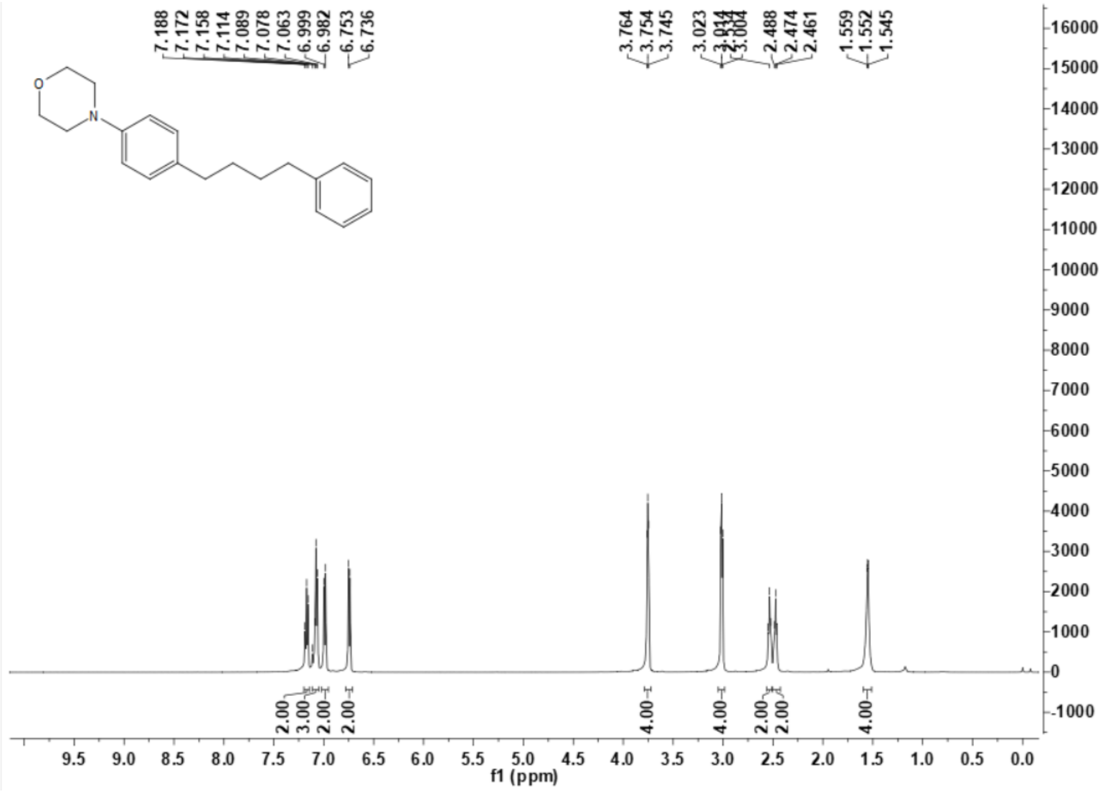

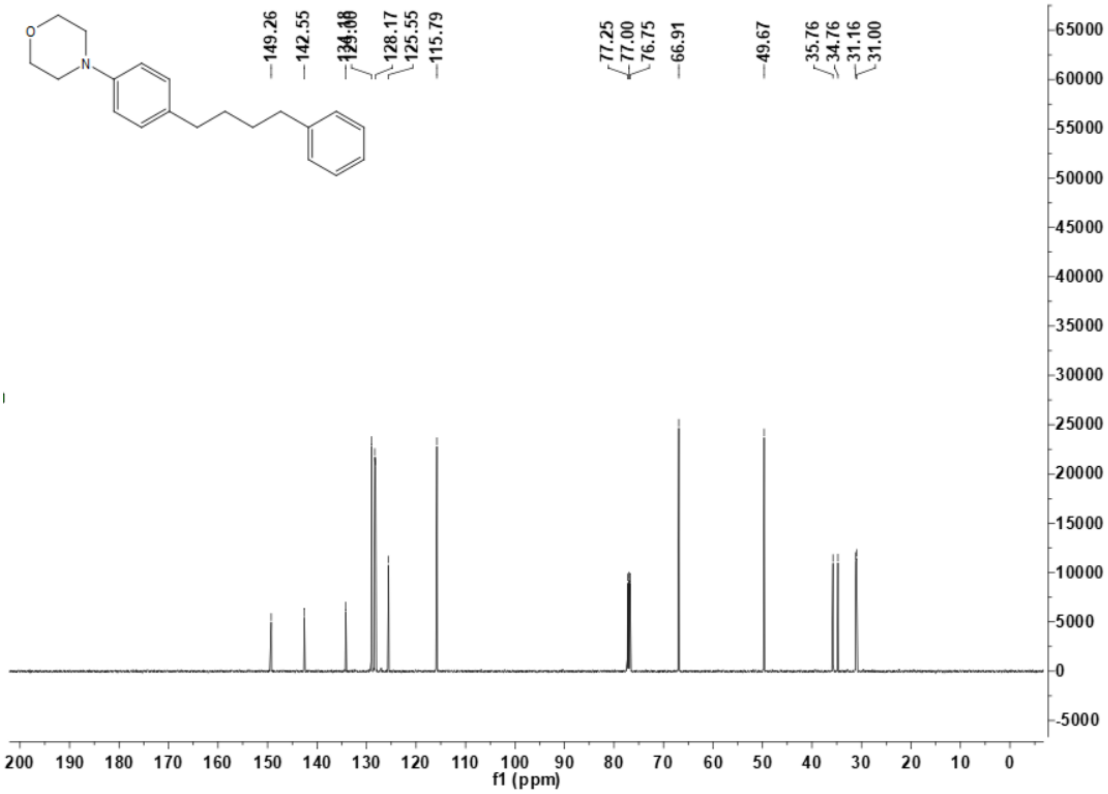

**1-methyl-2-(4-phenylbutyl) benzene (3la).**

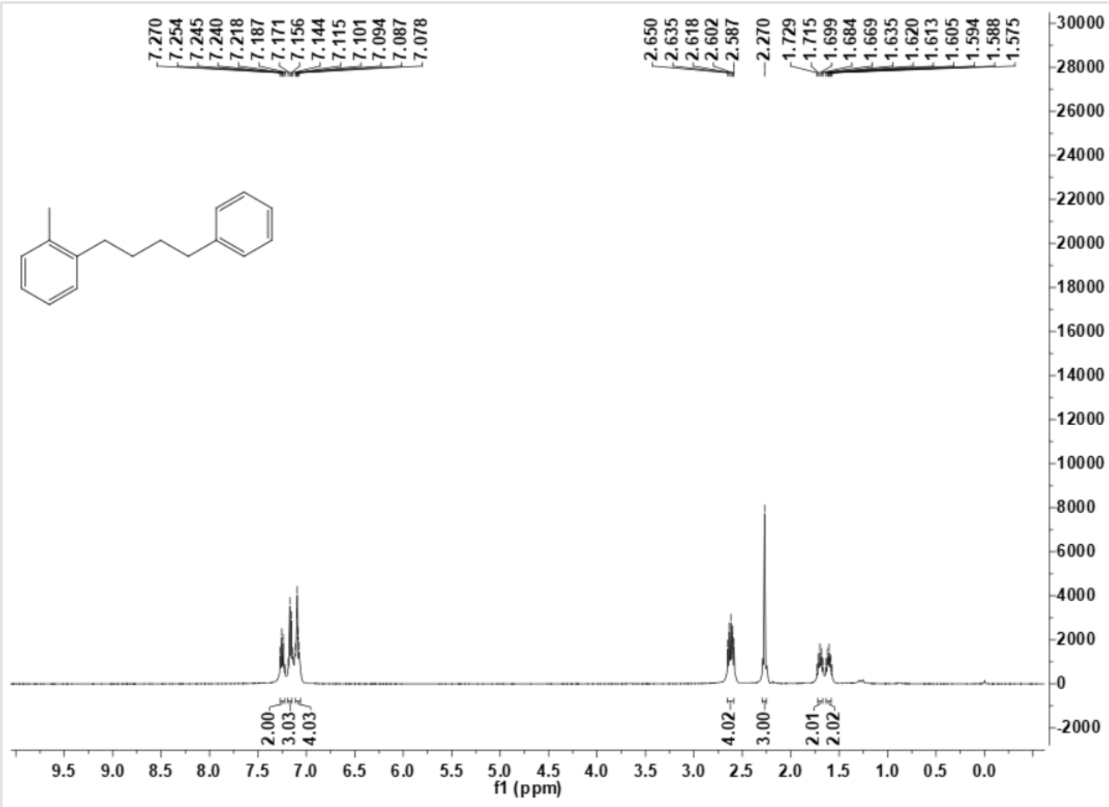


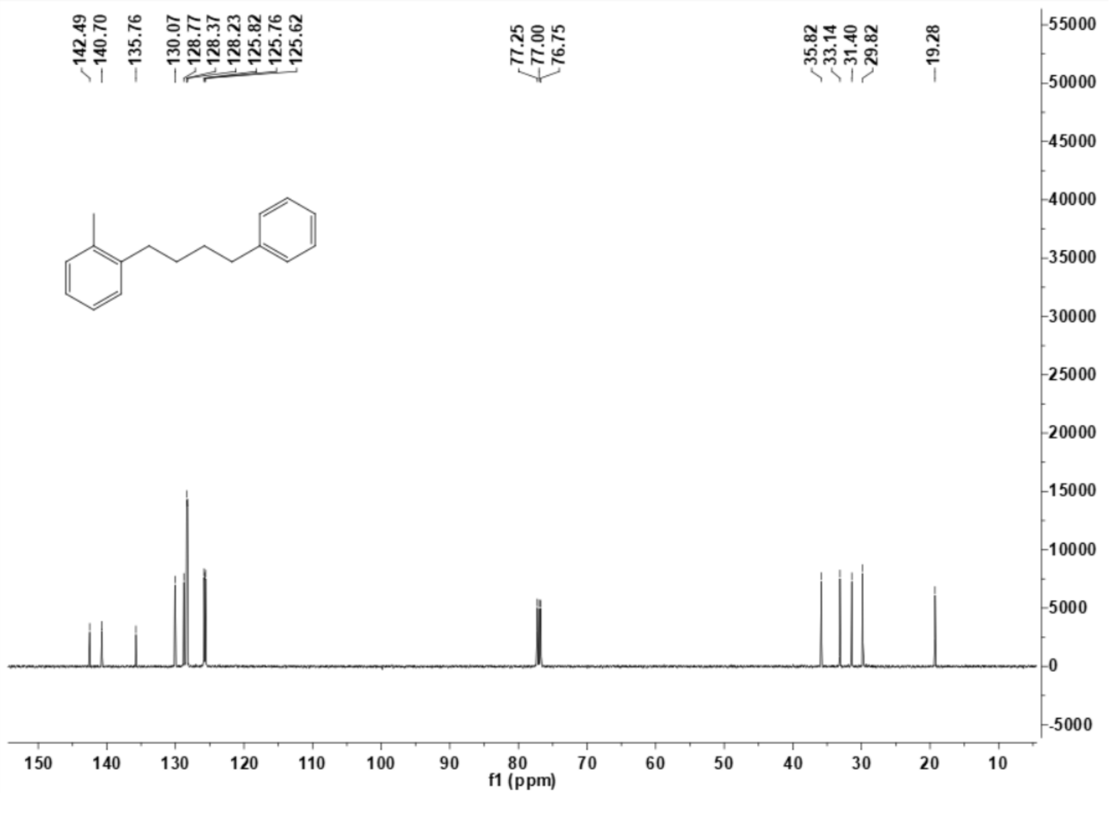

**1-bromo-2-(4-phenylbutyl) benzene (3ma)**

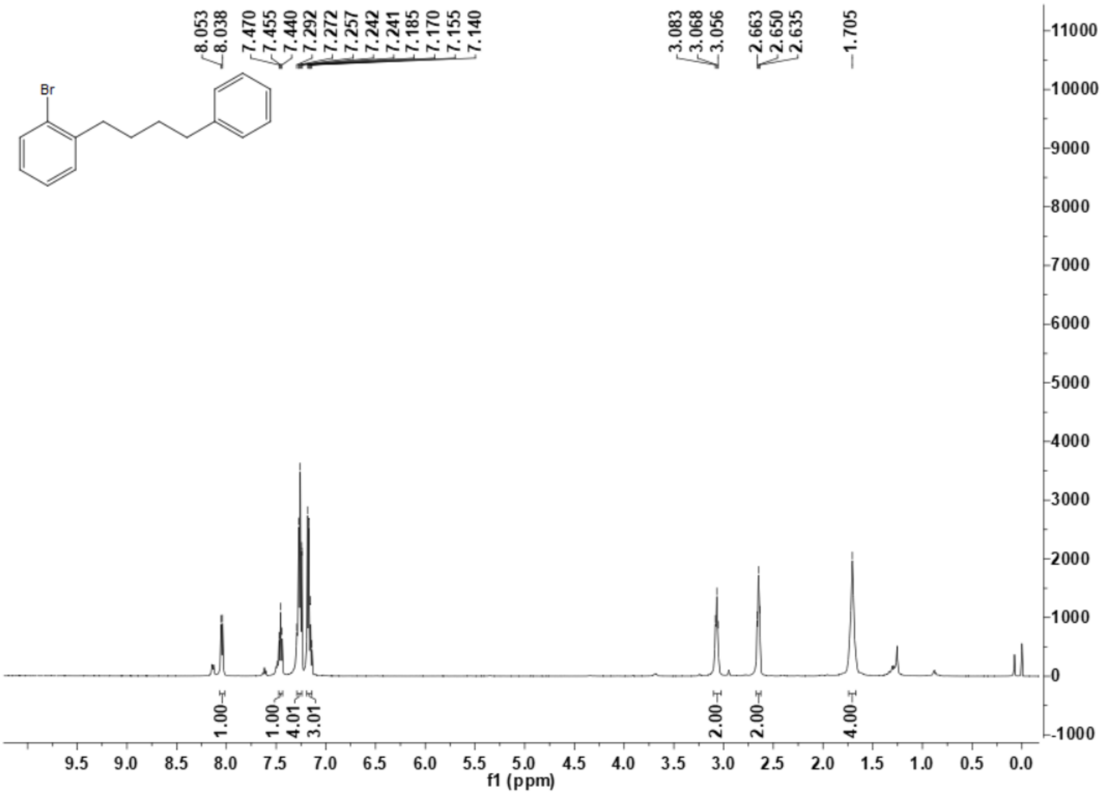


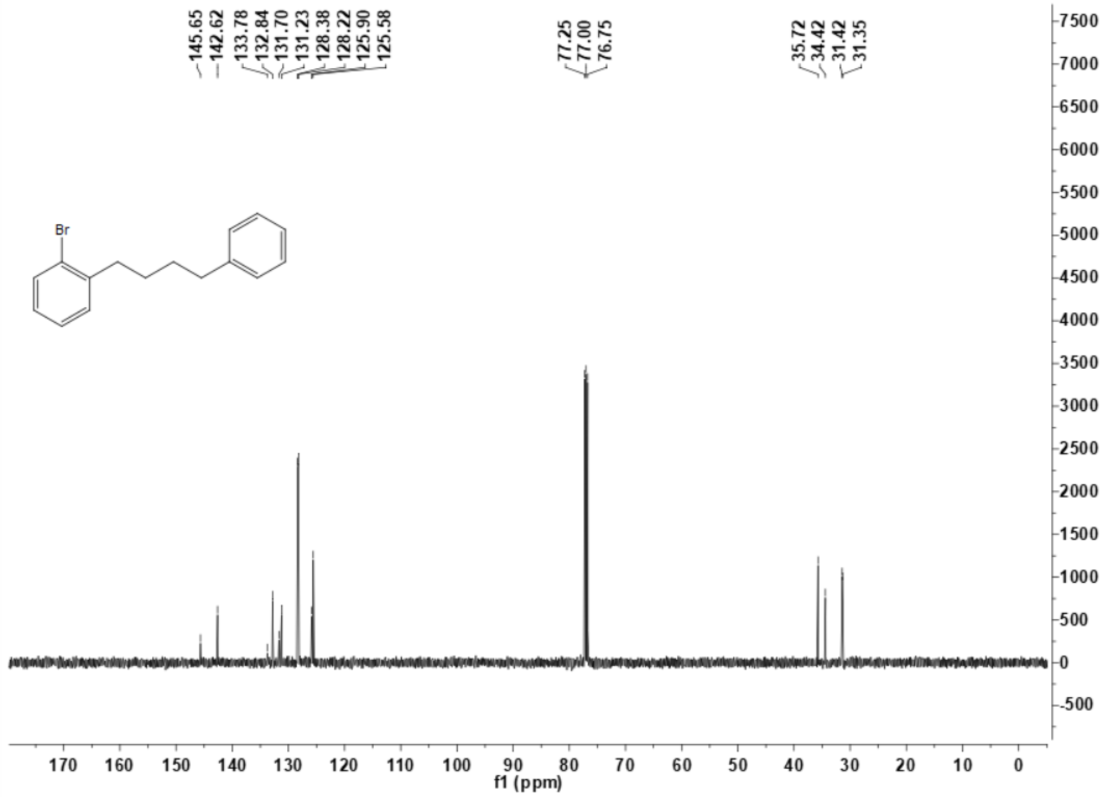

# 3-hydroxy-2-(4-phenylbutyl) benzaldehyde (3na)

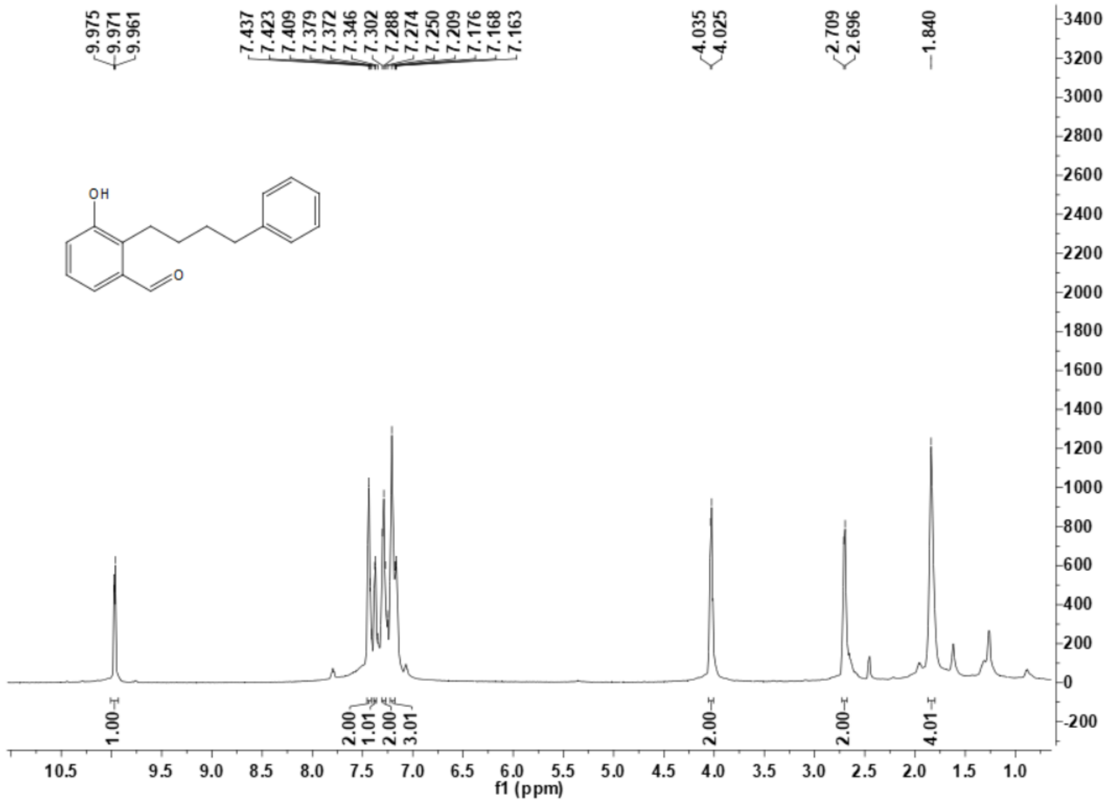


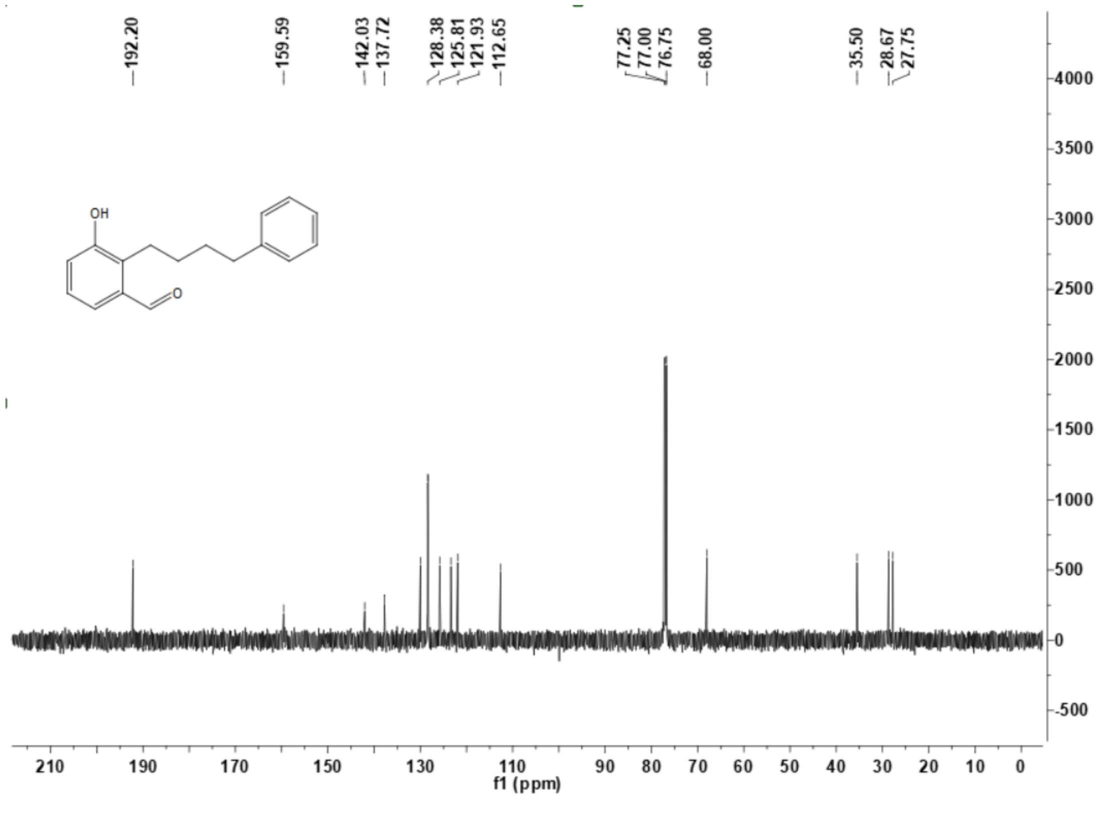

**Methyl 5-mesitylpentanoate (3oq)**

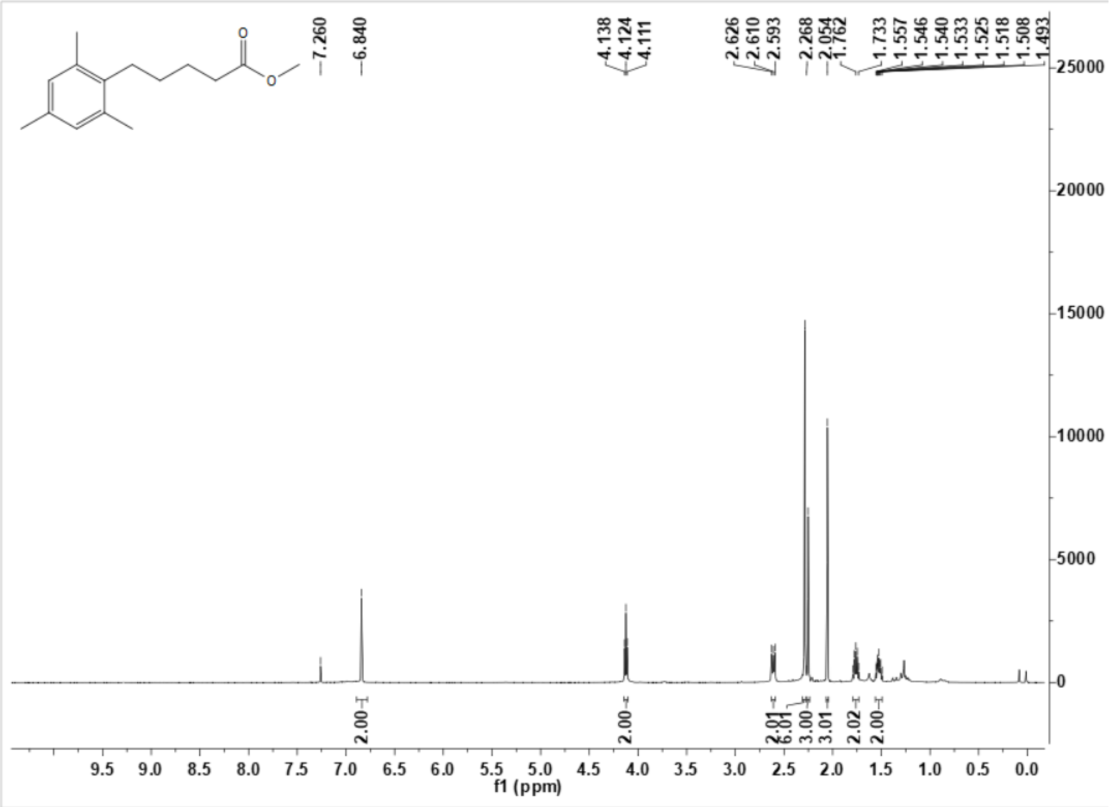


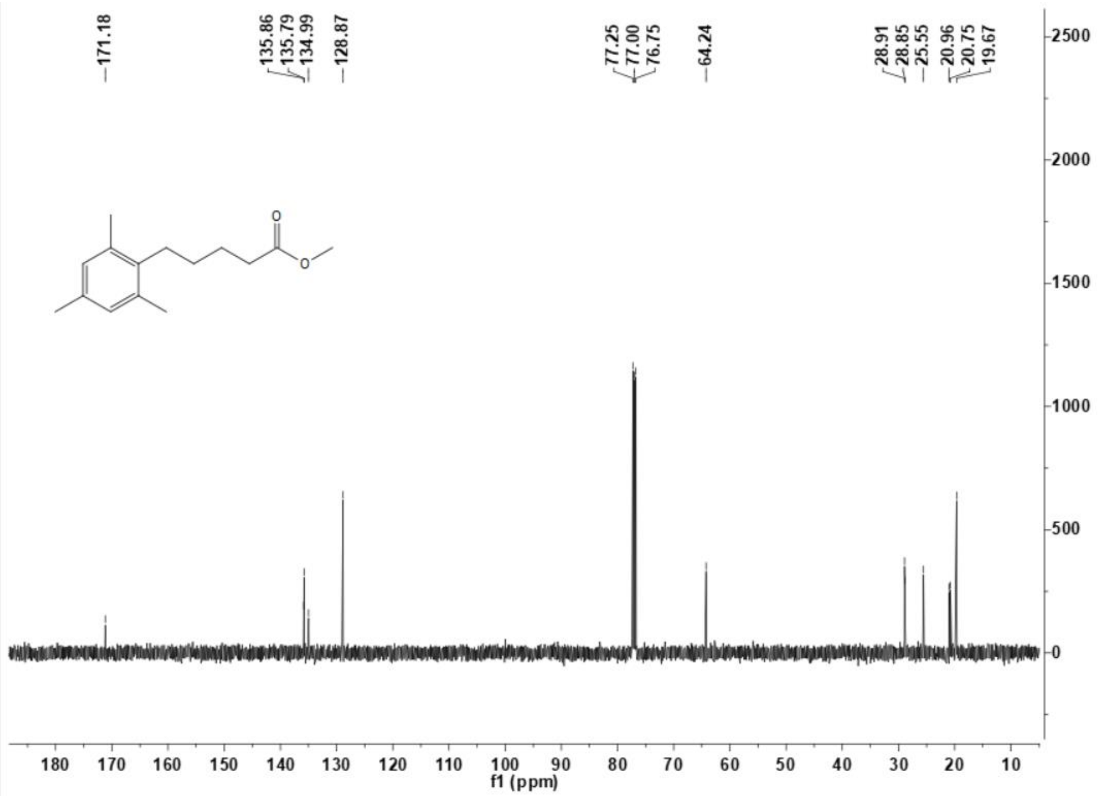

**1-(4-(4-phenylbutyl) phenyl) ethan-1-one (3pa)**

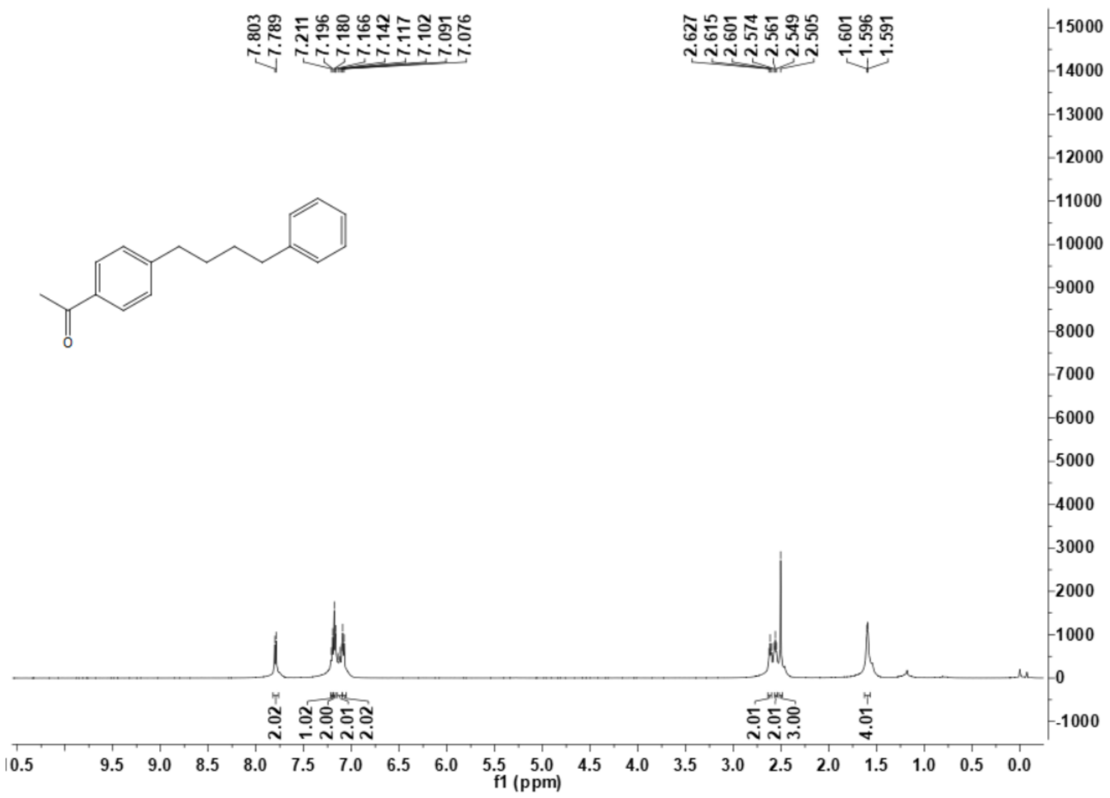


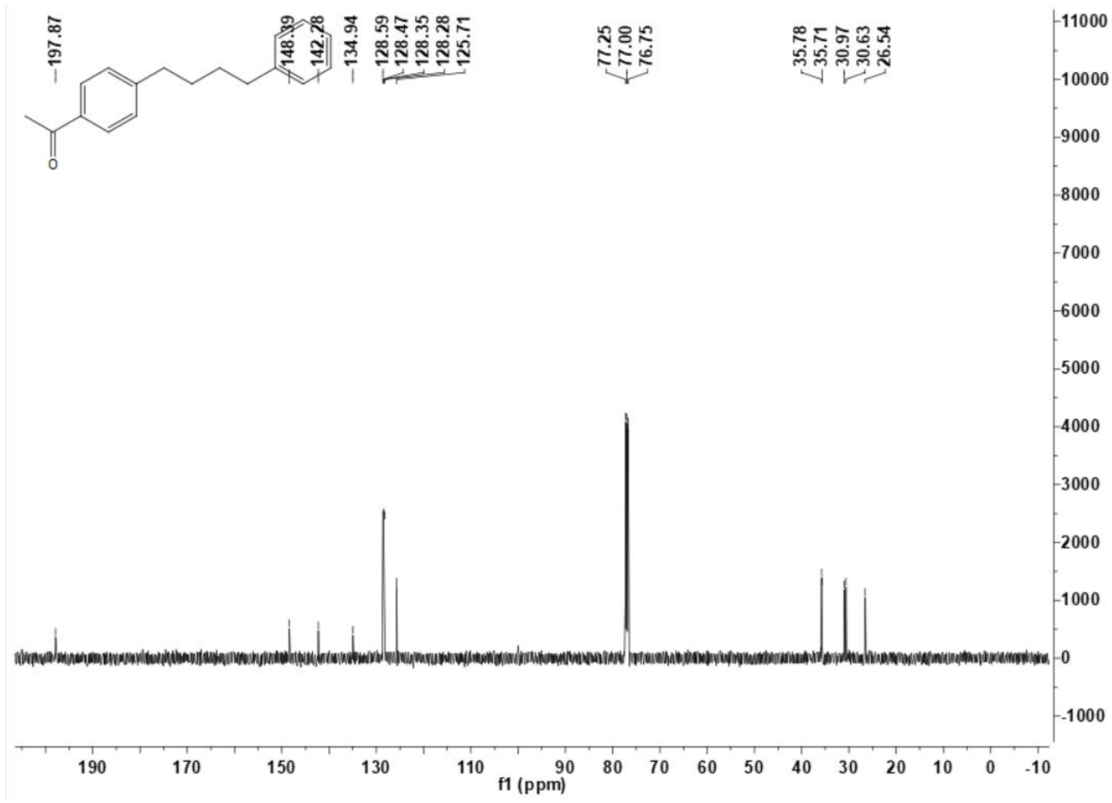

**4-(4-phenylbutyl) benzonitrile (3qa)**

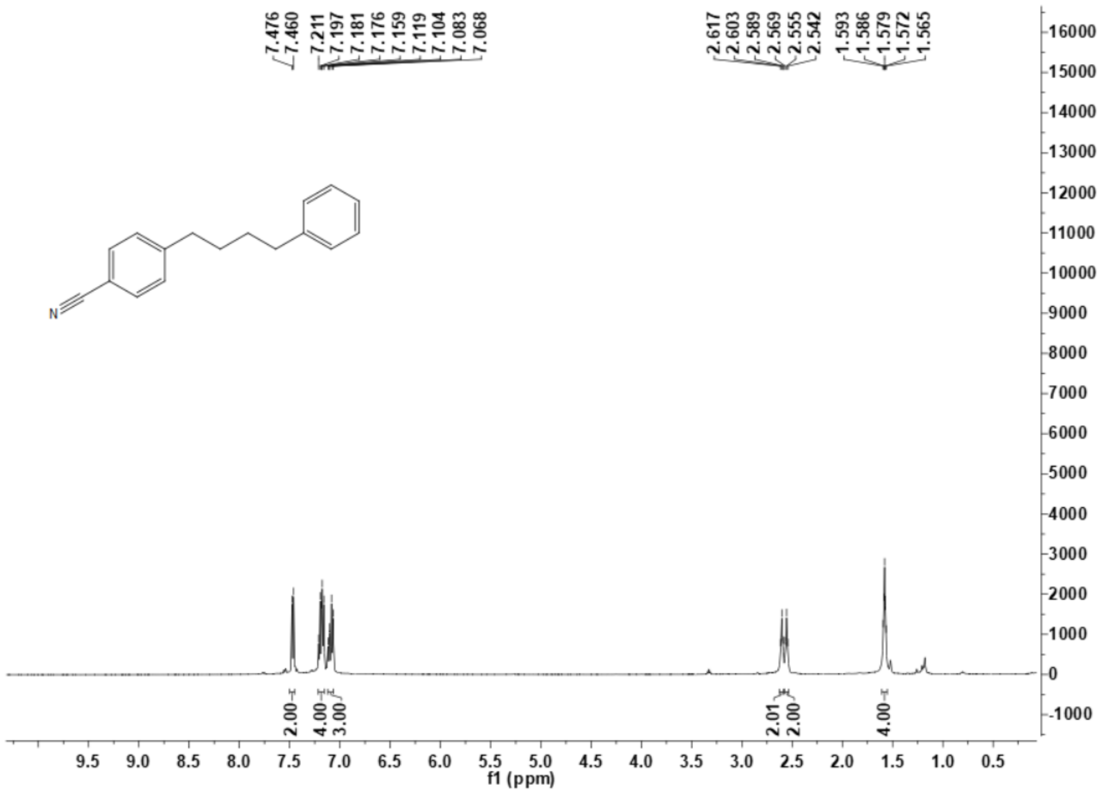


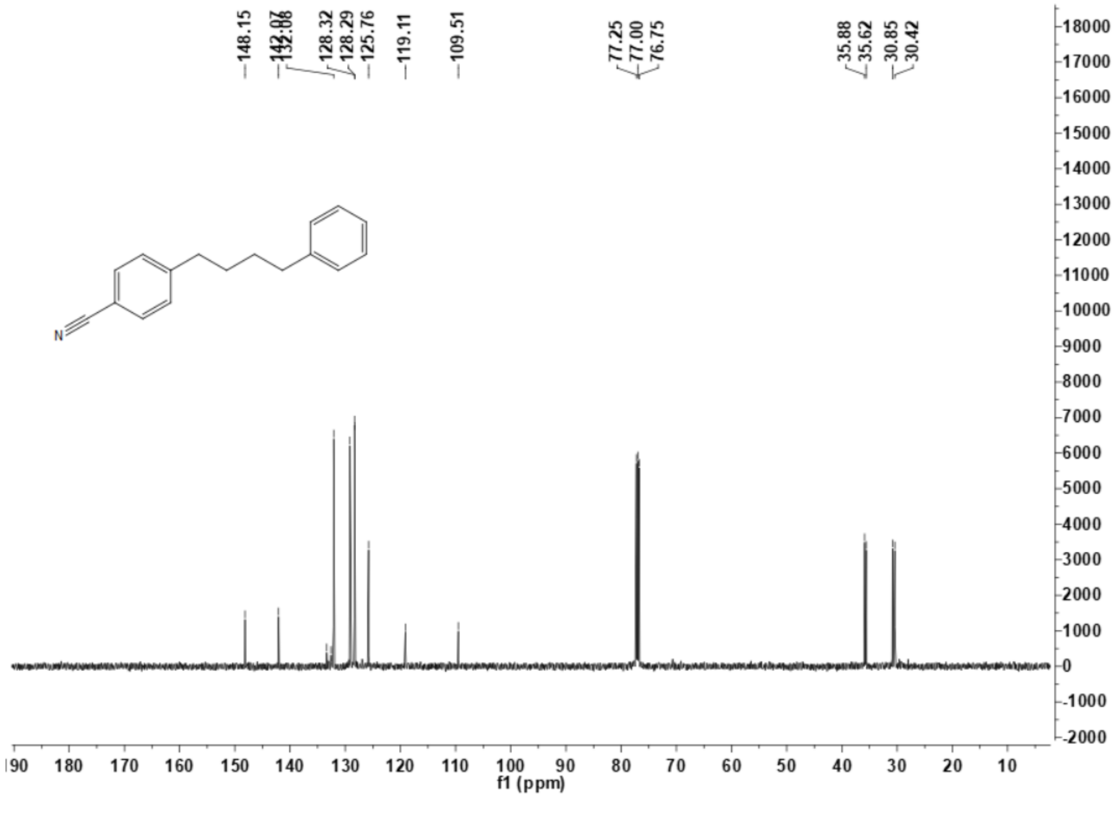

**4-(4-phenylbutyl) benzenethiol (3ra)**

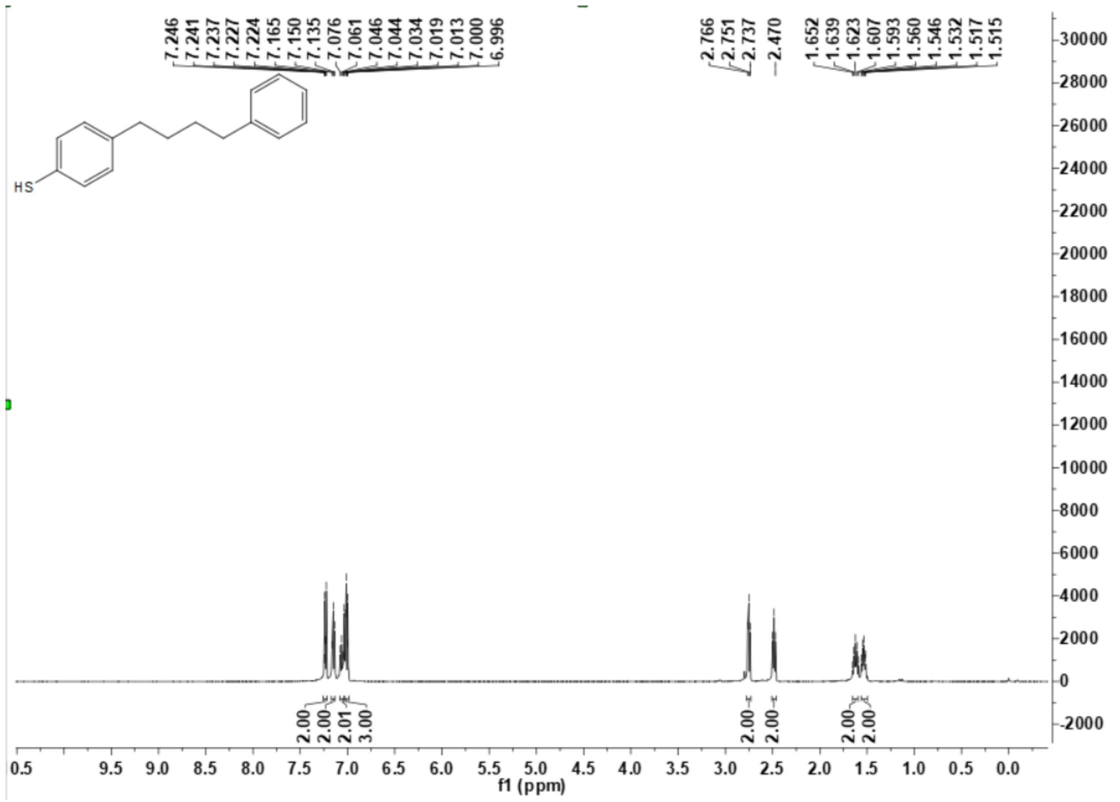

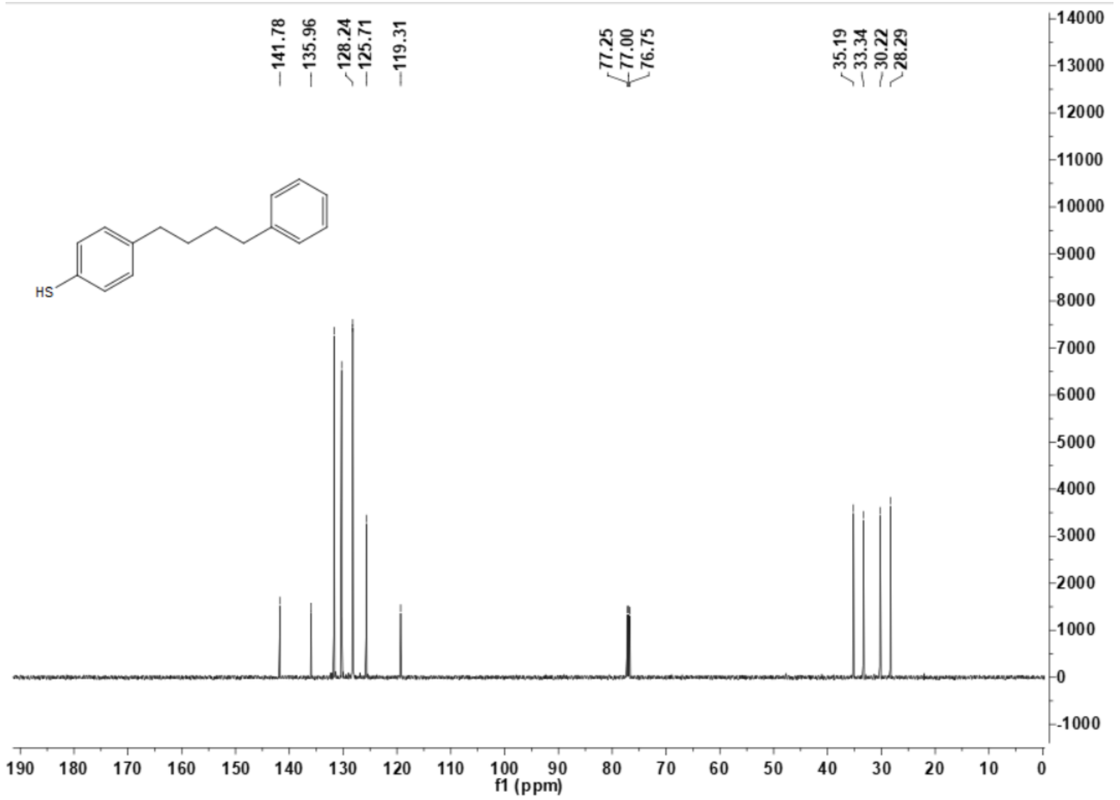

**Trimethyl(4-(4-phenylbutyl) phenyl) silane1 (3sa)**

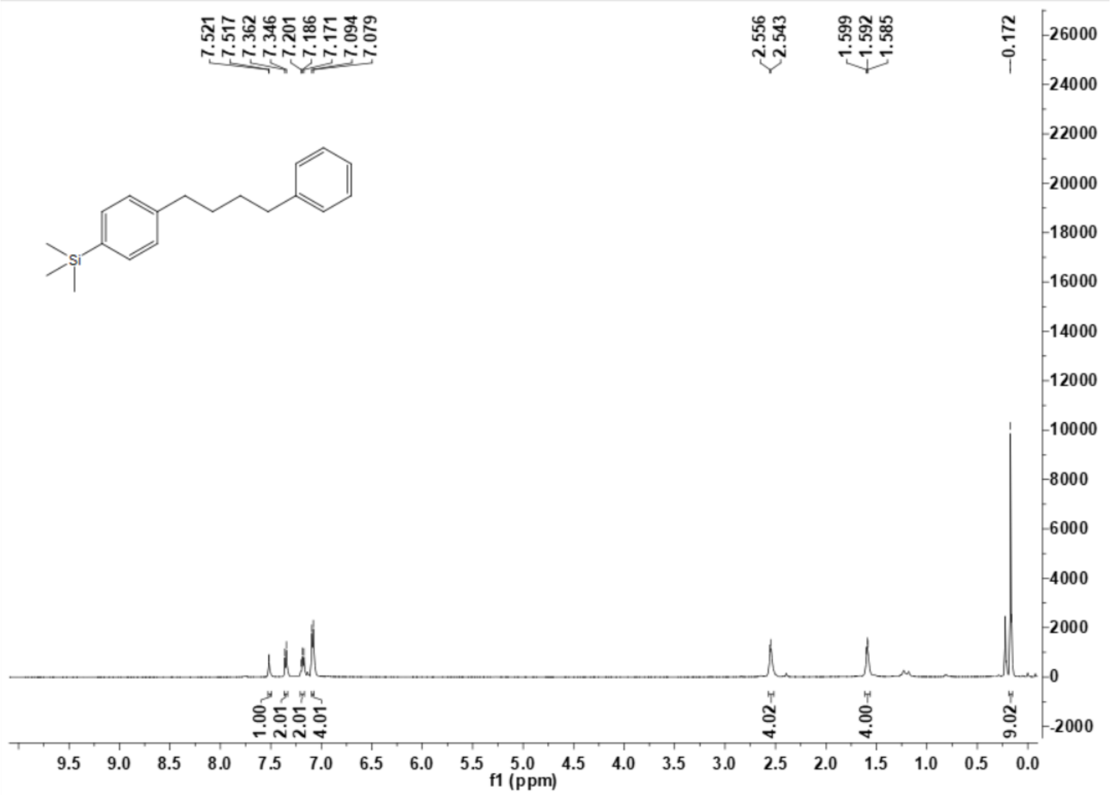


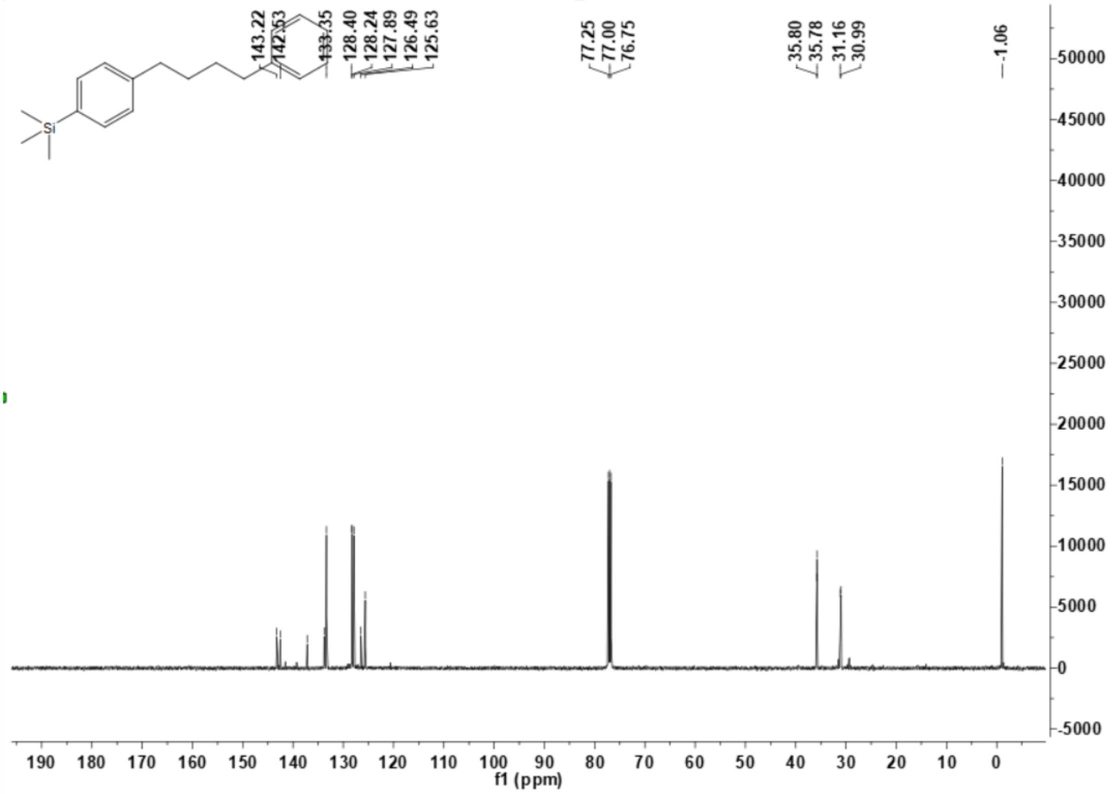

## 4,4,5,5-tetramethyl-2-(4-(4-phenylbutyl) phenyl)-1,3,2-dioxaborolane (3ta)

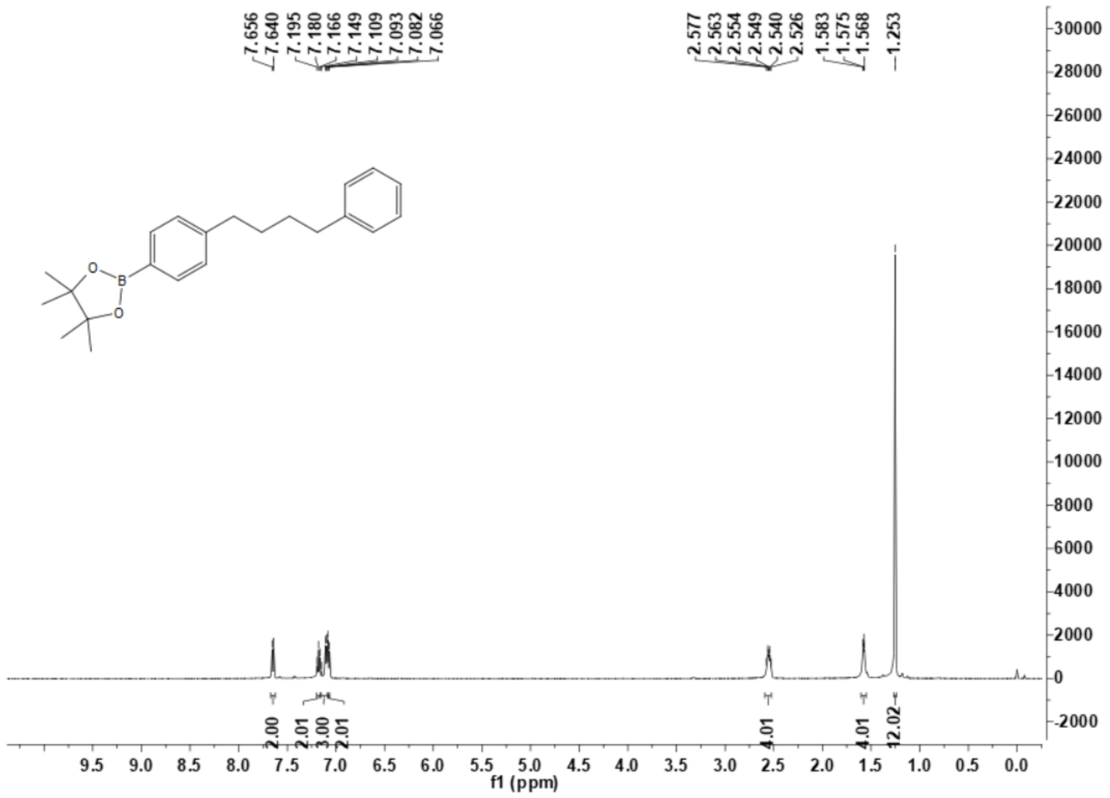


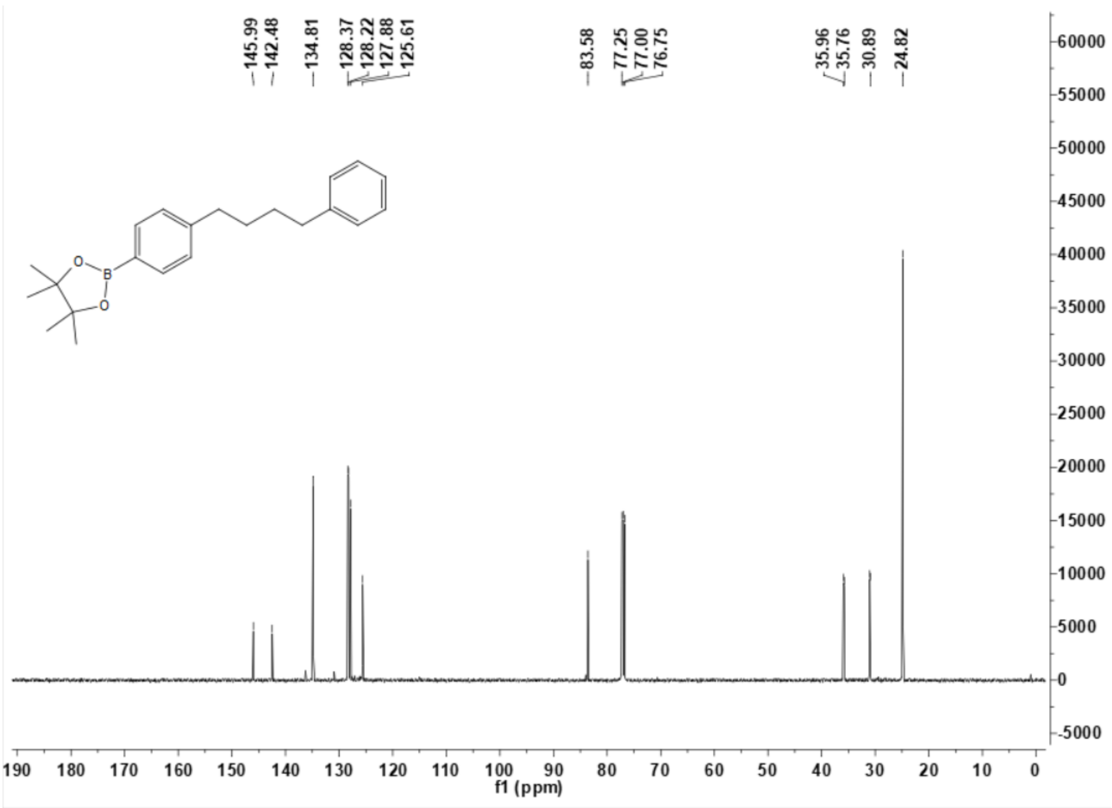

**2-(4-phenylbutyl) dibenzo[*b*,*d*]furan (3ua)**

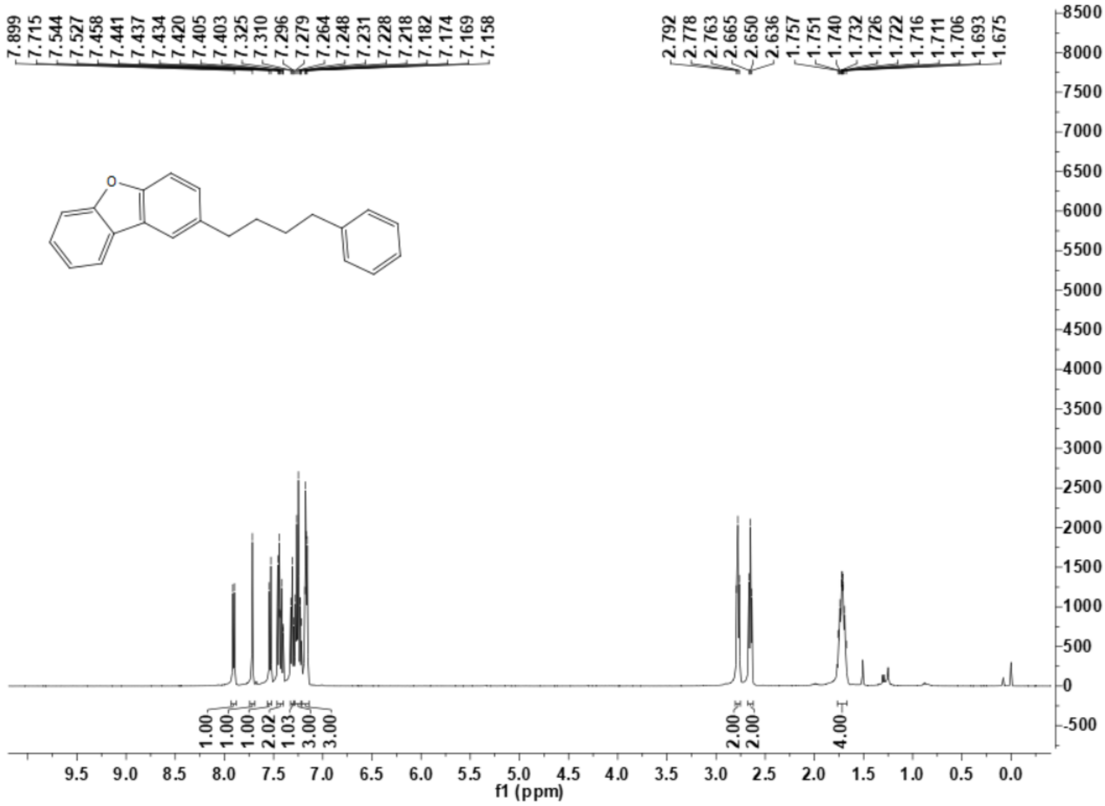


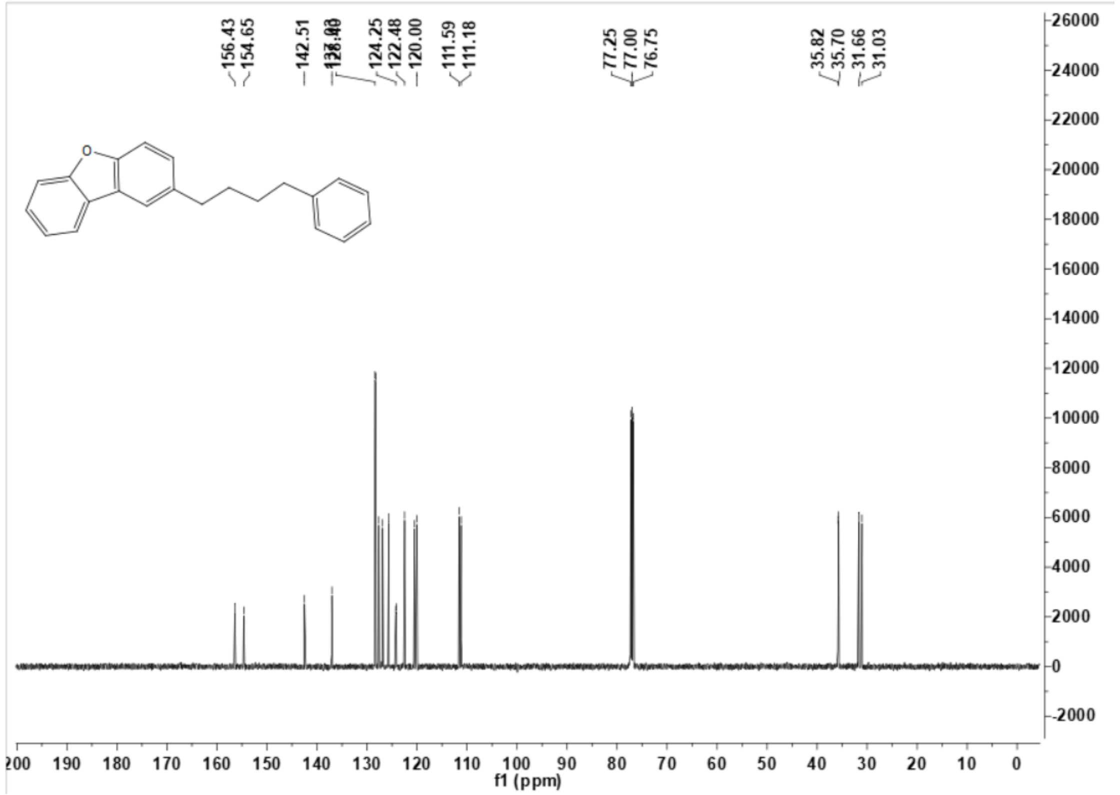

**5-(4-phenylbutyl) benzo[*b*]thiophene (3va)**

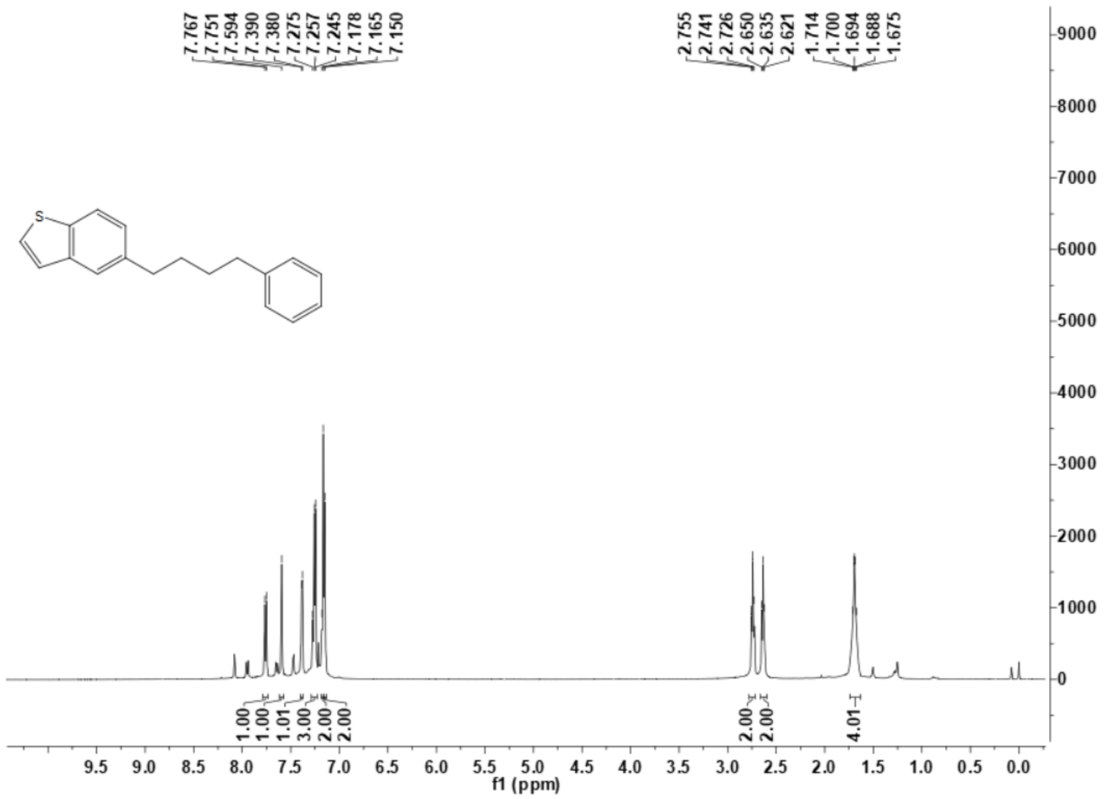


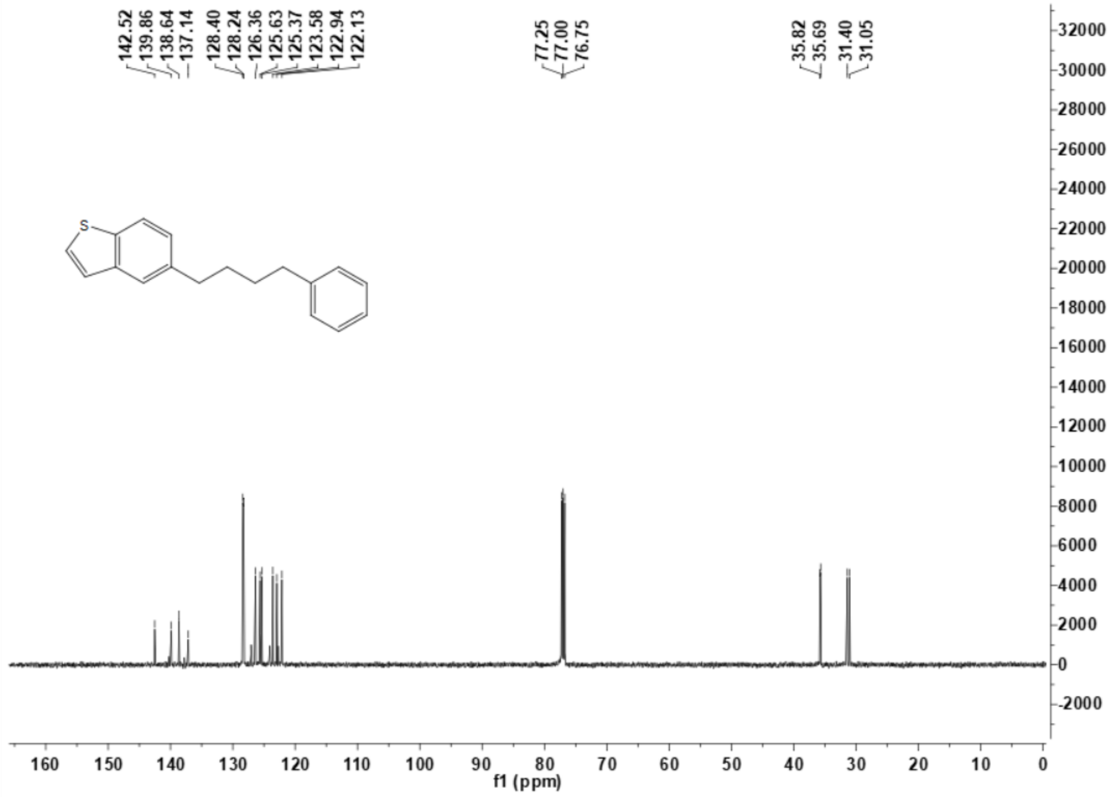

**1-methyl-5-(4-phenylbutyl)-1*H*-indole (3wa)**

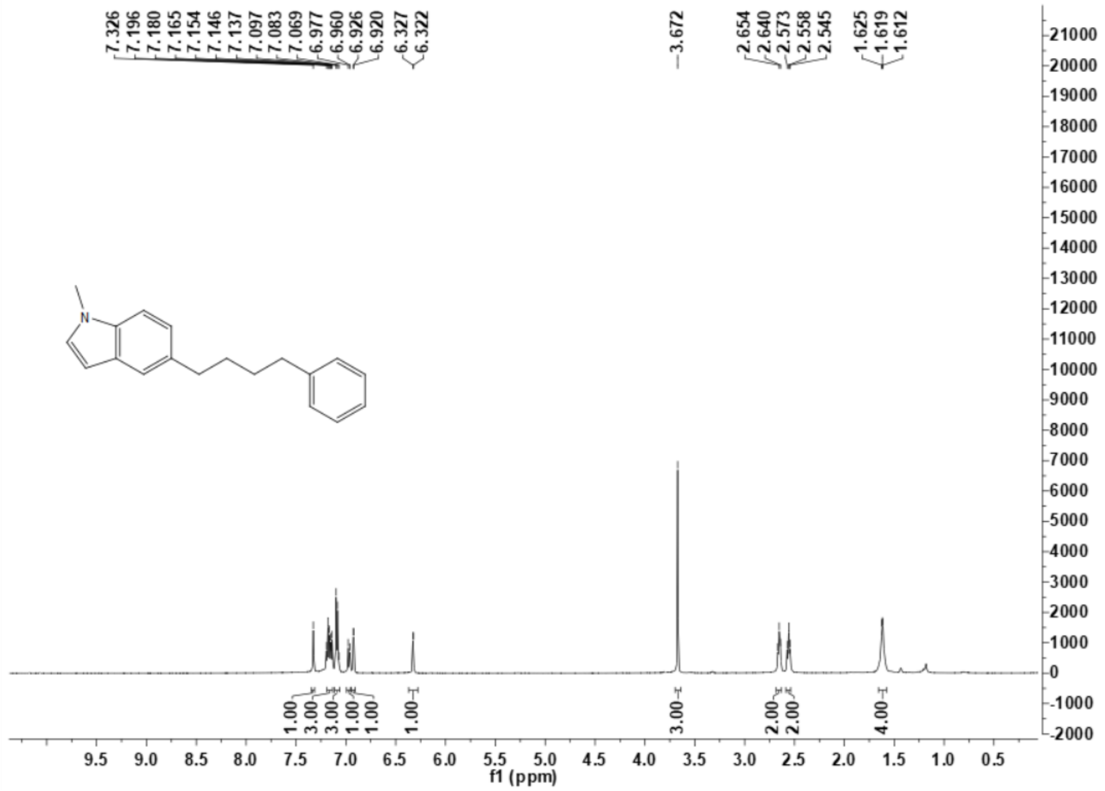


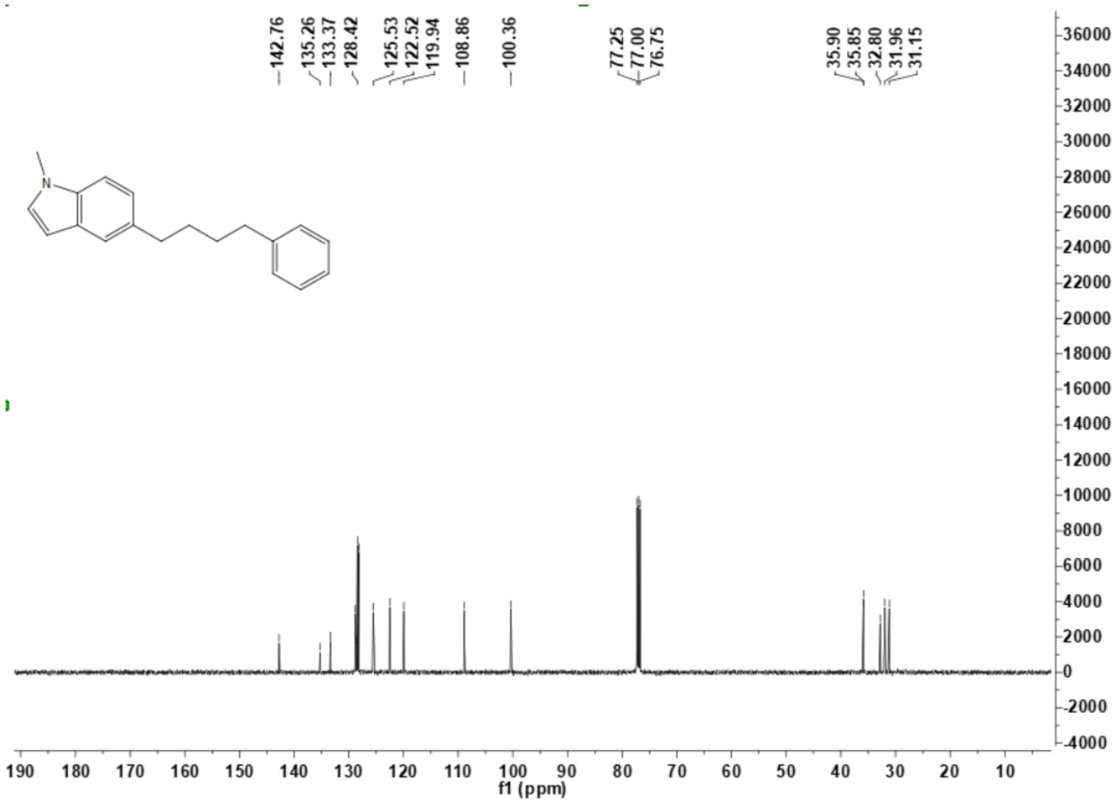

**9-phenyl-1-(4-phenylbutyl)-9*H*-carbazole (3xa)**

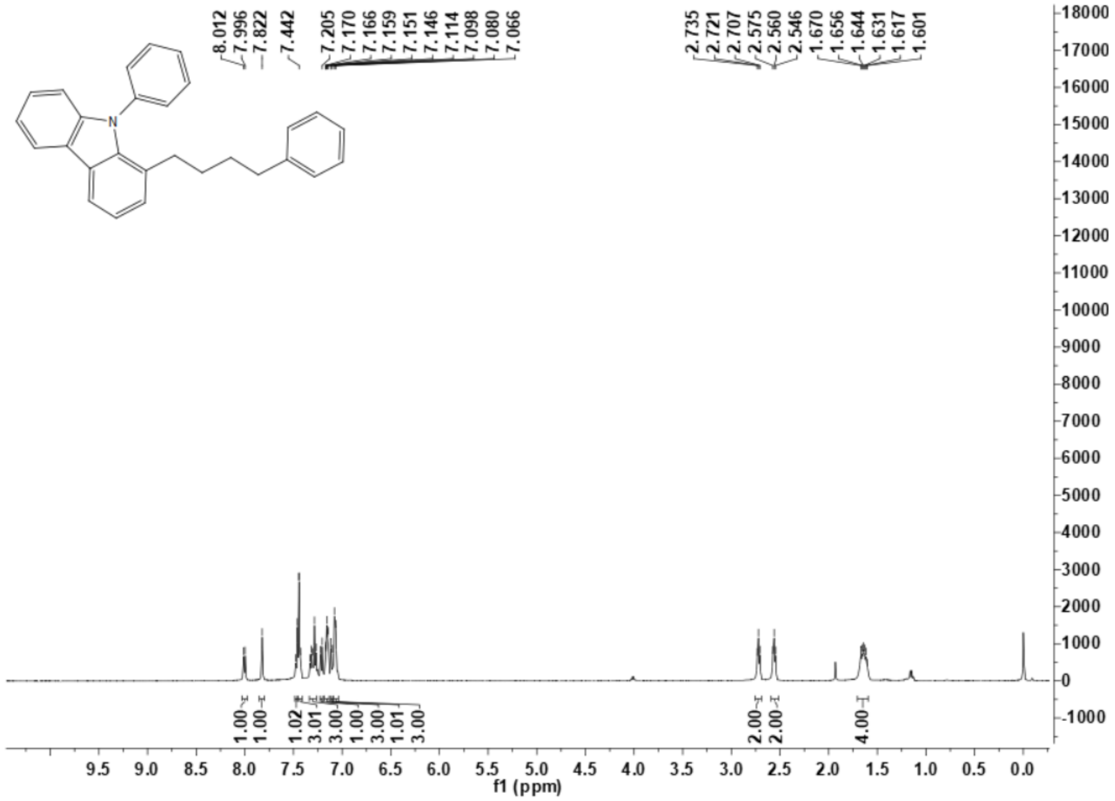

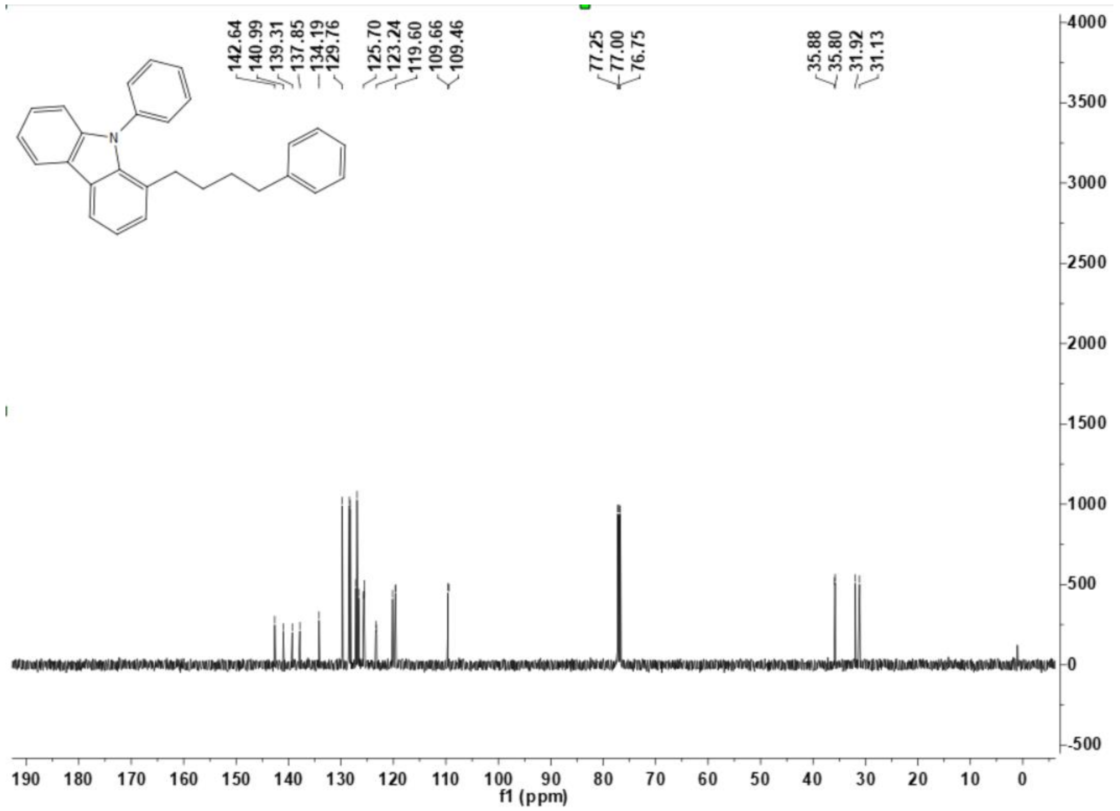

**2-(4-phenylbutyl) pyridine (3ya)**

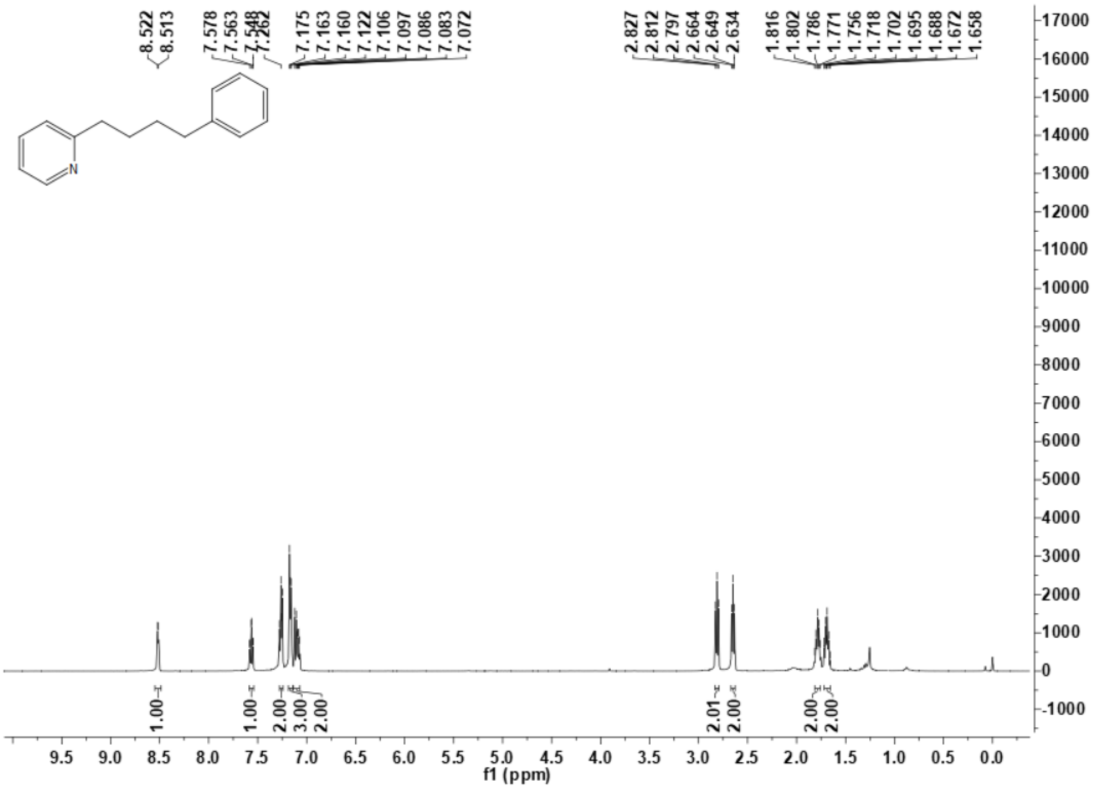


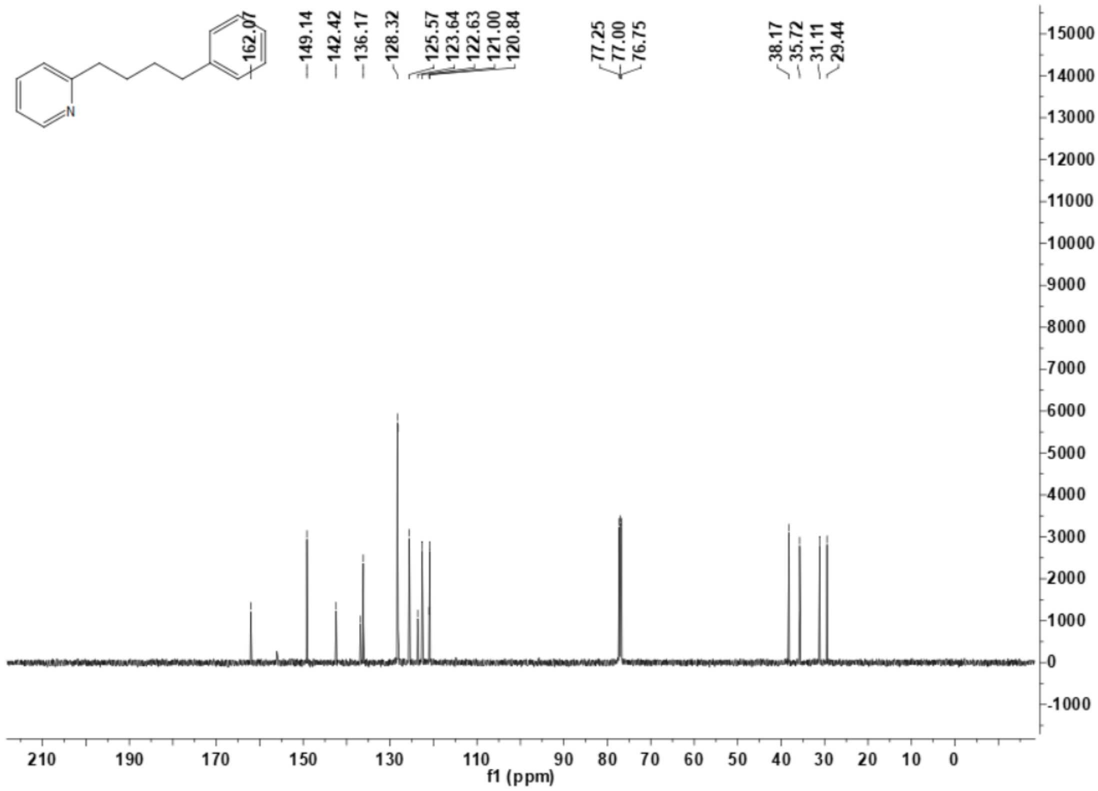

**2-(4-phenylbutyl)-6-(1*H*-pyrazol-1-yl)pyridine (3za)**

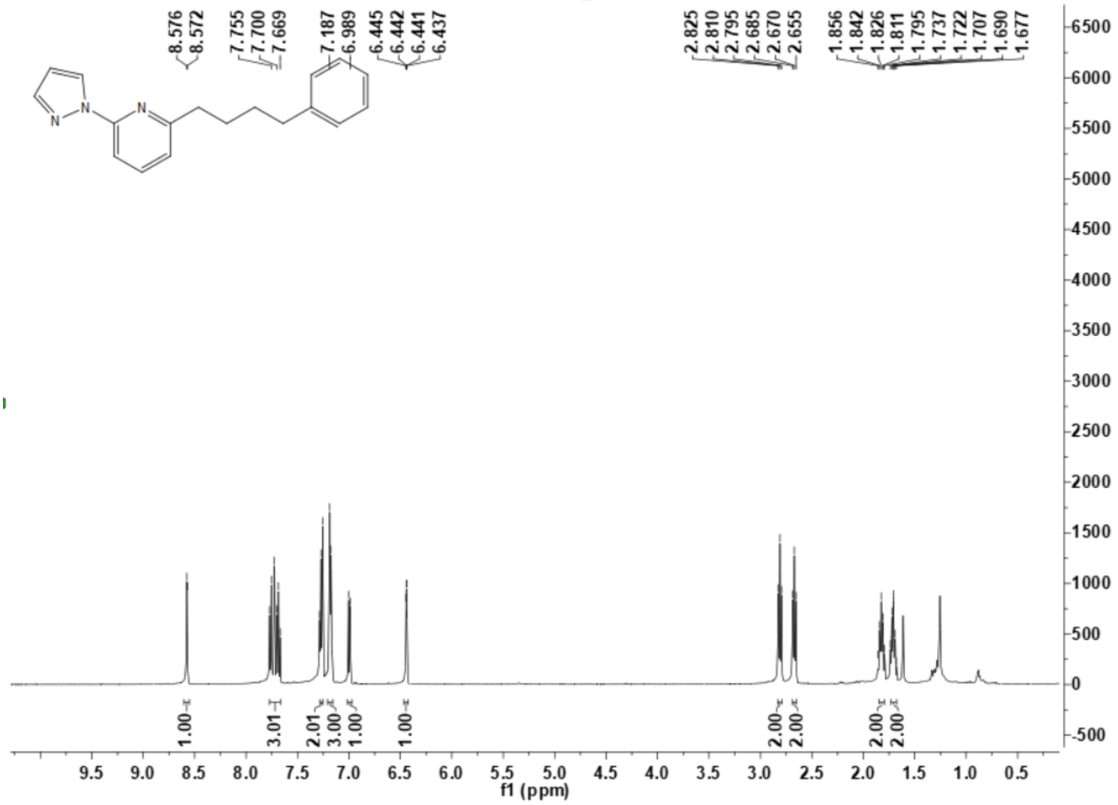

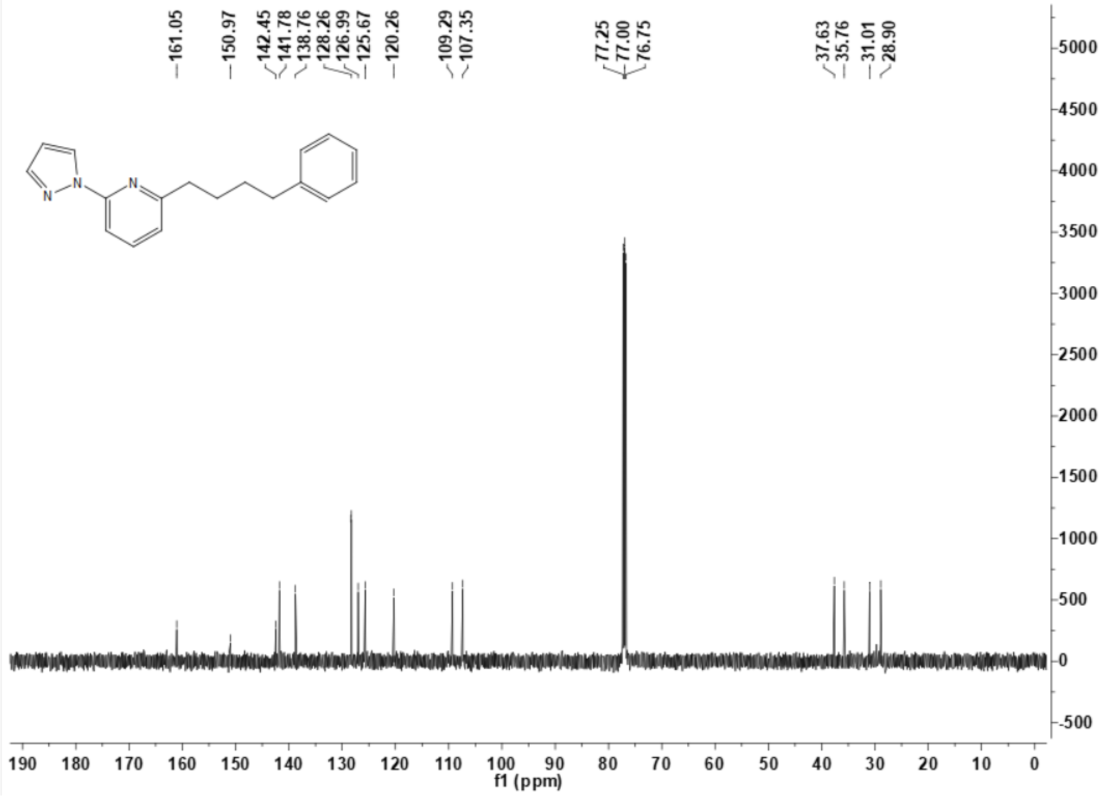

## 4-methyl-*N*,*N*-dipropylbenzenesulfonamide (6ab)

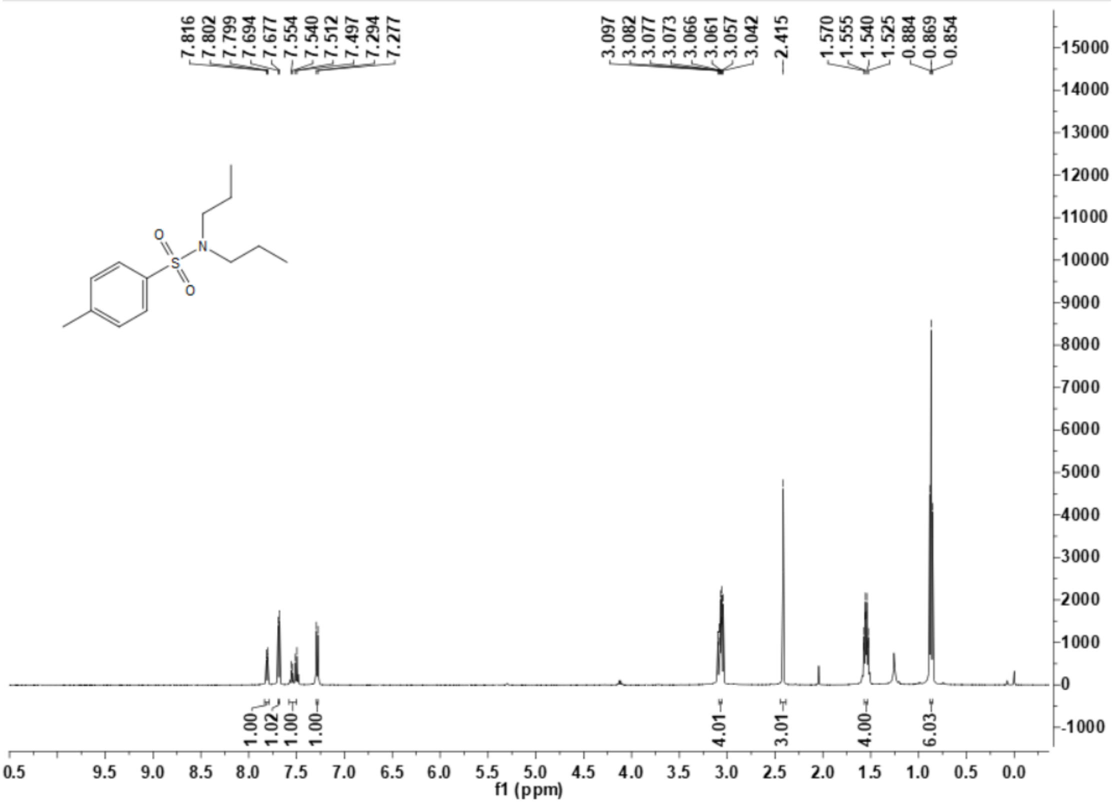


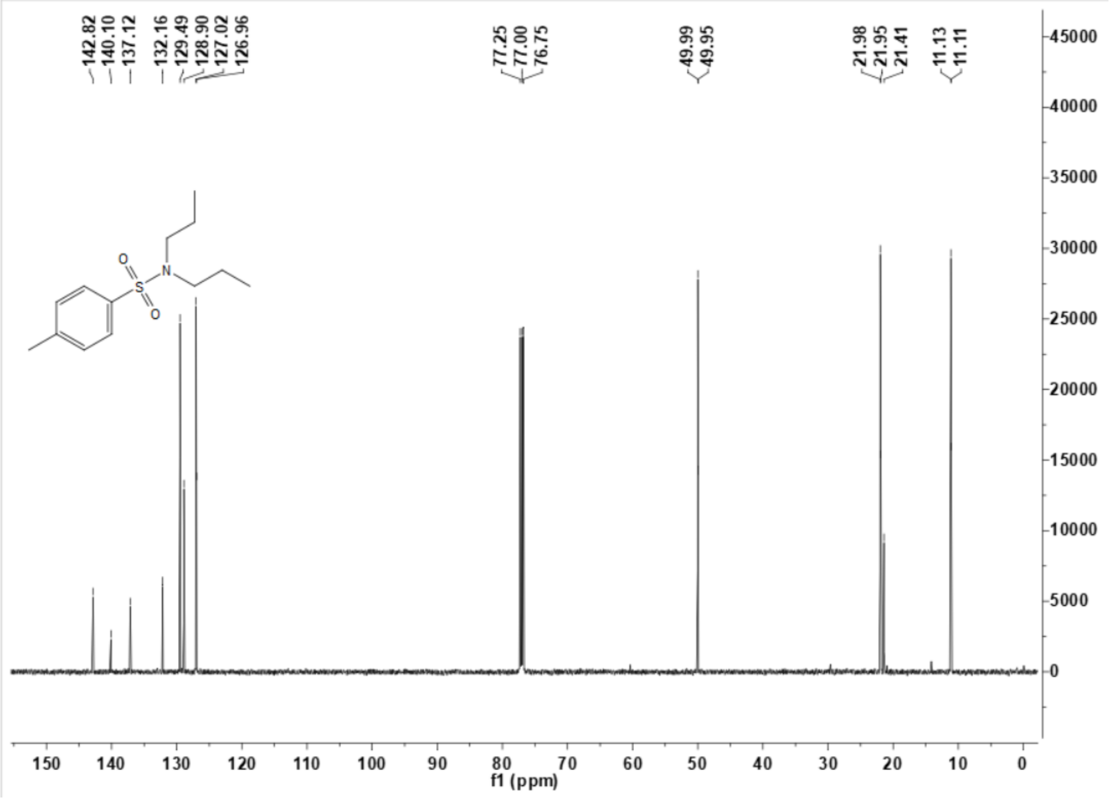

## 3,8-Dimethyl-2-phenyl-4*H*-chromen-4-one (6bb)

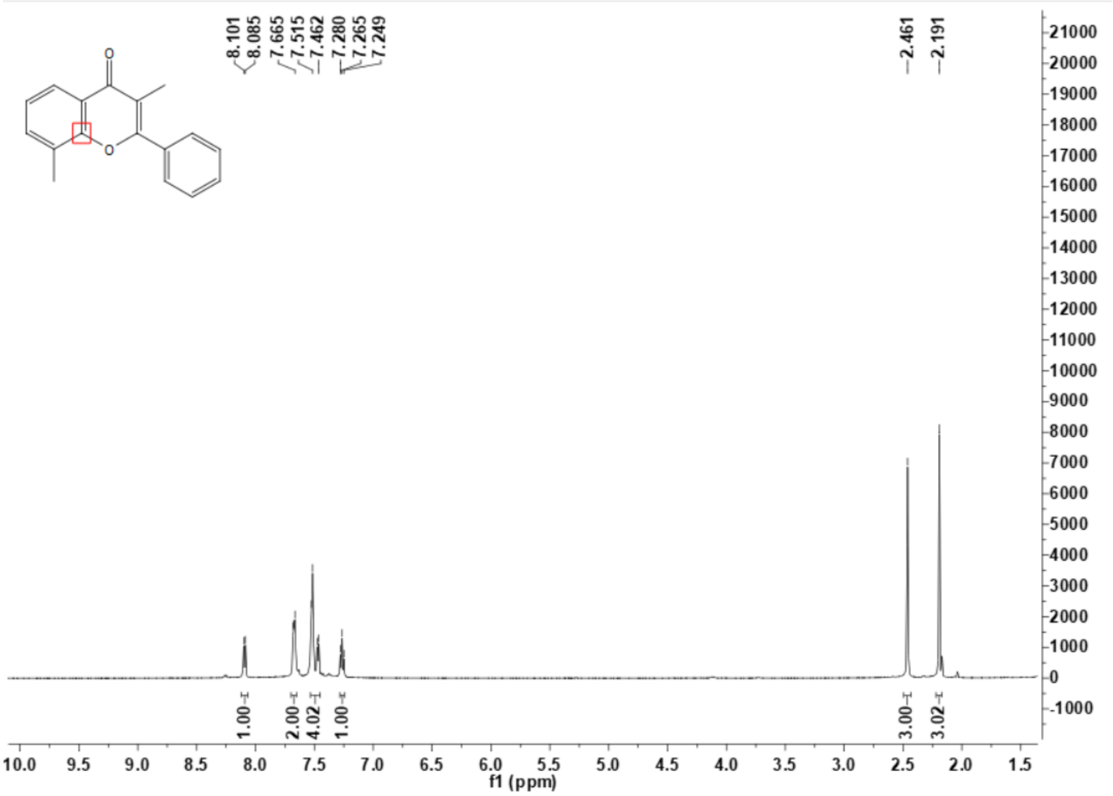


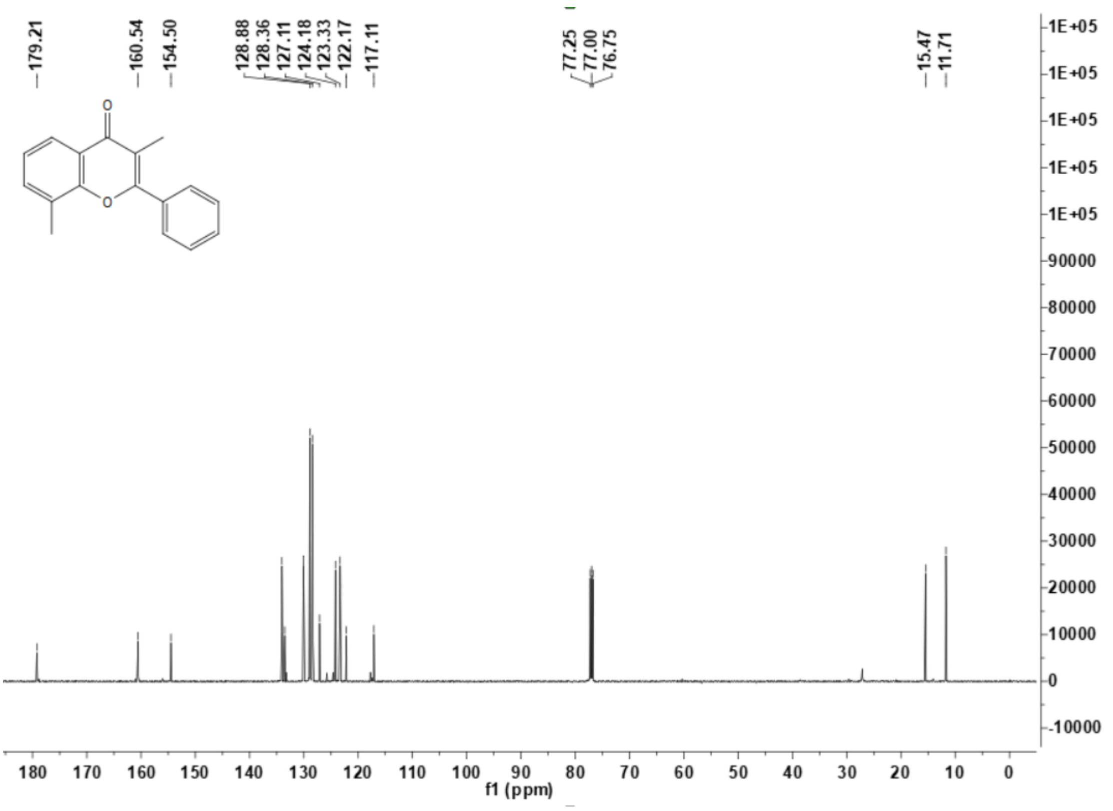

# 5-(4,5-dimethylthiazol-2-yl)-2-isobutoxybenzonitrile (6cb)

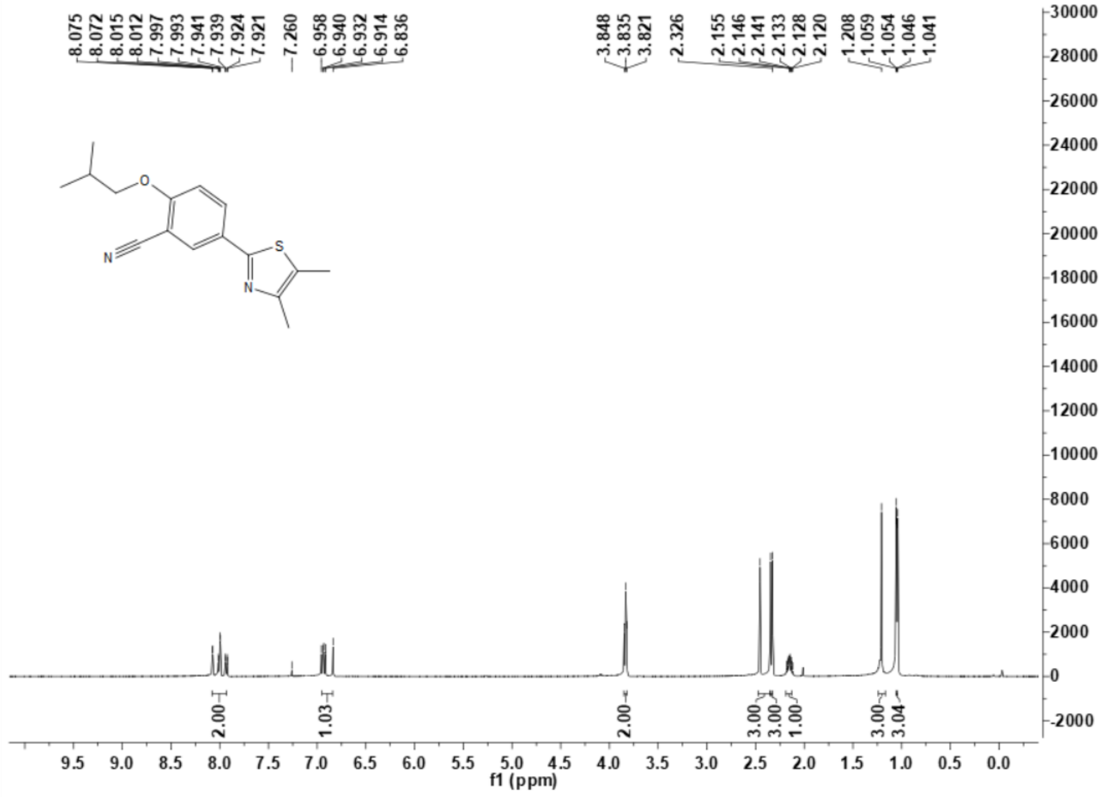


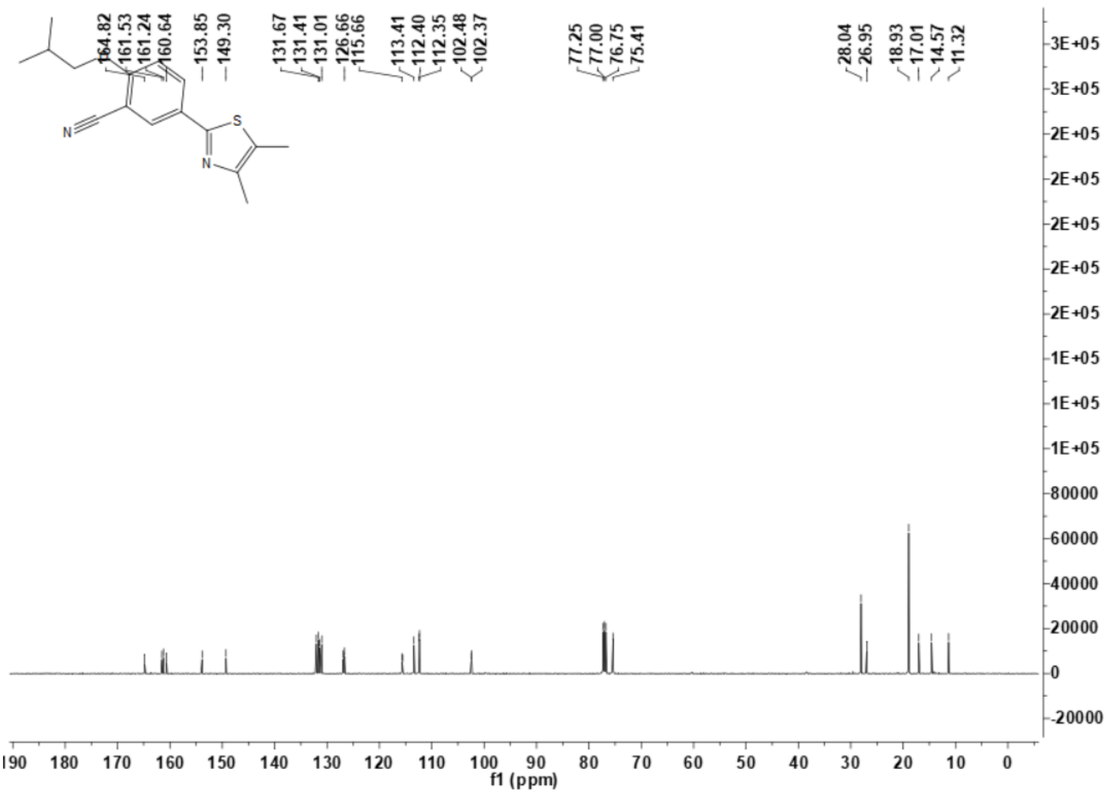

**1-(2-methoxy-5-(6-methylnaphthalen-2-yl)phenyl)adamantane (6db)**

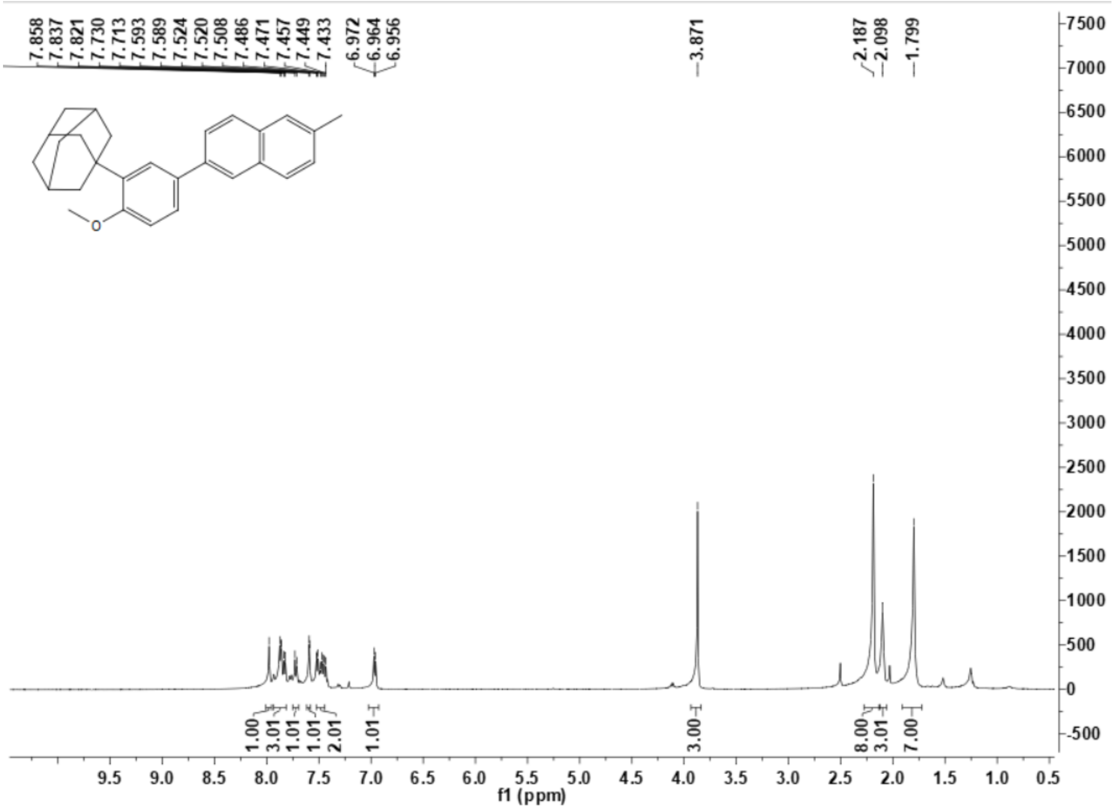


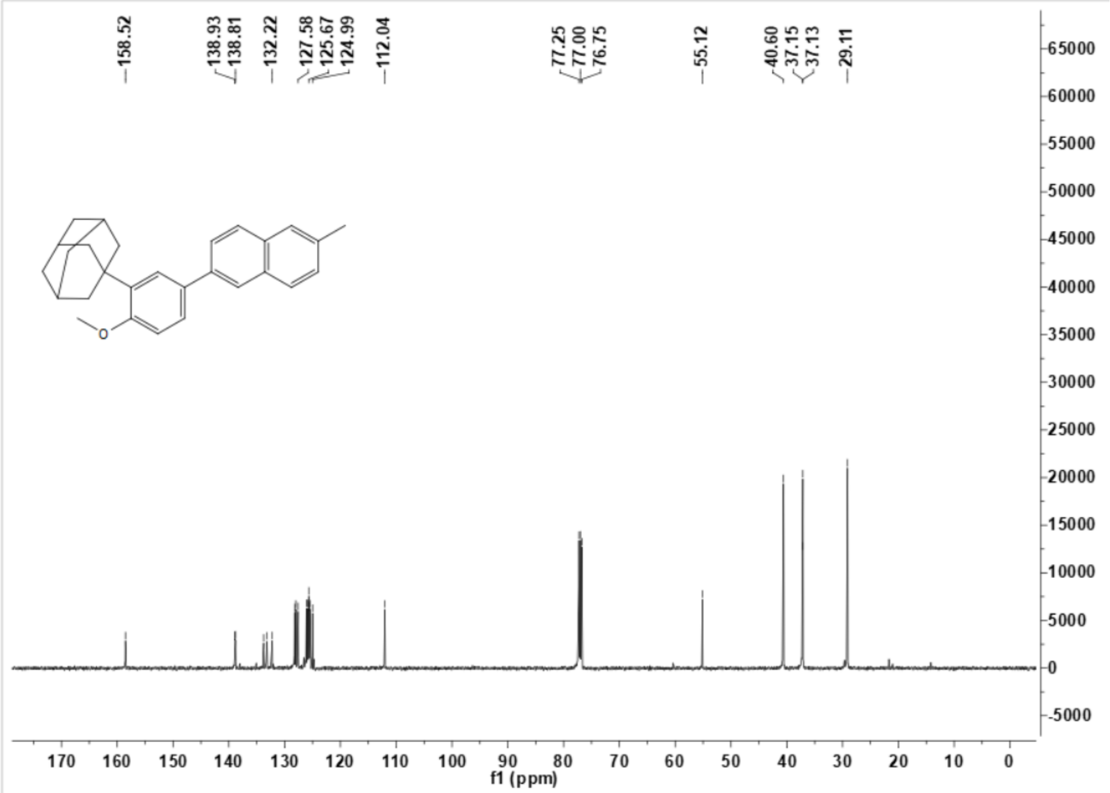

**2-(3-ethoxy-4-methylphenyl)-*N*-(3-methyl-1-(2-(piperidin-1-yl) phenyl) butyl) acetamide (6eb)**

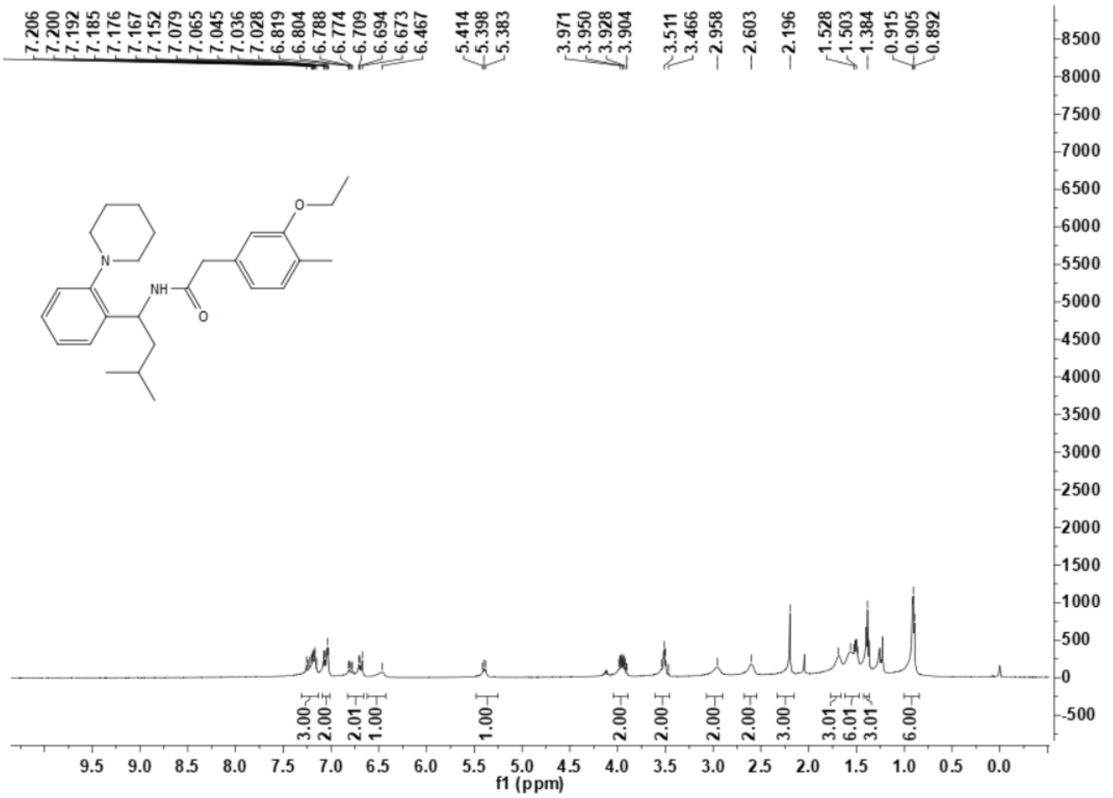

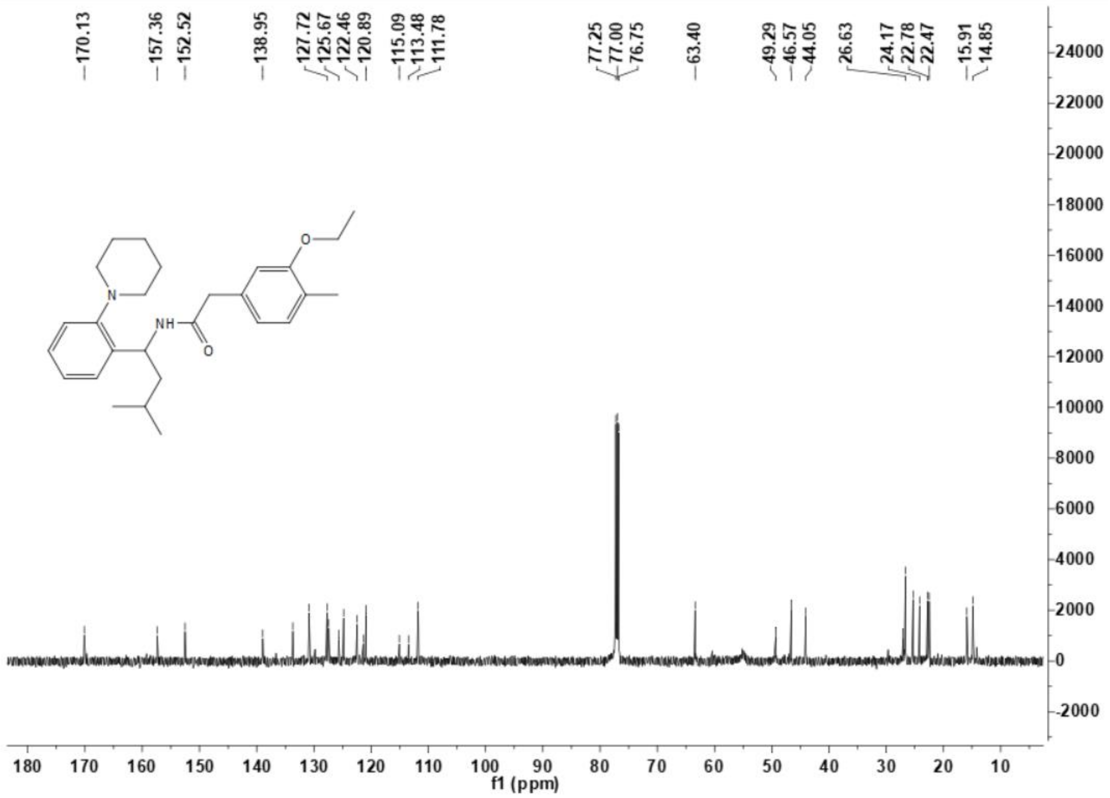

**1,1,4,4,5-pentamethyl-6-(1-(p-tolyl)vinyl)-1,2,3,4-tetrahydronaphthalene (6fb)**

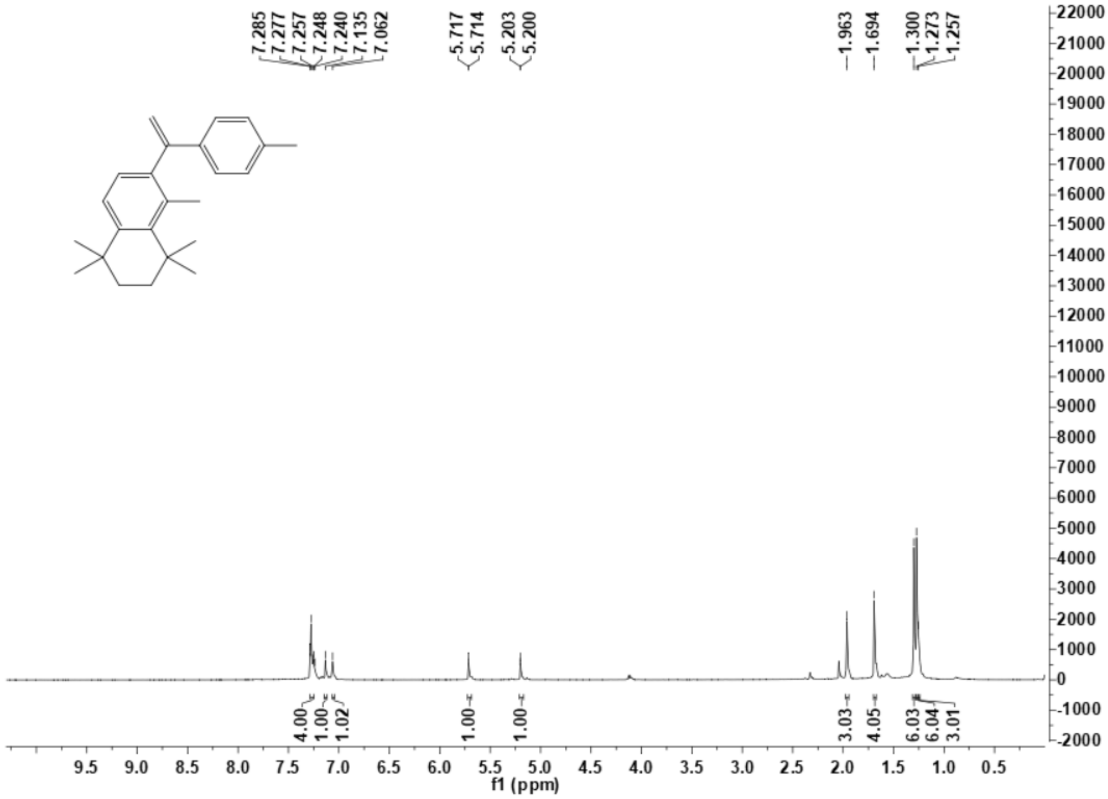


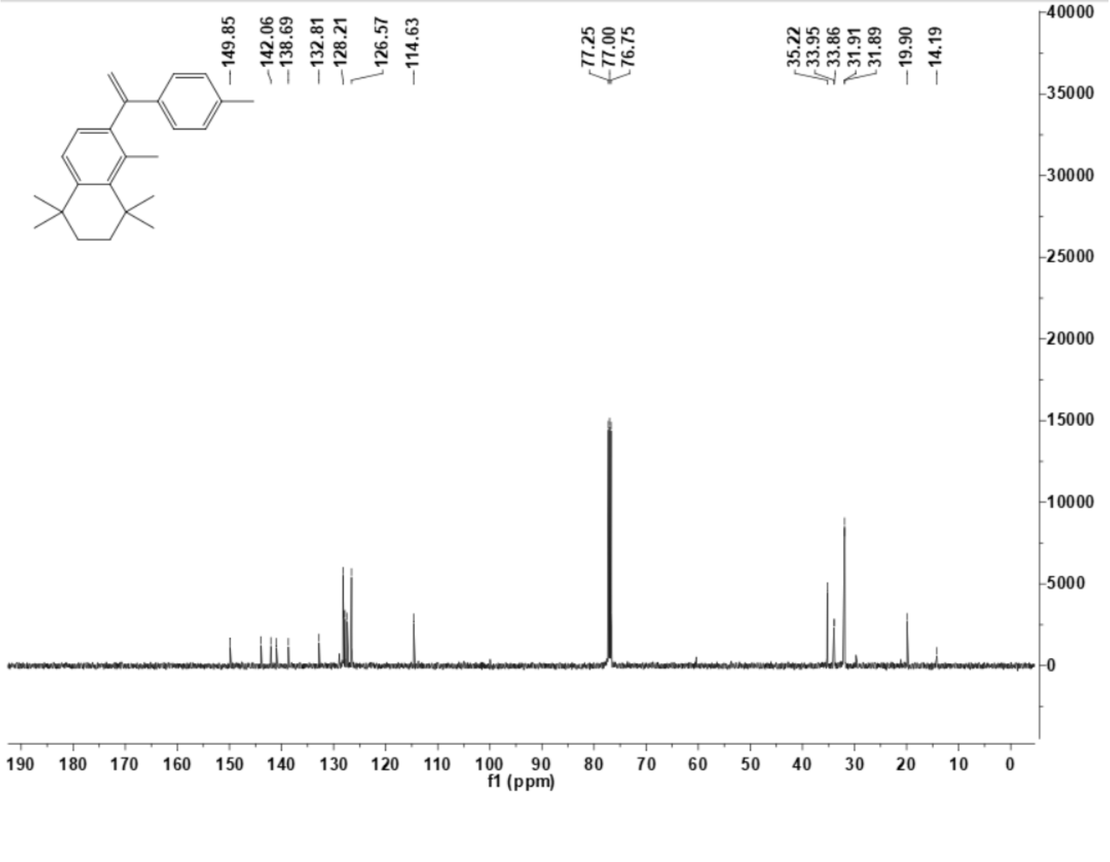

**1,4'-dimethyl-1'-((2'-methyl-[1,1'-biphenyl]-4-yl)methyl)-2'-propyl-1*H*,1'*H*-2,5'-bibenzo[*d*]imidazole (6gb)**

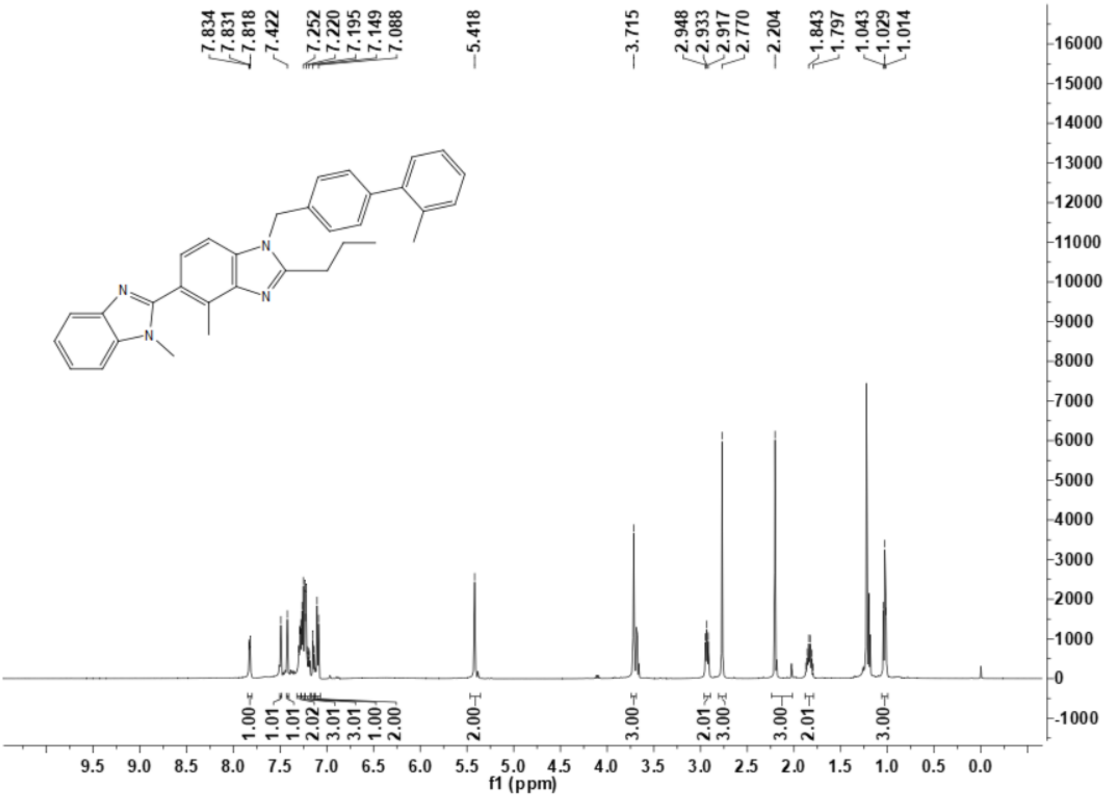

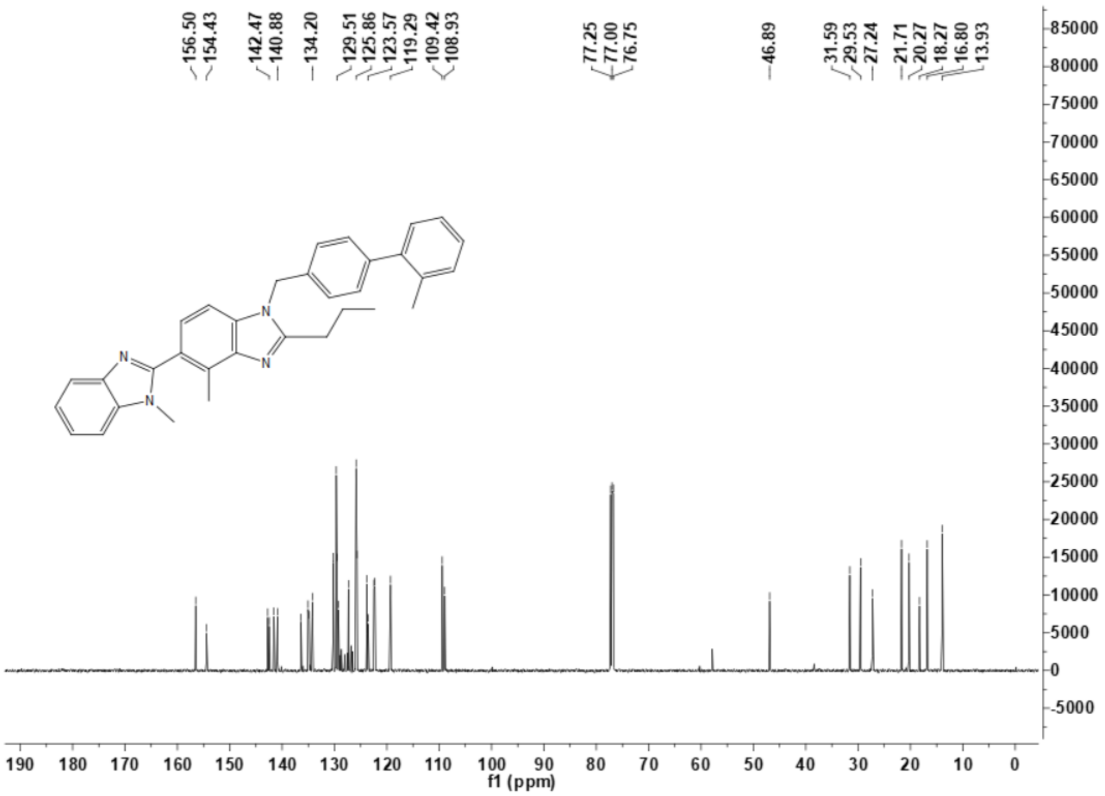

**(8*R*,9*S*,13*S*,14*S*)-13-methyl-17-oxo-7,8,9,11,12,13,14,15,16,17-decahydro-6*H*-cyclopenta[*a*]phenanthren-3-yl 4-methylbenzoate (6hb)**.

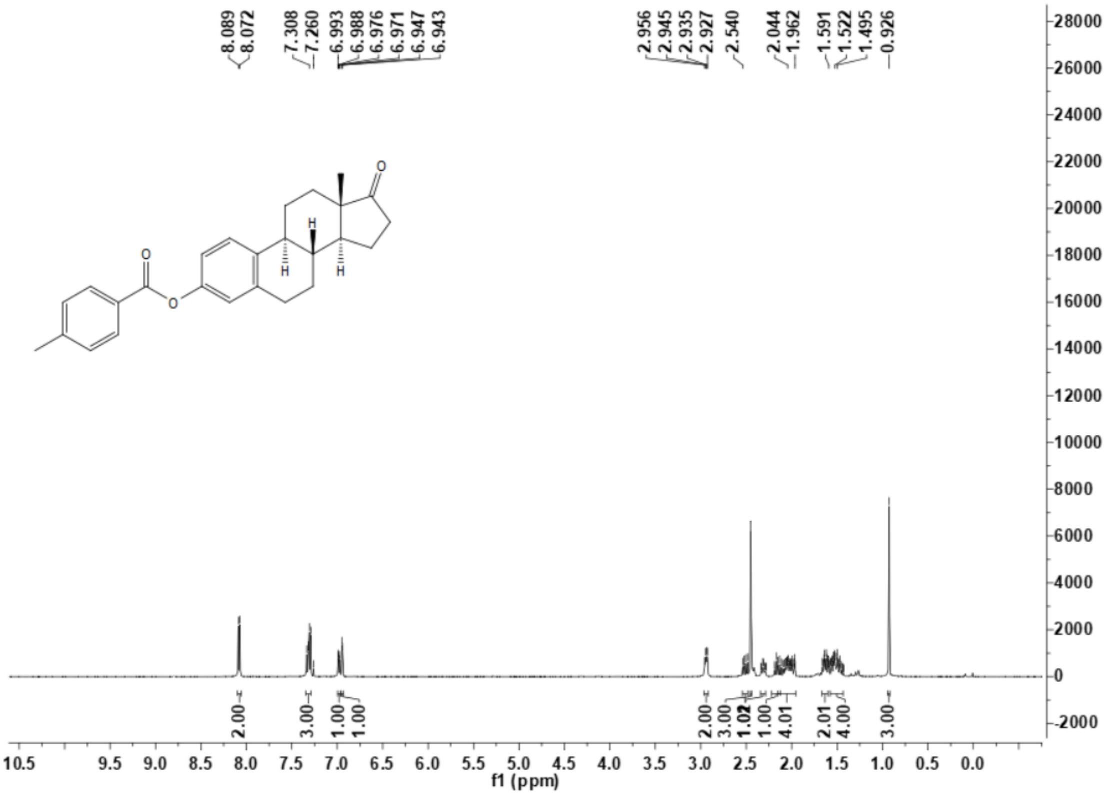

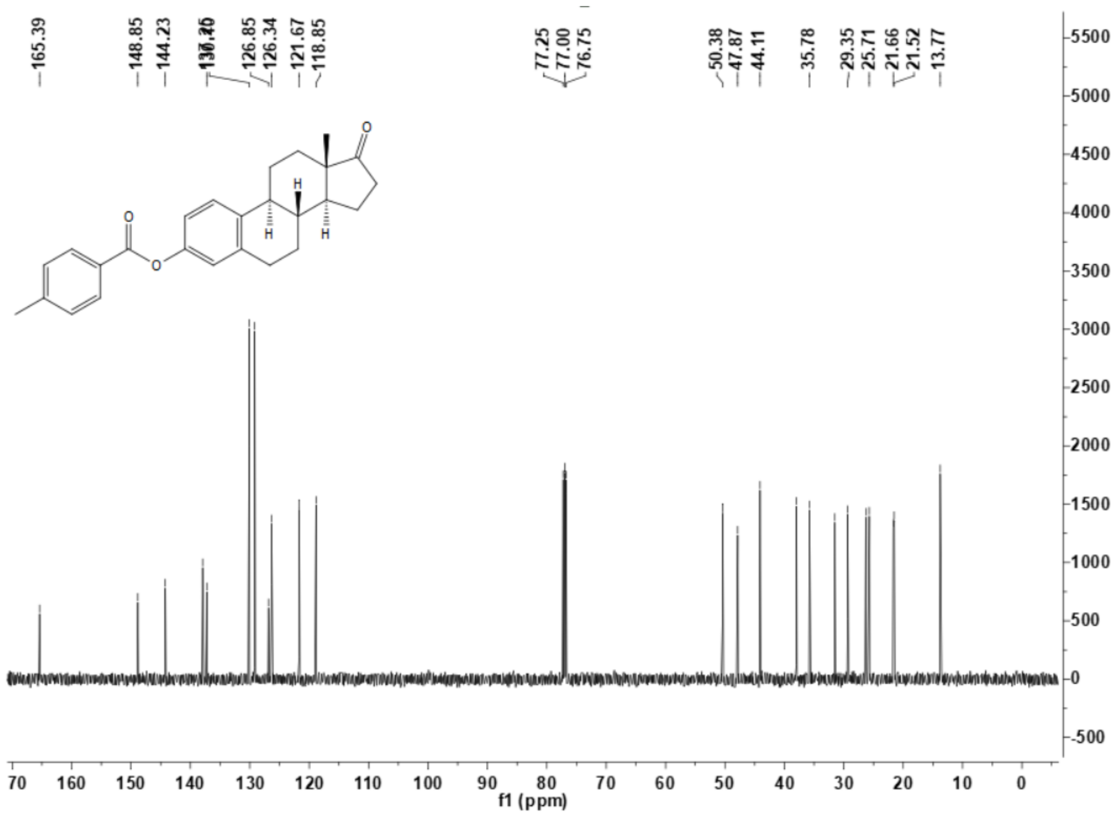

**(*R*)-2,5,7,8-tetramethyl-2-((4R,8R)-4,8,12-trimethyltridecyl) chroman-6-yl 4-methylbenzoate (6ib)**.

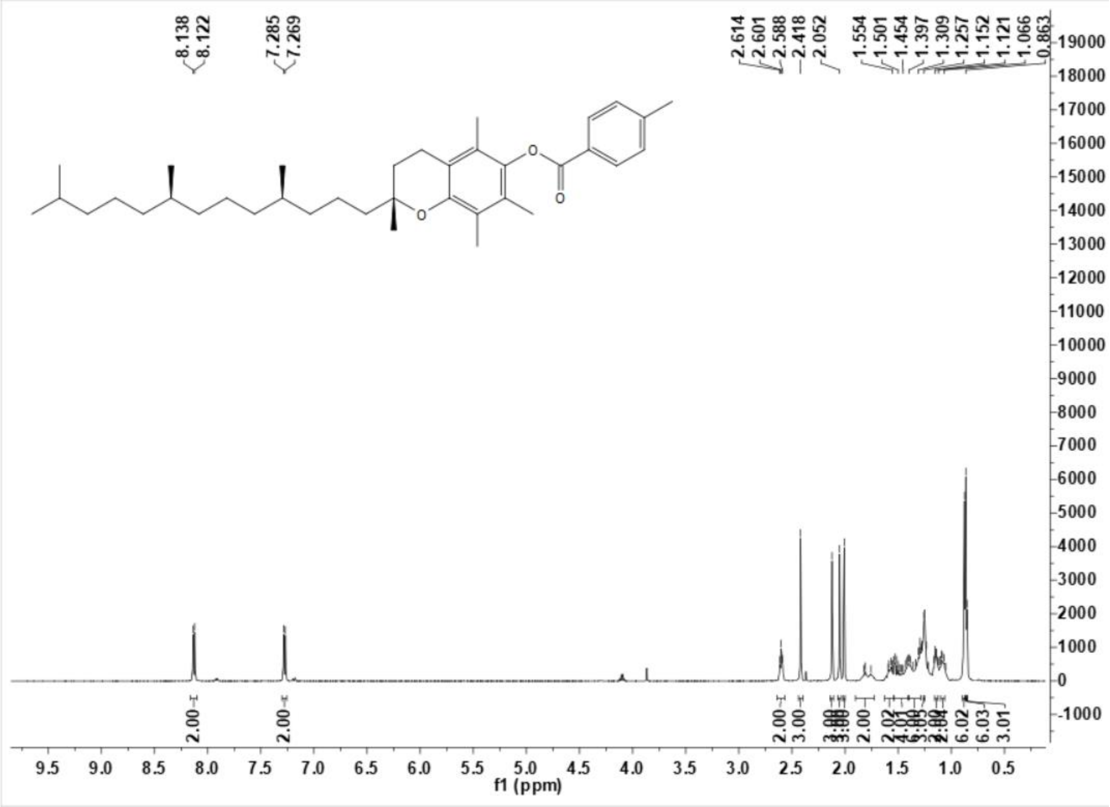

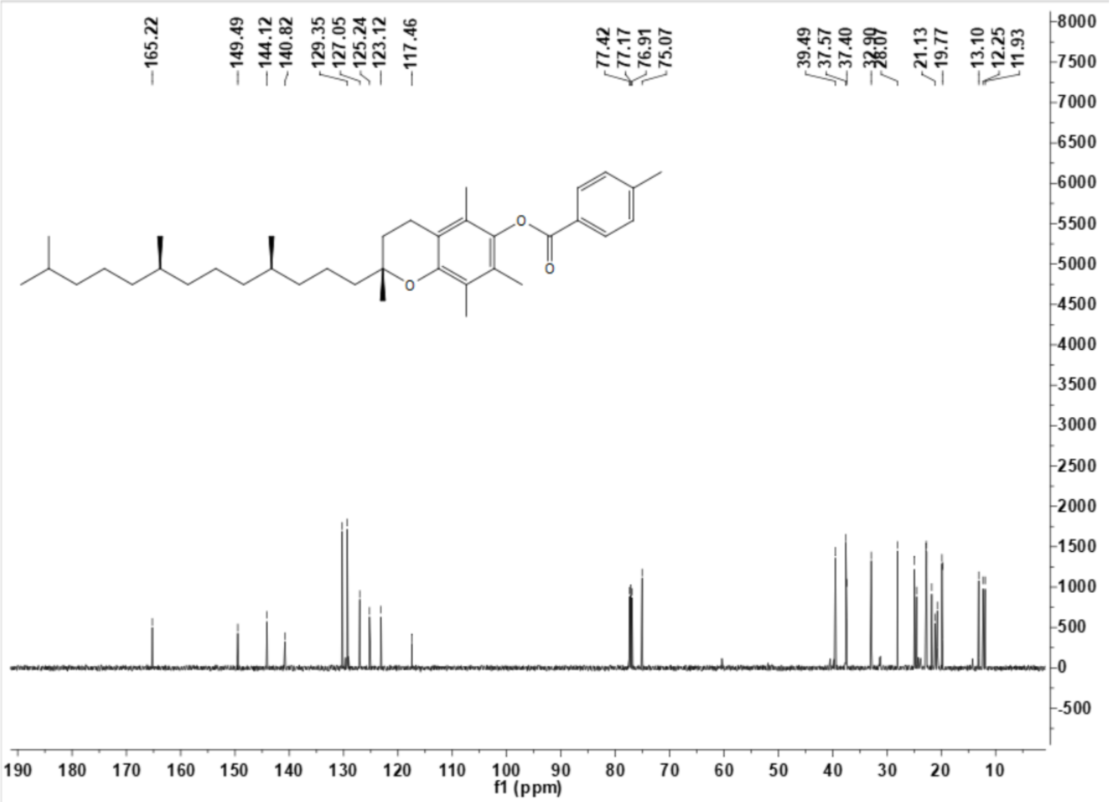

**4-Methyl-2-*oxo*-2*H*-chromen-7-yl 4-methylbenzoate (6jb)**.

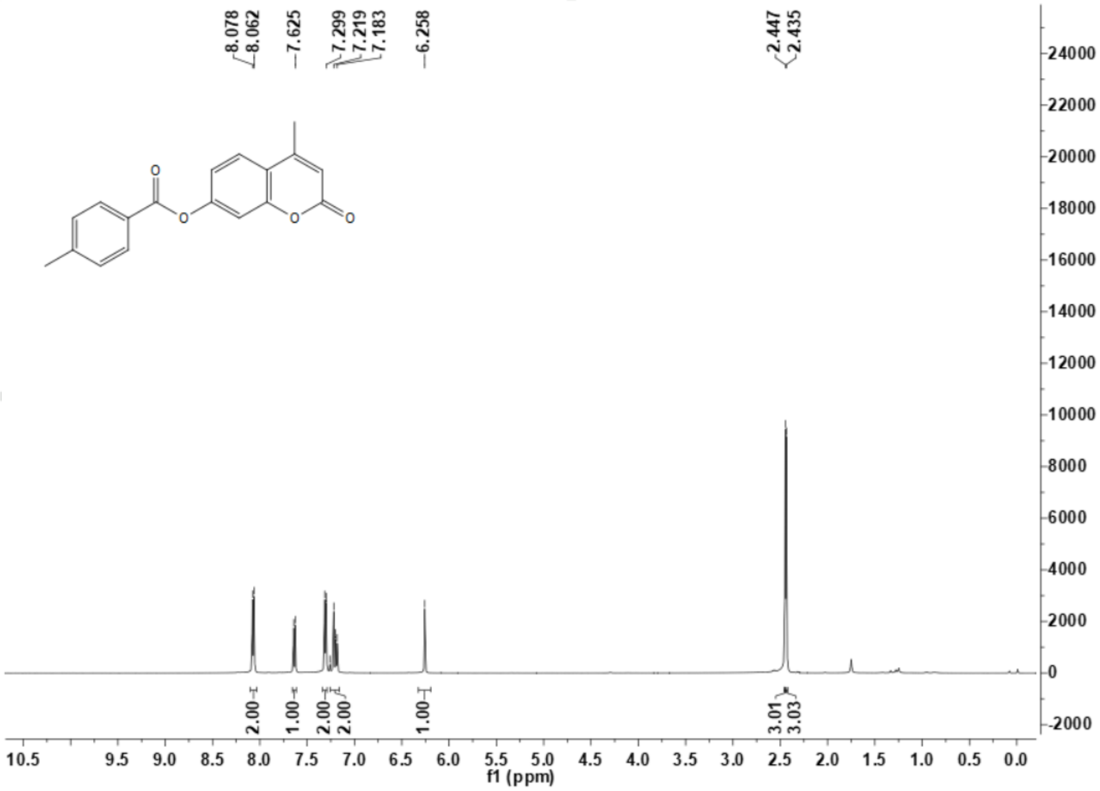


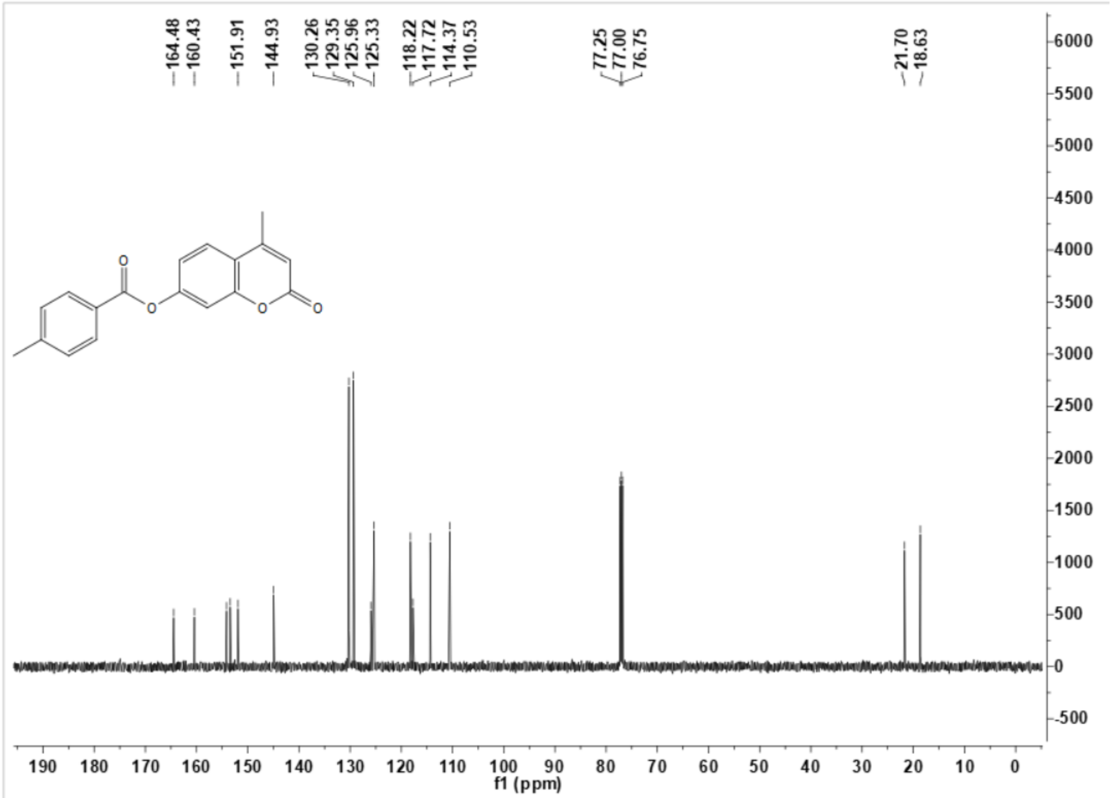

**(7*R*,8*R*,9*S*,13*S*,14*S*,17*S*)-7-(9-bromononyl)-17-hydroxy-13-methyl-7,8,9,11,12,13,14,15,16,17-decahydro-6*H*-cyclopenta[a]phenanthren-3-yl 4-methylbenzoate (6kb)**.

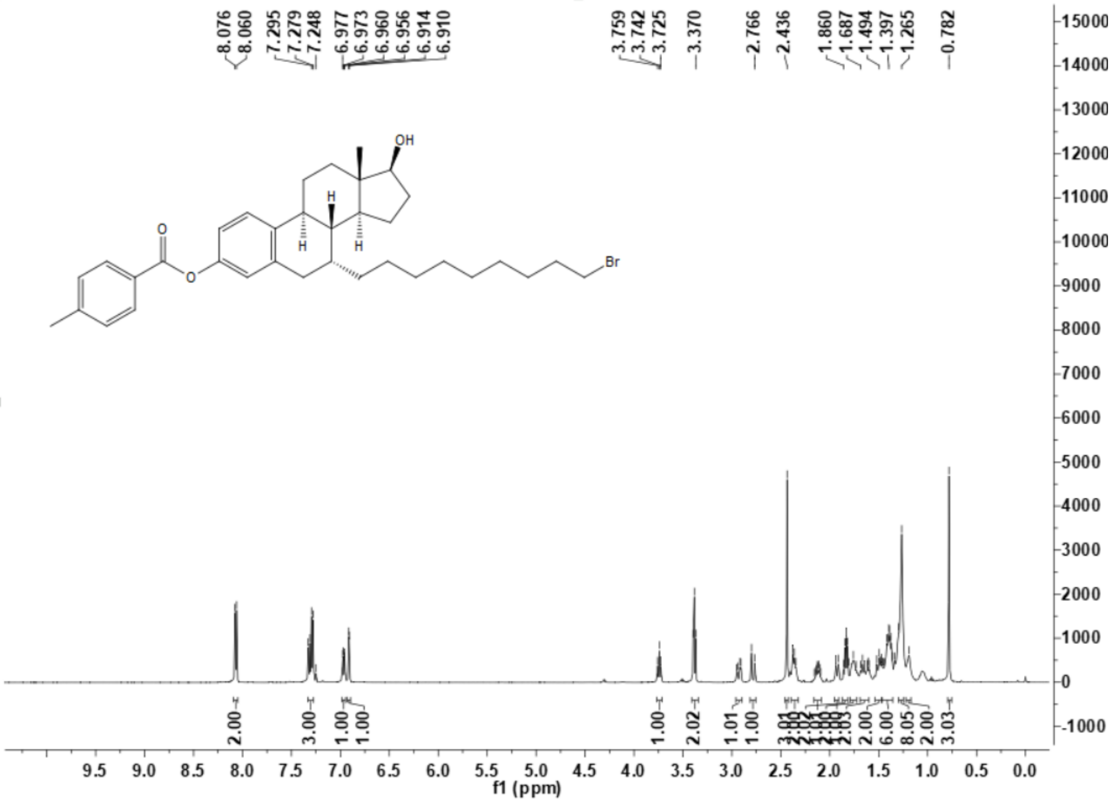


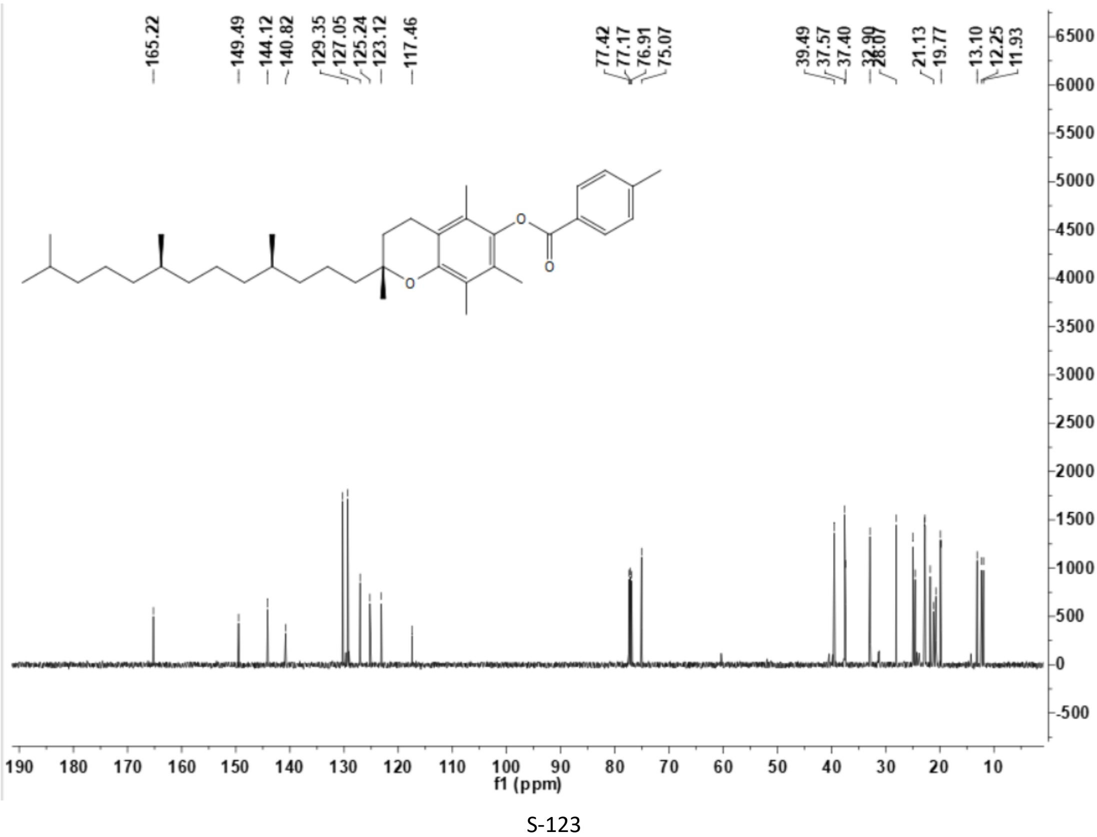

**4-(2-((((9*H*-fluoren-9-yl)methoxy)carbonyl)amino)-1-methoxy-1-oxohex-5-yn-3-yl)phenyl 4-methylbenzoate (6lb)**.

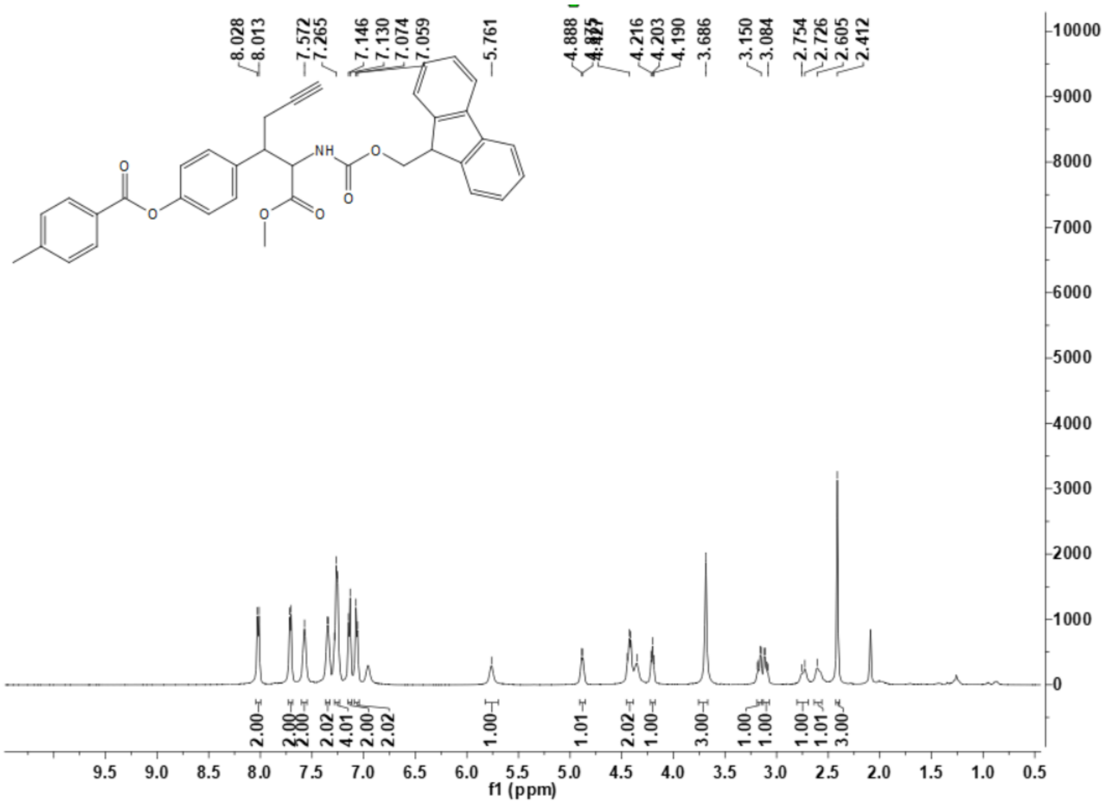

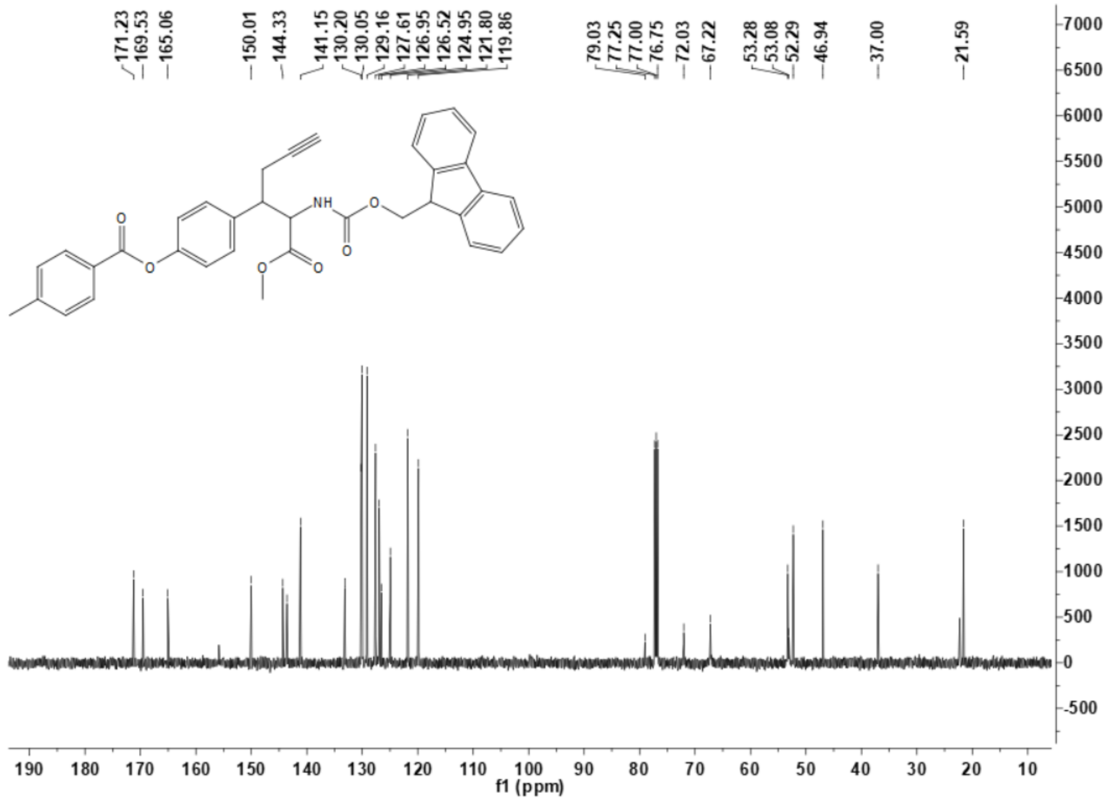

**4-(2-((((9*H*-fluoren-9-yl)methoxy)carbonyl)amino)-3-methoxy-3-oxopropyl)phenyl 4-methylbenzoate (6mb)**.

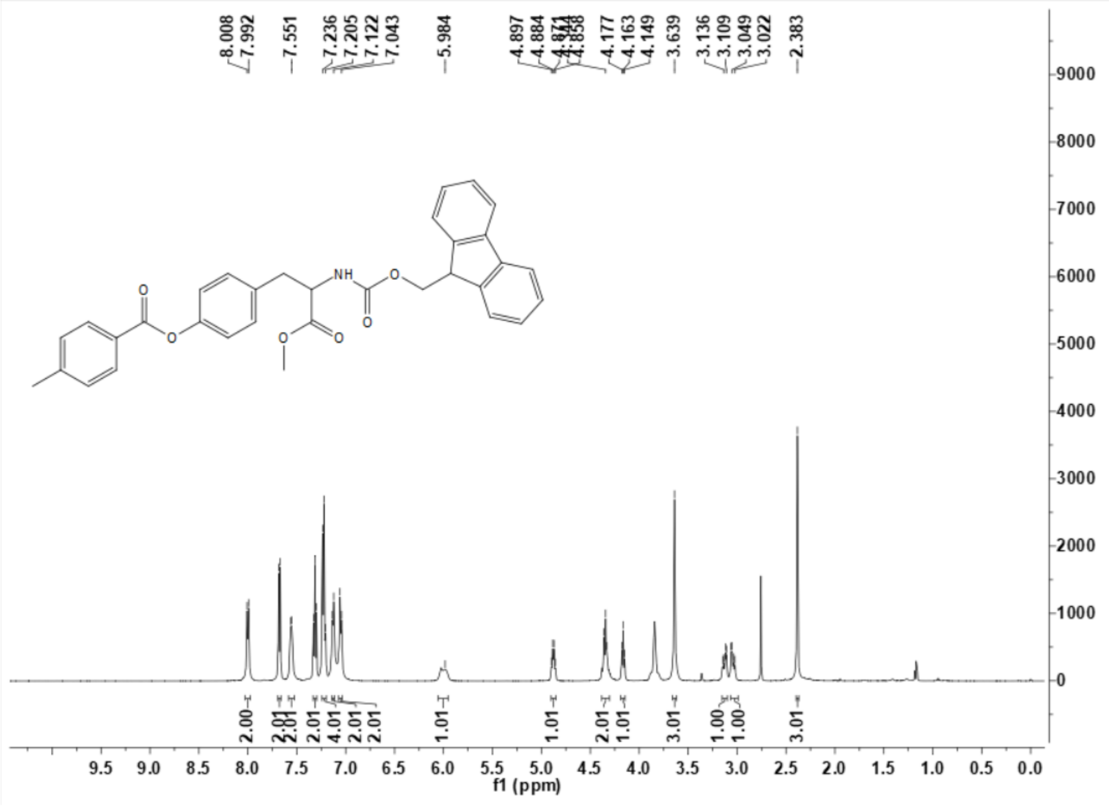

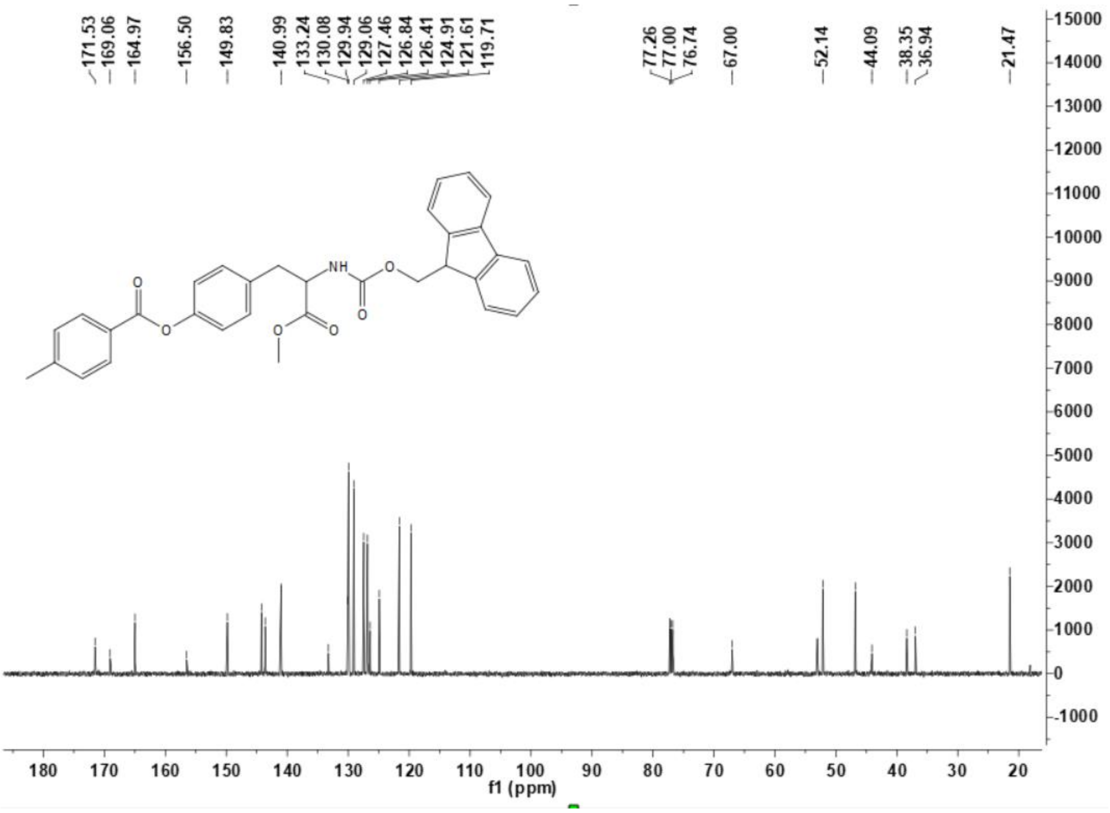

**4-(2-((*tert*-butoxycarbonyl) amino)-3-methoxy-3-oxopropyl) phenyl 4-methylbenzoate (6nb)**.

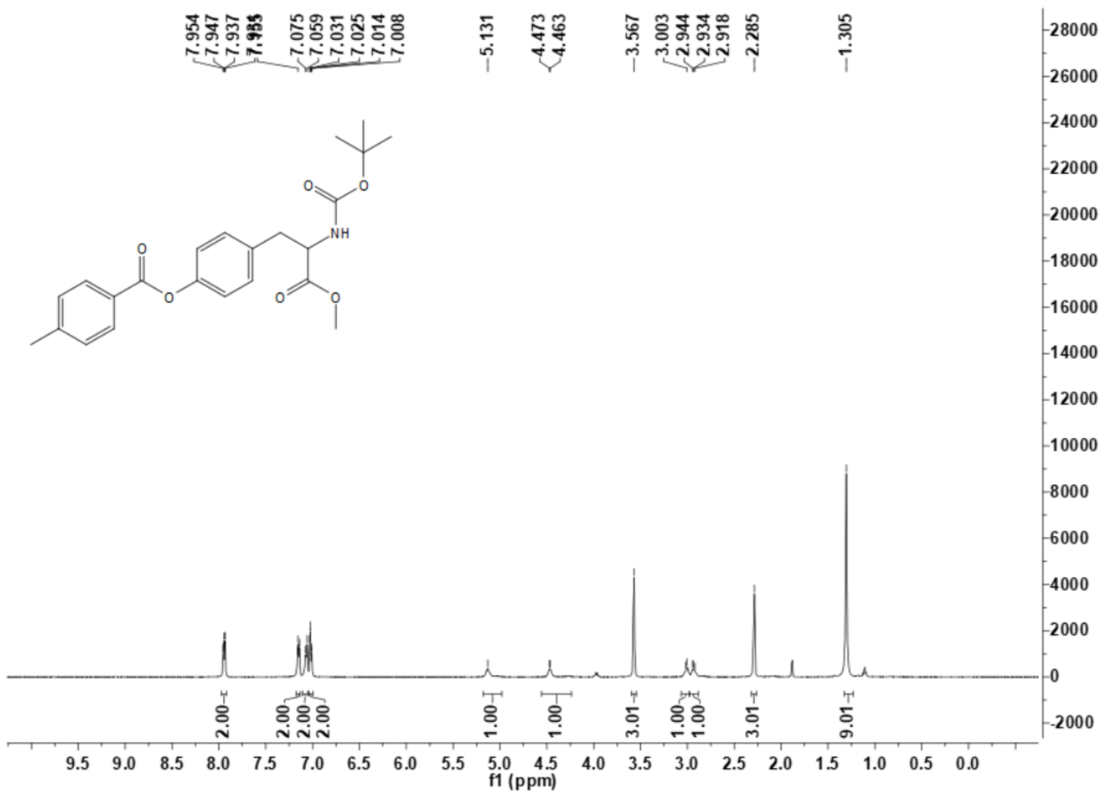


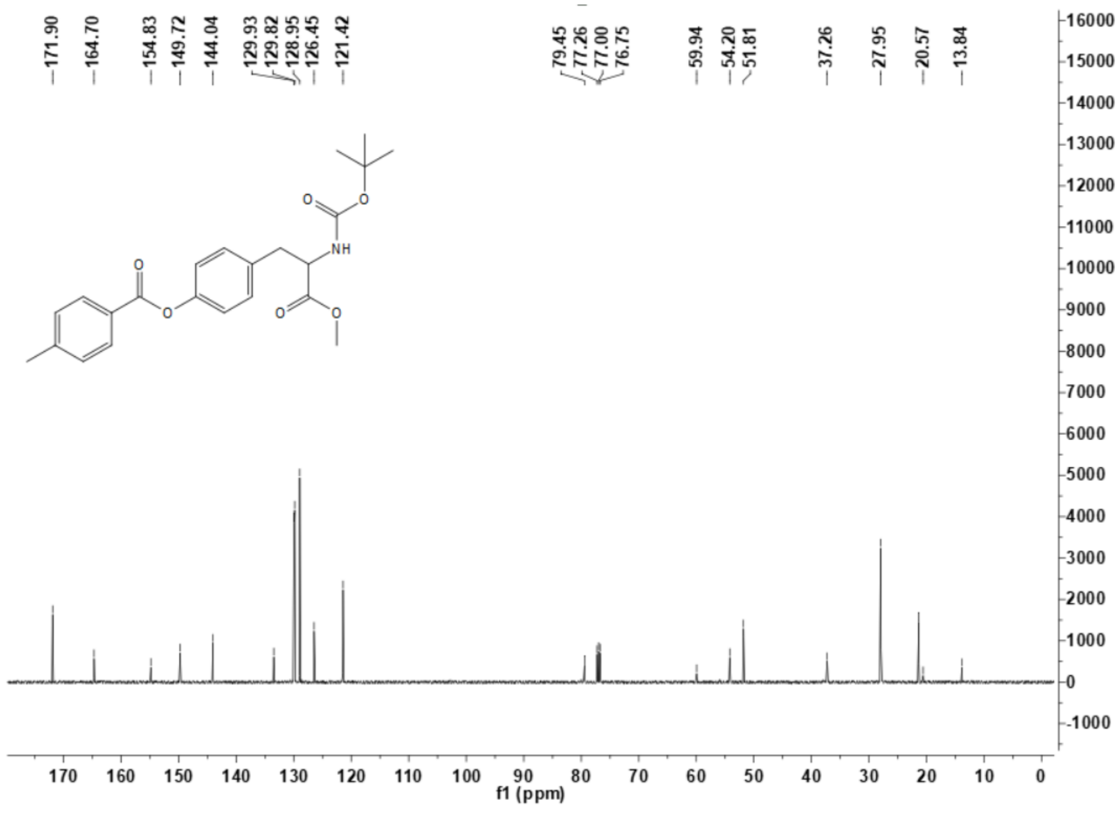

**Ni(dpph) (*o*-tolyl)Cl (9)**

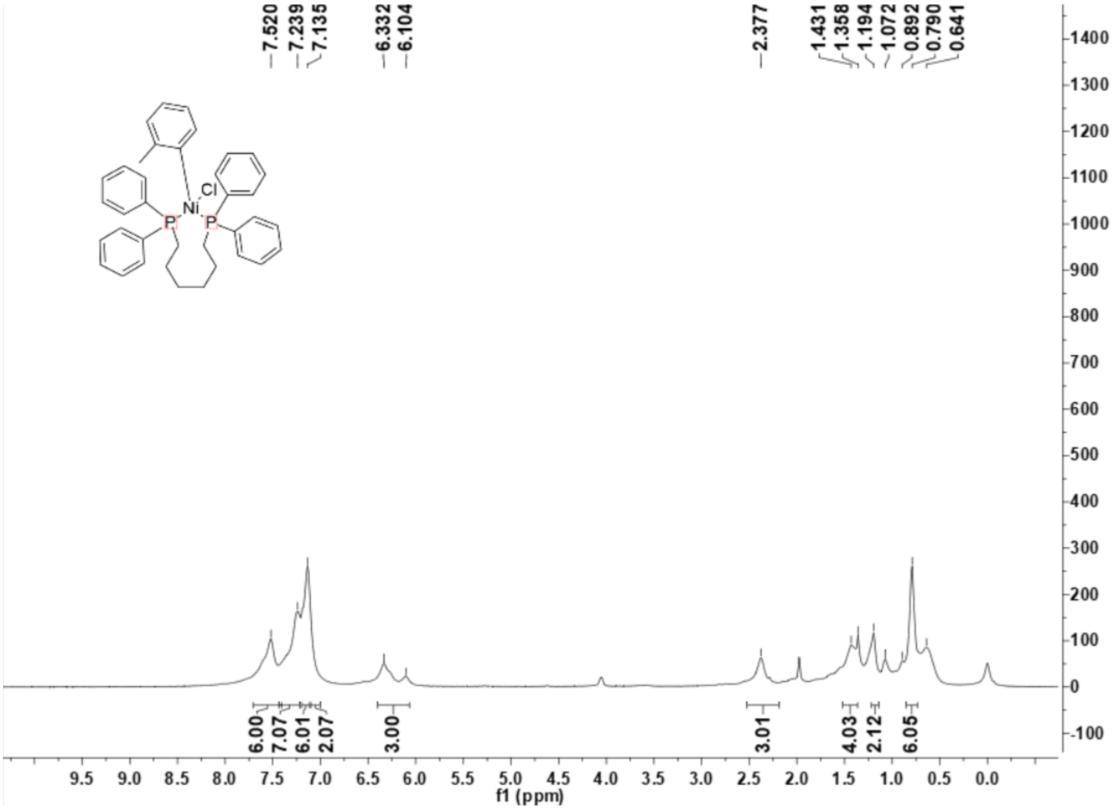

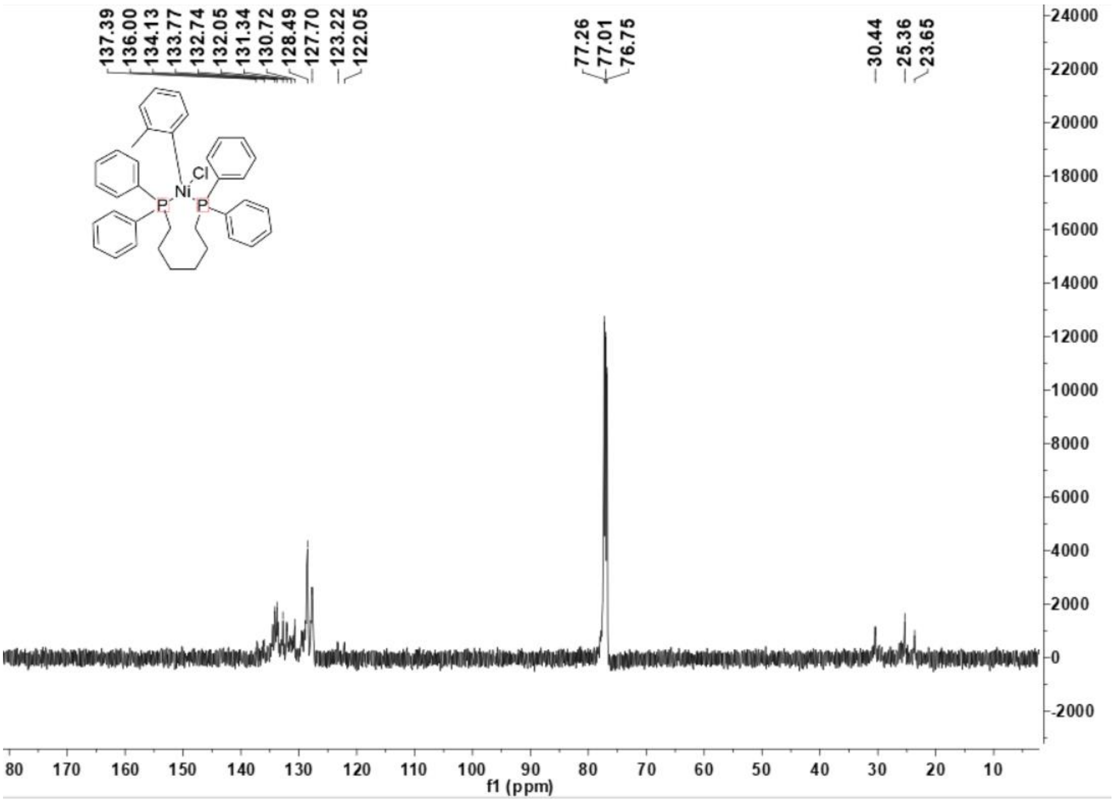

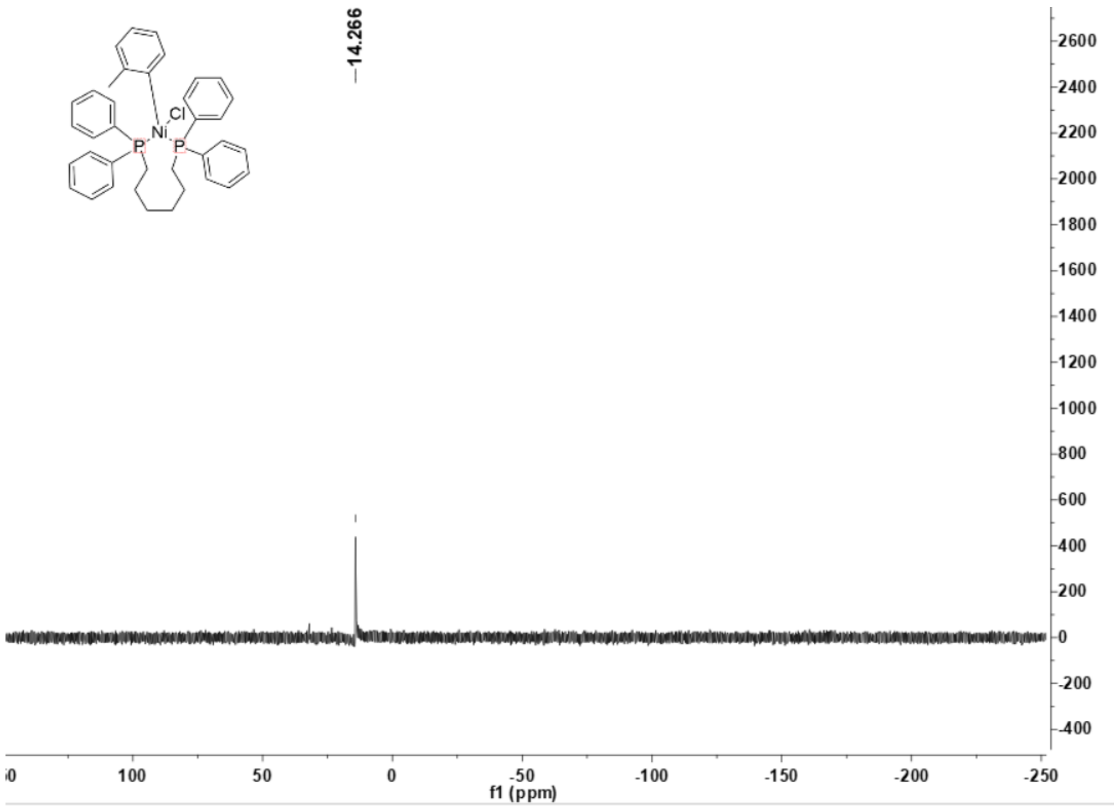


## Ni(dtbbpy) (*o*-formal) Cl (10)

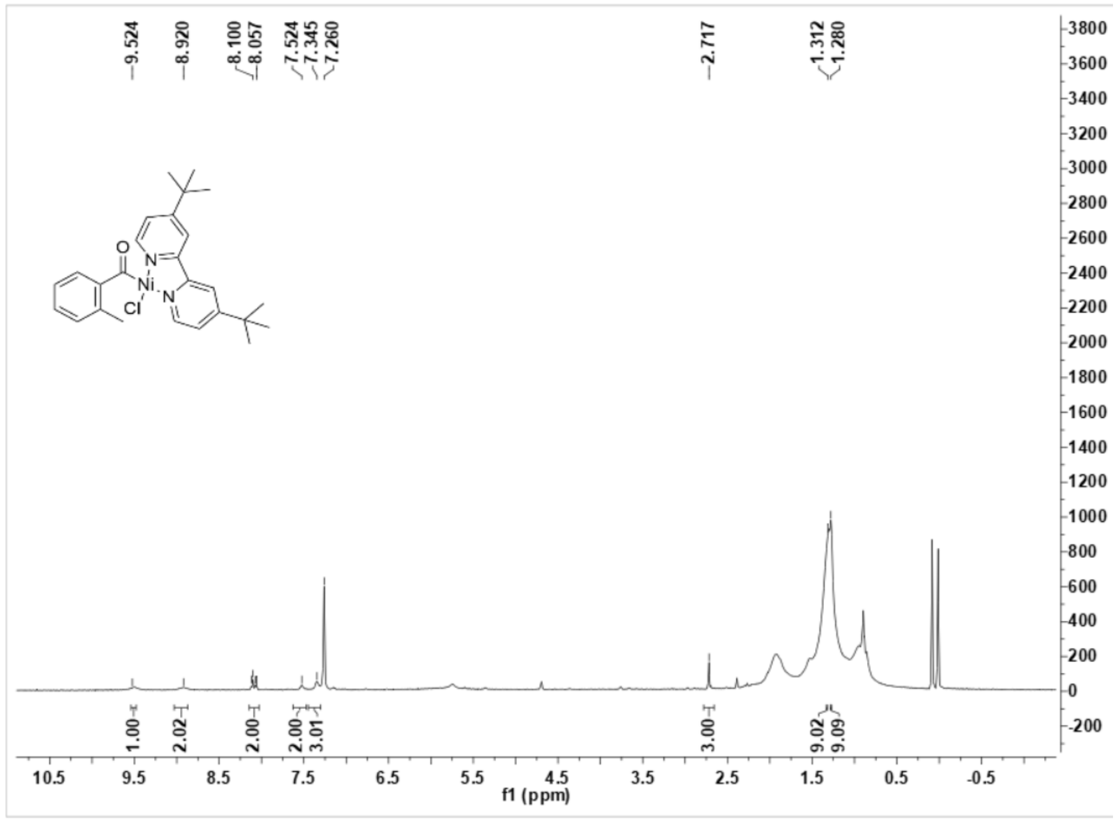

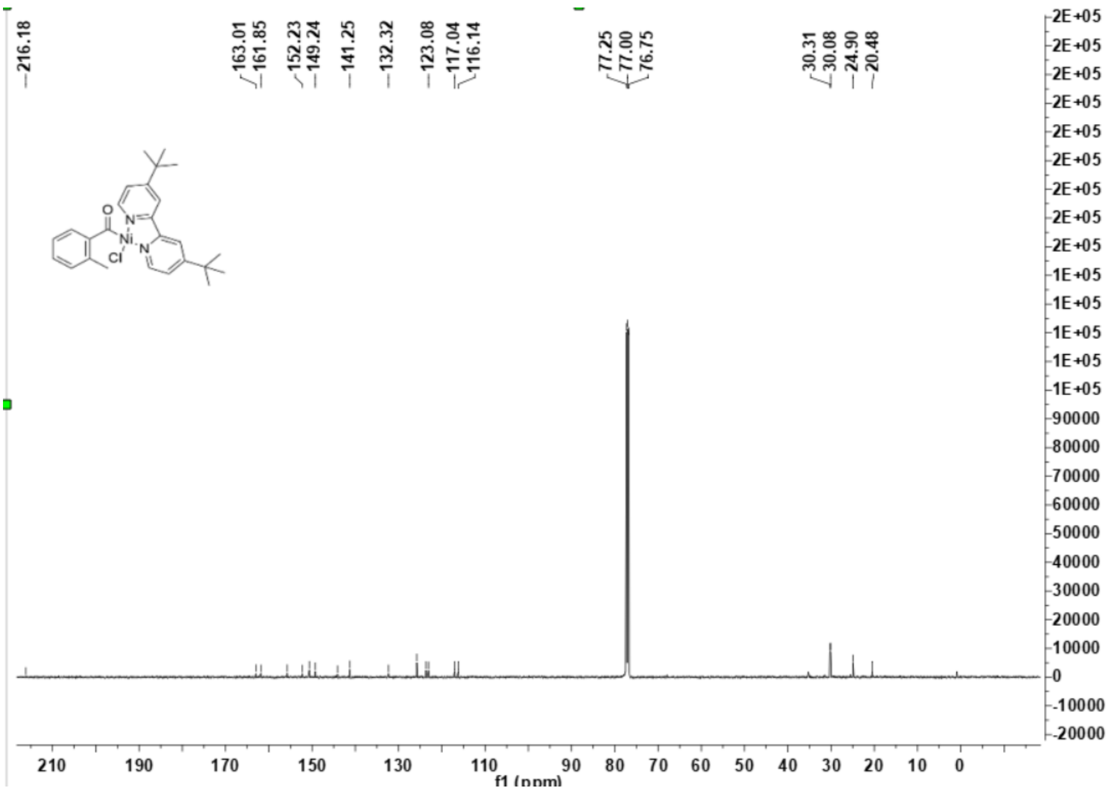


**Ni(dtbbpy)(*o*-tolyl)Cl (11)**

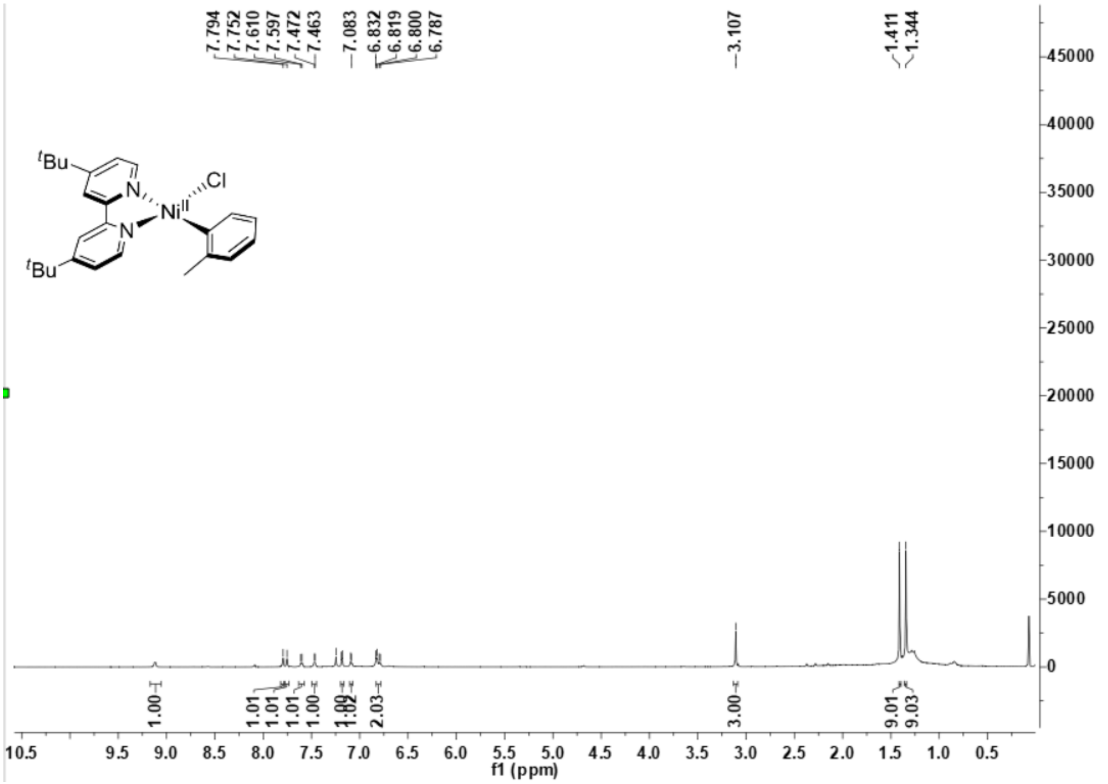

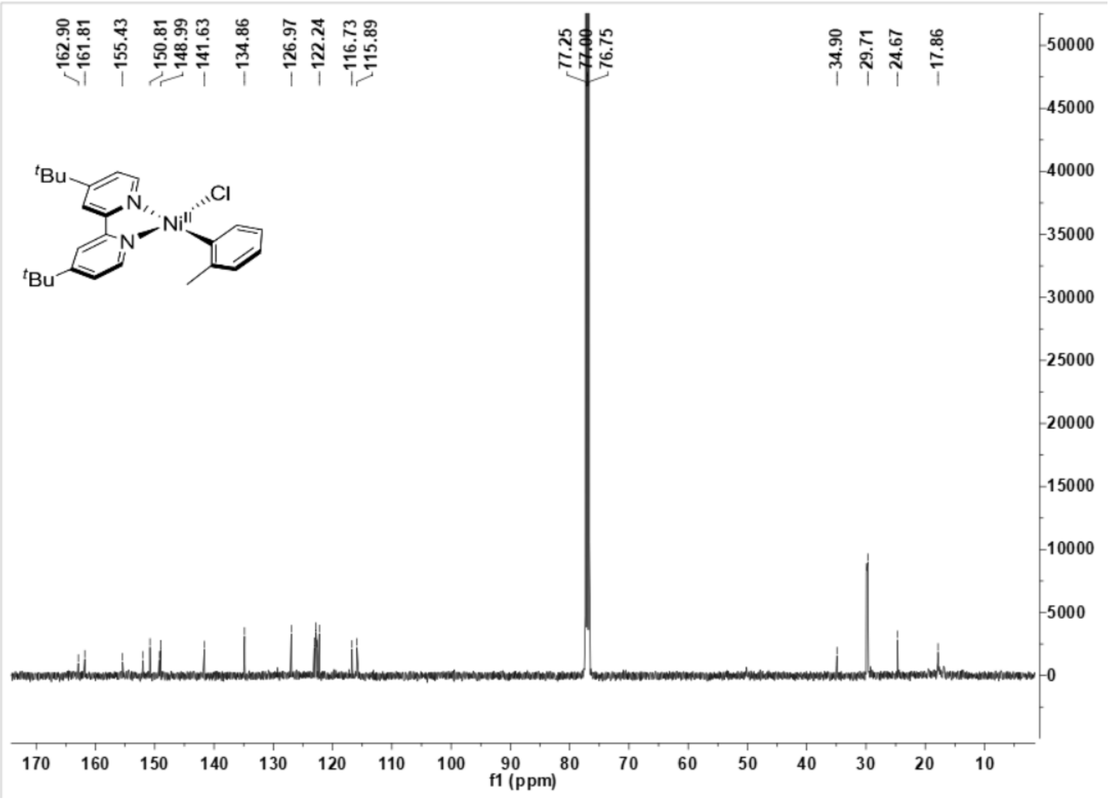


**Ni($PEt_3$)$_2$ (*o*-formal) Cl (21).**

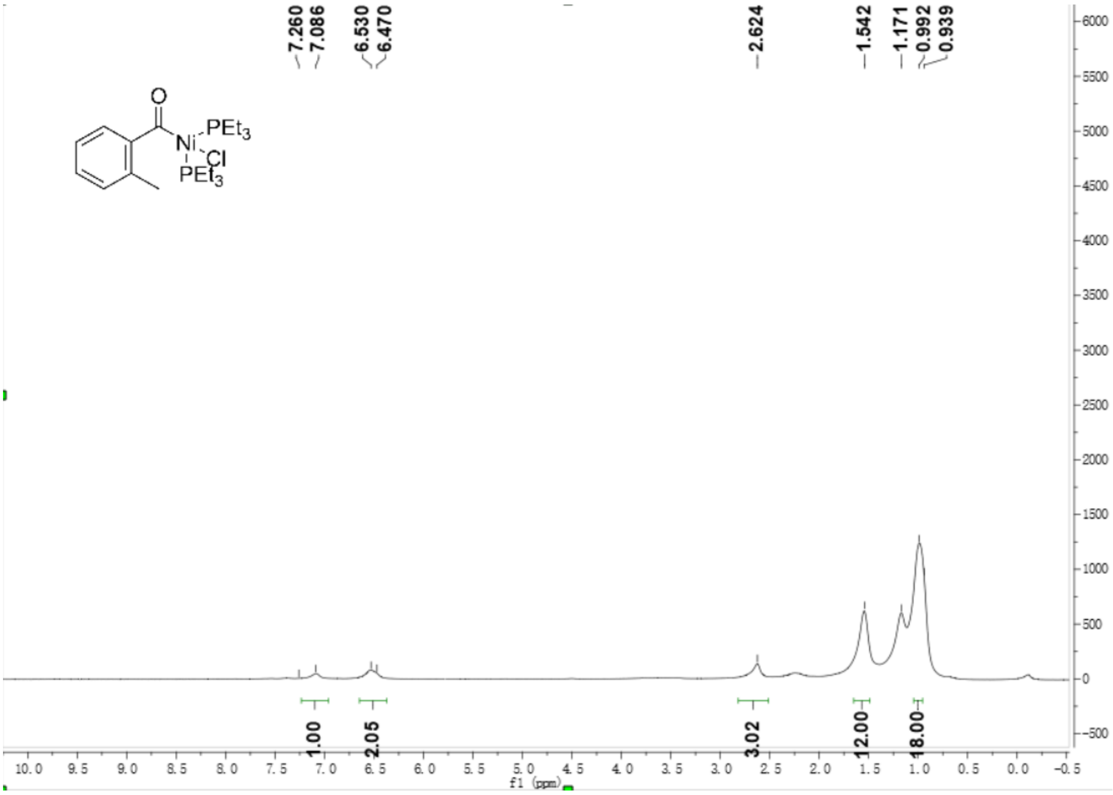

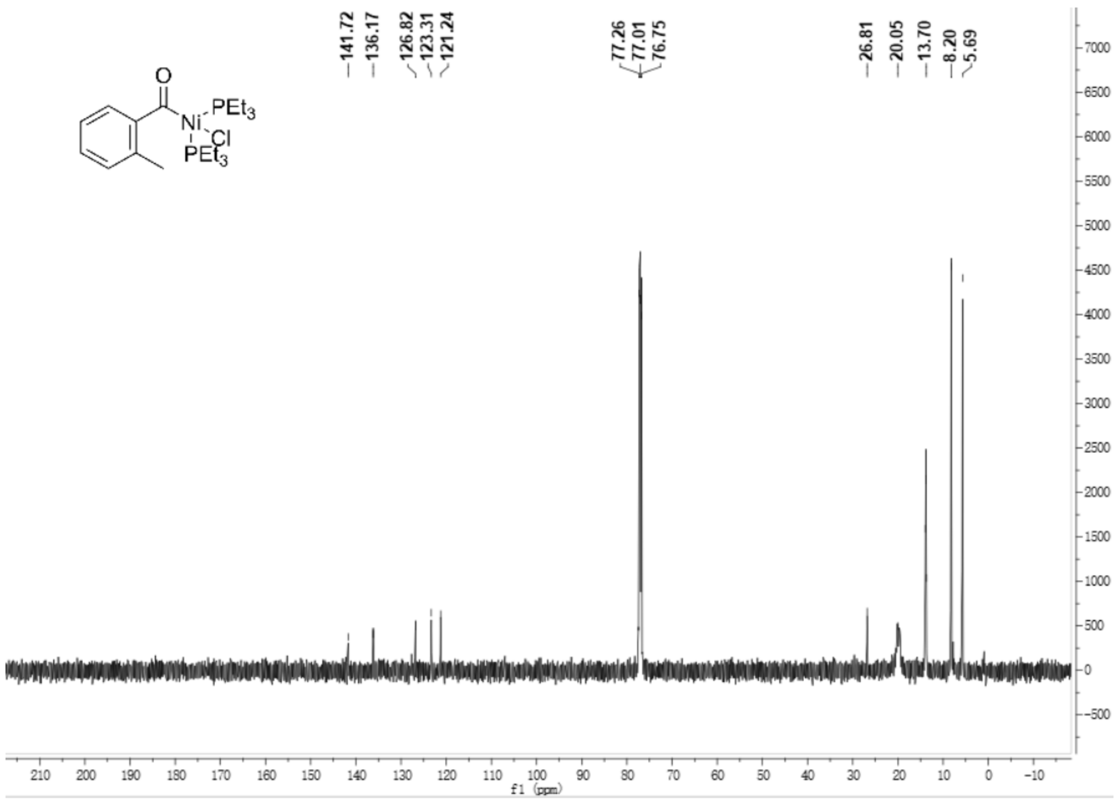
141.72
136.17
126.82
123.31
121.24
77.26
77.01
76.75
26.81
20.05
13.70
8.20
5.69
PEt3
Ni
Cl
PEt3
f1 (ppm)

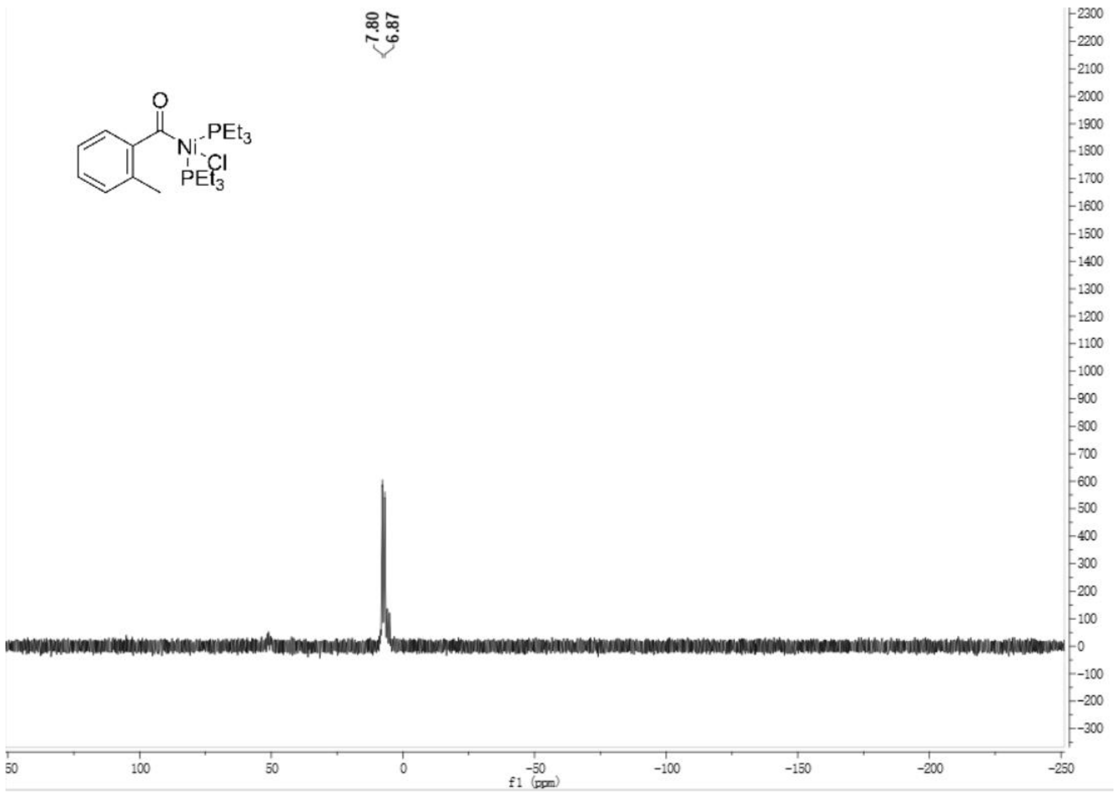
7.80
6.87
PEt3
Ni
Cl
PEt3
f1 (ppm)

## Ni(PEt$_3$)$_2$ (*o*-toluoyl) Cl (22)

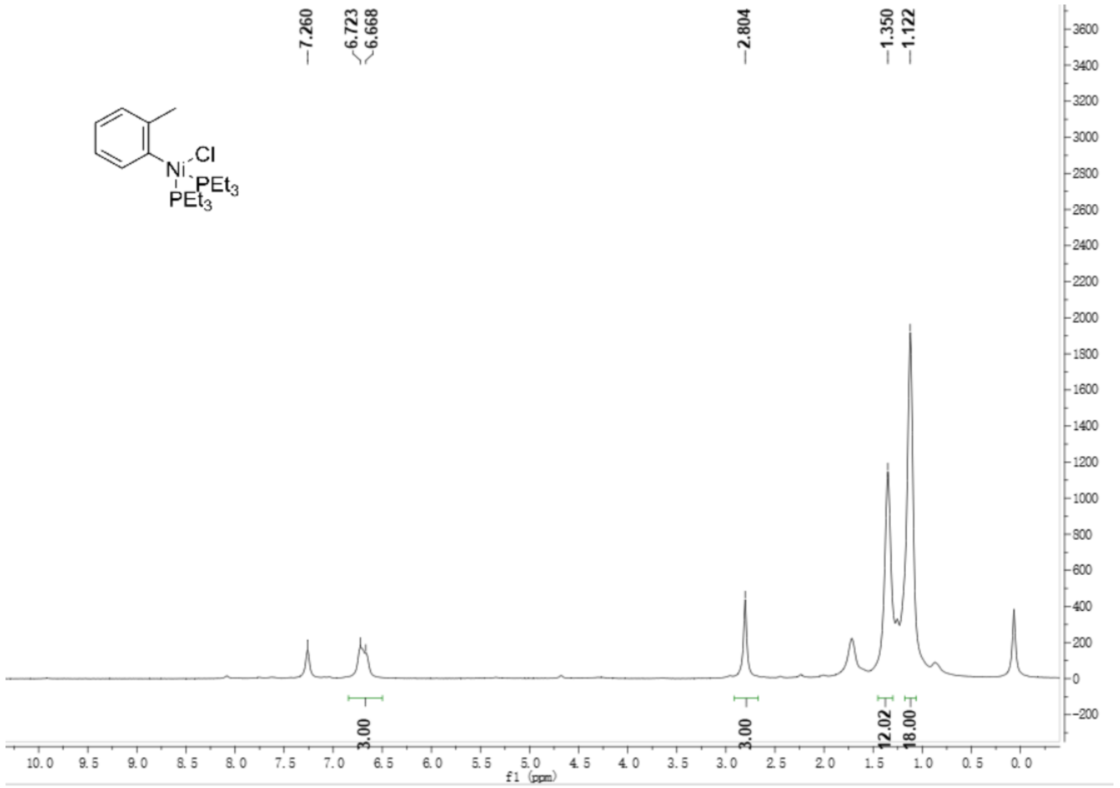


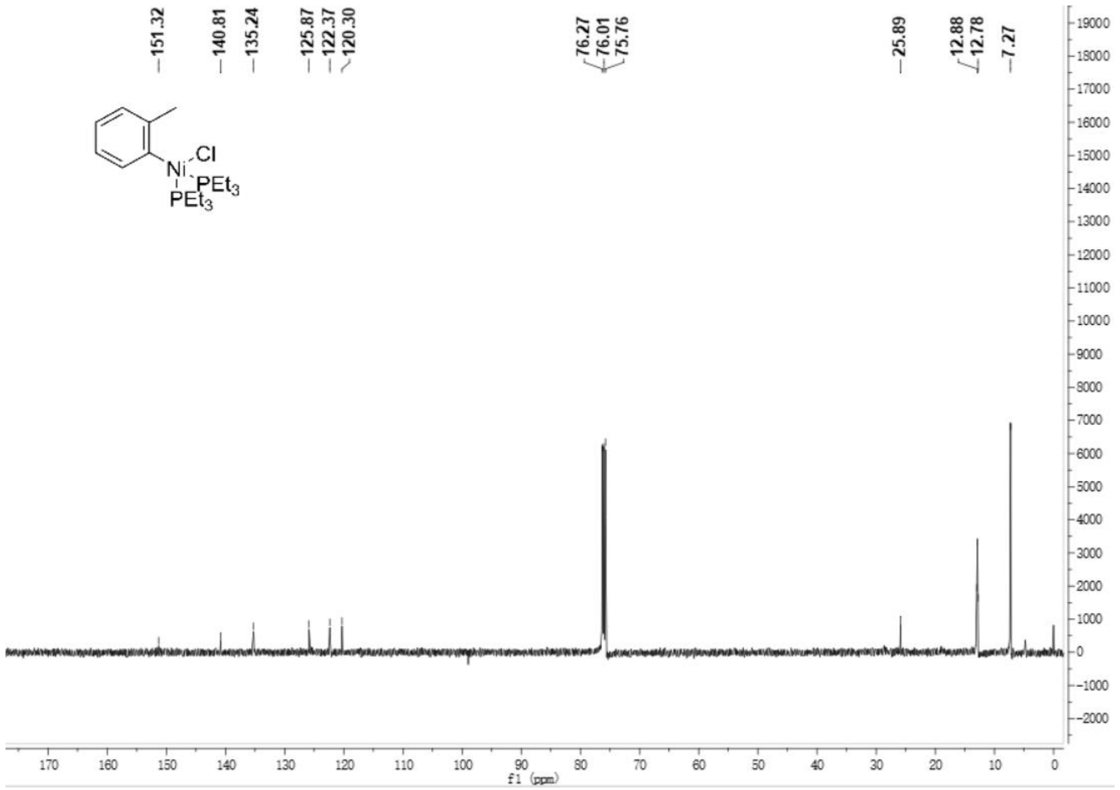

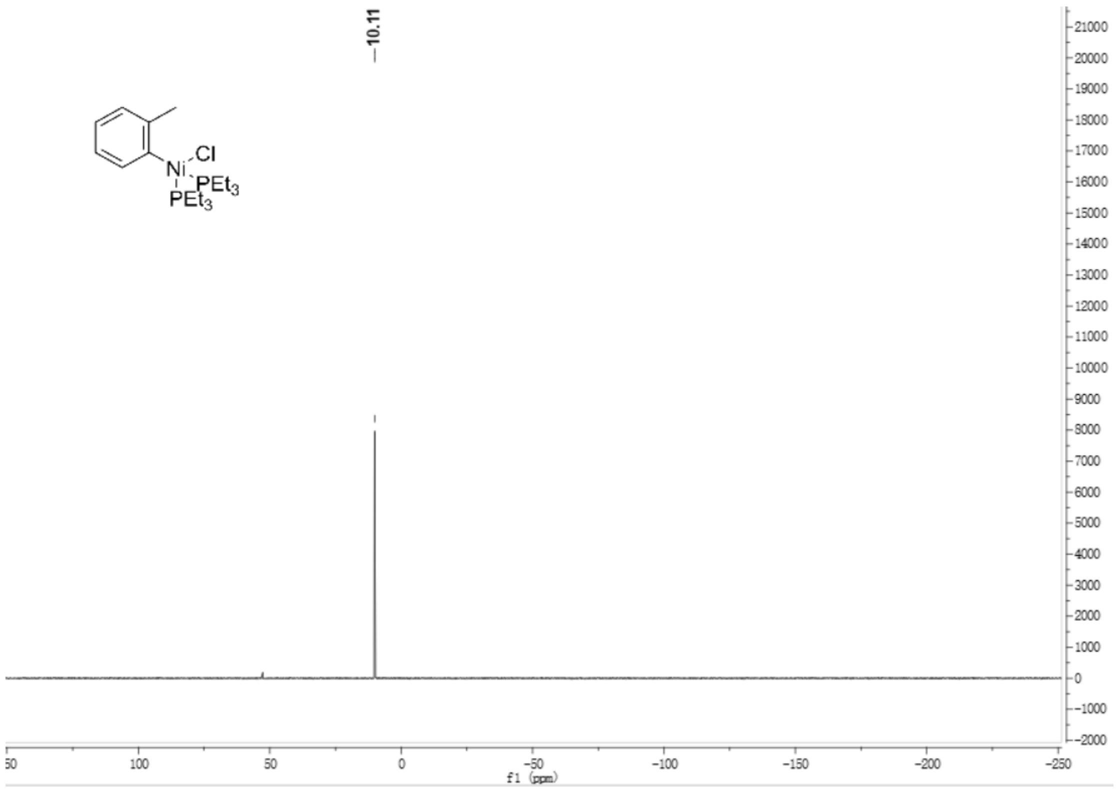


**1,4-diphenylbutan-1-one (12)**.

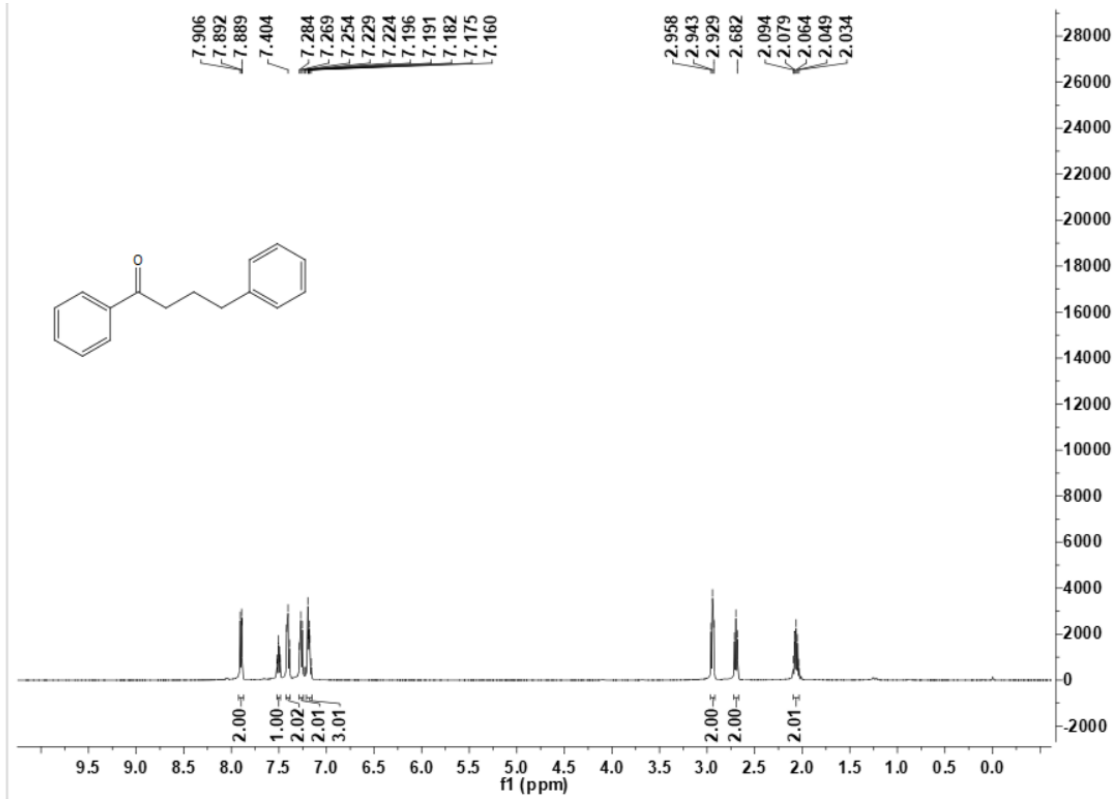

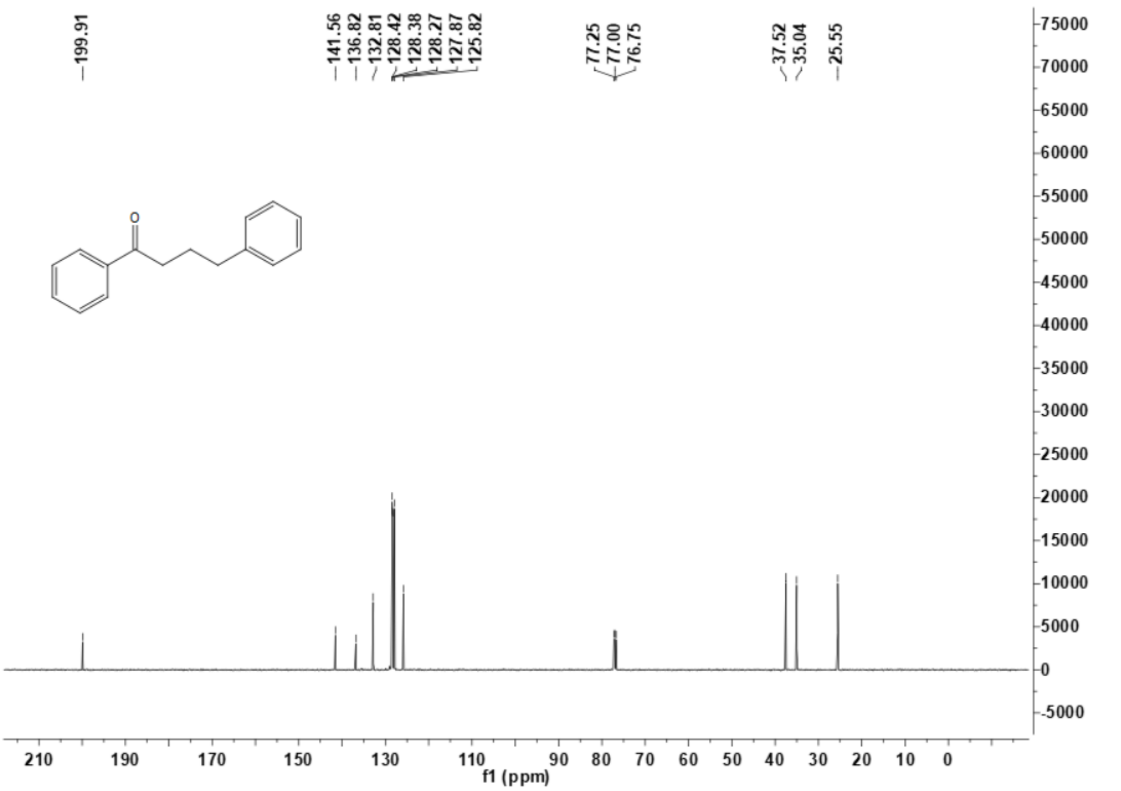


## 1-(4-methoxyphenyl)hept-2-yn-1-one (15)

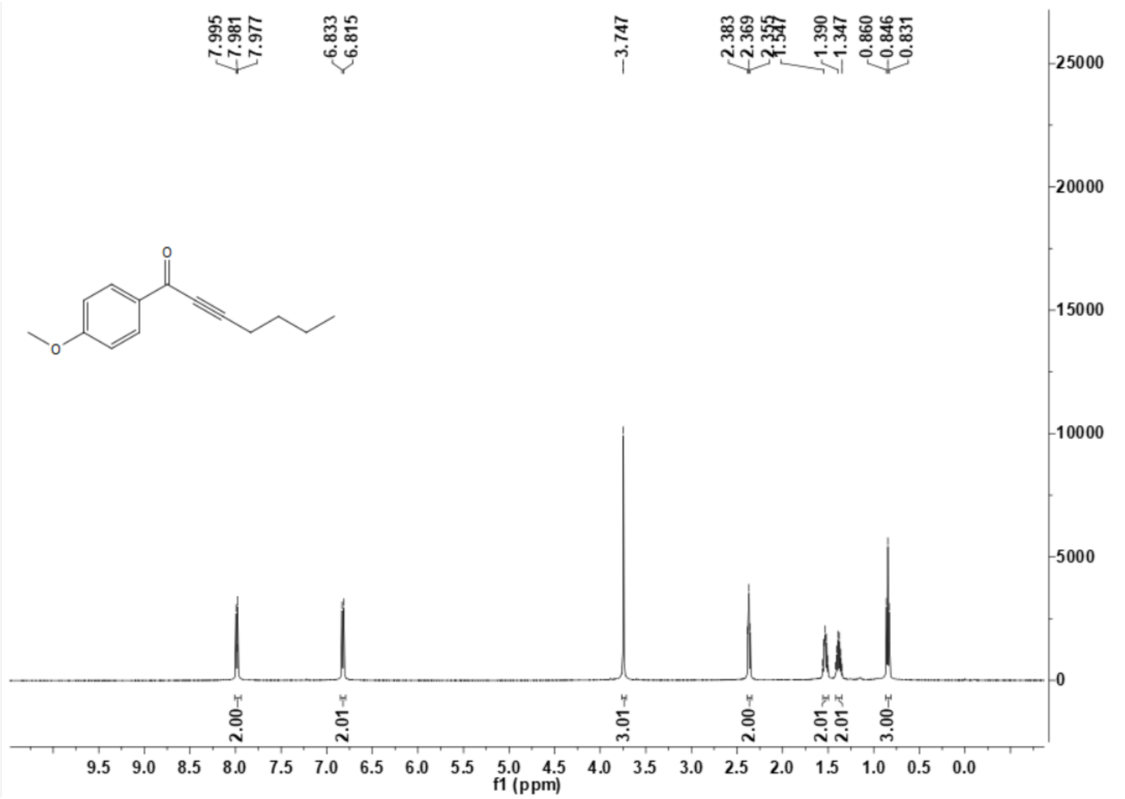

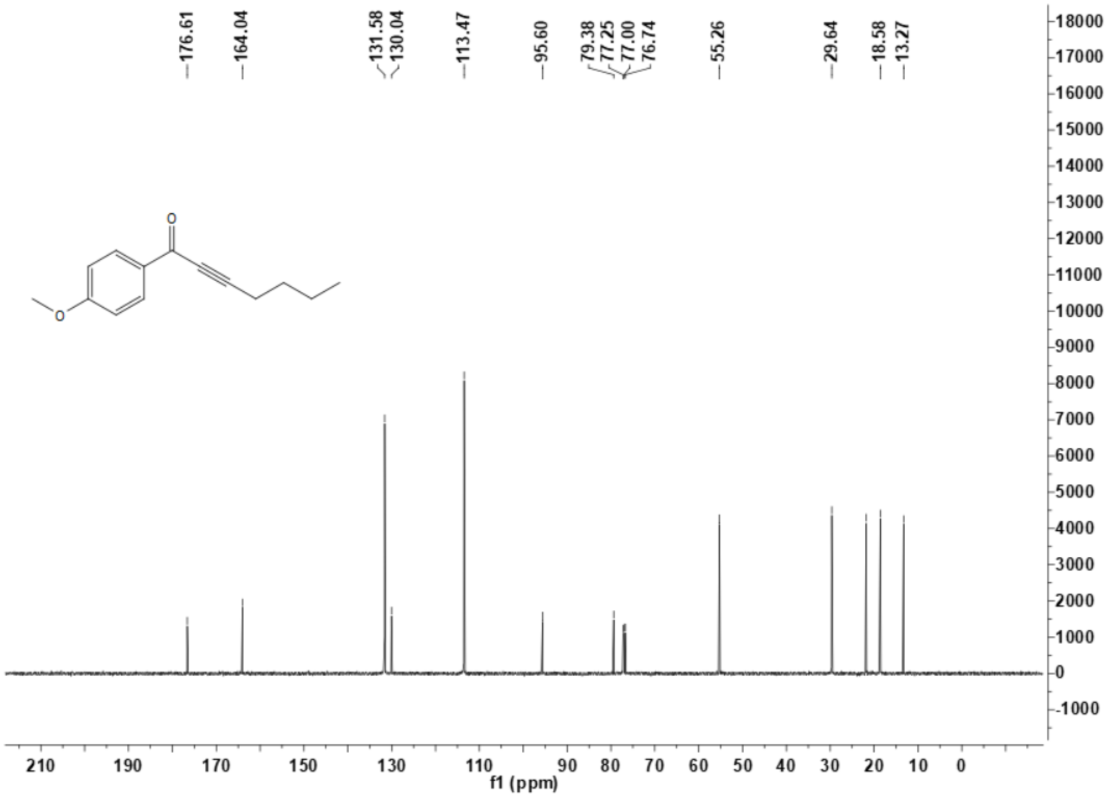


## Hex-5-en-1-ylbenzene (17)

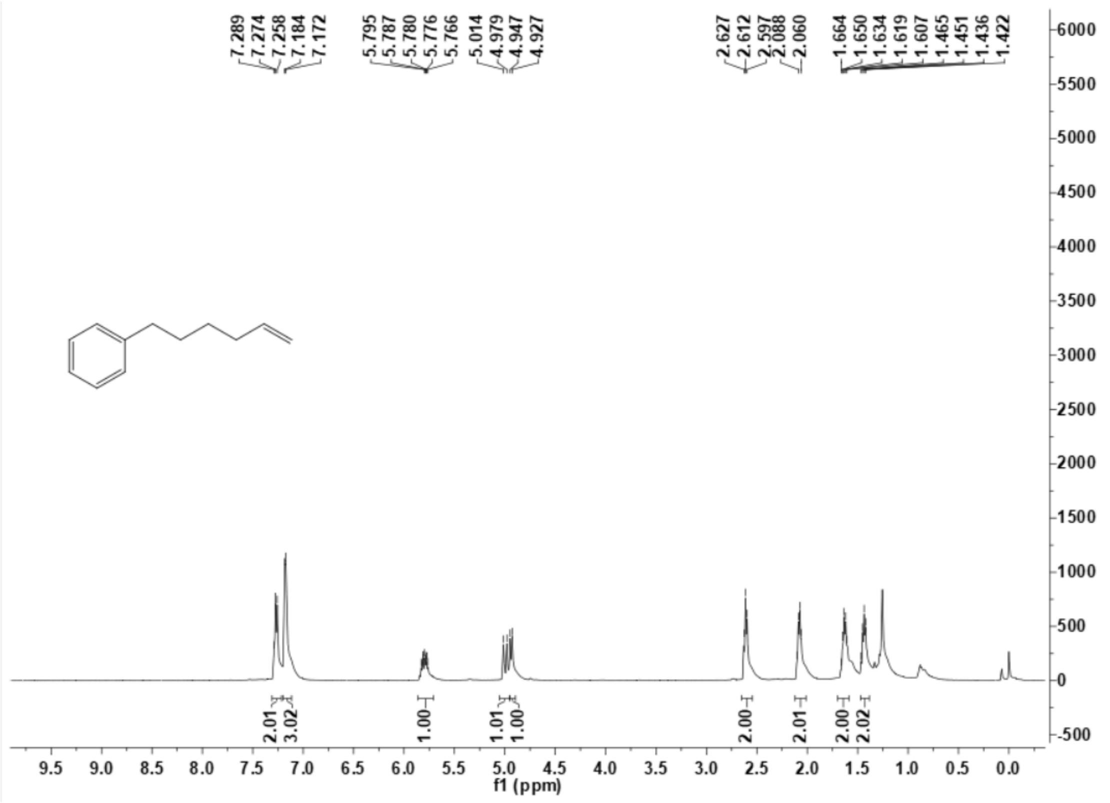

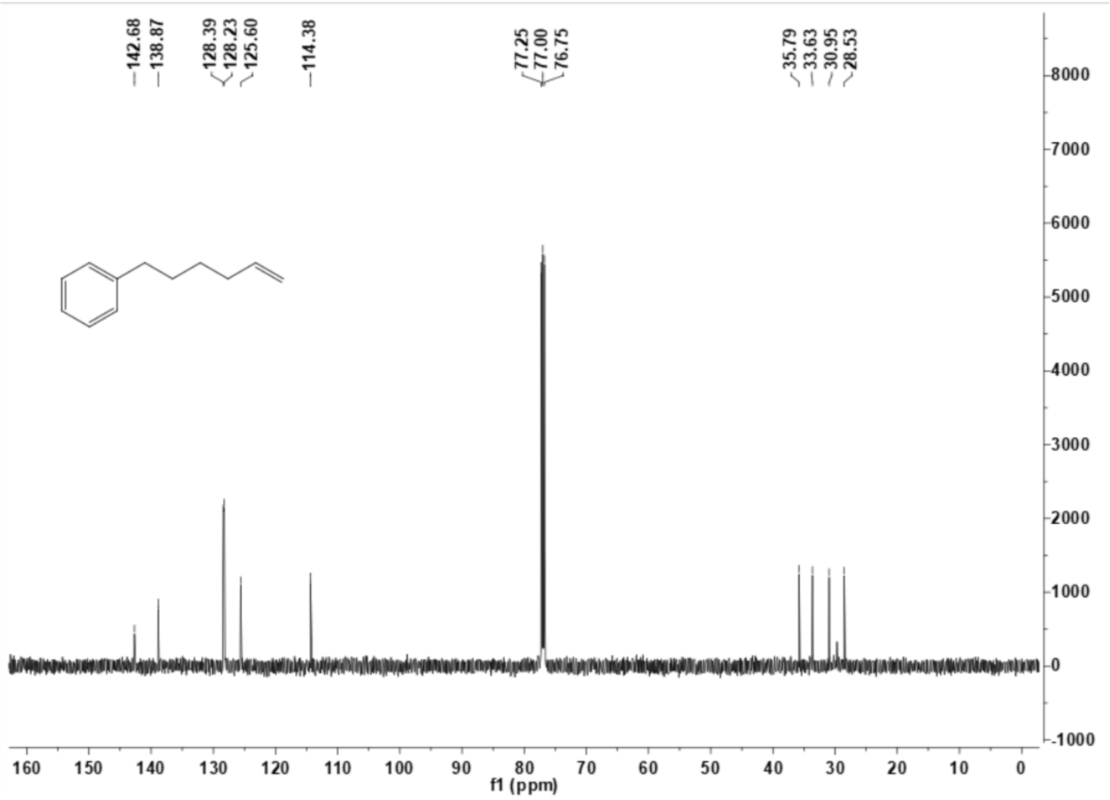


## (Cyclopentylmethyl)benzene (18)

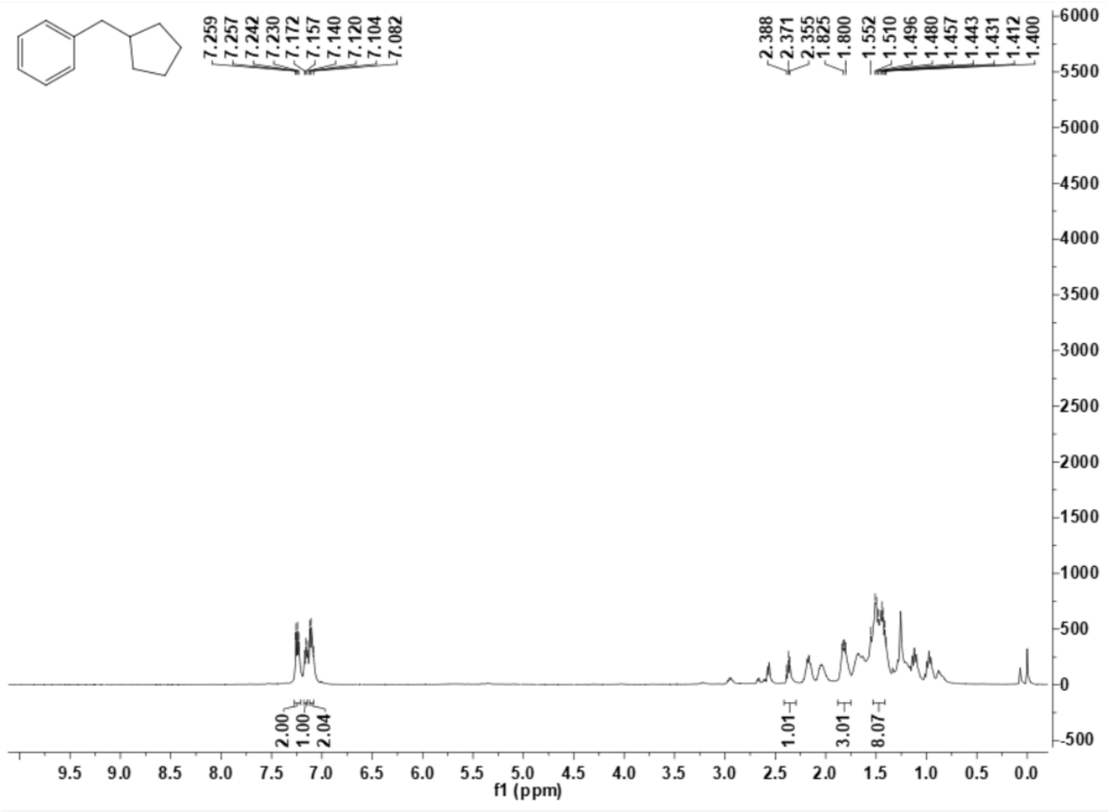

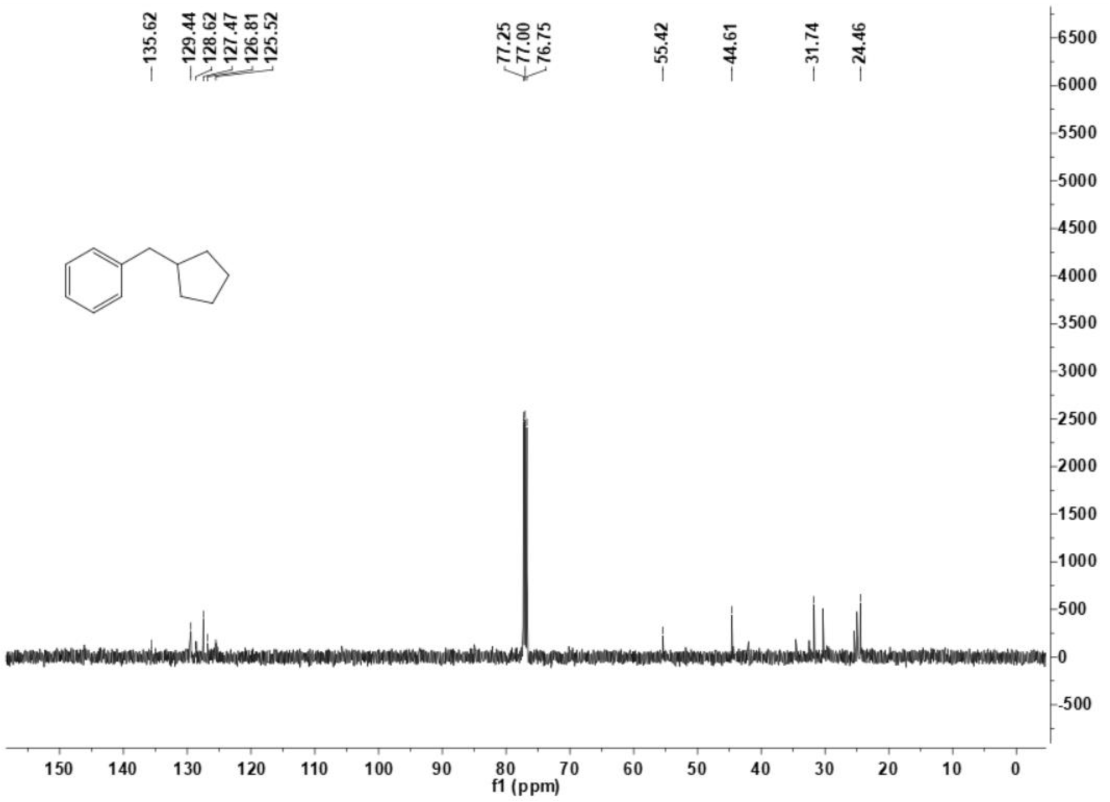


## But-3-en-1-ylbenzene (20)

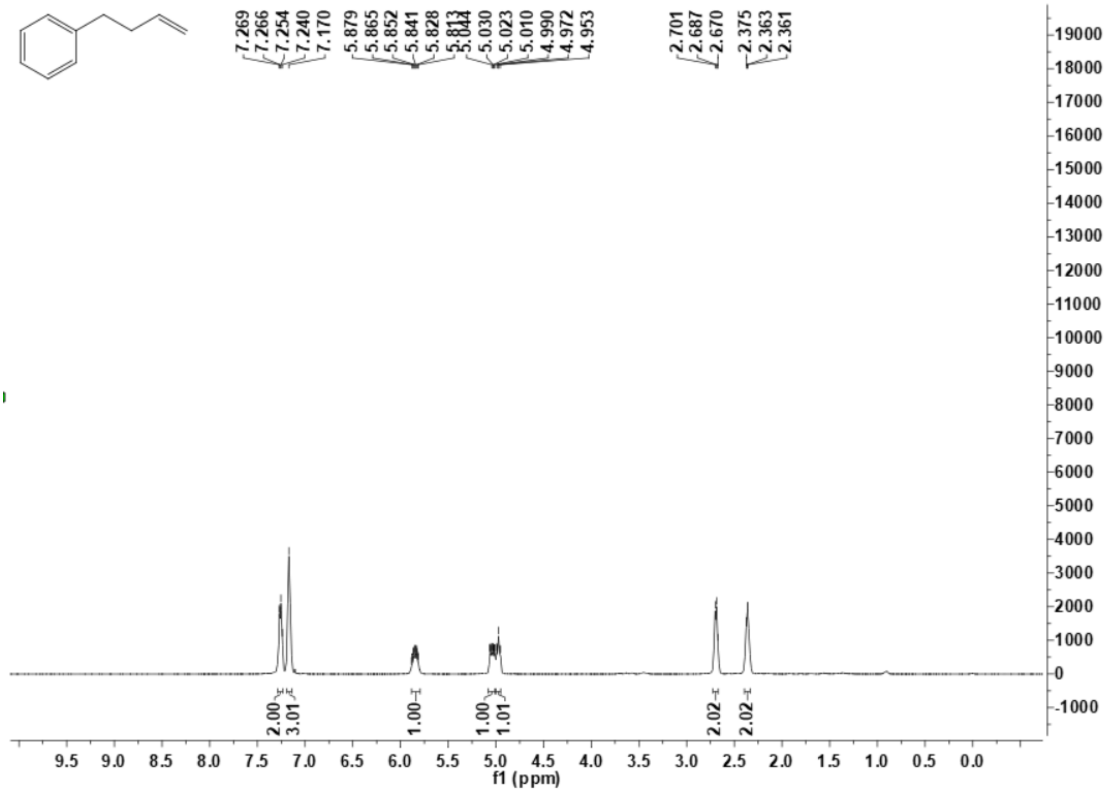

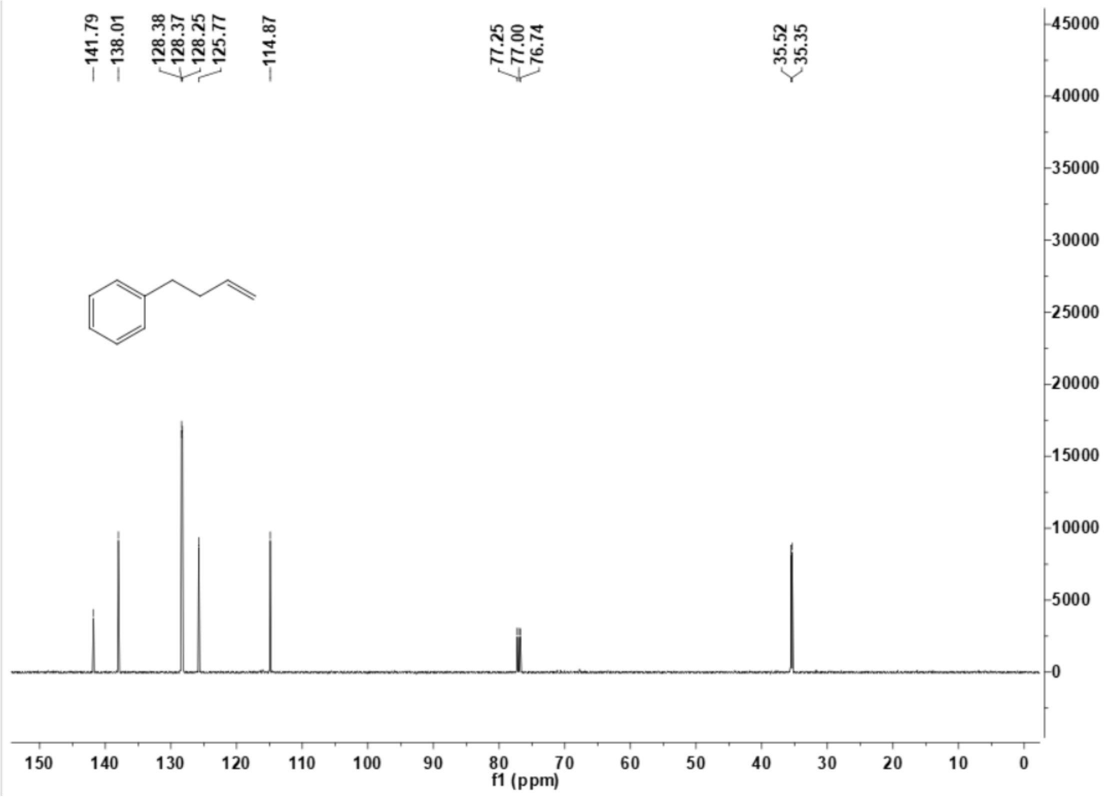

141.79
138.01
128.38
128.37
128.25
125.77
114.87
77.25
77.00
76.74
35.52
35.35
45000
40000
35000
30000
25000
20000
15000
10000
5000
0
150 140 130 120 110 100 90 80 70 60 50 40 30 20 10 0
f1 (ppm)
